# Supplementary material for: Radical-mediated C-C cleavage of unstrained cycloketones and DFT study for unusual regioselectivity
Source: Nat Commun. 2020 Feb 3;11:672. doi: 10.1038/s41467-020-14435-5 (PMC6997357; doi:10.1038/s41467-020-14435-5)
Supplement: Supplementary file 2 — Supplementary Information [file 41467_2020_14435_MOESM2_ESM.pdf]

Supplementary Information

Radical-Mediated C-C Cleavage of Unstrained Cycloketones and DFT Study for Unusual Regioselectivity

Wang et al.

Supplementary Methods

All reactions were maintained under a nitrogen atmosphere unless otherwise stated. Commercially available reagents were used without further purification. Infrared (FT-IR) spectra were recorded on a BRUKER VERTEX 70, ν_{max} in cm^{-1}. ^{1}H-NMR spectra were recorded on a BRUKER AVANCE III HD (400 MHz) spectrometer. Chemical shifts are reported in ppm from tetramethylsilane with the solvent resonance as internal standard ($CDCl_3$: δ 7.26). Data are reported as follows: chemical shift, multiplicity (s = singlet, d = doublet, t = triplet, q = quadruplet, br = broad, m = multiplet), coupling constants (Hz) and integration. ^{13}C-NMR spectra were recorded on a BRUKER AVANCE III HD (100 MHz) spectrometer with complete proton decoupling. Chemical shifts are reported in ppm from tetramethylsilane with the solvent resonance as the internal standard ($CDCl_3$: δ 77.16). ^{19}F-NMR spectra were recorded on a BRUKER AVANCE III HD (376 MHz) spectrometer. Mass spectra were measured with an Agilent Technologies 6120 Quadrupole LC/MS. High resolution mass spectrometry (HRMS) were measured with a GCT Premier^{TM} and BRUKER micrOTF-Q III. Melting points were measured using INESA WRR and values are uncorrected.

General procedure for ring opening of cycloketones:

Method A (for iodo-substrates): Cycloketones (**1**) (0.2 mmol, 1 equiv.) and AIBN (0.01 mmol, 5 mol %) were loaded in a reaction vial which was subjected to evacuation/ flushing with N_2 three times. Then TTMSS (0.3 mmol, 1.5 equiv.), H_2O (4 mmol, 20 equiv.) and $PhCF_3$ (2 mL) were added to the mixture which was then heated to 80 °C. After the reaction completion, the reaction mixture was concentrated *in vacuo*. Purification by flash column chromatography on silica gel afforded the desired product.

Method B (for bromo-substrates): Cycloketones (**1**) (0.2 mmol, 1 equiv.), NaBr (0.02 mmol, 10 mol %), K_2CO_3 (0.3 mmol, 1.5 equiv.), and $[Ir(dF(CF_3)ppy)_2(dtbbpy)]PF_6$ (5.2 mg, 6.0 μmol, 3 mol %) were loaded in a reaction vial which was subjected to evacuation/ flushing with N_2 three times. Then TTMSS (0.3 mmol, 1.5 equiv.), H_2O (0.2 mL), and acetonitrile (4 mL) were added to the mixture which was irradiated with 30 W blue LEDs. After the reaction completion, the crude reaction mixture was concentrated *in vacuo*. Purification by flash column chromatography on silica gel afforded the desired product.

General procedure for synthesis of starting materials:

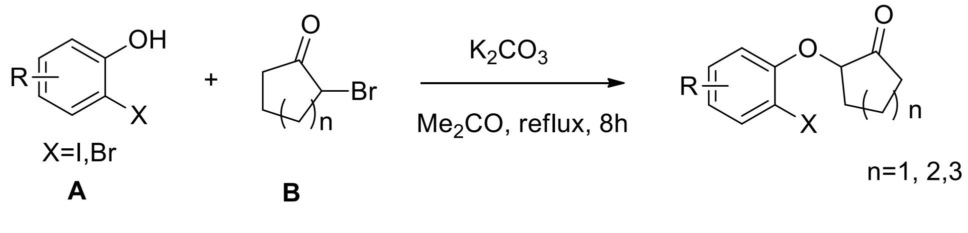

Supplementary Figure 1. General procedure 1 for synthesis of starting materials

o-Iodophenols (**A**) were conveniently synthesized from phenols according to the reported procedures.[1,2]

α-Bromocycloketones (**B**) were readily synthesized from cycloketones according to the reported procedures.[3,4]

Potassium carbonate (2.0 equiv.) was added to a solution of **A** (1.0 equiv.) and **B** (1.2 equiv.) in acetone (0.1 M), and the resulting mixture was heated under reflux for 8 h. The mixture was then allowed to cool to room temperature, filtered, and concentrated *in vacuo*. Purification by flash column chromatography afforded the desired product.

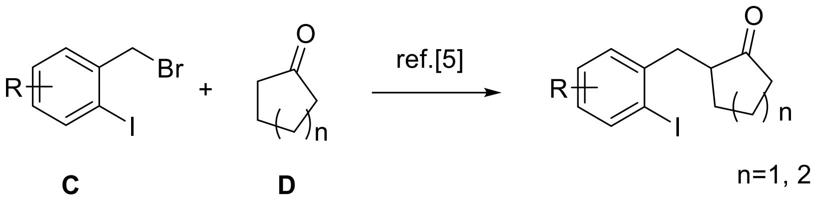

Supplementary Figure 2. General procedure 2 for synthesis of starting materials

A solution of LiHMDS (2.0 equiv., 1.0 M in THF) was added dropwise to a solution of cycloketones **D** (1.5 equiv.) in THF (0.5 M) at 0 °C under N_2. After the resulting mixture was stirred at 0 °C for 0.5 h, a solution of **C** (1.0 M in THF) was added dropwise at 0 °C. The reaction mixture was stirred at room temperature for 4 h, quenched with water, extracted with ethyl acetate, dried over Na_2SO_4, filtered, and concentrated in *vacuo*. Purification by flash column chromatography afforded the desired product.

Deuterium labelling experiments

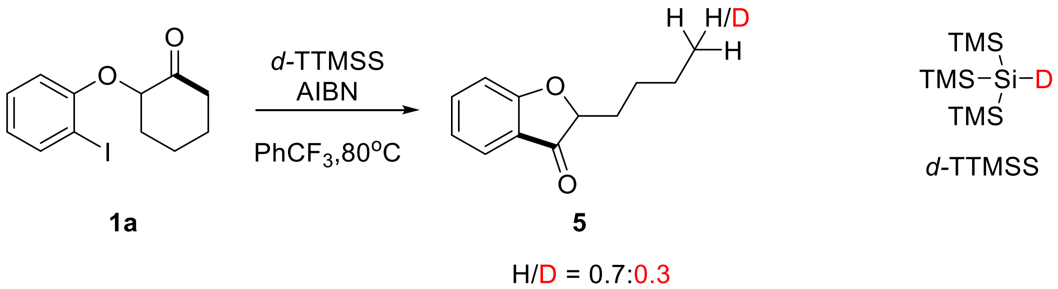

Supplementary Figure 3. Deuterium labelling experiments by using *d*-TTMSS

The reaction of **1a** in the presence of *d*-TTMSS delivered product **5** with H/D ratio as 0.7:0.3, indicating that the final step of H-abstraction could proceed via either the intramolecular 1,5-HAT or intermolecular H-abstraction from TTMSS.

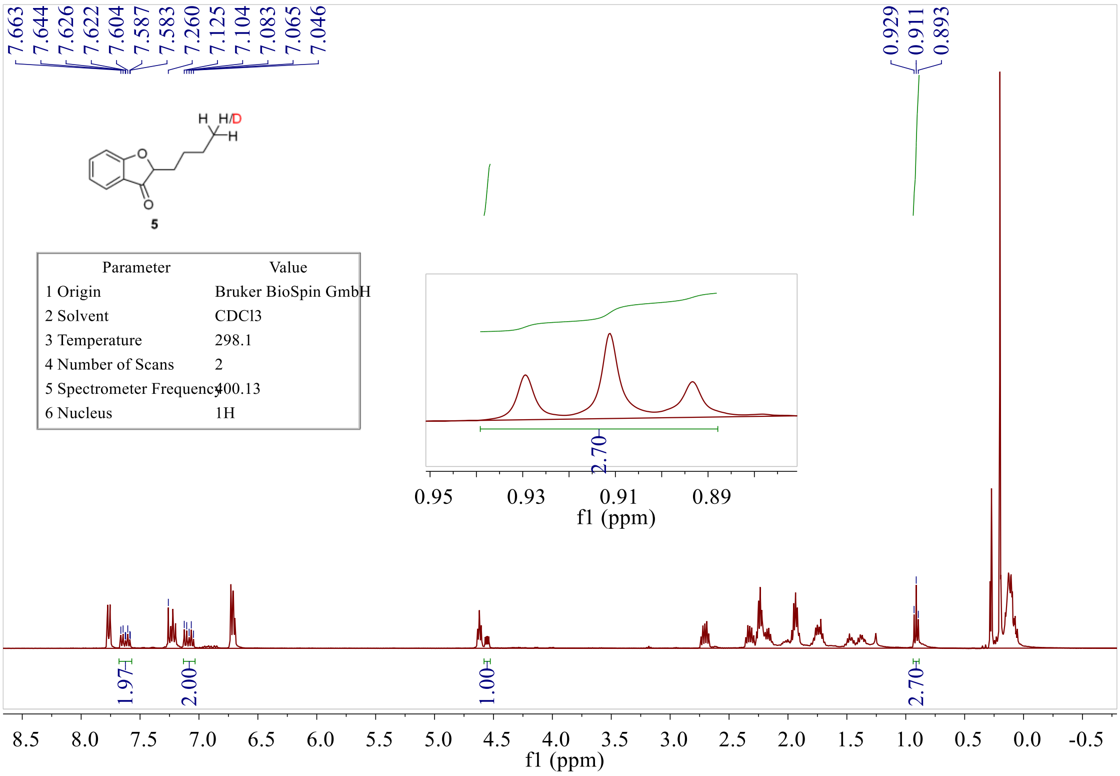

Supplementary Figure 4. ^{1}H NMR spectra of reaction

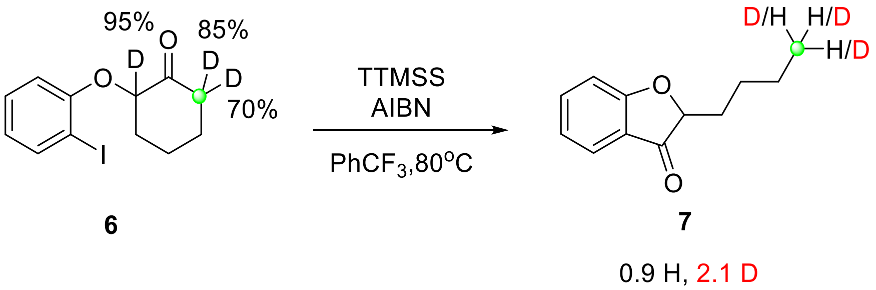

Supplementary Figure 5. Deuterium labelling experiments by using *d*-6

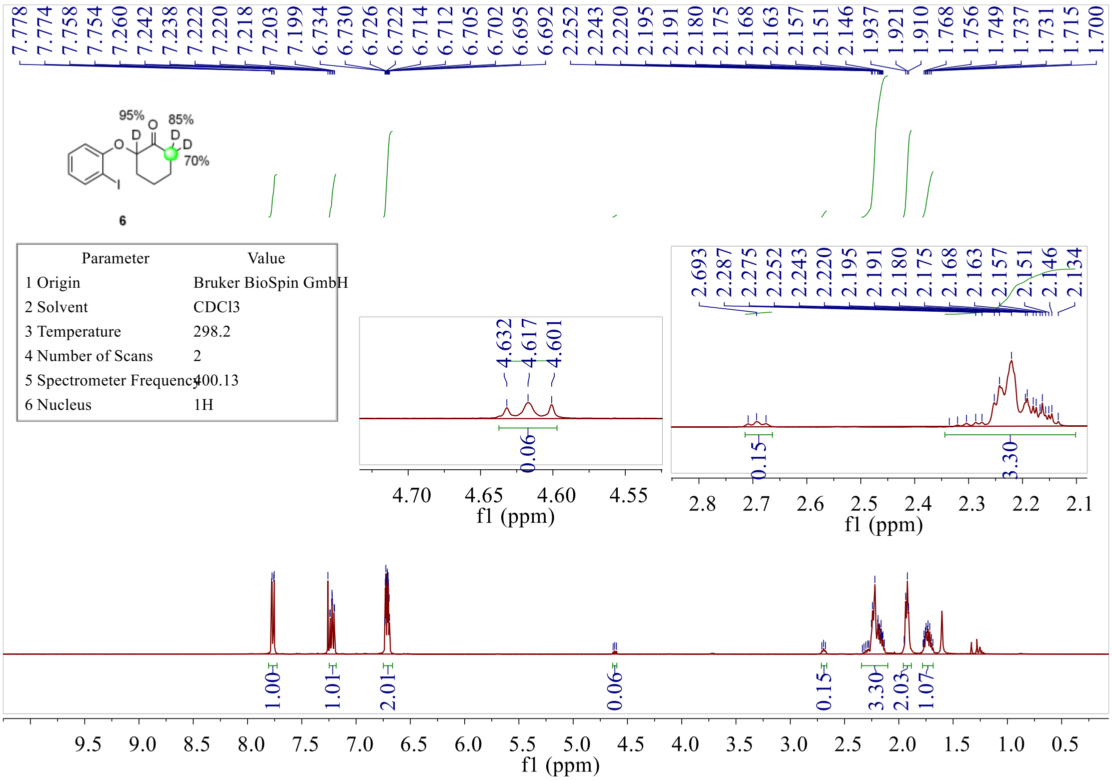

Supplementary Figure 6. ¹H NMR spectra of cyclohexanone 6

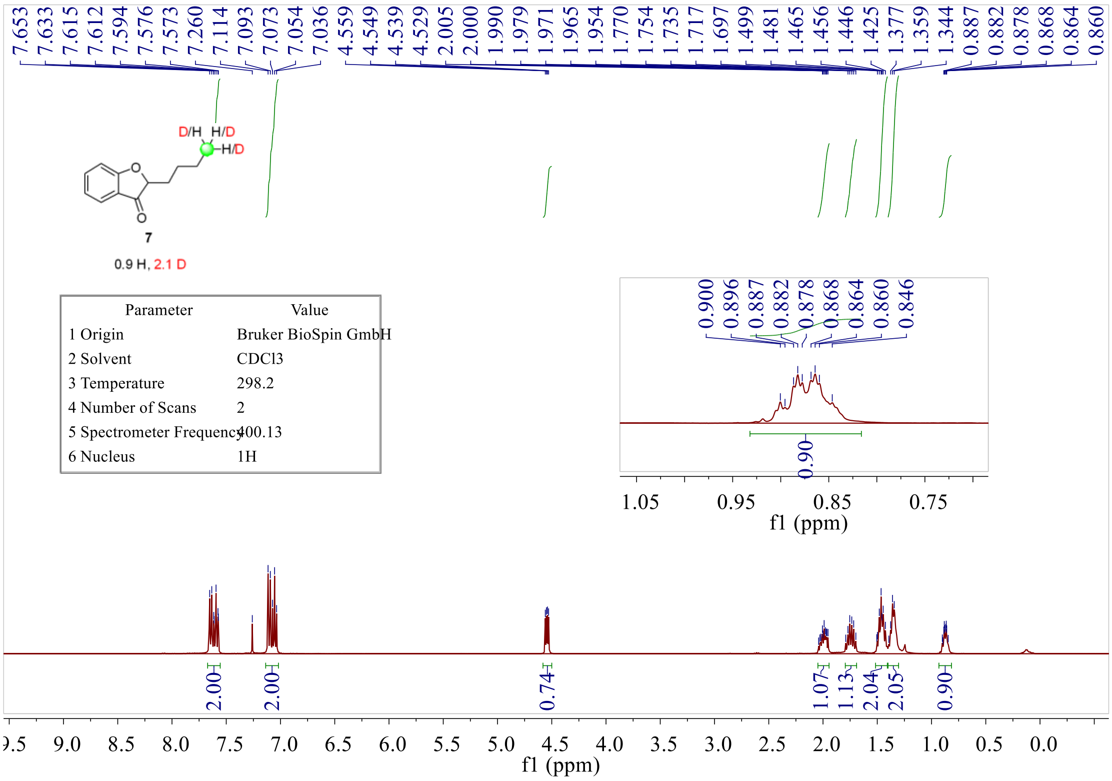

Supplementary Figure 7. ¹H NMR spectra of product 7

While employing the deuterated cyclohexanone **6** under the previous conditions, the counting of H and D in product **7** also suggested that the radical chain termination step could go through both two pathways.

Examining the influence of water

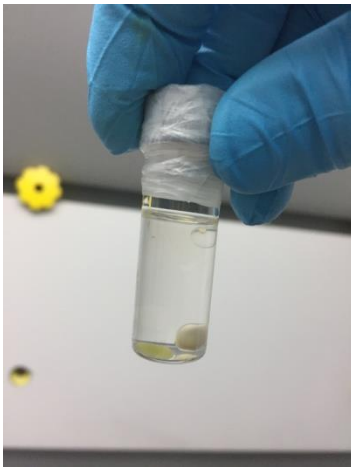

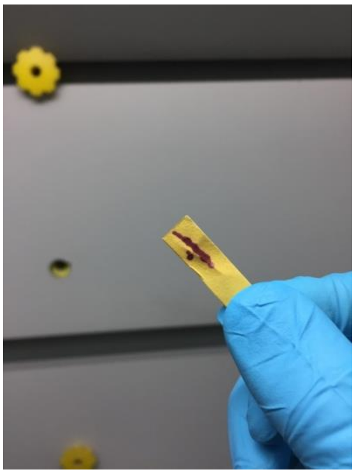

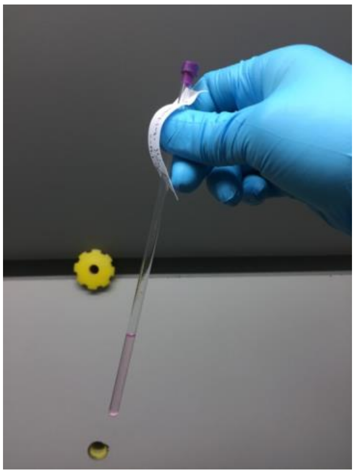

Supplementary Figure 8 **Supplementary Figure 9** **Supplementary Figure 10**

Supplementary Figure 8. Completion of reaction. A water drop precipitated in the reaction vial.

Supplementary Figure 9. The water drop is highly acidic (pH < 1), presumably the aqueous solution of HI which was obtained by hydrolysis of the by-product $(TMS)_3SiI$.

Supplementary Figure 10. Extraction of water drop with $CDCl_3$. A pink color might indicate the generation of iodine from air-oxidation of HI.

Reaction using TFA instead of water as additive: the reaction was significantly suppressed in the acidic conditions.

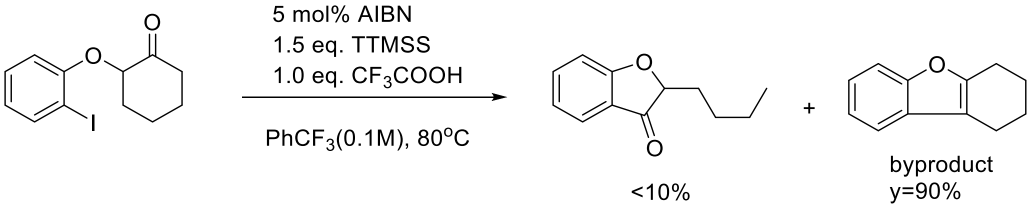

Supplementary Figure 11. Reaction using TFA instead of water as additive

Proposed mechanism for the formation of 2z

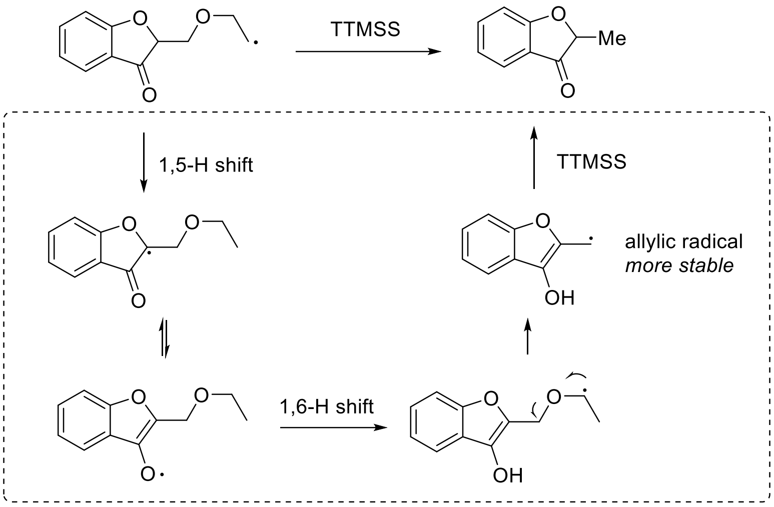

Supplementary Figure 12. Proposed mechanism for the formation of **2z**

Ring opening of cycloheptanone

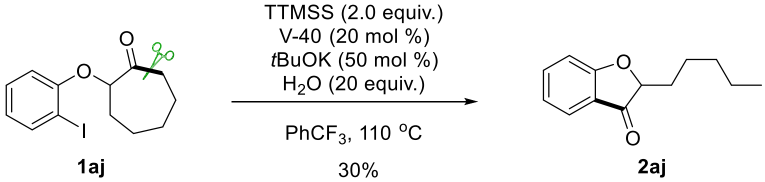

Supplementary Figure 13. Reaction with cycloheptanone

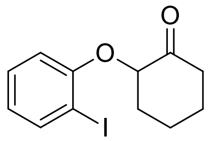

1a: white solid, m.p. 94-95 °C. ^{1}H NMR (400 MHz, $CDCl_3$) δ 7.79-7.74 (m, 1H), 7.25-7.19 (m, 1H), 6.74-6.68 (m, 2H), 4.62 (t, *J* = 6.0 Hz, 1H), 2.74-2.66 (m, 1H), 2.36-2.28 (m, 1H), 2.27-2.13 (m, 3H), 1.96-1.89 (m, 2H), 1.78-1.68 (m, 1H); ^{13}C NMR (100 MHz, $CDCl_3$) δ 207.9, 155.8, 139.1, 128.9, 122.9, 113.6, 86.7, 81.3, 39.5, 33.9, 27.5, 21.6. FT-IR: ν (cm^{-1}) 2949, 2927, 2863, 1719, 1579, 1468, 1447, 1440, 1429, 1272, 1243, 1078, 1018. HRMS [ESI] calcd for $C_{12}H_{13}INaO_2$ $[M+Na]^+$ 338.9852, found 338.9865.

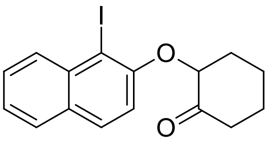

1b: white solid, m.p. 109-110 °C. ^{1}H NMR (400 MHz, $CDCl_3$) δ 8.15 (d, *J* = 8.4 Hz, 1H), 7.77-7.67 (m, 2H), 7.54 (t, *J* = 7.2 Hz, 1H), 7.43-7.34 (m, 1H), 7.10-7.02 (m, 1H), 4.78 (t, *J* = 6.4 Hz, 1H), 2.81-2.69 (m, 1H), 2.42-2.16 (m, 4H), 2.04-1.86 (m, 2H), 1.82-1.68(m, 1H); ^{13}C NMR (100 MHz, $CDCl_3$) δ 207.8, 154.5, 135.2, 131.0, 129.8, 129.7, 127.7, 127.7, 124.3, 115.1, 89.1, 82.2, 39.7, 34.1, 27.4, 21.8. FT-IR: ν (cm^{-1}) 3057, 2938, 2859, 1715, 1620, 1592, 1498, 1456, 1261, 1239, 1074. HRMS [ESI] calcd for $C_{16}H_{15}INaO_2$ $[M+Na]^+$ 389.0009, found 389.0015.

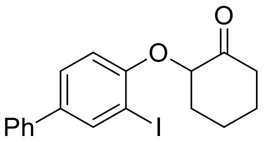

1c: white solid, m.p. 108-109 °C. ^{1}H NMR (400 MHz, $CDCl_3$) δ 8.01 (d, *J* = 2.0 Hz, 1H), 7.52-7.47 (m, 2H), 7.46-7.38 (m, 3H), 7.35-7.29 (m, 1H), 6.77 (d, *J* = 8.4 Hz, 1H), 4.69-4.64 (m, 1H), 2.77-2.66 (m, 1H),

2.42-2.31 (m, 1H), 2.30-2.14 (m, 3H), 2.00-1.91 (m, 2H), 1.81-1.69 (m, 1H); ^{13}C NMR (100 MHz, $CDCl_3$) δ 208.3, 155.7, 139.2, 138.1, 136.6, 128.8, 128.0, 127.3, 126.8, 114.0, 87.6, 81.9, 40.1, 34.4, 28.0, 22.2. FT-IR: ν (cm^{-1}) 2954, 2939, 2861, 1719, 1593, 1475, 1463, 1270, 1246, 1133, 1083, 1013. HRMS [ESI] calcd for $C_{18}H_{17}INaO_2$ $[M+Na]^+$ 415.0165, found 415.0174.

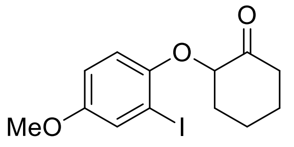

1d: white solid, m.p. 83-85 °C. ^{1}H NMR (400 MHz, $CDCl_3$) δ 7.30 (d, *J* = 2.8 Hz, 1H), 6.80-6.75 (m, 1H), 6.74-6.71 (m, 1H), 4.50 (ddd, *J* = 8.0, 4.8, 0.8 Hz, 1H), 3.73 (s, 3H), 2.73-2.63 (m, 1H), 2.35-2.09 (m, 4H), 1.98-1.85 (m, 2H), 1.78-1.64 (m, 1H); ^{13}C NMR (100 MHz, $CDCl_3$) δ 208.2, 154.5, 150.4, 124.1, 115.3, 114.3, 87.3, 82.5, 55.4, 39.6, 34.0, 27.4, 21.8. FT-IR: ν (cm^{-1}) 3002, 2938, 2860, 1717, 1487, 1459, 1334, 1290, 1216, 1207, 1027. HRMS [ESI] calcd for $C_{13}H_{15}INaO_3$ $[M+Na]^+$ 368.9958, found 368.9963.

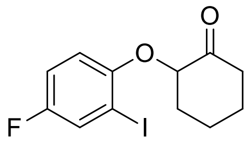

1e: white solid, m.p. 67-68 °C. ^{1}H NMR (400 MHz, $CDCl_3$) δ 7.48 (dd, *J* = 7.6, 3.2 Hz, 1H), 6.97-6.91 (m, 1H), 6.69 (dd, *J* = 9.2, 4.8 Hz, 1H), 4.54 (ddd, *J* = 8.4, 4.8, 0.8 Hz, 1H), 2.70-2.62 (m, 1H), 2.37-2.24 (m, 2H), 2.23-2.10 (m, 2H), 1.99-1.85 (m, 2H), 1.80-1.68 (m, 1H); ^{13}C NMR (100 MHz, $CDCl_3$) δ 208.1, 157.2 (d, J_{C-F} = 243.4 Hz), 153.1 (d, J_{C-F} = 2.6 Hz), 126.1 (d, J_{C-F} = 24.9 Hz), 115.6 (d, J_{C-F} = 22.7 Hz), 114.8 (d, J_{C-F} = 8.2 Hz), 86.8 (d, J_{C-F} = 8.5 Hz), 82.7, 40.1, 34.4, 27.8, 22.3; ^{19}F NMR (376 MHz, $CDCl_3$) δ -120.8 (s). FT-IR: ν (cm^{-1}) 2951, 2863, 1714, 1594, 1480, 1283, 1259, 1194, 1133, 1109, 1036. HRMS [ESI] calcd for $C_{12}H_{12}FINaO_2$ $[M+Na]^+$ 356.9758, found 356.9758.

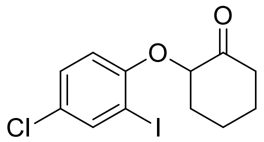

1f: white solid, m.p. 85-87 °C. ^{1}H NMR (400 MHz, $CDCl_3$) δ 7.73 (d, *J* = 2.4 Hz, 1H), 7.18 (dd, *J* = 8.8, 2.8 Hz, 1H), 6.61 (d, *J* = 8.8 Hz, 1H), 4.57 (dd, *J* = 8.0, 7.2 Hz, 1H), 2.71-2.60 (m, 1H), 2.38-2.10 (m, 4H), 2.01-1.84 (m, 2H), 1.80-1.68 (m, 1H); ^{13}C NMR (100 MHz, $CDCl_3$) δ 207.8, 155.3, 138.7, 129.1, 127.2, 114.5, 87.3, 82.2, 40.1, 34.3, 27.8, 22.2. FT-IR: ν (cm^{-1}) 2983, 2956, 2938, 2860, 1716, 1578, 1497, 1473, 1430, 1366, 1309, 1246. HRMS [ESI] calcd for $C_{12}H_{12}ClINaO_2$ $[M+Na]^+$ 372.9463, found 372.9470.

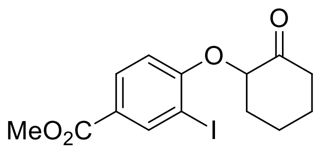

1g: white solid, m.p. 122-123 °C. ^{1}H NMR (400 MHz, $CDCl_3$) δ 8.44 (d, *J* = 2.0 Hz, 1H), 7.90 (dd, *J* = 8.4, 2.0 Hz, 1H), 6.63 (d, *J* = 8.4 Hz, 1H), 4.71 (dd, *J* = 8.4, 4.8 Hz, 1H), 3.86 (s, 3H), 2.70-2.61 (m, 1H), 2.40-2.11 (m, 4H), 2.02-1.86 (m, 2H), 1.81-1.69 (m, 1H); ^{13}C NMR (100 MHz, $CDCl_3$) δ 206.6, 164.9, 159.3, 140.7, 130.8, 124.4, 111.8, 85.7, 81.2, 51.7, 39.6, 33.8, 27.3, 21.8. FT-IR: ν (cm^{-1}) 2979, 2950, 2861, 1715, 1593, 1487, 1430, 1392, 1303, 1270, 1256, 1108, 1034. HRMS [ESI] calcd for $C_{14}H_{15}INaO_4$ $[M+Na]^+$ 396.9907, found 396.9920.

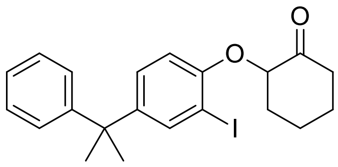

1h: colorless oil. ^{1}H NMR (400 MHz, $CDCl_3$) δ 7.65 (d, *J* = 2.4 Hz, 1H), 7.31-7.25 (m, 2H), 7.23-7.15 (m, 3H), 7.04 (dd, *J* = 8.8, 2.4 Hz, 1H), 6.60 (d, *J* = 8.8 Hz, 1H), 4.61-4.55 (m, 1H), 2.75-2.66 (m, 1H), 2.38-2.27 (m, 1H), 2.27-2.11 (m, 3H), 1.98-1.89 (m, 2H), 1.79-1.69 (m, 1H), 1.63 (s, 6H); ^{13}C NMR

(100 MHz, $CDCl_3$) δ 208.5, 154.2, 150.0, 146.0, 137.7, 128.1, 126.7, 125.8, 113.4, 86.9, 81.9, 42.2, 40.1, 34.5, 30.8, 30.8, 28.0, 22.2. FT-IR: ν (cm^{-1}) 3084, 2964, 2934, 1724, 1594, 1480, 1445, 1284, 1246, 1080, 1067, 1031. HRMS [ESI] calcd for $C_{21}H_{23}INaO_2$ $[M+Na]^+$ 457.0635 found 457.0644.

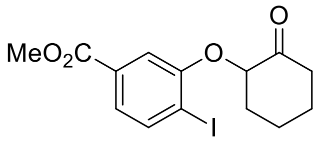

1i: white solid, m.p. 137-138 °C. ^1H NMR (400 MHz, $CDCl_3$) δ 7.84 (d, J = 8.0 Hz, 1H), 7.35 (dd, J = 8.4, 1.2 Hz, 1H), 7.27-7.23 (m, 1H), 4.75 (dd, J = 9.2, 5.2 Hz, 1H), 3.87 (s, 3H), 2.70-2.61 (m, 1H), 2.45-2.28 (m, 2H), 2.22-2.05 (m, 2H), 2.05-1.94 (m, 1H), 1.91-1.67 (m, 2H); ^{13}C NMR (100 MHz, $CDCl_3$) δ 206.9, 166.4, 156.5, 139.7, 131.4, 124.0, 113.7, 93.8, 81.7, 52.4, 40.4, 34.2, 27.6, 22.7. FT-IR: ν (cm^{-1}) 2953, 2873, 1710, 1568, 1472, 1454, 1428, 1303, 1259, 1230, 1126, 1076. HRMS [ESI] calcd for $C_{14}H_{15}INaO_4$ $[M+Na]^+$ 396.9907, found 396.9915.

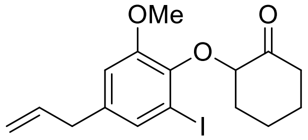

1j: colorless oil. ^1H NMR (400 MHz, $CDCl_3$) δ 7.18-7.13 (m, 1H), 6.67-6.61 (m, 1H), 5.94-5.81 (m, 1H), 5.11-5.03 (m, 2H), 4.57-4.49 (m, 1H), 3.72 (s, 3H), 3.26 (d, J = 6.4 Hz, 2H), 2.93-2.75 (m, 1H), 2.41-2.18 (m, 2H), 2.16-1.95 (m, 2H), 1.96-1.78 (m, 2H), 1.69-1.54 (m, 1H); ^{13}C NMR (100 MHz, $CDCl_3$) δ 207.3, 151.2, 144.8, 137.4, 136.1, 130.2, 116.0, 112.9, 92.2, 84.6, 55.3, 39.8, 38.8, 34.4, 27.2, 21.8. FT-IR: ν (cm^{-1}) 3076, 2938, 2863, 1724, 1590, 1561, 1461, 1431, 1410, 1308, 1268, 1230, 1145, 1040. HRMS [ESI] calcd for $C_{16}H_{19}INaO_3$ $[M+Na]^+$ 409.0271, found 409.0279.

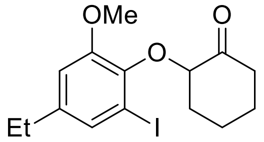

1k: yellow oil. ^1H NMR (400 MHz, $CDCl_3$) δ 7.18-7.14 (m, 1H), 6.67-6.63 (m, 1H), 4.55-4.47 (m, 1H), 3.73 (s, 3H), 2.92-2.81 (m, 1H), 2.52 (q, J = 7.6 Hz, 2H), 2.38-2.23 (m, 2H), 2.14-2.00 (m, 2H), 1.91-1.80 (m, 2H), 1.68-1.55 (m, 1H), 1.18 (t, J = 7.6 Hz, 3H); ^{13}C NMR (100 MHz, $CDCl_3$) δ 207.8, 151.6, 144.9, 142.1, 130.0, 112.8, 92.6, 85.1, 55.8, 40.2, 34.8, 28.1, 27.7, 22.3, 15.4. FT-IR: ν (cm^{-1}) 2961, 2936, 2864, 1723, 1591, 1560, 1460, 1432, 1411, 1300, 1269, 1228, 1146, 1058, 1041. HRMS [ESI] calcd for $C_{15}H_{19}INaO_3$ $[M+Na]^+$ 397.0271, found 397.0282.

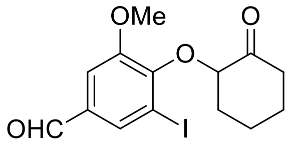

1l: yellow soild, m.p. 90-91 °C. ^1H NMR (400 MHz, $CDCl_3$) δ 9.78 (s, 1H), 7.84 (d, J = 1.6 Hz, 1H), 7.35 (d, J = 1.6 Hz, 1H), 4.96 (ddd, J = 10.0, 5.6, 1.2 Hz, 1H), 3.81 (s, 3H), 2.77-2.67 (m, 1H), 2.40-2.29 (m, 1H), 2.29-2.14 (m, 2H), 2.07-1.92 (m, 2H), 1.85-1.76 (m, 1H), 1.74-1.64 (m, 1H); ^{13}C NMR (100 MHz, $CDCl_3$) δ 206.1, 189.1, 151.6, 151.1, 134.8, 132.8, 110.7, 91.6, 84.4, 55.6, 39.8, 34.5, 27.0, 22.3. FT-IR: ν (cm^{-1}) 2938, 1726, 1684, 1584, 1560, 1507, 1456, 1410, 1381, 1229, 1142, 1037. HRMS [ESI] calcd for $C_{14}H_{15}INaO_4$ $[M+Na]^+$ 396.9907, found 396.9919.

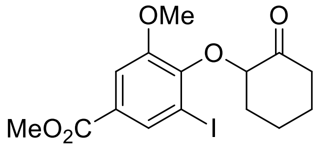

1m: white solid, 105-107 °C. ^1H NMR (400 MHz, $CDCl_3$) δ 8.09-8.04 (m, 1H), 7.52-7.48 (m, 1H), 4.84 (t, J = 7.2, 1H), 3.88 (s, 3H), 3.81 (s, 3H), 2.84-2.74 (m, 1H), 2.43-2.29 (m, 1H), 2.26-2.14 (m, 2H), 2.07-1.99 (m, 1H), 1.99-1.89 (m, 1H), 1.89-1.74 (m, 1H),

1.74-1.59 (m, 1H); ^{13}C NMR (100 MHz, $CDCl_3$) δ 206.2, 164.8, 150.3, 150.1, 132.3, 126.3, 113.3, 91.3, 84.3, 55.6, 51.8, 39.8, 34.4, 27.0, 22.2. FT-IR: ν (cm^{-1}) 2939, 2870, 2854, 1727, 1705, 1585, 1561, 1467, 1432, 1318, 1290, 1176, 1037. HRMS [ESI] calcd for $C_{15}H_{17}INaO_5$ $[M+Na]^+$ 427.0013, found 427.0024.

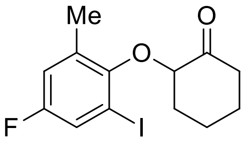

1n: white solid, m.p. 51-52 ºC. ^{1}H NMR (400 MHz, $CDCl_3$) δ 7.32-7.27 (m, 1H), 6.87-6.80 (m, 1H), 4.65-4.58 (m, 1H), 2.68-2.56 (m, 1H), 2.43-2.29 (m, 1H), 2.29-2.17 (m, 4H), 2.07-1.90 (m, 3H), 1.80-1.55 (m, 2H); ^{13}C NMR (100 MHz, $CDCl_3$) δ 206.2, 158.2 (d, J_{C-F} = 246.0 Hz), 152.1 (d, J_{C-F} = 3.0 Hz), 133.8 (d, J_{C-F} = 8.1 Hz), 124.0 (d, J_{C-F} = 24.7 Hz), 118.0 (d, J_{C-F} = 22.0 Hz), 90.5 (d, J_{C-F} = 9.2 Hz), 85.4, 41.0, 34.5, 27.3, 23.6, 17.9; ^{19}F NMR (376 MHz, $CDCl_3$) δ -118.3 (s). FT-IR: ν (cm^{-1}) 2957, 2942, 2871, 1709, 1598, 1574, 1471, 1432, 1206, 1194, 1128, 1049. HRMS [ESI] calcd for $C_{13}H_{14}FINaO_2$ $[M+Na]^+$ 370.9915, found 370.9920.

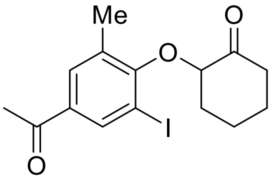

1o: white solid, m.p. 92-93 ºC. ^{1}H NMR (400 MHz, $CDCl_3$) δ 8.17 (s, 1H), 7.71 (s, 1H), 4.79 (dd, J = 11.2, 5.6 Hz, 1H), 2.66-2.56 (m, 1H), 2.51 (s, 3H), 2.41-2.19 (m, 5H), 2.10-1.90 (m, 3H), 1.83-1.59 (m, 2H); ^{13}C NMR (100 MHz, $CDCl_3$) δ 205.8, 195.9, 159.7, 138.3, 134.3, 132.9, 131.8, 91.1, 85.2, 41.0, 34.7, 27.2, 26.5, 23.6, 17.8. FT-IR: ν (cm^{-1}) 2944, 2862, 1731, 1692, 1676, 1554, 1463, 1356, 1245, 1051, 1024. HRMS [ESI] calcd for $C_{15}H_{17}INaO_3$ $[M+Na]^+$ 395.0115, found 395.0130.

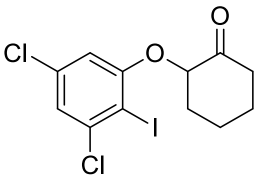

1p: white solid, m.p. 125-126 ºC. ^{1}H NMR (400 MHz, $CDCl_3$) δ 7.12 (d, J = 2.0 Hz, 1H), 6.51 (d, J = 2.0 Hz, 1H), 4.66-4.95 (m, 1H), 2.71-2.62 (m, 1H), 2.43-2.27 (m, 2H), 2.27-2.09 (m, 2H), 2.06-1.94 (m, 1H), 1.95-1.83 (m, 1H), 1.82-1.69 (m, 1H); ^{13}C NMR (100 MHz, $CDCl_3$) δ 206.7, 158.4, 140.4, 135.3, 122.3, 111.9, 90.2, 82.3, 40.2, 34.3, 27.7, 22.5. FT-IR: ν (cm^{-1}) 2943, 2864, 1717, 1561, 1446, 1398, 1377, 1361, 1214, 1072, 1018. HRMS [ESI] calcd for $C_{12}H_{11}Cl_2INaO_2$ $[M+Na]^+$ 406.9073, found 406.9084.

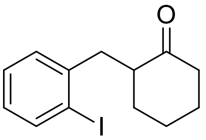

1q: colorless oil. ^{1}H NMR (400 MHz, $CDCl_3$) δ 7.82-7.77 (m, 1H), 7.25-7.20 (m, 2H), 6.91-6.82 (m, 1H), 3.34 (dd, J = 13.6, 5.2 Hz, 1H), 2.73-2.62 (m, 1H), 2.52 (dd, J = 13.6, 8.0 Hz, 1H), 2.47-2.39 (m, 1H), 2.37-2.28 (m, 1H), 2.13-1.99 (m, 2H), 1.89-1.80 (m, 1H), 1.71-1.56 (m, 2H), 1.50-1.37 (m, 1H); ^{13}C NMR (100 MHz, $CDCl_3$) δ 211.9, 143.0, 139.5, 131.0, 128.5, 127.9, 100.9, 50.8, 42.3, 40.0, 33.8, 28.2, 25.3. FT-IR: ν (cm^{-1}) 3057, 2930, 2858, 1705, 1585, 1561, 1463, 1444, 1310, 1127, 1009. HRMS [ESI] calcd for $C_{13}H_{15}INaO$ $[M+Na]^+$ 337.0060, found 337.0074.

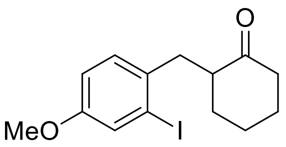

1r: colorless oil. ^{1}H NMR (400 MHz, $CDCl_3$) δ 7.33 (d, J = 2.8 Hz, 1H), 7.14-7.08 (m, 1H), 6.83-6.77 (m, 1H), 3.74 (s, 3H), 3.26 (dd, J = 14.0, 5.2 Hz, 1H), 2.67-2.56 (m, 1H), 2.50-2.26 (m, 3H), 2.12-1.97 (m, 2H), 1.88-1.78 (m, 1H), 1.72-1.52 (m, 2H), 1.48-1.33 (m, 1H); ^{13}C

NMR (100 MHz, $CDCl_3$) δ 212.2, 158.1, 135.0, 131.0, 124.4, 114.1, 100.6, 55.5, 51.1, 42.3, 38.9, 33.7, 28.2, 25.3. FT-IR: ν (cm^{-1}) 3000, 2932, 2858, 2835, 1705, 1596, 1561, 1485, 1438, 1284, 1233, 1126. HRMS [ESI] calcd for $C_{14}H_{18}IO_2$ $[M+H]^+$ 345.0346, found 345.0358.

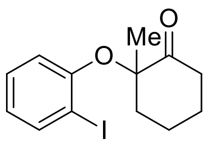

1s: colorless oil. ^1H NMR (400 MHz, $CDCl_3$) δ 7.80-7.74 (m, 1H), 7.18-7.11 (m, 1H), 6.73-6.66 (m, 1H), 6.63-6.58 (m, 1H), 2.80-2.68 (m, 1H), 2.61-2.52 (m, 1H), 2.43-2.27 (m, 2H), 2.16-2.08 (m, 1H), 1.78-1.65 (m, 3H), 1.43-1.39 (m, 3H); ^{13}C NMR (100 MHz, $CDCl_3$) δ 212.8, 154.0, 139.3, 128.8, 123.0, 115.2, 89.1, 84.6, 43.0, 38.8, 28.6, 20.5, 18.9. FT-IR: ν (cm^{-1}) 3060, 2938, 2863, 1717, 1580, 1570, 1466, 1438, 1376, 1347, 1307, 1275, 1154. HRMS [ESI] calcd for $C_{13}H_{15}INaO_2$ $[M+Na]^+$ 353.0009, found 353.0017.

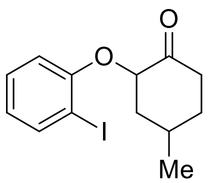

1t: white solid, m.p. 104-106 ^oC. ^1H NMR (400 MHz, $CDCl_3$) δ 7.76 (dd, J = 8.0, 1.6 Hz, 1H), 7.23-7.15 (m, 1H), 6.73-6.66 (m, 1H), 6.63-6.57 (m, 1H), 4.71 (dd, J = 12.4, 6.0 Hz, 1H), 2.55-2.41 (m, 3H), 2.14-2.00 (m, 2H), 1.78 (q, J = 12.4 Hz, 1H), 1.53-1.38 (m, 1H), 1.08 (d, J = 6.4 Hz, 3H); ^{13}C NMR (100 MHz, $CDCl_3$) δ 206.5, 156.8, 139.5, 129.2, 123.4, 114.5, 87.6, 81.5, 42.3, 40.0, 35.3, 31.1, 21.0. FT-IR: ν (cm^{-1}) 2957, 2943, 1715, 1582, 1473, 1458, 1439, 1282, 1249, 1235, 1111, 1051, 1019. HRMS [ESI] calcd for $C_{13}H_{15}INaO_2$ $[M+Na]^+$ 353.0009, found 353.0021.

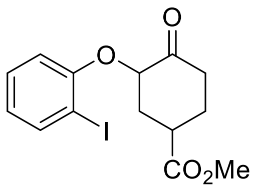

1u: white solid, m.p. 108-109 ^oC. ^1H NMR (400 MHz, $CDCl_3$) δ 7.79-7.65 (m, 1H), 7.24-7.12 (m, 1H), 6.75-6.65 (m, 1H), 6.66-6.55 (m, 1H), 4.73 (dd, J = 12.0, 6.0 Hz, 1H), 3.73-3.65 (m, 3H), 2.98-2.85 (m, 1H), 2.80-2.70 (m, 1H), 2.66-2.54 (m, 1H), 2.54-2.42 (m, 1H), 2.38-2.16 (m, 2H), 2.00-1.83 (m, 1H); ^{13}C NMR (100 MHz, $CDCl_3$) δ 204.9, 173.2, 156.6, 139.6, 129.3, 123.6, 114.6, 87.4, 80.6, 52.2, 40.6, 39.0, 36.0, 29.3. FT-IR: ν (cm^{-1}) 2959, 2869, 1731, 1720, 1583, 1472, 1433, 1270, 1247, 1226, 1172, 1044. HRMS [ESI] calcd for $C_{14}H_{15}INaO_4$ $[M+Na]^+$ 396.9907, found 396.9916.

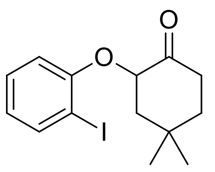

1v: white solid, m.p. 74-76 ^oC. ^1H NMR (400 MHz, $CDCl_3$) δ 7.84-7.80 (m, 1H), 7.33-7.23 (m, 1H), 6.80-6.74 (m, 1H), 6.80-6.73 (m, 1H), 4.81 (dd, J = 12.0, 6.4 Hz, 1H), 2.69-2.49 (m, 2H), 2.35-2.23 (m, 1H), 2.12-2.02 (m, 1H), 1.90-1.77 (m, 2H), 1.32 (s, 3H), 1.21 (s, 3H); ^{13}C NMR (100 MHz, $CDCl_3$) δ 205.8, 156.8, 143.1, 139.6, 129.3, 128.8, 127.0, 126.7, 123.6, 114.8, 87.7, 81.8, 42.1, 41.2, 40.3, 34.6. FT-IR: ν (cm^{-1}) 2951, 2924, 2859, 1721, 1581, 1471, 1438, 1277, 1244, 1166, 1114, 1053, 1016. HRMS [ESI] calcd for $C_{14}H_{17}INaO_2$ $[M+Na]^+$ 367.0165, found 367.0186.

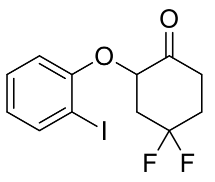

1w: white solid, m.p. 89-90 ^oC. ^1H NMR (400 MHz, $CDCl_3$) δ 7.78 (dd, J = 8.0, 1.6 Hz, 1H), 7.27-7.20 (m, 1H), 6.79-6.73 (m, 1H), 6.69 (dd, J = 8.4, 1.2 Hz, 1H), 4.87 (dd, J = 11.6, 6.4 Hz, 1H), 3.06-2.91 (m, 1H), 2.74-2.43 (m, 4H), 2.39-2.20 (m, 1H); ^{13}C NMR (100 MHz, $CDCl_3$) δ 203.1 (d, J_{C-F} = 1.2 Hz), 156.4, 139.8, 129.4, 124.1, 121.2 (dd, J_{C-F} = 245.0, 236.9 Hz), 114.6, 87.4, 77.4 (dd, J_{C-F} = 11.5, 1.3 Hz), 40.3 (dd, J_{C-F} = 25.7, 25.7 Hz), 34.2 (dd, J_{C-F} = 9.7, 1.7 Hz),

33.3 (dd, $J_{C\text{-}F}$ = 25.7, 25.7 Hz); ^{19}F NMR (376 MHz, $CDCl_3$) δ -94.1 (d, *J* = 248.9 Hz), -99.1 (d, *J* = 248.9 Hz) . FT-IR: ν (cm^{-1}) 3059, 2957, 1734, 1582, 1500, 1439, 1377, 1328, 1283, 1257, 1219, 1124, 1016. HRMS [ESI] calcd for $C_{12}H_{11}F_2INaO_2$ [M+Na]$^+$ 374.9664, found 374.9663.

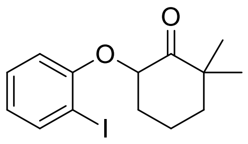

1x: white solid, m.p. 140-141 °C. ^{1}H NMR (400 MHz, $CDCl_3$) δ 7.76 (dd, *J* = 7.6, 1.2 Hz, 1H), 7.23-7.17 (m, 1H), 6.72-6.66 (m, 1H), 6.57 (dd, *J* = 8.0, 1.2 Hz, 1H), 4.93 (dd, *J* = 11.2, 6.0 Hz, 1H), 2.50-2.37 (m, 1H), 2.10-1.98 (m, 1H), 1.97-1.87 (m, 2H), 1.84-1.74 (m, 1H), 1.72-1.62 (m, 1H), 1.27 (s, 3H), 1.12 (s, 3H); ^{13}C NMR (100 MHz, $CDCl_3$) δ 209.7, 156.4, 139.1, 128.8, 122.6, 113.2, 86.7, 79.2, 45.8, 40.7, 34.3, 24.6, 24.2, 19.1. FT-IR: ν (cm^{-1}) 2979, 2966, 2925, 28405, 1713, 1580, 1469, 1459, 1440, 1277, 1251, 1016. HRMS [ESI] calcd for $C_{14}H_{17}INaO_2$ [M+Na]$^+$ 367.0165, found 367.0181.

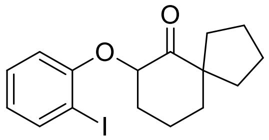

1y: white solid, m.p. 72-73 °C. ^{1}H NMR (400 MHz, $CDCl_3$) δ 7.78-7.73 (m, 1H), 7.24-7.16 (m, 1H), 6.72-6.66 (m, 1H), 6.60-6.55 (m, 1H), 4.85 (dd, *J* = 11.2, 6.0 Hz, 1H), 2.50-2.28 (m, 2H), 2.11-1.56 (m, 11H), 1.31-1.21 (m, 1H); ^{13}C NMR (100 MHz, $CDCl_3$) δ 208.2, 156.4, 139.1, 128.8, 122.6, 113.1, 86.8, 80.0, 57.0, 39.1, 35.3, 33.8, 33.3, 24.9, 24.1, 20.1. FT-IR: ν (cm^{-1}) 2936, 2866, 1710, 1580, 1568, 1468, 1449, 1439, 1245, 1212, 1046. HRMS [ESI] calcd for $C_{16}H_{19}INaO_2$ [M+Na]$^+$ 393.0322, found 393.0330.

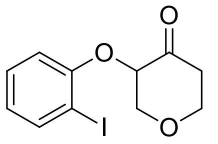

1z: white solid, m.p. 151-152 °C. ^{1}H NMR (400 MHz, $CDCl_3$) δ 7.76 (dd, *J* = 7.6, 1.6 Hz, 1H), 7.28-7.19 (m, 1H), 6.79-6.71 (m, 2H), 4.71 (dd, *J* = 8.4, 6.0 Hz, 1H), 4.44-4.36 (m, 1H), 4.23-4.13 (m, 1H), 3.90 (dd, *J* = 11.2, 8.8 Hz, 1H), 3.87-3.80 (m, 1H), 2.74-2.67 (m, 2H); ^{13}C NMR (100 MHz, $CDCl_3$) δ 202.4, 155.9, 139.2, 129.0, 123.5, 114.2, 86.8, 79.8, 71.1, 68.1, 41.8. FT-IR: ν (cm^{-1}) 2980, 2864, 1718, 1572, 1469, 1438, 1317, 1280, 1248, 1202, 1092, 1018. HRMS [ESI] calcd for $C_{11}H_{11}INaO_3$ [M+Na]$^+$ 340.9645, found 340.9647.

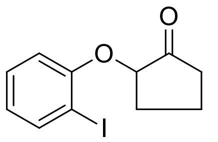

1aa: yellow oil. ^{1}H NMR (400 MHz, $CDCl_3$) δ 7.77-7.72 (m, 1H), 7.30-7.23 (m, 1H), 7.02-6.96 (m, 1H), 6.78-6.68 (m, 1H), 4.61-4.55 (m, 1H), 2.48-2.33 (m, 3H), 2.24-2.08 (m, 2H), 1.98-1.83 (m, 1H); ^{13}C NMR (100 MHz, $CDCl_3$) δ 213.4, 156.9, 139.4, 129.5, 123.7, 115.0, 87.5, 80.9, 35.3, 29.8, 17.6. FT-IR: ν (cm^{-1}) 3060, 2970, 2882, 1749, 1579, 1467, 1438, 1400, 1272, 1242, 1165, 1138, 1046, 1016. HRMS [ESI] calcd for $C_{11}H_{11}INaO_2$ [M+Na]$^+$ 324.9696, found 324.9711.

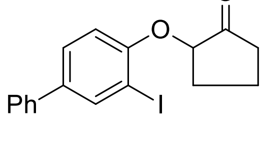

1ab: yellow solid, m.p. 88-89 °C. ^{1}H NMR (400 MHz, $CDCl_3$) δ 8.02-7.96 (m, 1H), 7.54-7.46 (m, 3H), 7.44-7.37 (m, 2H), 7.36-7.29 (m, 1H), 7.08-7.03 (m, 1H), 4.63 (t, *J* = 8.4 Hz, 1H), 2.52-2.29 (m, 3H), 2.26-2.09 (m, 2H), 2.00-1.84 (m, 1H); ^{13}C NMR (100 MHz, $CDCl_3$) δ 213.4, 156.3, 139.2, 138.0, 136.9, 128.8, 128.1, 127.3, 126.8, 114.9, 87.9, 81.0, 35.3, 29.8, 17.6. FT-IR: ν (cm^{-1})3057, 3028, 2969, 2882, 1750, 1593, 1472, 1400, 1269, 1245, 1094, 1036. HRMS [ESI] calcd for $C_{17}H_{15}INaO_2$ [M+Na]$^+$ 401.0009, found 401.0008.

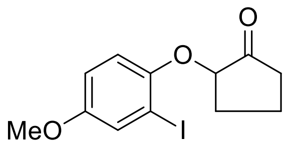

1ac: white solid, m.p. 62-64 ºC. ^{1}H NMR (400 MHz, $CDCl_3$) δ 7.28 (d, *J* = 2.8 Hz, 1H), 6.99 - 6.94 (m, 1H), 6.84-6.80 (m, 1H), 4.47-4.41 (m, 1H), 3.74 (s, 3H), 2.44-2.32 (m, 3H), 2.23-2.06 (m, 2H), 1.93-1.82 (m, 1H); ^{13}C NMR (100 MHz, $CDCl_3$) δ 213.3, 154.8, 151.0, 123.7, 116.4, 114.5, 87.8, 81.7, 55.4, 34.8, 29.4, 17.1. FT-IR: ν (cm^{-1}) 3108, 3042, 2935, 2860, 2833, 1750, 1717, 1488, 1448, 1286, 1222, 1184, 1099. HRMS [ESI] calcd for $C_{12}H_{13}INaO_3$ $[M+Na]^+$ 354.9802, found 354.9800.

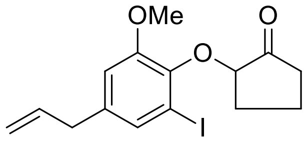

1ad: colorless oil. ^{1}H NMR (400 MHz, $CDCl_3$) δ 7.18-7.15 (m, 1H), 6.67 (d, *J* = 2.0 Hz, 1H), 5.97-5.83 (m, 1H), 5.12-5.03 (m, 2H), 4.74-4.63 (m, 1H), 3.79 (s, 3H), 3.30-3.26 (m, 2H), 2.50-2.36 (m, 1H), 2.32-2.11 (m, 4H), 1.91-1.74 (m, 1H); ^{13}C NMR (100 MHz, $CDCl_3$) δ 213.3, 151.8, 144.4, 138.1, 136.6, 130.6, 116.5, 113.3, 92.9, 81.8, 55.9, 39.4, 35.6, 29.9, 18.0. FT-IR: ν (cm^{-1}) 3075, 3000, 2970, 1745, 1589, 1561, 1461, 1406, 1266, 1226, 1142, 1039. HRMS [ESI] calcd for $C_{15}H_{17}INaO_3$ $[M+Na]^+$ 395.0115, found 395.0121.

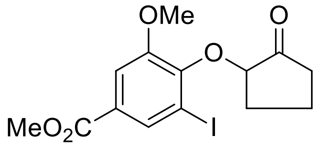

1ae: white solid, m.p. 82-84 ºC. ^{1}H NMR (400 MHz, $CDCl_3$) δ 8.06-8.03 (m, 1H), 7.55-7.52 (m, 1H), 4.88 (t, *J* = 7.6 Hz, 1H), 3.89-3.87 (m, 3H), 3.87-3.85 (m, 3H), 2.48-2.36 (m, 1H), 2.32-2.14 (m, 4H), 1.95-1.79 (m, 1H); ^{13}C NMR (100 MHz, $CDCl_3$) δ 212.8, 165.4, 151.4, 150.0, 132.6, 127.4, 113.7, 92.3, 81.8, 56.1, 52.4, 35.5, 29.9, 17.9. FT-IR: ν (cm^{-1}) 3079, 3014, 2948, 2922, 2853, 1743, 1717, 1586, 1559, 1464, 1403, 1284, 1035. HRMS [ESI] calcd for $C_{14}H_{15}INaO_5$ $[M+Na]^+$ 412.9856, found 412.9864.

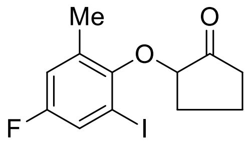

1af: colorless oil. ^{1}H NMR (400 MHz, $CDCl_3$) δ 7.31 (dd, *J* = 7.6, 3.2 Hz, 1H), 6.89-6.83 (m, 1H), 4.49-4.43 (m, 1H), 2.38-2.31 (m, 2H), 2.30-2.23 (m, 4H), 2.20-2.09 (m, 2H), 1.88-1.74 (m, 1H); ^{13}C NMR (100 MHz, $CDCl_3$) δ 212.9, 158.4 (d, J_{C-F} = 245.9 Hz), 152.2 (d, J_{C-F} = 3.0 Hz), 133.5 (d, J_{C-F} = 8.2 Hz), 123.7 (d, J_{C-F} = 24.7 Hz), 118.1 (d, J_{C-F} = 22.1 Hz), 90.9 (d, J_{C-F} = 9.2 Hz), 83.3, 35.4, 30.1, 17.6 (d, J_{C-F} = 1.2 Hz), 17.5; ^{19}F NMR (376 MHz, $CDCl_3$) δ -117.9 (s). FT-IR: ν (cm^{-1}) 3074, 2968, 2883, 1754, 1597, 1574, 1462, 1430, 1403, 1377, 1193. HRMS [ESI] calcd for $C_{12}H_{12}FINaO_2$ $[M+Na]^+$ 356.9758, found 356.9770.

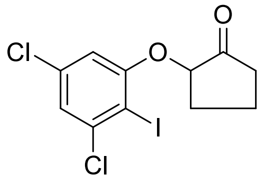

1ag: white solid, m.p. 72-73 ºC. ^{1}H NMR (400 MHz, $CDCl_3$) δ 7.15 (d, *J* = 2.0 Hz, 1H), 6.88-6.86 (m, 1H), 4.63-4.56 (m, 1H), 2.52-2.42 (m, 1H), 2.42-2.36 (m, 2H), 2.25-2.12 (m, 2H), 1.99-1.88 (m, 1H); ^{13}C NMR (100 MHz, $CDCl_3$) δ 212.5, 158.9, 140.2, 135.3, 122.6, 112.8, 90.4, 81.3, 35.2, 29.6, 17.5. FT-IR: ν (cm^{-1}) 3119, 3080, 2973, 2927, 1748, 1559, 1464, 1429, 1395, 1259, 1180, 1092. HRMS [ESI] calcd for $C_{11}H_9Cl_2INaO_2$ $[M+Na]^+$ 392.8916, found 392.8927.

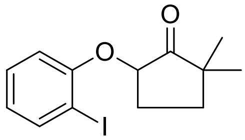

1ah: colorless oil. ^{1}H NMR (400 MHz, $CDCl_3$) δ 7.75 (dd, *J* = 7.6, 1.6 Hz, 1H), 7.30-7.22 (m, 1H), 7.04-7.00 (m, 1H), 6.77-6.71 (m, 1H), 4.66 (t, *J* = 8.0 Hz, 1H), 2.43-2.34 (m, 1H), 2.19-1.97 (m, 2H), 1.81-1.70 (m, 1H),

1.17 (s, 3H), 1.14 (s, 3H); ^{13}C NMR (100 MHz, $CDCl_3$) δ 217.5, 157.0, 139.4, 129.4, 123.7, 115.2, 87.6, 80.9, 43.3, 33.5, 26.6, 25.1, 25.0. FT-IR: ν (cm^{-1}) 3060, 2962, 2869, 1746, 1579, 1468, 1439, 1272, 1242, 1054, 1017. HRMS [ESI] calcd for $C_{13}H_{15}INaO_2$ [M+Na]$^+$ 353.0009, found 353.0028.

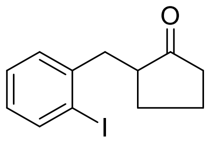

1ai: colorless oil. ^{1}H NMR (400 MHz, $CDCl_3$) δ 7.87-7.80 (m, 1H), 7.31-7.25 (m, 1H), 7.24-7.18 (m, 1H), 6.95-6.87 (m, 1H), 3.32 (dd, *J* = 14.0, 4.4 Hz, 1H), 2.65 (dd, *J* = 13.6, 9.6 Hz, 1H), 2.55-2.44 (m, 1H), 2.44-2.33 (m, 1H), 2.23-2.11 (m, 1H), 2.11-1.97 (m, 2H), 1.84-1.71 (m, 1H), 1.67-1.55 (m, 1H); ^{13}C NMR (100 MHz, $CDCl_3$) δ 219.1, 142.3, 139.2, 129.6, 127.8, 127.6, 100.2, 49.3, 39.6, 37.5, 28.8, 20.1. FT-IR: ν (cm^{-1}) 2961, 2874, 2362, 1737, 1467, 1435, 1404, 1152, 1101. HRMS [ESI] calcd for $C_{12}H_{15}IO$ [M+H]$^+$ 301.0084, found 301.0087.

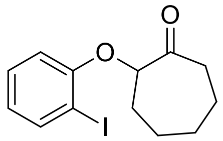

1aj: white solid, m.p. 63-64 °C. ^{1}H NMR (400 MHz, $CDCl_3$) δ 7.79 (dd, *J* = 7.6, 1.6 Hz, 1H), 7.26-7.20 (m, 1H), 6.75-6.69 (m, 1H), 6.62 (dd, *J* = 8.4, 1.2 Hz, 1H), 4.79 (dd, *J* = 6.8, 2.4 Hz, 1H), 2.62-2.54 (m, 1H), 2.45-2.36 (m, 1H), 2.26-2.00 (m, 3H), 1.91-1.71 (m, 4H), 1.44-1.34 (m, 1H); ^{13}C NMR (100 MHz, $CDCl_3$) δ 212.0, 156.2, 139.9, 129.6, 123.2, 112.3, 86.5, 84.1, 40.7, 31.0, 27.6, 25.1, 22.4. FT-IR: ν (cm^{-1}) 2951, 2862, 2360, 2341, 1717, 1685, 1584, 1471, 1443, 1364, 1311, 1274, 1244. HRMS [ESI] calcd for $C_{13}H_{16}IO_2$ [M+H]$^+$ 331.0189, found 331.0179.

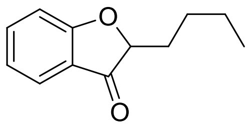

2a: colorless oil. ^{1}H NMR (400 MHz, $CDCl_3$) δ 7.67-7.63 (m, 1H), 7.63-7.57 (m, 1H), 7.13-7.09 (m, 1H), 7.09-7.04 (m, 1H), 4.55 (dd, *J* = 8.0, 4.0 Hz, 1H), 2.06-1.95 (m, 1H), 1.81-1.71 (m, 1H), 1.52-1.43 (m, 2H), 1.42-1.33 (m, 2H), 0.91 (t, *J* = 7.2 Hz, 3H); ^{13}C NMR (100 MHz, $CDCl_3$) δ 201.8, 172.3, 137.4, 123.8, 121.2, 120.6, 113.0, 85.2, 30.6, 26.4, 21.9, 13.3. FT-IR: ν (cm^{-1}) 2961, 2935, 2875, 1715, 1612, 1476, 1462, 1318, 1296, 1247, 1209, 1194. HRMS [ESI] calcd for $C_{12}H_{15}O_2$ [M+H]$^+$ 191.1067, found 191.1067.

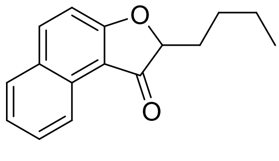

2b: yellow oil. ^{1}H NMR (400 MHz, $CDCl_3$) δ 8.78-8.73 (m, 1H), 8.04 (d, *J* = 8.8 Hz, 1H), 7.81 (d, *J* = 8.0 Hz, 1H), 7.68-7.62 (m, 1H), 7.48-7.42 (m, 1H), 7.23 (d, *J* = 9.2 Hz, 1H), 4.67 (dd, *J* = 8.0, 4.0 Hz, 1H), 2.14-2.05 (m, 1H), 1.88-1.76 (m, 1H), 1.56-1.47 (m, 2H), 1.44-1.34 (m, 2H), 0.92 (t, *J* = 7.2 Hz, 3H); ^{13}C NMR (100 MHz, $CDCl_3$) δ 201.6, 175.4, 139.8, 129.8, 129.3, 129.1, 128.5, 125.3, 123.1, 114.0, 113.0, 86.6, 31.2, 26.9, 22.5, 13.9. FT-IR: ν (cm^{-1}) 2957, 2930, 2871, 1698, 1629, 1578, 1530, 1461, 1209, 1155, 1132, 1095. HRMS [ESI] calcd for $C_{16}H_{17}O_2$ [M+H]$^+$ 241.1223, found 241.1228.

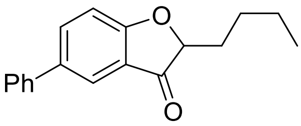

2c: white solid, m.p. 55-56 °C. ^{1}H NMR (400 MHz, $CDCl_3$) δ 7.88-7.84 (m, 2H), 7.57-7.52 (m, 2H), 7.47-7.41 (m, 2H), 7.38-7.32 (m, 1H), 7.21-7.15 (m, 1H), 4.63 (dd, *J* = 8.0, 4.4 Hz, 1H), 2.09-1.99 (m, 1H), 1.85-1.74 (m, 1H), 1.56-1.46 (m, 2H), 1.43-1.34 (m, 2H), 0.93 (t, *J* = 7.2 Hz, 3H); ^{13}C NMR (100 MHz, $CDCl_3$) δ 202.2, 172.2, 139.6, 137.2, 135.4, 129.0, 127.4, 126.9, 122.2, 121.5, 113.7, 86.3, 31.1, 26.9, 22.5, 13.9. FT-IR: ν (cm^{-1}) 2954, 2921, 2871,

2856, 1706, 1623, 1508, 1474, 1324, 1264, 1209, 1172. HRMS [ESI] calcd for $C_{18}H_{18}NaO_2$ $[M+Na]^+$ 289.1199, found 289.1209.

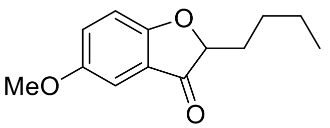

2d: colorless oil. ^{1}H NMR (400 MHz, $CDCl_3$) δ 7.25-7.21 (m, 1H), 7.05-7.01 (m, 2H), 4.56 (dd, J = 8.0, 4.0 Hz, 1H), 3.79 (s, 3H), 2.04-1.94 (m, 1H), 1.80-1.68 (m, 1H), 1.50-1.32 (m, 4H), 0.90 (t, J = 7.6 Hz, 3H); ^{13}C NMR (100 MHz, $CDCl_3$) δ 202.5, 168.1, 154.81, 128.0, 120.9, 114.3, 103.9, 86.4, 55.9, 31.2, 26.9, 22.4, 13.8. FT-IR: ν (cm^{-1}) 2956, 2872, 2837, 1706, 1487, 1438, 1338, 1273, 1240, 1194, 1154. HRMS [ESI] calcd for $C_{13}H_{16}NaO_3$ $[M+Na]^+$ 243.0992, found 243.0987.

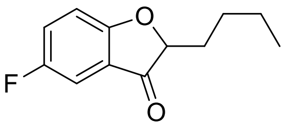

2e: yellow oil. ^{1}H NMR (400 MHz, $CDCl_3$) δ 7.36-7.30 (m, 1H), 7.30-7.25 (m, 1H), 7.10-7.05 (m, 1H), 4.59 (dd, J = 8.0, 4.0 Hz, 1H), 2.04-1.94 (m, 1H), 1.80-1.69 (m, 1H), 1.50-1.30 (m, 4H), 0.93-0.87 (m, 3H); ^{13}C NMR (100 MHz, $CDCl_3$) δ 201.7 (d, J_{C-F} = 3.0 Hz), 168.9, 157.6 (d, J_{C-F} = 241.4 Hz), 125.6 (d, J_{C-F} = 25.8 Hz), 121.5 (d, J_{C-F} = 7.7 Hz), 114.5 (d, J_{C-F} = 7.8 Hz), 109.2 (d, J_{C-F} = 23.4 Hz), 86.8 , 31.1, 26.8, 22.4, 13.8; ^{19}F NMR (376 MHz, $CDCl_3$) δ -121.3 (s). FT-IR: ν (cm^{-1}) 2958, 2933, 2873, 1716, 1622, 1482, 1445, 1316, 1261, 1188, 1149. HRMS [ESI] calcd for $C_{12}H_{14}FO_2$ $[M+H]^+$ 209.0972, found 209.0979.

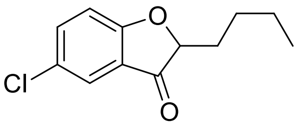

2f: colorless oil. ^{1}H NMR (400 MHz, $CDCl_3$) δ 7.60-7.58 (m, 1H), 7.56-7.51 (m, 1H), 7.09-7.04 (m, 1H), 4.59 (dd, J = 8.0, 4.0 Hz, 1H), 2.04-1.95 (m, 1H), 1.80-1.70 (m, 1H), 1.49-1.41 (m, 2H), 1.39-1.32 (m, 2H), 0.90 (t, J = 7.2 Hz, 3H); ^{13}C NMR (100 MHz, $CDCl_3$) δ 201.0, 171.0, 137.8, 127.3, 123.6, 122.2, 114.8, 86.6, 31.0, 26.8, 22.4, 13.8. FT-IR: ν (cm^{-1}) 2959, 2932, 2873, 1755, 1708, 1608, 1466, 1300, 1267, 1249, 1211, 1133, 1083. HRMS [ESI] calcd for $C_{12}H_{13}ClNaO_2$ $[M+Na]^+$ 247.0496, found 247.0495.

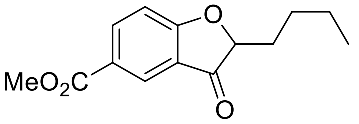

2g: colorless oil. ^{1}H NMR (400 MHz, $CDCl_3$) δ 8.35-8.32 (m, 1H), 8.31-8.26 (m, 1H), 7.16-7.11 (m, 1H), 4.64 (dd, J = 8.0, 4.4 Hz, 1H), 3.90 (s, 3H), 2.07-1.95 (m, 1H), 1.83-1.71 (m, 1H), 1.51-1.41 (m, 2H), 1.40-1.32 (m, 2H), 0.89 (t, J = 7.2 Hz, 3H); ^{13}C NMR (100 MHz, $CDCl_3$) δ 200.5, 174.7, 165.4, 138.6, 126.3, 123.7, 120.7, 113.0, 86.4, 51.8, 30.5, 26.2, 21.9, 13.3. FT-IR: ν (cm^{-1}) 2954, 2925, 2870, 1719, 1708, 1622, 1487, 1431, 1260, 1209, 1138. HRMS [ESI] calcd for $C_{14}H_{17}O_4$ $[M+H]^+$ 249.1121, found 249.1119.

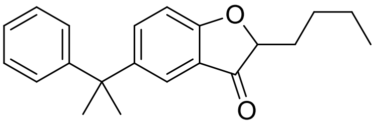

2h: colorless oil. ^{1}H NMR (400 MHz, $CDCl_3$) δ 7.65 (d, J = 2.0 Hz, 1H), 7.40-7.35 (m, 1H), 7.31-7.25 (m, 2H), 7.23-7.16 (m, 3H), 7.00-6.96 (m, 1H), 4.56 (dd, J = 8.0, 4.0 Hz, 1H), 2.08-1.95 (m, 1H), 1.80-1.72 (m, 1H), 1.69 (s, 6H), 1.55-1.45 (m, 2H), 1.43-1.34 (m, 2H), 0.92 (t, J = 7.2 Hz, 3H); ^{13}C NMR (100 MHz, $CDCl_3$) δ 202.5, 171.3, 149.8, 144.7, 138.2, 128.2, 126.6, 125.9, 120.8, 120.4, 113.1, 86.2, 42.7, 31.1, 30.8, 30.7, 27.0, 22.4, 13.9. FT-IR: ν (cm^{-1}) 2961, 2931, 2871, 1709, 1619, 1597, 1486, 1466, 1323, 1281, 1237. HRMS [ESI] calcd for $C_{21}H_{25}O_2$ $[M+H]^+$ 309.1849, found 309.1852.

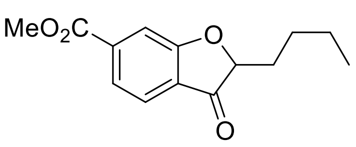

2i: colorless oil. ^{1}H NMR (400 MHz, $CDCl_3$) δ 7.76 (s, 1H), 7.73-7.67 (m, 2H), 4.62 (dd, *J* = 8.0, 4.0 Hz, 1H), 3.95 (s, 3H), 2.06-1.95 (m, 1H), 1.82-1.73 (m, 1H), 1.48-1.33 (m, 4H), 0.90 (t, *J* = 7.2 Hz, 3H); ^{13}C NMR (100 MHz, $CDCl_3$) δ 202.0, 172.3, 165.9, 138.6, 124.2, 124.2, 122.6, 114.9, 86.4, 52.8, 31.1, 26.8, 22.4, 13.8. FT-IR: ν (cm^{-1}) 2958, 2932, 2874, 1763, 1726, 1700, 1622, 1436, 1382, 1281, 1075. HRMS [ESI] calcd for $C_{14}H_{17}O_4$ $[M+H]^+$ 249.1121, found 249.1128.

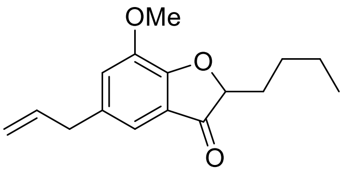

2j: colorless oil. ^{1}H NMR (400 MHz, $CDCl_3$) δ 7.06-7.03 (m, 1H), 6.94-6.92 (m, 1H), 6.01-5.84 (m, 1H), 5.14-5.10 (m, 2H), 4.60 (dd, *J* = 7.6, 4.4 Hz, 1H), 3.94 (s, 3H), 3.37 (d, *J* = 6.8 Hz, 2H), 2.07-1.96 (m, 1H), 1.85-1.73 (m, 1H), 1.52-1.41 (m, 2H), 1.40-1.30 (m, 2H), 0.89 (t, *J* = 7.2 Hz, 3H); ^{13}C NMR (100 MHz, $CDCl_3$) δ 202.1, 161.8, 146.4, 136.8, 134.6, 122.2, 119.2, 116.5, 114.4, 86.5, 56.2, 39.8, 31.1, 26.6, 22.5, 13.8. FT-IR: ν (cm^{-1}) 2958, 2933, 2871, 1709, 1636, 1616, 1506, 1457, 1274, 1184, 1147. HRMS [ESI] calcd for $C_{16}H_{21}O_3$ $[M+H]^+$ 261.1485, found 261.1492.

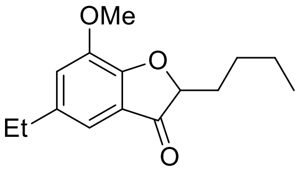

2k: yellow oil. ^{1}H NMR (400 MHz, $CDCl_3$) δ 7.11-7.08 (m, 1H), 7.01-6.98 (m, 1H), 4.64 (dd, *J* = 7.6, 4.4 Hz, 1H), 3.99 (s, 3H), 2.69 (q, *J* = 7.6 Hz, 2H), 2.12-2.01 (m, 1H), 1.90-1.77 (m, 1H), 1.56-1.47 (m, 2H), 1.45-1.36 (m, 2H), 1.28 (t, *J* = 7.6 Hz, 3H), 0.93 (t, *J* = 7.2 Hz, 3H); ^{13}C NMR (100 MHz, $CDCl_3$) δ 201.9, 161.0, 145.7, 138.3, 121.6, 118.4, 112.7, 85.9, 55.6, 30.6, 28.0, 26.1, 22.0, 15.0, 13.3. FT-IR: ν (cm^{-1}) 2960, 2933, 2871, 1710, 1618, 1506, 1462, 1284, 1149, 1107, 1059. HRMS [ESI] calcd for $C_{15}H_{20}NaO_3$ $[M+Na]^+$ 271.1305, found 271.1300.

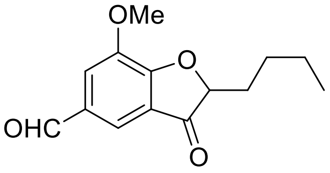

2l: white solid, m.p. 75-76 °C. ^{1}H NMR (400 MHz, $CDCl_3$) δ 9.90 (s, 1H), 7.74 (s, 1H), 7.65 (s, 1H), 4.76 (dd, *J* = 7.6, 4.4 Hz, 1H), 4.02 (s, 3H), 2.11-2.01 (m, 1H), 1.91-1.80 (m, 1H), 1.54-1.45 (m, 2H), 1.41-1.31 (m, 2H), 0.90 (t, *J* = 7.6 Hz, 3H); ^{13}C NMR (100 MHz, $CDCl_3$) δ 200.6, 190.1, 167.2, 147.6, 131.9, 122.3, 121.5, 114.7, 87.9, 56.4, 31.0, 26.5, 22.4, 13.8. FT-IR: ν (cm^{-1}) 2957, 2853, 1712, 1692, 1612, 1599, 1504, 1328, 1299, 1189, 1147. HRMS [ESI] calcd for $C_{14}H_{17}O_4$ $[M+H]^+$ 249.1121, found 249.1121.

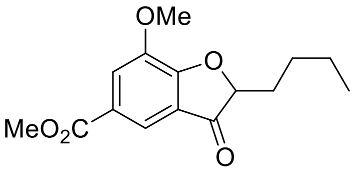

2m: white solid, m.p. 99-100 °C. ^{1}H NMR (400 MHz, $CDCl_3$) δ 7.95 (d, *J* = 1.6 Hz, 1H), 7.77-7.74 (m, 1H), 4.69 (dd, *J* = 7.6, 4.4 Hz, 1H), 3.99 (s, 3H), 3.90 (s, 3H), 2.09-1.97 (m, 1H), 1.88-1.75 (m, 1H), 1.52-1.41 (m, 2H), 1.41-1.31 (m, 2H), 0.88 (t, *J* = 7.2 Hz, 3H); ^{13}C NMR (100 MHz, $CDCl_3$) δ 200.9, 166.0, 165.7, 146.5, 124.8, 122.1, 118.4, 118.1, 87.5, 56.4, 52.4, 31.0, 26.5, 22.4, 13.8. FT-IR: ν (cm^{-1}) 2956, 2922, 2852, 1705, 1610, 1503, 1463, 1445, 1430, 1331, 1224, 1175. HRMS [ESI] calcd for $C_{15}H_{18}NaO_5$ $[M+Na]^+$ 301.1046, found 301.1054.

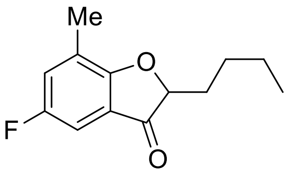

2n: colorless oil. ^{1}H NMR (400 MHz, $CDCl_3$) δ 7.19-7.12 (m, 1H), 7.13-7.07 (m, 1H), 4.58 (dd, *J* = 8.0, 4.4 Hz, 1H), 2.31 (s, 3H), 2.04-1.93 (m, 1H), 1.80-1.68 (m, 1H), 1.51-1.42 (m, 2H), 1.41-1.33 (m, 2H), 0.91 (t, *J* = 7.2 Hz, 3H); ^{13}C NMR (100 MHz, $CDCl_3$) δ 202.2 (d, $J_{C\text{-}F}$ = 3.1 Hz), 168.0, 157.5 (d, $J_{C\text{-}F}$ = 240.8 Hz), 125.9 (d, $J_{C\text{-}F}$ = 25.6 Hz), 125.5 (d, $J_{C\text{-}F}$ = 7.4 Hz), 120.6 (d, $J_{C\text{-}F}$ = 8.4 Hz), 106.0 (d, $J_{C\text{-}F}$ = 23.3 Hz), 86.5, 31.1, 26.8, 22.4, 14.3, 13.8; ^{19}F NMR (376 MHz, $CDCl_3$) δ -121.8 (s). FT-IR: ν (cm^{-1}) 2958, 2932, 2872, 1714, 1617, 1490, 1459, 1333, 1281, 1232, 1184. HRMS [ESI] calcd for $C_{13}H_{16}FO_2$ $[M+H]^+$ 223.1129, found 223.1136.

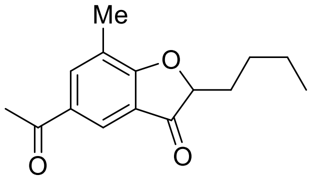

2o: white solid, m.p. 76-77 ºC. ^{1}H NMR (400 MHz, $CDCl_3$) δ 8.12-8.07 (m, 1H), 8.06-8.02 (m, 1H), 4.66 (dd, *J* = 8.0, 4.4 Hz, 1H), 2.56 (s, 3H), 2.34 (s, 3H), 2.07-1.97 (m, 1H), 1.83-1.71 (m, 1H), 1.50-1.32 (m, 4H), 0.89 (t, *J* = 7.2 Hz, 3H); ^{13}C NMR (100 MHz, $CDCl_3$) δ 201.2, 195.8, 174.0, 137.2, 130.8, 123.8, 122.7, 119.8, 86.6, 30.5, 26.2, 25.9, 21.9, 13.8, 13.3. FT-IR: ν (cm^{-1}) 2950, 2932, 2870, 1712, 1677, 1606, 1486, 1328, 1296, 1254, 1196, 1082. HRMS [ESI] calcd for $C_{15}H_{18}NaO_3$ $[M+Na]^+$ 269.1148, found 269.1139.

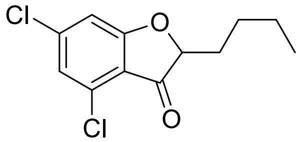

2p: colorless oil. ^{1}H NMR (400 MHz, $CDCl_3$) δ 7.06-7.00 (m, 2H), 4.61 (dd, *J* = 8.0, 4.4 Hz, 1H), 2.05-1.97 (m, 1H), 1.82-1.72 (m, 1H), 1.48-1.34 (m, 4H), 0.91 (t, *J* = 7.2 Hz, 3H); ^{13}C NMR (100 MHz, $CDCl_3$) δ 197.6, 173.2, 143.9, 132.5, 123.5, 117.0, 112.4, 86.9, 31.1, 26.7, 22.4, 13.8. FT-IR: ν (cm^{-1}) 2951, 2927, 2871, 1708, 1635, 1594, 1576, 1406, 1315, 1162, 1079. HRMS [ESI] calcd for $C_{12}H_{12}Cl_2NaO_2$ $[M+Na]^+$ 281.0107, found 281.0111.

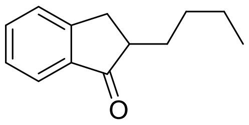

2q: colorless oil. ^{1}H NMR (400 MHz, $CDCl_3$) δ 7.78-7.72 (m, 1H), 7.61-7.54 (m, 1H), 7.48-7.43 (m, 1H), 7.39-7.33 (m, 1H), 3.32 (dd, *J* = 17.2, 8.0 Hz, 1H), 2.86-2.78 (m, 1H), 2.69-2.60 (m, 1H), 2.02-1.89 (m, 1H), 1.51-1.31 (m, 5H), 0.92 (t, *J* = 7.2 Hz, 3H); ^{13}C NMR (100 MHz, $CDCl_3$) δ 209.2, 153.8, 136.9, 134.6, 127.3, 126.6, 123.9, 47.5, 32.9, 31.2, 29.6, 22.7, 14.0. FT-IR: ν (cm^{-1}) 2956, 2927, 2856, 1708, 1608, 1464, 1434, 1378, 1327, 1284, 1207, 1182. HRMS [ESI] calcd for $C_{13}H_{17}O$ $[M+H]^+$ 189.1274, found 189.1274.

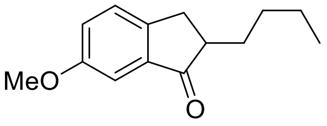

2r: colorless oil. ^{1}H NMR (400 MHz, $CDCl_3$) δ 7.37-7.31 (m, 1H), 7.21-7.15 (m, 2H), 3.83 (s, 3H), 3.24 (dd, *J* = 16.8, 7.6 Hz, 1H), 2.81-2.64 (m, 2H), 2.00-1.88 (m, 1H), 1.49-1.30 (m, 5H), 0.95-0.87 (m, 3H); ^{13}C NMR (100 MHz, $CDCl_3$) δ 209.2, 159.3, 146.7, 138.0, 127.2, 124.1, 105.0, 55.6, 48.3, 32.2, 31.3, 29.6, 22.7, 14.0. FT-IR: ν (cm^{-1}) 2955, 2928, 2856, 1704, 1616, 1490, 1464, 1434, 1275, 1231, 1028. HRMS [ESI] calcd for $C_{14}H_{19}O_2$ $[M+H]^+$ 219.1380, found 219.1381.

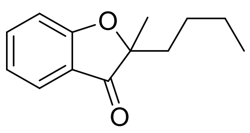

2s: colorless oil. ^{1}H NMR (400 MHz, $CDCl_3$) δ 7.67-7.63 (m, 1H), 7.63-7.58 (m, 1H), 7.10-7.03 (m, 2H), 1.86-1.78 (m, 2H), 1.42 (s, 3H), 1.34-1.25 (m, 3H), 1.18-1.06 (m, 1H), 0.86-0.80 (m, 3H); ^{13}C NMR (100 MHz, $CDCl_3$) δ 204.1, 171.0, 137.5, 124.1, 121.0, 120.1, 112.9, 90.0, 36.3, 24.7, 22.3, 21.4, 13.3. FT-IR: ν (cm^{-1}) 2957, 2932, 2871, 1715, 1611, 1477, 1462, 1372, 1323, 1298, 1267, 1189. HRMS [ESI] calcd for $C_{13}H_{17}O_2$ [M+H]$^+$ 205.1223, found 205.1233.

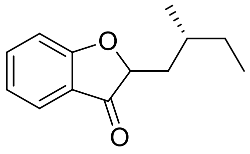

2t (*d.r.* = 1.2:1): colorless oil. ^{1}H NMR (400 MHz, $CDCl_3$) δ 7.68-7.63 (m, 1H, two isomers), 7.63-7.57 (m, 1H, two isomers), 7.13-7.09 (m, 1H, two isomers), 7.09-7.03 (m, 1H, two isomers), 4.64-4.56 (m, 1H, two isomers), 1.98-1.89 (m, 0.45H, one isomer), 1.84-1.78 (m, 0.55H, one isomer), 1.74-1.70 (m, 0.45H, one isomer),1.69-1.61(m, 0.55H, one isomer), 1.58-1.48 (m, 1H, two isomers), 1.45-1.34 (m, 0.55H, two isomers), 1.32-1.20 (m, 1.4H, two isomers), 1.04-0.97 (m, 3H, two isomers), 0.95-0.87 (m, 3H, two isomers); ^{13}C NMR (100 MHz, $CDCl_3$) δ 202.6 & 202.6 (two isomers), 172.6 (overlap), 137.9 & 137.9 (two isomers), 124.3 (overlap), 121.7 & 121.7 (two isomers), 120.9 & 120.9 (two isomers), 113.6 & 113.5 (two isomers), 84.6 & 84.3 (two isomers), 38.3 & 38.2 (two isomers), 31.5 & 31.3 (two isomers), 30.0 & 28.7 (two isomers), 19.7 & 18.4 (two isomers), 11.2 & 11.1 (two isomers). FT-IR: ν (cm^{-1}) 2961, 2929, 2875, 1715, 1613, 1476, 1462, 1380, 1319, 1297, 1143. HRMS [ESI] calcd for $C_{13}H_{17}O_2$ [M+H]$^+$ 205.1223, found 205.1224.

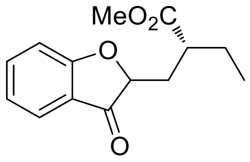

2u (*d.r.* = 1.3:1): colorless oil. ^{1}H NMR (400 MHz, $CDCl_3$) δ 7.67-7.56 (m, 2H, two isomers), 7.11-7.04 (m, 2H, two isomers), 4.60-4.54 (m, 1H, two isomers), 369-3.65 (m, 3H, two isomers), 2.75-2.65 (m, 0.43H, one isomer), 2.62-2.53 (m, 0.57H, one isomer), 2.45-2.37 (m, 0.57H, one isomer), 2.16-1.97 (m, 1H, two isomers), 1.86-1.77 (m, 0.57H, one isomer), 1.70-1.52 (m, 2H, two isomers), 0.96-0.86 (m, 3H, two isomers); ^{13}C NMR (100 MHz, $CDCl_3$) δ 201.3 & 201.3 (two isomers), 175.6 & 175.4 (two isomers), 172.6 & 172.4 (two isomers), 138.0 & 138.0 (two isomers), 124.4 & 124.3 (two isomers), 122.0 (overlap), 120.8 & 120.6 (two isomers), 113.5 & 113.5(two isomers), 83.8 & 83.6 (two isomers), 51.6 & 51.6 (two isomers), 43.6 & 43.0 (two isomers), 33.3 & 33.3 (two isomers), 26.0 & 25.2 (two isomers), 11.5 & 11.4 (two isomers). FT-IR: ν (cm^{-1}) 2964, 2924, 2878, 1715, 1612, 1475, 1462, 1435, 1317, 1210, 1194, 1167. HRMS [ESI] calcd for $C_{14}H_{17}O_4$ [M+H]$^+$ 249.1121, found 249.1128.

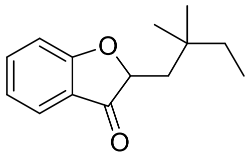

2v: colorless oil. ^{1}H NMR (400 MHz, $CDCl_3$) δ 7.68-7.63 (m, 1H), 7.63-7.56 (m, 1H), 7.14-7.01 (m, 2H), 4.58 (dd, *J* = 10.4, 1.6 Hz, 1H), 1.90 (dd, *J* = 15.2, 1.6 Hz, 1H), 1.54-1.45 (m, 1H), 1.44-1.36 (m, 2H), 1.02 (d, *J* = 2.4 Hz, 6H), 0.88 (t, *J* = 7.6 Hz, 3H); ^{13}C NMR (100 MHz, $CDCl_3$) δ 203.0, 172.7, 137.9, 124.3, 121.7, 120.6, 113.6, 84.0, 43.1, 34.8, 33.0, 27.0, 26.8, 8.4. FT-IR: ν (cm^{-1}) 2962, 2879, 1719, 1613, 1475, 1462, 1318, 1300, 1196, 1142, 1103. HRMS [ESI] calcd for $C_{14}H_{19}O_2$ [M+H]$^+$ 219.1380, found 219.1386.

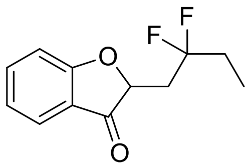

2w: colorless oil. ^{1}H NMR (400 MHz, $CDCl_3$) δ 7.70-7.59 (m, 2H), 7.20-7.07 (m, 2H), 4.80-4.75 (m, 1H), 2.66-2.51 (m, 1H), 2.22-1.95 (m, 3H), 1.07 (t, J = 7.6 Hz, 3H); ^{13}C NMR (100 MHz, $CDCl_3$) δ 200.5, 172.6, 138.4, 124.4, 123.8 (t, $J_{C\text{-}F}$ = 240.7 Hz), 122.3, 120.2, 113.7, 80.3 (dd, $J_{C\text{-}F}$ = 5.2, 3.6 Hz), 37.9 (t, $J_{C\text{-}F}$ = 26.6 Hz), 30.2 (t, $J_{C\text{-}F}$ = 25.3 Hz), 6.5 (t, $J_{C\text{-}F}$ = 5.6 Hz); ^{19}F NMR (376 MHz, $CDCl_3$) δ -98.4 (d, J = 242.1 Hz), -99.1 (d, J = 242.1 Hz). FT-IR: ν (cm^{-1}) 2985, 2947, 2892, 1716, 1613, 1476, 1463, 1317, 1301, 1294, 1193, 1143. HRMS [ESI] calcd for $C_{12}H_{13}F_2O_2$ $[M+H]^+$ 227.0878, found 227.0887.

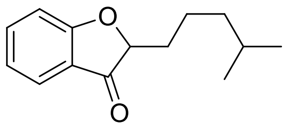

2x: colorless oil. ^{1}H NMR (400 MHz, $CDCl_3$) δ 7.67-7.63 (m, 1H), 7.63-7.57 (m, 1H), 7.14-7.08 (m, 1H), 7.09-7.04 (m, 1H), 4.55 (dd, J = 8.4, 4.4 Hz, 1H), 2.02-1.92 (m, 1H), 1.79-1.67 (m, 1H), 1.59-1.45 (m, 3H), 1.26-1.19 (m, 2H), 0.87 (d, J = 6.8 Hz, 6H); ^{13}C NMR (100 MHz, $CDCl_3$) δ 202.2, 172.7, 137.9, 124.3, 121.7, 121.1, 113.5, 85.7, 38.6, 31.6, 27.8, 22.7, 22.6, 22.5. FT-IR: ν (cm^{-1}) 2953, 2868, 1715, 1613, 1475, 1462, 1317, 1298, 1211, 1143, 1081. HRMS [ESI] calcd for $C_{14}H_{18}NaO_2$ $[M+Na]^+$ 241.1199, found 241.1210.

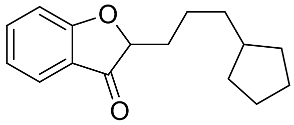

2y: colorless oil. ^{1}H NMR (400 MHz, $CDCl_3$) δ 7.67-7.63 (m, 1H), 7.63-7.57 (m, 1H), 7.12 (d, J = 8.4 Hz, 1H), 7.09-7.03 (m, 1H), 4.55 (dd, J = 8.4, 4.4 Hz, 1H), 2.04-1.95 (m, 1H), 1.80-1.70 (m, 4H), 1.63-1.55 (m, 2H), 1.53-1.44 (m, 4H), 1.39-1.32 (m, 2H), 1.10-1.00 (m, 2H); ^{13}C NMR (100 MHz, $CDCl_3$) δ 201.8, 172.3, 137.4, 123.8, 121.2, 120.6, 113.0, 85.2, 39.4, 35.3, 32.2, 32.1, 31.1, 24.6, 23.5. FT-IR: ν (cm^{-1}) 2945, 2865, 1717, 1614, 1475, 1462, 1316, 1298, 1247, 1211, 1193. HRMS [ESI] calcd for $C_{16}H_{21}O_2$ $[M+H]^+$ 245.1536, found 245.1538.

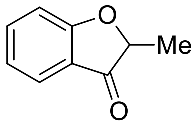

2z: colorless oil. ^{1}H NMR (400 MHz, $CDCl_3$) δ 7.69-7.65 (m, 1H), 7.64-7.58 (m, 1H), 7.13-7.05 (m, 2H), 4.64 (q, J = 7.2 Hz, 1H), 1.53 (d, J = 7.2 Hz, 3H); ^{13}C NMR (100 MHz, $CDCl_3$) δ 202.6, 172.4, 138.0, 124.5, 121.9, 120.4, 113.6, 81.8, 16.4. FT-IR: ν (cm^{-1}) 2981, 2930, 2855, 1714, 1612, 1476, 1463, 1327, 1316, 1282, 1212, 1120. HRMS [ESI] calcd for $C_9H_9O_2$ $[M+H]^+$ 149.0597, found 149.0605.

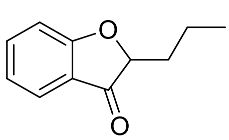

2aa: colorless oil. ^{1}H NMR (400 MHz, $CDCl_3$) δ 7.67-7.62 (m, 1H), 7.62-7.56 (m, 1H), 7.13-7.08 (m, 1H), 7.08-7.02 (m, 1H), 4.56 (dd, J = 8.0, 4.0 Hz, 1H), 2.02-1.91 (m, 1H), 1.79-1.69 (m, 1H), 1.58-1.48 (m, 2H), 0.97 (t, J = 7.2 Hz, 3H); ^{13}C NMR (100 MHz, $CDCl_3$) δ 201.8, 172.3, 137.4, 123.8, 121.2, 120.6, 113.0, 85.0, 32.9, 17.7, 13.3. FT-IR: ν (cm^{-1}) 2961, 2935, 2875, 1715, 1612, 1476, 1462, 1318, 1296, 1247, 1209, 1194. HRMS [ESI] calcd for $C_{11}H_{13}O_2$ $[M+H]^+$ 177.0910, found 177.0919.

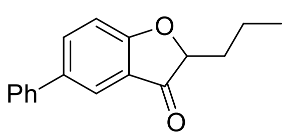

2ab: white solid, m.p. 60-61 °C. ^{1}H NMR (400 MHz, $CDCl_3$) δ 7.89-7.84 (m, 2H), 7.58-7.52 (m, 2H), 7.47-7.41 (m, 2H), 7.38-7.32 (m, 1H), 7.21-7.16 (m, 1H), 4.64 (dd, J = 8.0, 4.0 Hz, 1H), 2.06-1.95 (m, 1H), 1.85-1.73 (m, 1H), 1.61-1.50 (m, 2H), 1.00 (t, J = 7.2 Hz, 3H); ^{13}C NMR (100 MHz, $CDCl_3$) δ 201.8, 171.7, 139.2, 136.7, 134.9, 128.5, 127.0, 126.4, 121.7,

121.0, 113.2, 85.7, 32.9, 17.7, 13.3. FT-IR: ν (cm^{-1}) 2962, 2937, 2873, 1714, 1615, 1596, 1508, 1264, 1210, 1170, 1144. HRMS [ESI] calcd for $C_{17}H_{16}NaO_2$ $[M+Na]^+$ 275.1043, found 275.1041.

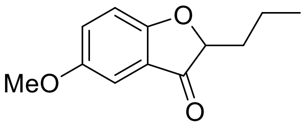

2ac: colorless oil. ^1H NMR (400 MHz, $CDCl_3$) δ 7.25-7.21 (m, 1H), 7.05-7.02 (m, 2H), 4.57 (dd, *J* = 8.0, 4.0 Hz, 1H), 3.79 (s, 3H), 2.02-1.91 (m, 1H), 1.79-1.68 (m, 1H), 1.57-1.47 (m, 2H), 0.97 (t, *J* = 7.2 Hz, 3H); ^{13}C NMR (100 MHz, $CDCl_3$) δ 202.0, 167.7, 154.3, 127.5, 120.4, 113.9, 103.4, 85.7, 55.4, 32.9, 17.7, 13.3. FT-IR: ν (cm^{-1}) 2961, 2935, 2874, 1704, 1621, 1487, 1438, 1380, 1275, 1238, 1154. HRMS [ESI] calcd for $C_{12}H_{15}O_3$ $[M+H]^+$ 207.1016, found 207.1025.

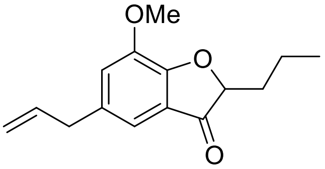

2ad: colorless oil. ^1H NMR (400 MHz, $CDCl_3$) δ 7.07-6.99 (m, 1H), 6.94-6.91 (m, 1H), 6.01-5.84 (m, 1H), 5.13-5.05 (m, 2H), 4.61 (dd, *J* = 7.6, 4.0 Hz, 1H), 3.93 (s, 3H), 3.39-3.34 (m, 2H), 2.03-1.92 (m, 1H), 1.84-1.72 (m, 1H), 1.58-1.46 (m, 2H), 0.94 (t, *J* = 7.2 Hz, 3H); ^{13}C NMR (100 MHz, $CDCl_3$) δ 202.1, 161.8, 146.4, 136.7, 134.6, 122.2, 119.2, 116.5, 114.3, 86.3, 56.2, 39.7, 33.3, 18.0, 13.8. FT-IR: ν (cm^{-1}) 3004, 2960, 2874, 1708, 1616, 1505, 1463, 1445, 1275, 1184, 1148. HRMS [ESI] calcd for $C_{15}H_{19}NaO_3$ $[M+Na]^+$ 269.1148, found 269.1157.

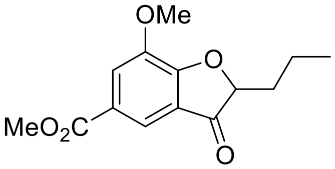

2ae: white solid, m.p. 118-119 °C. ^1H NMR (400 MHz, $CDCl_3$) δ 7.95 (d, *J* = 1.2 Hz, 1H), 7.75 (d, *J* = 1.6 Hz, 1H), 4.70 (dd, *J* = 7.6, 4.4 Hz, 1H), 3.99 (s, 3H), 3.90 (s, 3H), 2.04-1.94 (m, 1H), 1.86-1.75 (m, 1H), 1.59-1.47 (m, 2H), 0.95 (t, *J* = 7.2 Hz, 3H); ^{13}C NMR (100 MHz, $CDCl_3$) δ 200.9, 166.0, 165.6, 146.5, 124.8, 122.0, 118.4, 118.1, 87.3, 56.3, 52.4, 33.2, 17.9, 13.7. FT-IR: ν (cm^{-1}) 2965, 2874, 1716, 1607, 1504, 1464, 1433, 1337, 1318, 1299, 1234, 1220, 1176. HRMS [ESI] calcd for $C_{14}H_{16}NaO_5$ $[M+Na]^+$ 287.0890, found 287.0885.

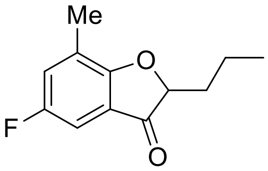

2af: colorless oil. ^1H NMR (400 MHz, $CDCl_3$) δ 7.20-7.14 (m, 1H), 7.13-7.08 (m, 1H), 4.60 (dd, *J* = 8.0, 4.4 Hz, 1H), 2.32 (s, 3H), 2.03-1.91 (m, 1H), 1.79-1.69 (m, 1H), 1.58-1.47 (m, 2H), 0.97 (t, *J* = 7.2 Hz, 3H); ^{13}C NMR (100 MHz, $CDCl_3$) δ 202.3 (d, J_{C-F} = 3.2 Hz), 167.9, 157.4 (d, J_{C-F} = 240.9 Hz), 125.8 (d, J_{C-F} = 25.6 Hz), 125.5 (d, J_{C-F} = 7.6 Hz), 120.6 (d, J_{C-F} = 8.3 Hz), 106.0 (d, J_{C-F} = 23.4 Hz), 86.4, 33.4, 18.2, 14.3 (d, J_{C-F} = 1.0 Hz), 13.8; ^{19}F NMR (376 MHz, $CDCl_3$) δ -121.8 (s). FT-IR: ν (cm^{-1}) 2961, 2934, 2875, 1713, 1616, 1490, 1460, 1413, 1382, 1280, 1234, 1184. HRMS [ESI] calcd for $C_{12}H_{14}FO_2$ $[M+H]^+$ 209.0972, found 209.0981.

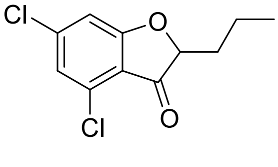

2ag: white solid, m.p. 80-81 °C. ^1H NMR (400 MHz, $CDCl_3$) δ 7.05-7.00 (m, 2H), 4.62 (dd, *J* = 8.4, 4.4 Hz, 1H), 2.03-1.91 (m, 1H), 1.80-1.68 (m, 1H), 1.57-1.46 (m, 2H), 0.97 (t, *J* = 7.6 Hz, 3H); ^{13}C NMR (100 MHz, $CDCl_3$) δ 197.2, 172.7, 143.4, 132.0, 123.0, 116.5,

112.0, 86.3, 32.9, 17.6, 13.2. FT-IR: ν (cm^{-1}) 3089, 2965, 2935, 1875, 1709, 1589, 1452, 1410, 1313, 1103, 1066. HRMS [ESI] calcd for $C_{11}H_{10}Cl_2NaO_2$ $[M+Na]^+$ 266.9950, found 266.9968.

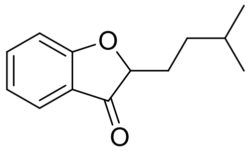

2ah: colorless oil. ^1H NMR (400 MHz, $CDCl_3$) δ 7.67-7.63 (m, 1H), 7.63-7.57 (m, 1H), 7.13-7.09 (m, 1H), 7.08-7.03 (m, 1H), 4.53 (dd, *J* = 8.0, 4.0 Hz, 1H), 2.08-1.95 (m, 1H), 1.79-1.67 (m, 1H), 1.62-1.52 (m, 1H), 1.41-1.33 (m, 2H), 0.90 (d, *J* = 2.8 Hz, 3H), 0.88 (d, *J* = 3.2 Hz, 3H); ^{13}C NMR (100 MHz, $CDCl_3$) δ 202.2, 172.8, 137.9, 124.3, 121.7, 121.1, 113.5, 85.9, 33.6, 29.4, 27.9, 22.4, 22.3. FT-IR: ν (cm^{-1}) 2955, 2871, 1715, 1613, 1475, 1463, 1386, 1315, 1192, 1143, 1102. HRMS [ESI] calcd for $C_{13}H_{17}O_2$ $[M+H]^+$ 205.1223, found 205.1228.

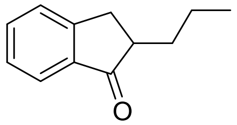

2ai: colorless oil. ^1H NMR (400 MHz, $CDCl_3$) δ 7.78-7.72 (m, 1H), 7.61-7.54 (m, 1H), 7.48-7.42 (m, 1H), 7.39-7.32 (m, 1H), 4.32 (dd, *J* = 17.2, 8.0 Hz, 1H), 2.81 (dd, *J* = 17.2, 3.6 Hz, 1H), 2.72-2.62 (m, 1H), 1.99-1.86 (m, 1H), 1.54-1.38 (m, 3H), 0.99-0.93 (m, 3H); ^{13}C NMR (100 MHz, $CDCl_3$) δ 209.1, 153.8, 136.9, 134.6, 127.3, 126.5, 123.9, 47.3, 33.6, 32.9, 20.7, 14.1. FT-IR: ν (cm^{-1}) 2958, 2931, 2872, 2360, 2342, 1711, 1610, 1464, 1328, 1296, 1272, 1211. HRMS [ESI] calcd for $C_{12}H_{14}NaO$ $[M+Na]^+$ 197.0937, found 197.0934.

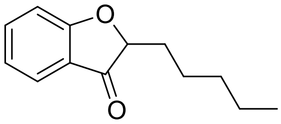

2aj: yellow oil. ^1H NMR (400 MHz, $CDCl_3$) δ 7.67-7.64 (m, 1H), 7.63-7.58 (m, 1H), 7.12 (d, *J* = 8.4 Hz, 1H), 7.09-7.04 (m, 1H), 4.56 (dd, *J* = 8.0, 4.4 Hz, 1H), 2.05-1.95 (m, 1H), 1.80-1.70 (m, 1H), 1.54-1.45 (m, 2H), 1.36-1.29 (m, 4H), 0.92-0.86 (m, 3H); ^{13}C NMR (100 MHz, $CDCl_3$) δ 202.3, 172.8, 137.9, 124.3, 121.7, 121.1, 113.5, 85.7, 31.5, 31.3, 24.5, 22.4, 14.0. FT-IR: ν (cm^{-1}) 2955, 2927, 2858, 1716, 1613, 1476, 1463, 1317, 1298, 1249, 1211, 1194, 1026. HRMS [ESI] calcd for $C_{13}H_{16}NaO_2$ $[M+Na]^+$ 227.1043, found 227.1033.

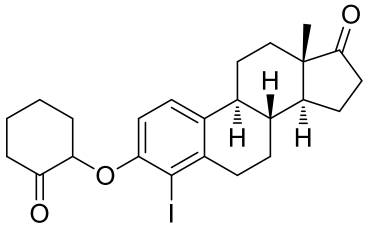

3a (*d.r.* = 1:1): white solid. (Decomposed at 182 °C). ^1H NMR (400 MHz, $CDCl_3$) δ 7.19-7.14 (m, 1H, two isomers), 6.57-6.51 (m, 1H, two isomers), 4.65-4.57 (m, 1H, two isomers), 3.00-2.91 (m, 1H, two isomers), 2.78-2.66 (m, 2H, two isomers), 2.56-2.46 (m, 1H, two isomers), 2.40-2.05 (m, 9H, two isomers), 1.97-1.89 (m, 3H, two isomers), 1.76-1.69 (m, 1H, two isomers), 1.64-1.55 (m, 1H, isomers), 1.55-1.36 (m, 5H, two isomers), 0.88 (s, 3H, two isomers); ^{13}C NMR (100 MHz, $CDCl_3$) δ 220.7 & 220.7 (two isomers), 208.8 & 208.7 (two isomers), 154.3 & 154.2 (two isomers), 140.7 & 140.7 (two isomers), 135.1 & 135.0 (two isomers), 126.1 & 126.0 (two isomers), 111.1 & 111.0 (two isomers), 96.2 & 96.1 (two isomers), 81.9 & 81.8 (two isomers), 50.4 & 50.3 (two isomers), 47.9 (overlap), 44.3 & 44.3 (two isomers), 39.9 (overlap), 37.6 & 37.6 (two isomers), 37.2 & 37.2 (two isomers), 35.9 (overlap), 34.5 (overlap), 31.5 (overlap), 28.1 & 28.0 (two isomers), 27.5 & 27.4 (two isomers), 26.2 & 26.1 (two isomers), 22.1 & 22.0 (two isomers), 21.6 (overlap), 13.8 (overlap). FT-IR: ν (cm^{-1}) 2925, 2862, 1732, 1684, 1465, 1425, 1394, 1273, 1257, 1208, 1083. HRMS [ESI] calcd for $C_{24}H_{29}INaO_3$ $[M+Na]^+$ 515.1054, found 515.1047.

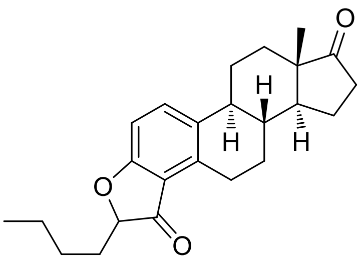

4a (*d.r.* = 1:1): colorless oil. ^{1}H NMR (400 MHz, $CDCl_3$) δ 7.52 (d, *J* = 8.8 Hz, 1H, two isomers), 6.88 (d, *J* = 8.4 Hz, 1H, two isomers), 4.50-4.45 (m, 1H, two isomers), 3.54-3.41 (m, 1H, two isomers), 3.08-2.92 (m, 1H, two isomers), 2.55-2.45 (m, 1H, two isomers), 2.42-2.34 (m, 1H, two isomers), 2.32-2.23 (m, 1H, two isomers), 2.20-2.11 (m, 1H, two isomers), 2.10-2.02 (m, 2H, two isomers), 2.01-1.92 (m, 2H, two isomers), 1.74-1.65 (m, 1H, two isomers), 1.63-1.32 (m, 10H, two isomers), 0.93-0.87 (m, 6H, two isomers); ^{13}C NMR (100 MHz, $CDCl_3$) δ 220.6 (overlap), 203.0 (overlap), 171.7 (overlap), 138.1 (overlap), 135.1 (overlap), 133.0 & 133.0 (two isomers), 118.3 & 118.2 (two isomers), 110.3 (overlap), 85.6 (overlap), 50.3 & 50.3 (two isomers), 47.9 (overlap), 43.9 & 43.8 (two isomers), 37.8 (overlap), 35.8 (overlap), 31.6 (overlap), 31.3 & 31.2 (two isomers), 26.9 (overlap), 26.3 & 26.3 (two isomers), 26.1 (overlap), 25.6 & 25.6 (two isomers), 22.5 (overlap), 21.6 (overlap), 13.9 (overlap), 13.9 (overlap). FT-IR: ν (cm^{-1}) 2958, 2931, 2872, 1726, 1705, 1653, 1621, 1597, 1478, 1431, 1258, 1223. HRMS [ESI] calcd for $C_{24}H_{31}O_3$ $[M+H]^+$ 367.2268, found 367.2273.

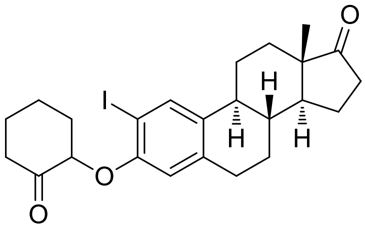

3b (*d.r.* = 1:1): white solid, m.p. 157-158 °C. ^{1}H NMR (400 MHz, $CDCl_3$) δ 7.64 (s, 1H, two isomers), 6.45 (d, *J* = 2.8 Hz, 1H, two isomers), 4.60-4.51 (m, 1H, two isomers), 2.84-2.74 (m, 2H, two isomers), 2.74-2.66 (m, 1H, two isomers), 2.55-2.45 (m, 1H, two isomers), 2.37-2.26 (m, 2H, two isomers), 2.26-2.08 (m, 5H, two isomers), 2.06-1.89 (m, 5H, two isomers), 1.77-1.68 (m, 1H, two isomers), 1.63-1.55 (m, 1H, two isomers), 1.55-1.33 (m, 5H, two isomers), 0.89 (s, 3H, two isomers); ^{13}C NMR (100 MHz, $CDCl_3$) δ 220.3 (overlap), 208.4 (overlap), 153.8 & 153.8 (two isomers), 137.7 & 137.6 (two isomers), 136.0 & 136.0 (two isomers), 134.9 & 134.9 (two isomers), 114.0 & 113.9 (two isomers), 83.5 & 83.4 (two isomers), 81.5 & 81.4 (two isomers), 49.8 & 49.8 (two isomers), 47.4 (overlap), 43.2 & 43.2 (two isomers), 39.5 (overlap), 37.5& 37.5 (two isomers), 35.4 (overlap), 34.0 (overlap), 31.0 (overlap), 29.0 & 29.0 (two isomers), 27.5 & 27.5 (two isomers), 25.8 & 25.8 (two isomers), 25.4 & 25.3 (two isomers), 21.5 & 21.5 (two isomers), 21.1 (overlap), 13.3 (overlap). FT-IR: ν (cm^{-1}) 2930, 2862, 1733, 1475, 1455, 1390, 1374, 1252, 1187, 1081. HRMS [ESI] calcd for $C_{24}H_{29}INaO_3$ $[M+Na]^+$ 515.1054, found 515.1048.

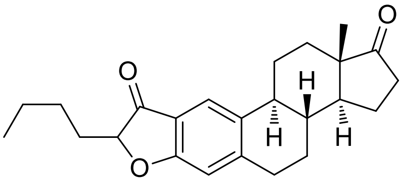

4b (*d.r.* = 1:1): colorless oil. ^{1}H NMR (400 MHz, $CDCl_3$) δ 7.56 (s, 1H, two isomers), 6.84 (s, 1H, two isomers), 4.52 (dd, *J* = 8.0, 4.0 Hz, 1H, two isomers), 3.02-2.94 (m, 2H, two isomers), 2.55-2.45 (m, 1H, two isomers), 2.44-2.37 (m, 1H, two isomers), 2.30-2.21 (m, 1H, two isomers), 2.20-2.11 (m, 1H, two isomers), 2.09-1.94 (m, 4H, two isomers), 1.81-1.65 (m, 2H, two isomers), 1.65-1.30 (m, 9H, two isomers), 0.92-0.86 (m, 6H, two isomers); ^{13}C NMR (100 MHz, $CDCl_3$) δ 220.5 (overlap), 201.9 (overlap), 171.0 & 171.0 (two isomers), 149.1 (overlap), 134.6 & 134.5 (two isomers), 120.5 (overlap), 119.0 (overlap), 112.6 & 112.6 (two isomers), 85.9 & 85.9 (two isomers), 50.5 (overlap), 47.9 (overlap), 43.8 (overlap), 37.9 & 37.9 (two isomers), 35.8 (overlap),

31.4 (overlap), 31.2 & 31.2 (two isomers), 30.6 (overlap), 26.8 & 26.8 (overlap), 26.1 (overlap), 25.8 & 25.7 (two isomers), 22.5 (overlap), 21.6 (overlap), 13.9 (overlap), 13.8 (overlap). FT-IR: ν (cm^{-1}) 2930, 2862, 1737, 1707, 1620, 1589, 1468, 1447, 1246, 1198. HRMS [ESI] calcd for $C_{24}H_{31}O_3$ $[M+H]^+$ 367.2268, found 367.2274.

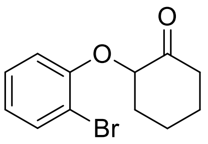

8a: white solid, m.p. 107-108 °C. ^1H NMR (400 MHz, $CDCl_3$) δ 7.53 (dd, *J* = 8.0, 1.6 Hz, 1H), 7.21-7.15 (m, 1H), 6.87-6.78 (m, 2H), 4.65-4.60 (m, 1H), 2.71-2.63 (m, 1H), 2.37-2.07 (m, 4H), 2.00-1.83 (m, 2H), 1.78-1.68 (m, 1H); ^{13}C NMR (100 MHz, $CDCl_3$) δ 208.1, 154.2, 133.5, 128.4, 122.9, 115.7, 113.0, 81.9, 40.1, 34.4, 27.9, 22.3. FT-IR: ν (cm^{-1}) 2951, 2862, 2359, 2342, 1720, 1685, 1585, 1573, 1473, 1364, 1273, 1244, 1018. HRMS [ESI] calcd for $C_{12}H_{13}BrNaO_2$ $[M+Na]^+$ 290.9991, found 290.9984.

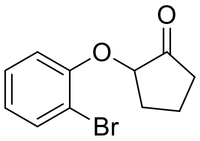

8b: yellow oil. ^1H NMR (400 MHz, $CDCl_3$) δ 7.53-7.47 (m, 1H), 7.25-7.18 (m, 1H), 7.08-7.01 (m, 1H), 6.89-6.81 (m, 1H), 4.58 (dd, *J* = 8.8, 7.6 Hz, 1H), 2.48-2.28 (m, 3H), 2.20-2.02 (m, 2H), 1.95-1.82 (m, 1H); ^{13}C NMR (100 MHz, $CDCl_3$) δ 213.4, 154.6, 133.4, 128.5, 123.2, 116.3, 113.1, 80.9, 35.3, 29.6, 17.4. FT-IR: ν (cm^{-1}) 3064, 2972, 2884, 1751, 1584, 1573, 1474, 1442, 1401, 1273, 1245, 1125, 1092. HRMS [ESI] calcd for $C_{11}H_{11}BrNaO_2$ $[M+Na]^+$ 276.9835, found 276.9845.

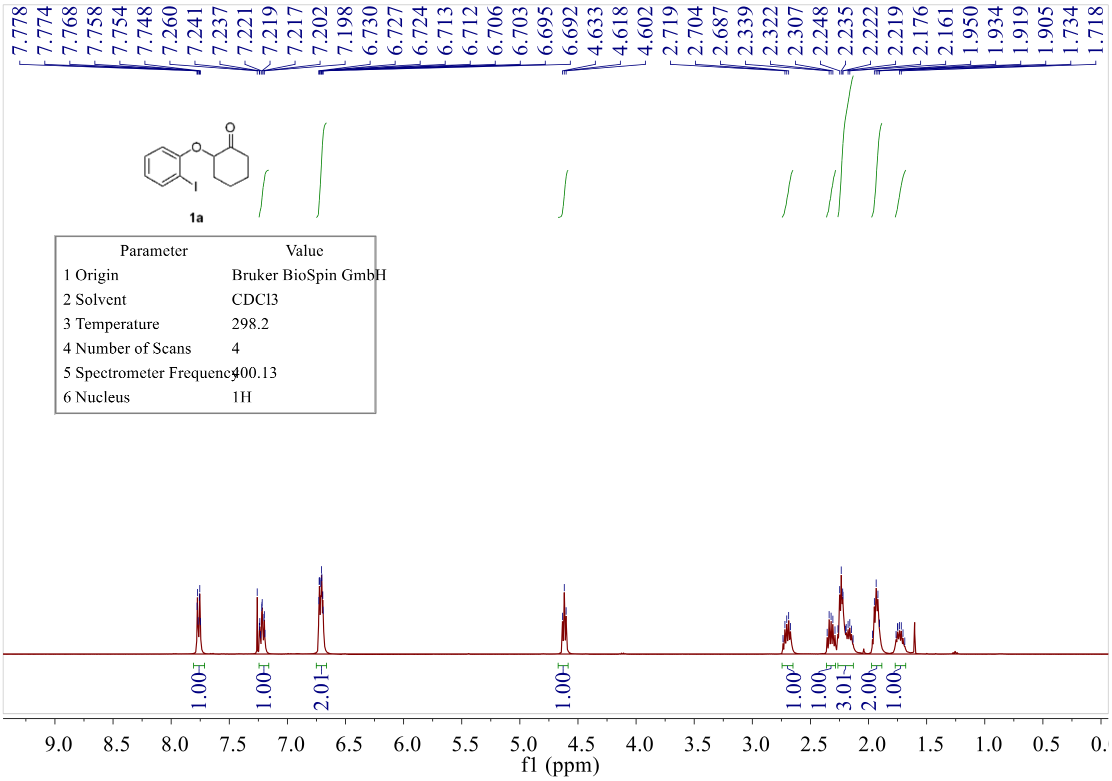

Supplementary Figure 14. ^{1}H NMR (400 MHz, $CDCl_3$) spectra for compound 1a

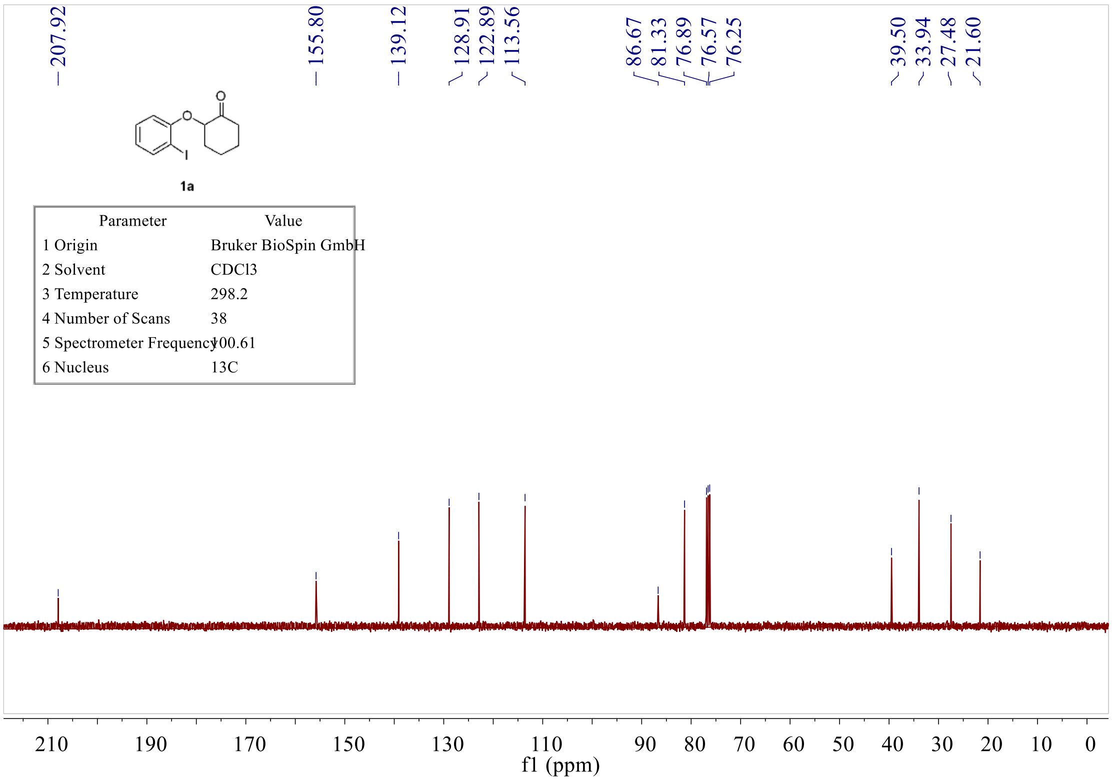

Supplementary Figure 15. ^{13}C NMR (100 MHz, $CDCl_3$) spectra for compound 1a

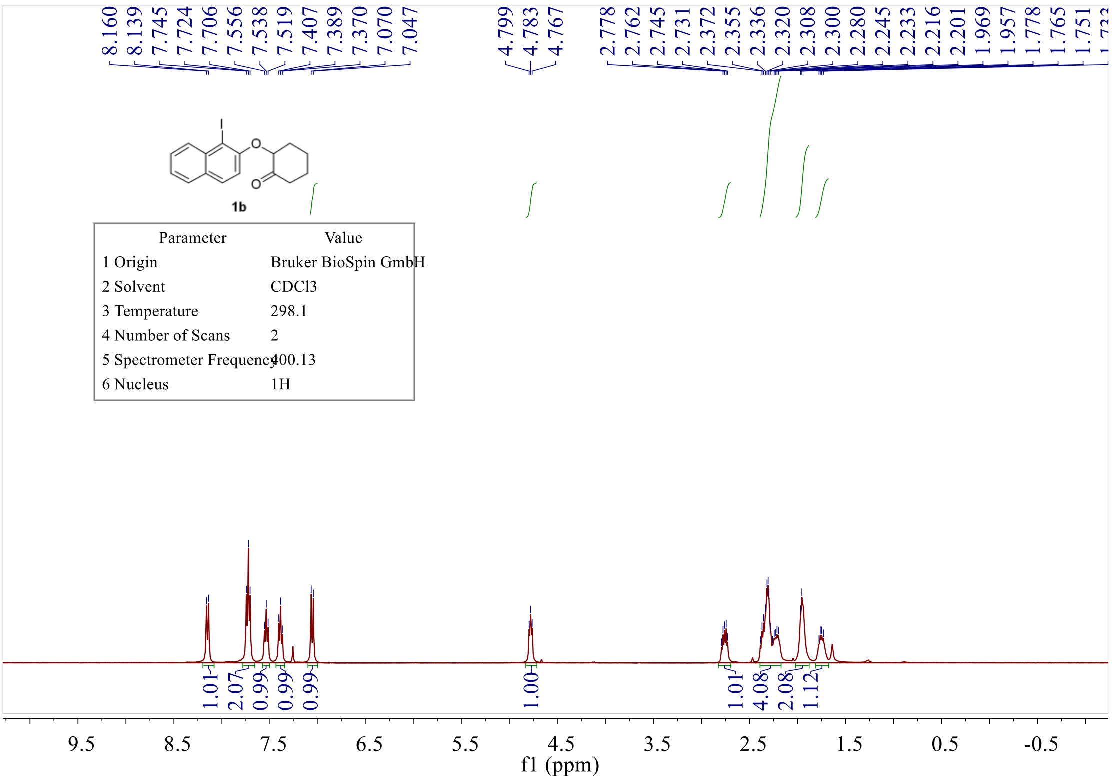

Supplementary Figure 16. ^{1}H NMR (400 MHz, $CDCl_3$) spectra for compound 1b

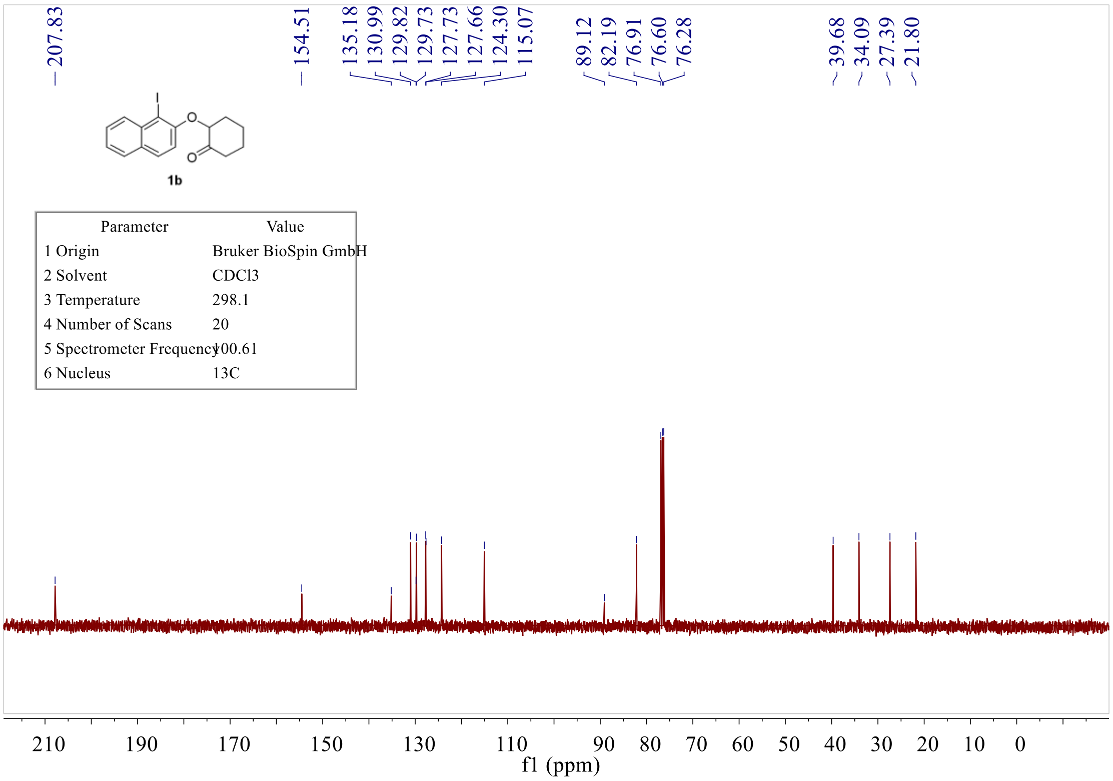

Supplementary Figure 17. ^{13}C NMR (100 MHz, $CDCl_3$) spectra for compound 1b

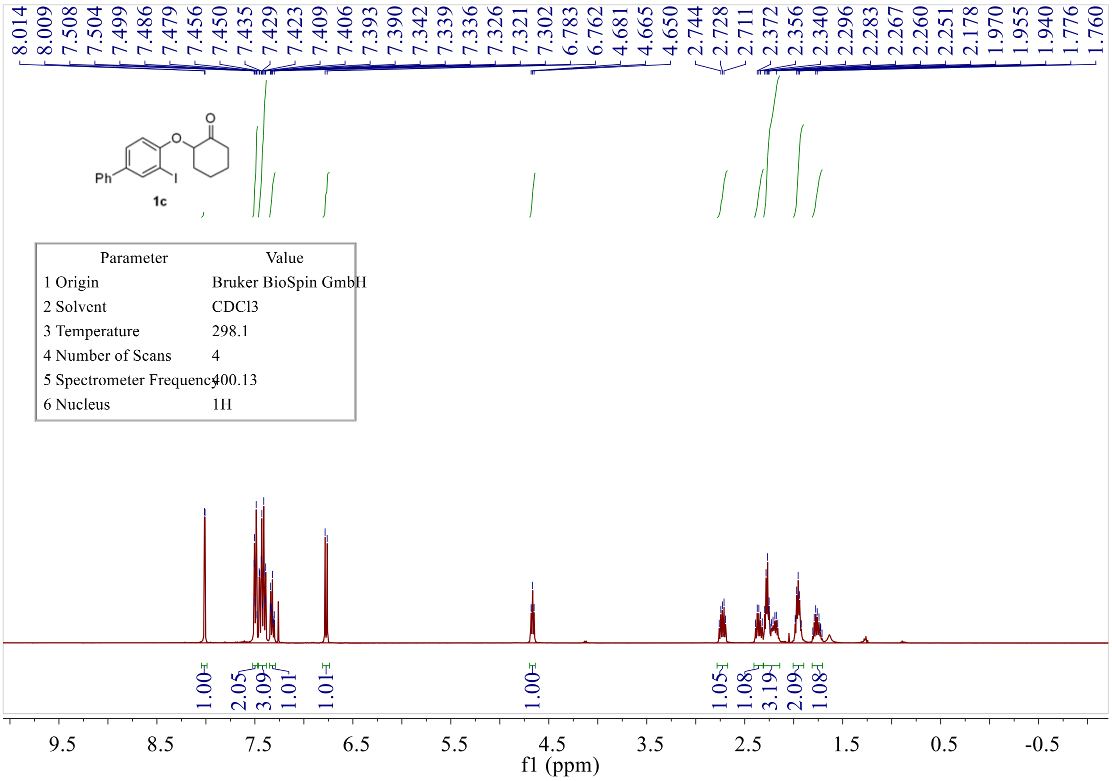

Supplementary Figure 18. ^{1}H NMR (400 MHz, $CDCl_3$) spectra for compound 1c

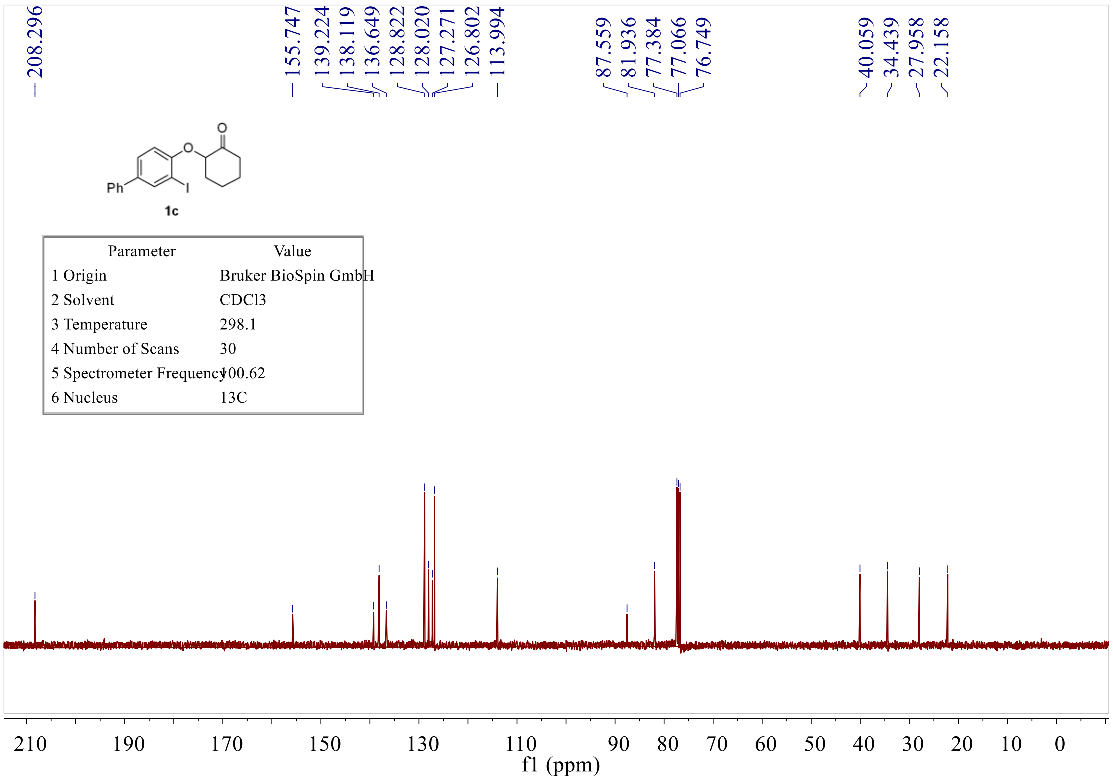

Supplementary Figure 19. ^{13}C NMR (100 MHz, $CDCl_3$) spectra for compound 1c

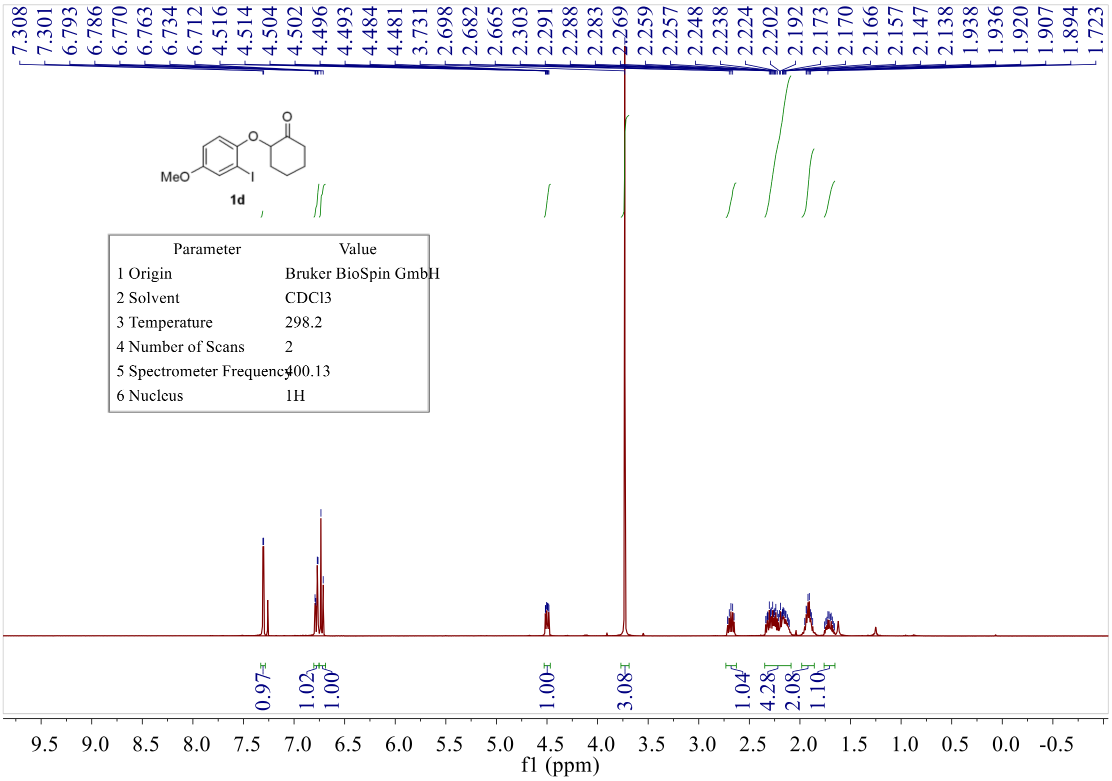

Supplementary Figure 20. **^{1}H NMR (400 MHz, $CDCl_3$) spectra for compound 1d**

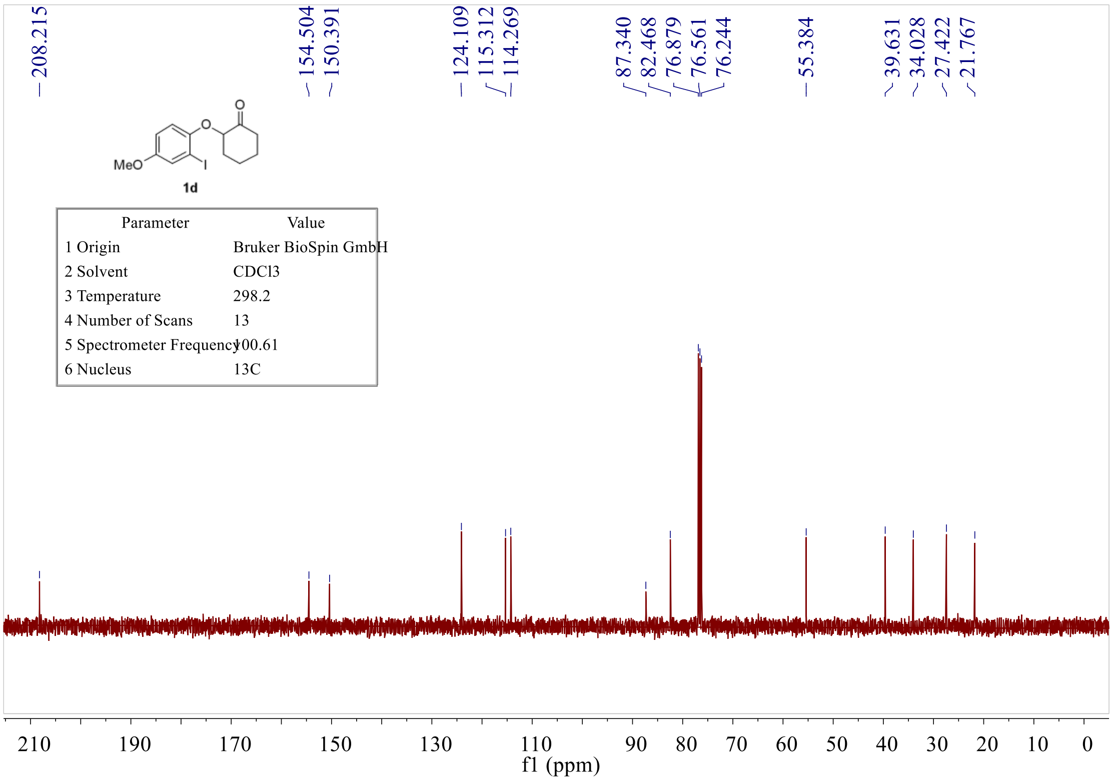

Supplementary Figure 21. **^{13}C NMR (100 MHz, $CDCl_3$) spectra for compound 1d**

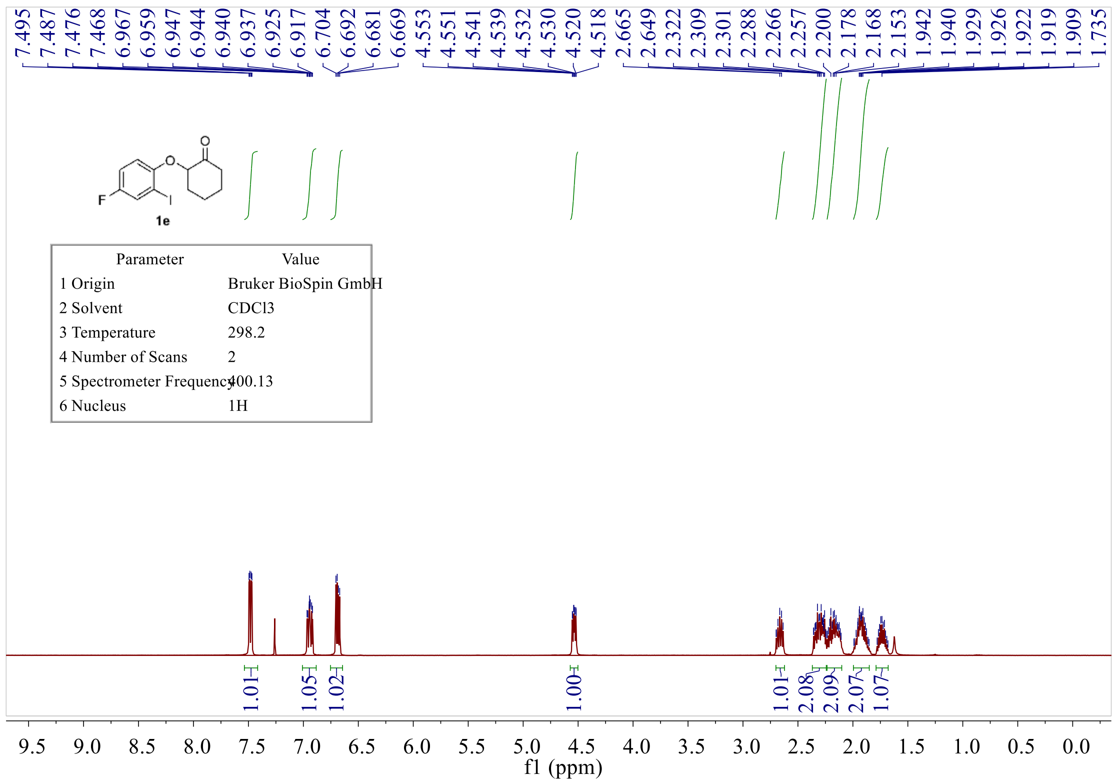

Supplementary Figure 22. ^{1}H NMR (400 MHz, CDCl$_3$) spectra for compound 1e

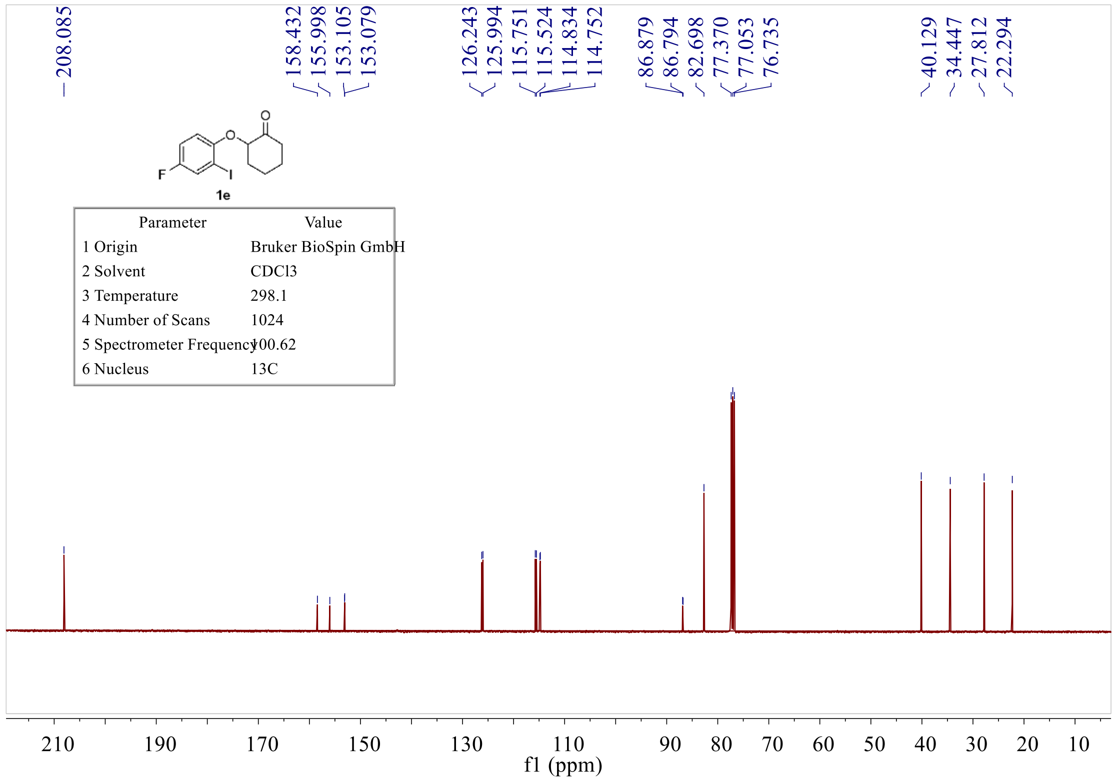

Supplementary Figure 23. ^{13}C NMR (100 MHz, CDCl$_3$) spectra for compound 1e

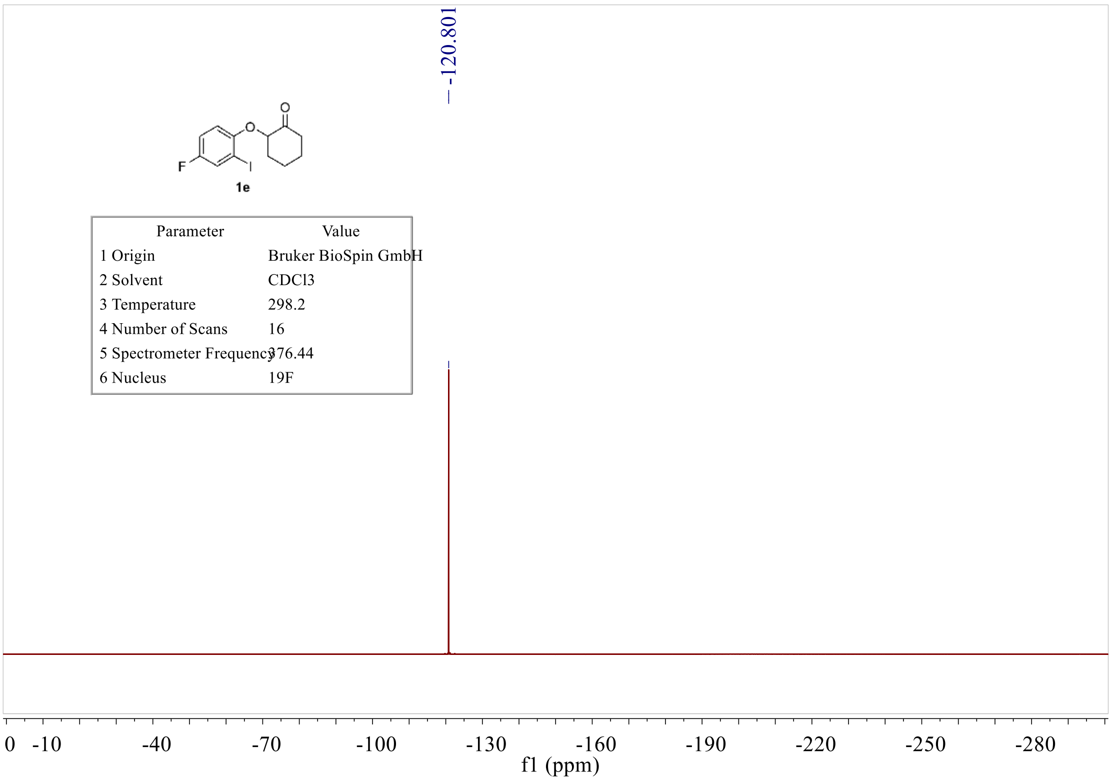

Supplementary Figure 24. ^{19}**F NMR (376 MHz, CDCl$_3$) spectra for compound 1e**

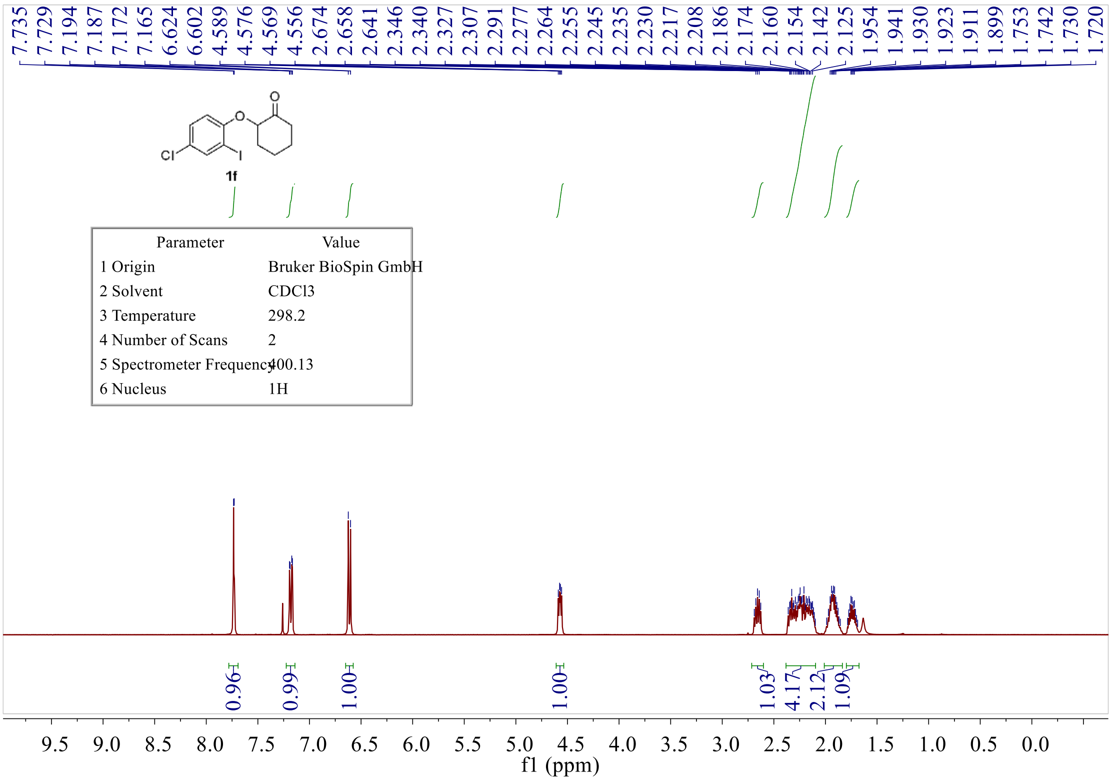

Supplementary Figure 25. ^{1}**H NMR (400 MHz, CDCl$_3$) spectra for compound 1f**

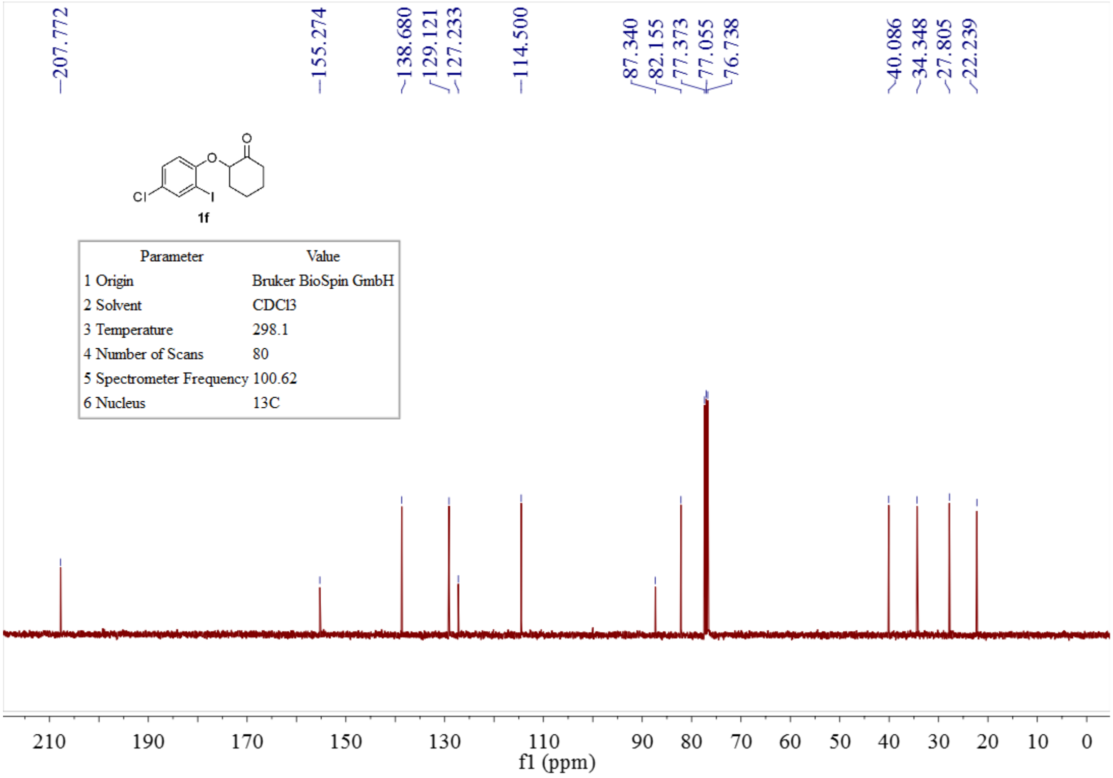

Supplementary Figure 26. ^{13}C NMR (100 MHz, $CDCl_3$) spectra for compound 1f

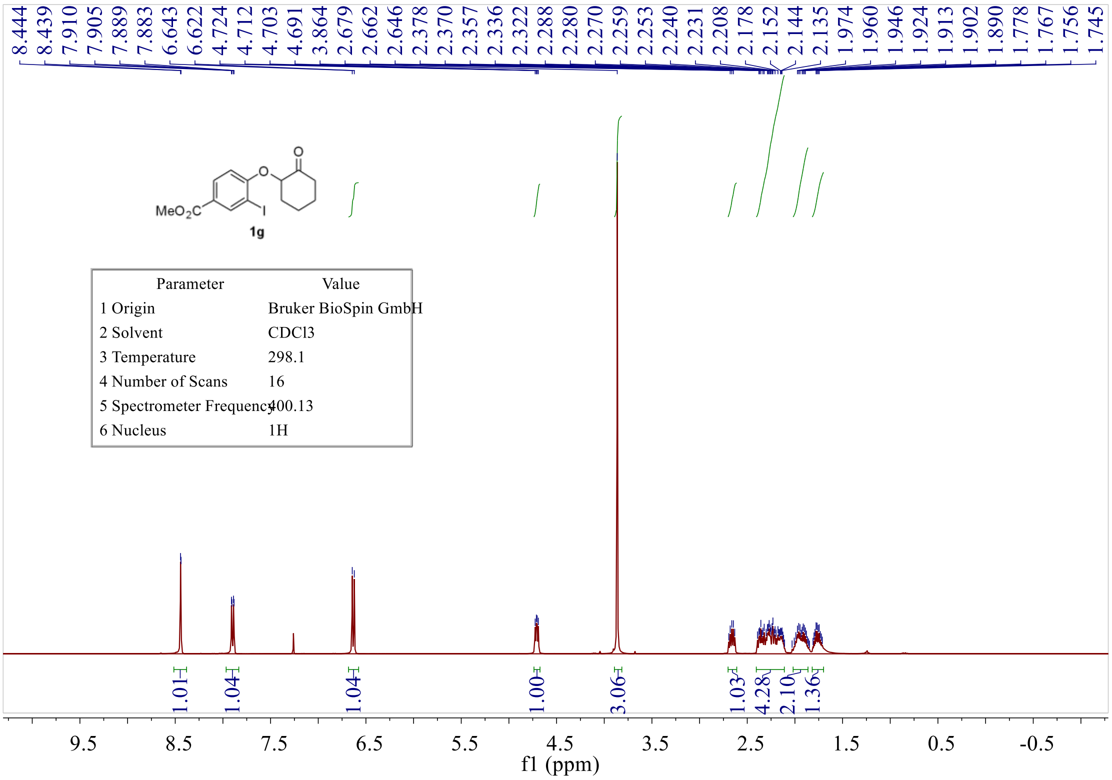

Supplementary Figure 27. ^{1}H NMR (400 MHz, $CDCl_3$) spectra for compound 1g

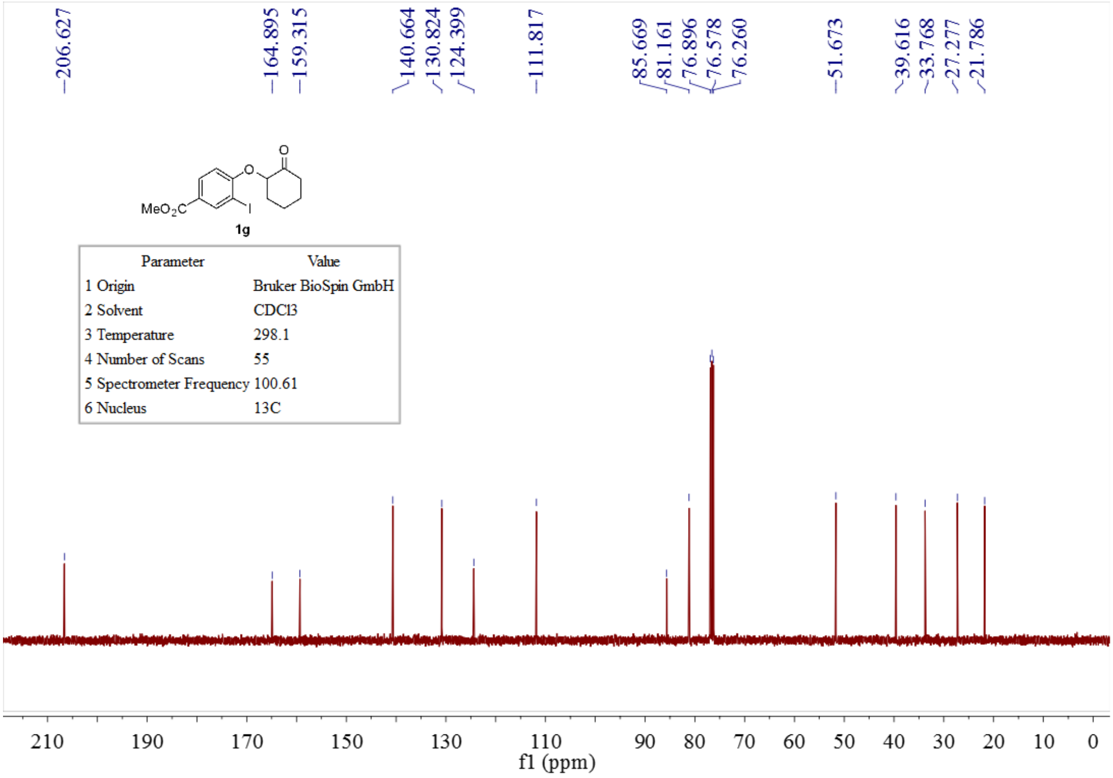

Supplementary Figure 28. ^{13}C **NMR (100 MHz,** $CDCl_3$**) spectra for compound 1g**

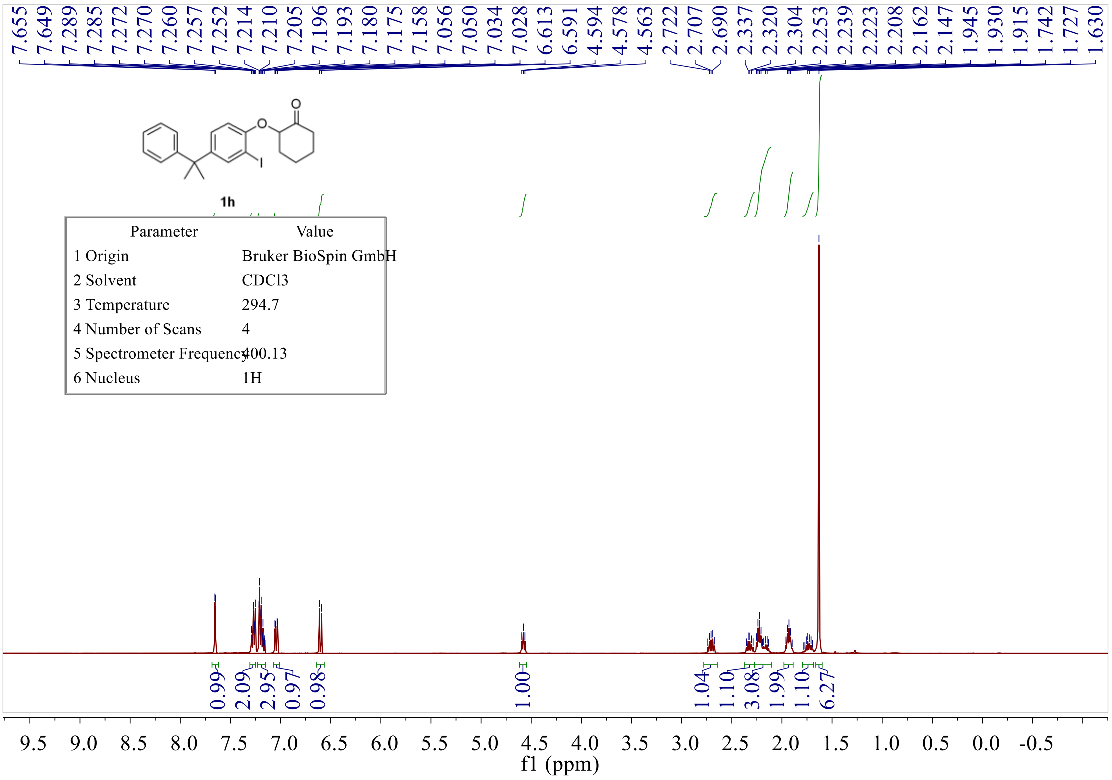

Supplementary Figure 29. ^{1}H **NMR (400 MHz,** $CDCl_3$**) spectra for compound 1h**

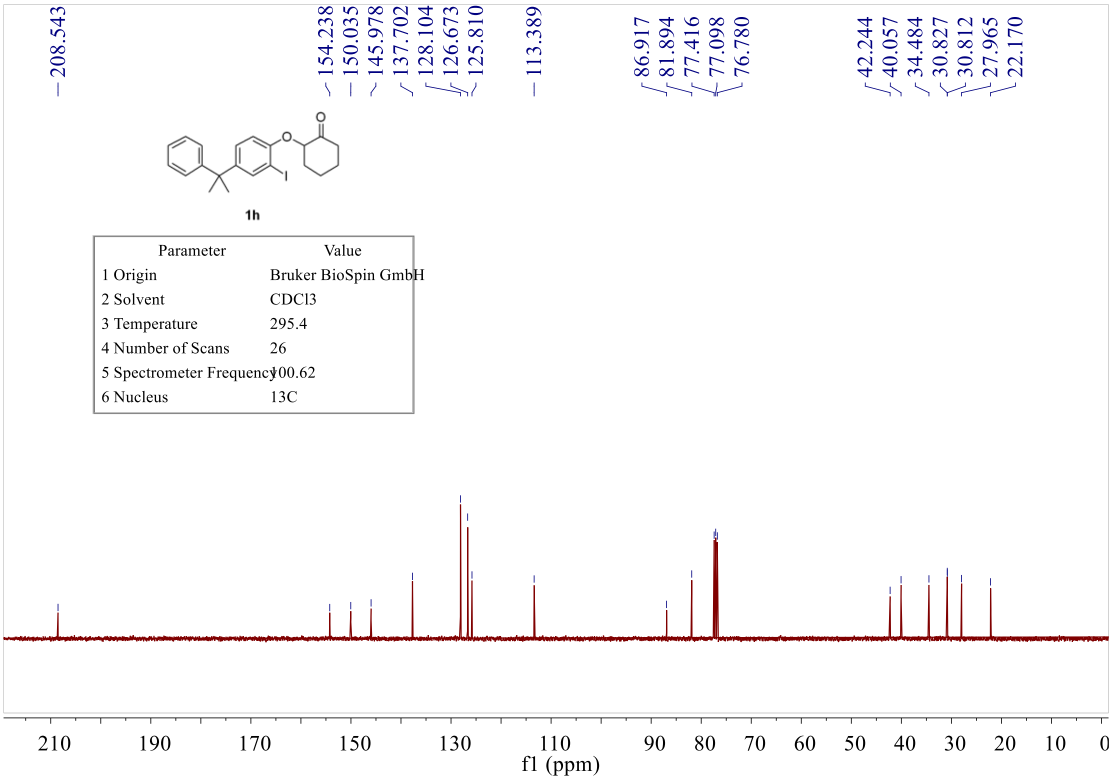

Supplementary Figure 30. ^{13}**C NMR (100 MHz, CDCl**$_3$**) spectra for compound 1h**

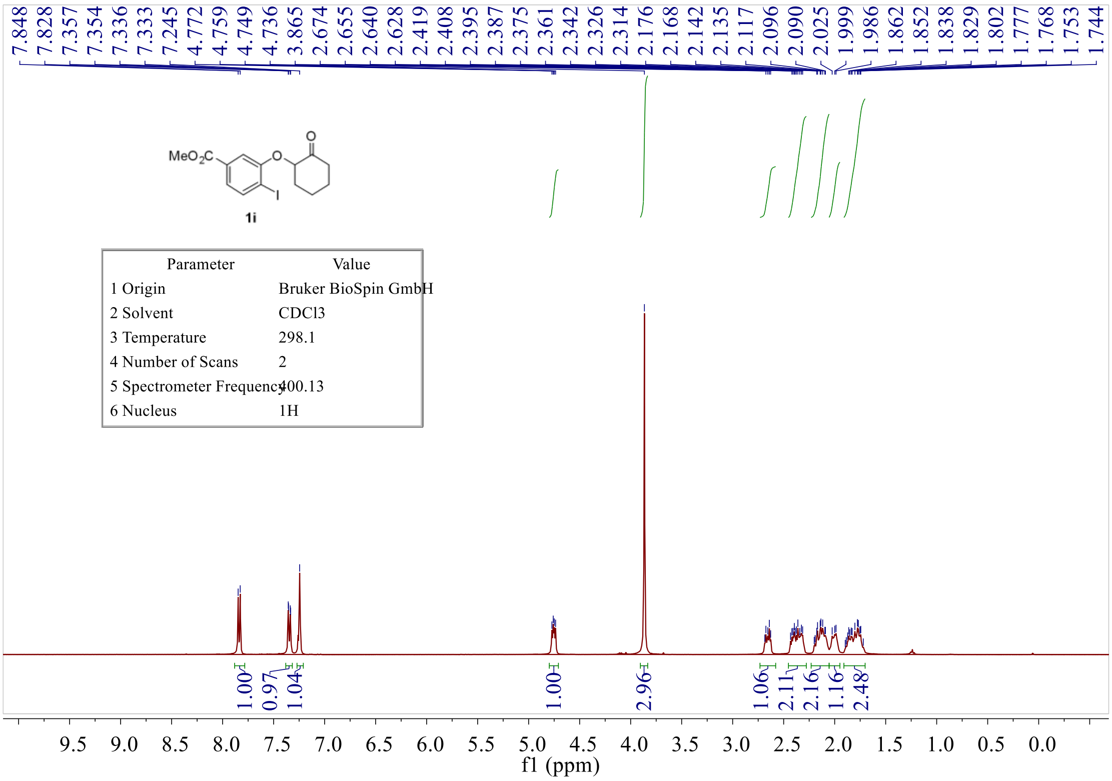

Supplementary Figure 31. ^{1}**H NMR (400 MHz, CDCl**$_3$**) spectra for compound 1i**

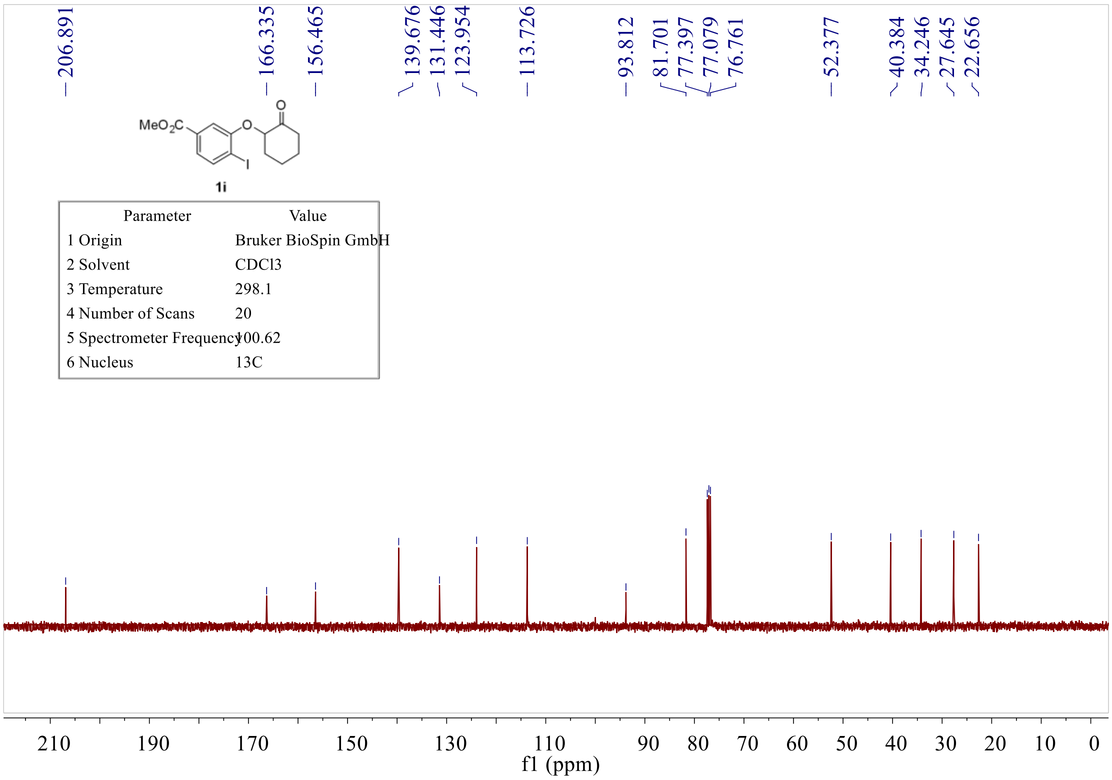

Supplementary Figure 32. ^{13}C NMR (100 MHz, CDCl$_3$) spectra for compound 1i

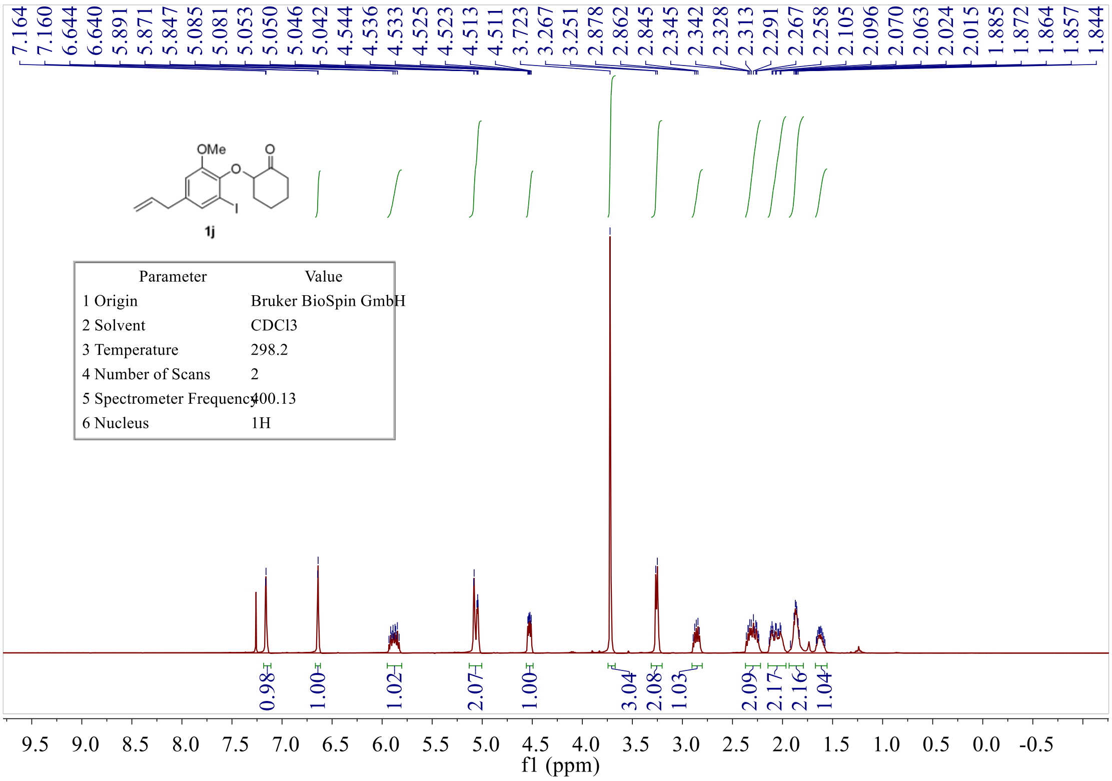

Supplementary Figure 33. ^{1}H NMR (400 MHz, CDCl$_3$) spectra for compound 1j

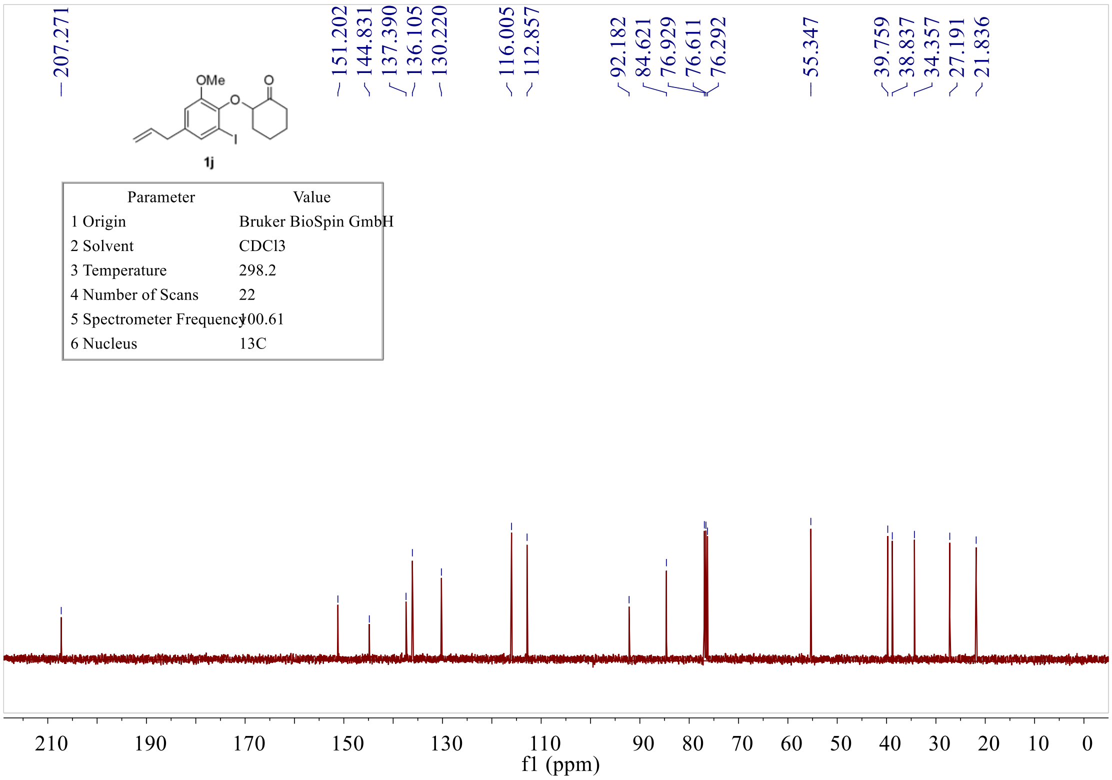

Supplementary Figure 34. ^{13}**C NMR (100 MHz, CDCl$_3$) spectra for compound 1j**

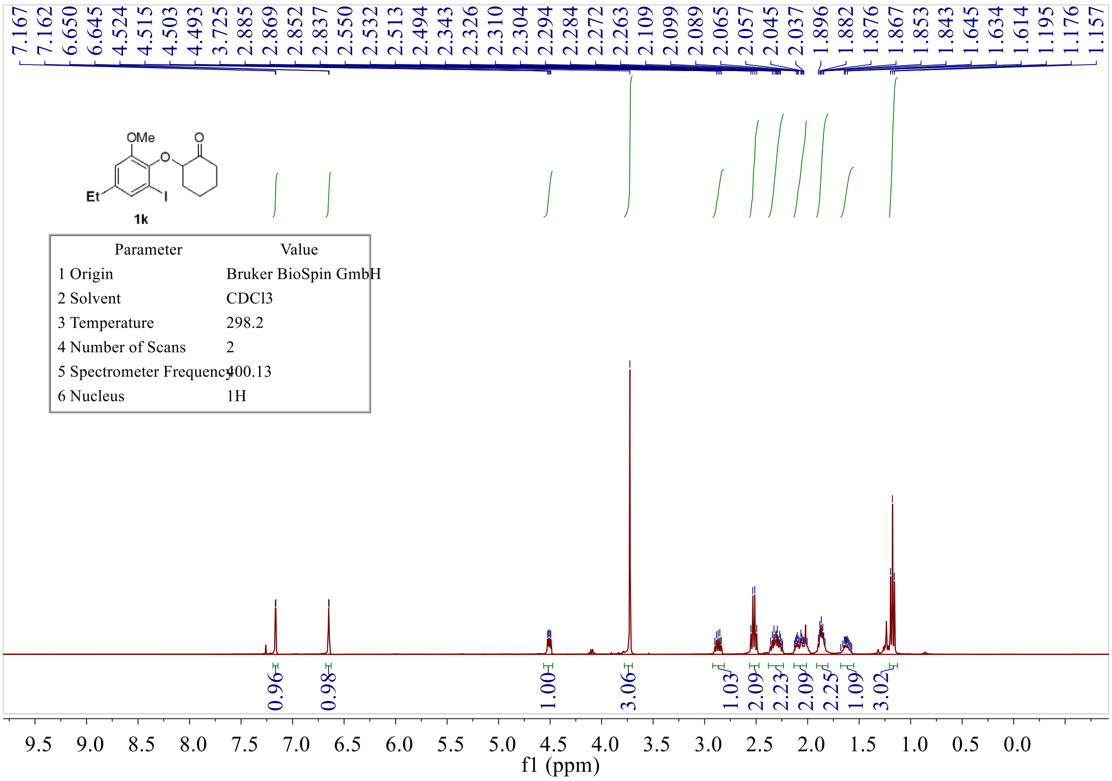

Supplementary Figure 35. ^{1}**H NMR (400 MHz, CDCl$_3$) spectra for compound 1k**

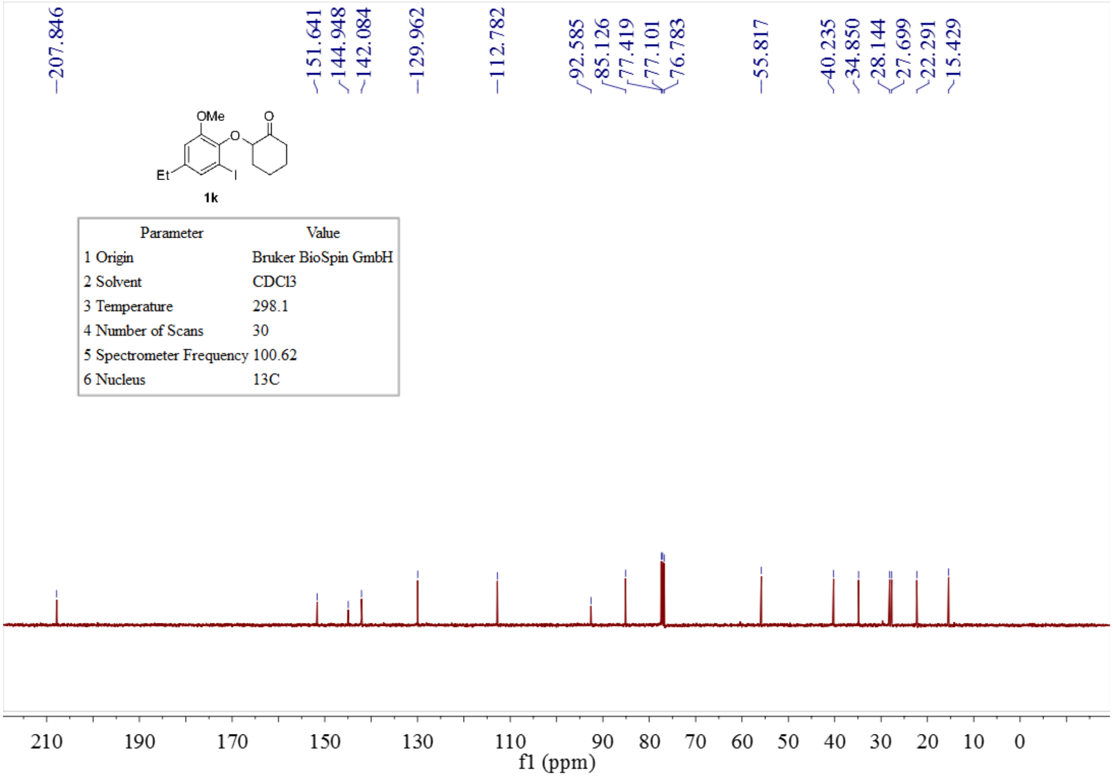

Supplementary Figure 36. ^{13}C NMR (100 MHz, $CDCl_3$) spectra for compound 1k

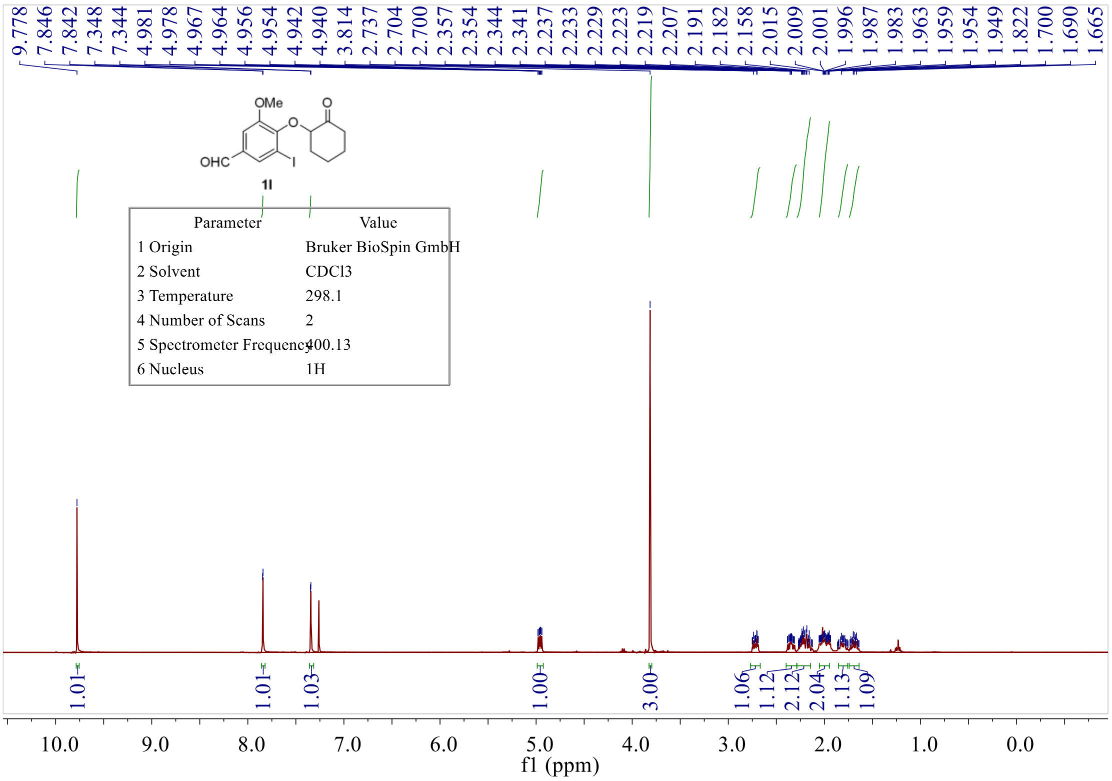

Supplementary Figure 37. ^{1}H NMR (400 MHz, $CDCl_3$) spectra for compound 1l

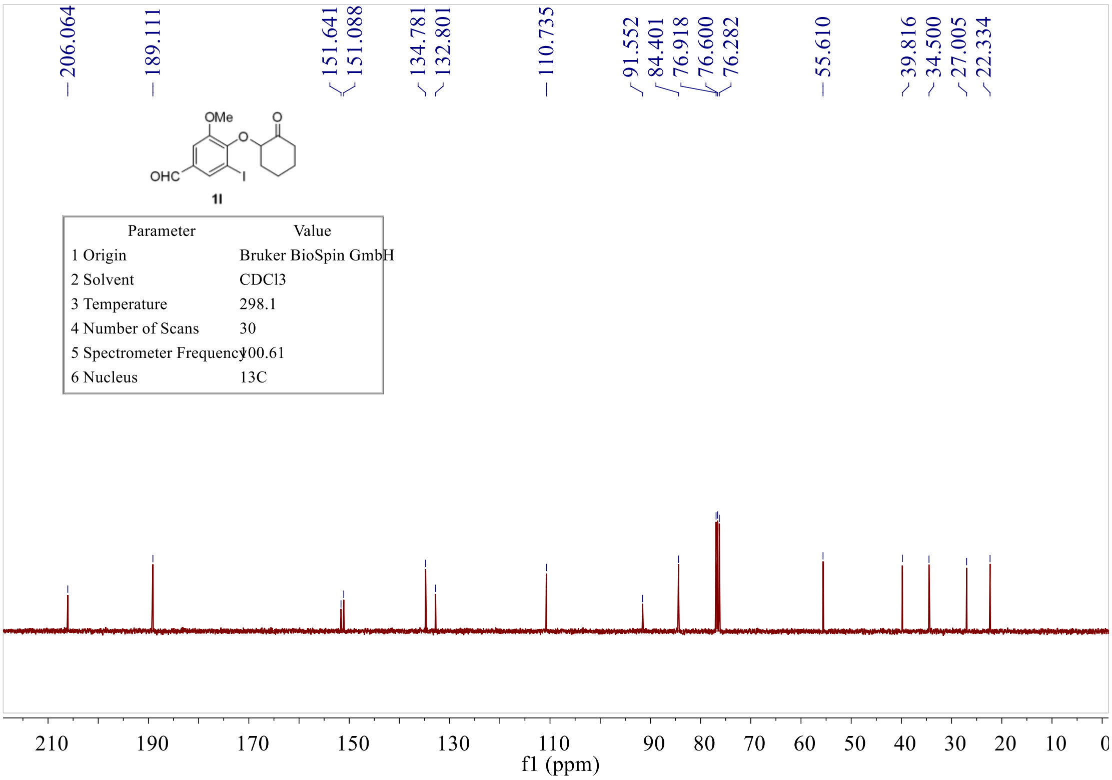

Supplementary Figure 38. ^{13}**C NMR (100 MHz, CDCl$_3$) spectra for compound 1l**

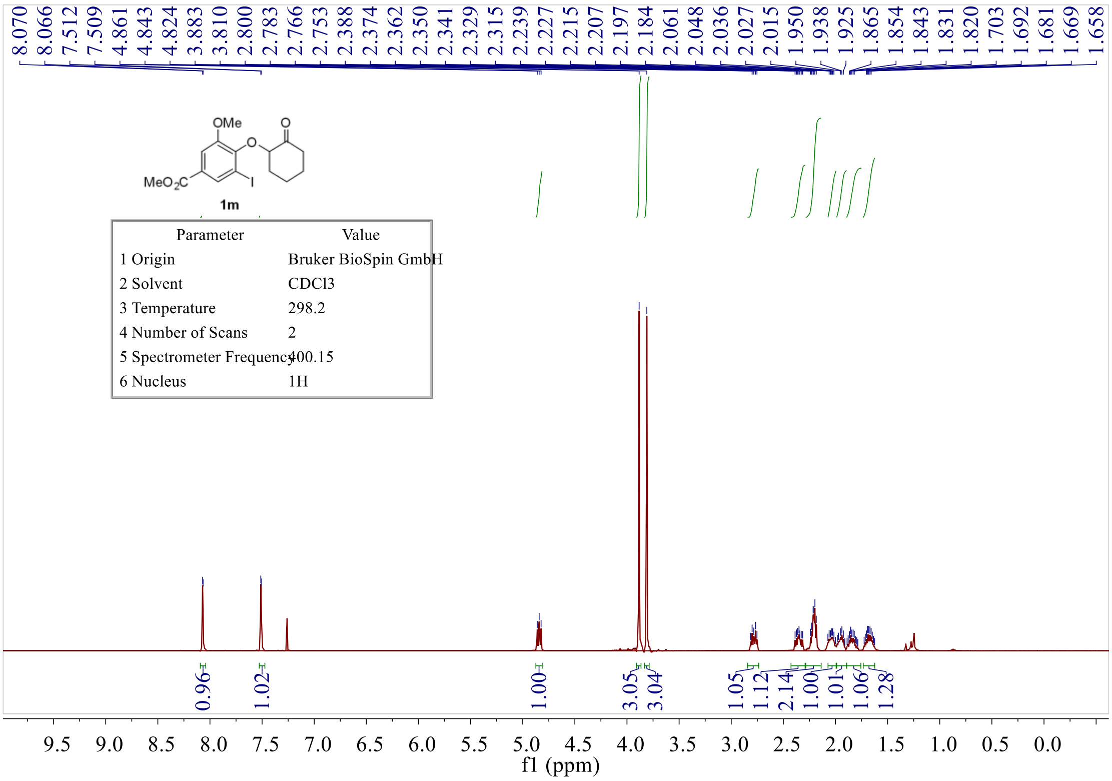

Supplementary Figure 39. ^{1}**H NMR (400 MHz, CDCl$_3$) spectra for compound 1m**

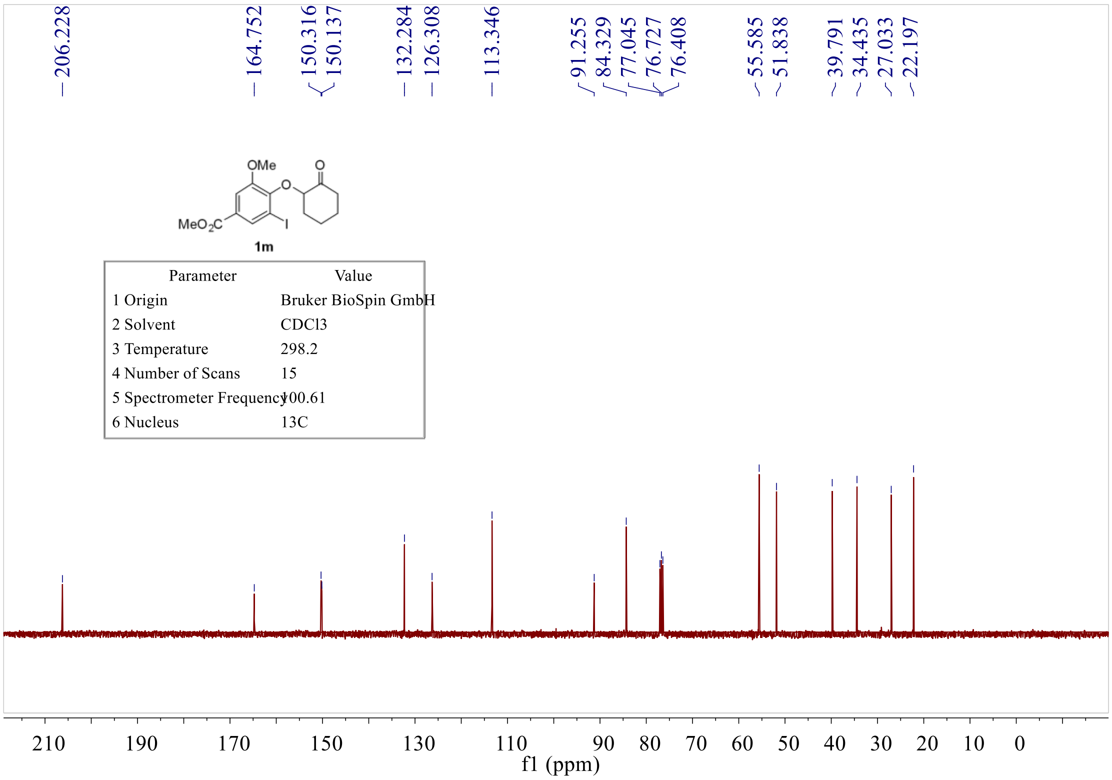

Supplementary Figure 40. ^{13}**C NMR (100 MHz, CDCl**$_3$**) spectra for compound 1m**

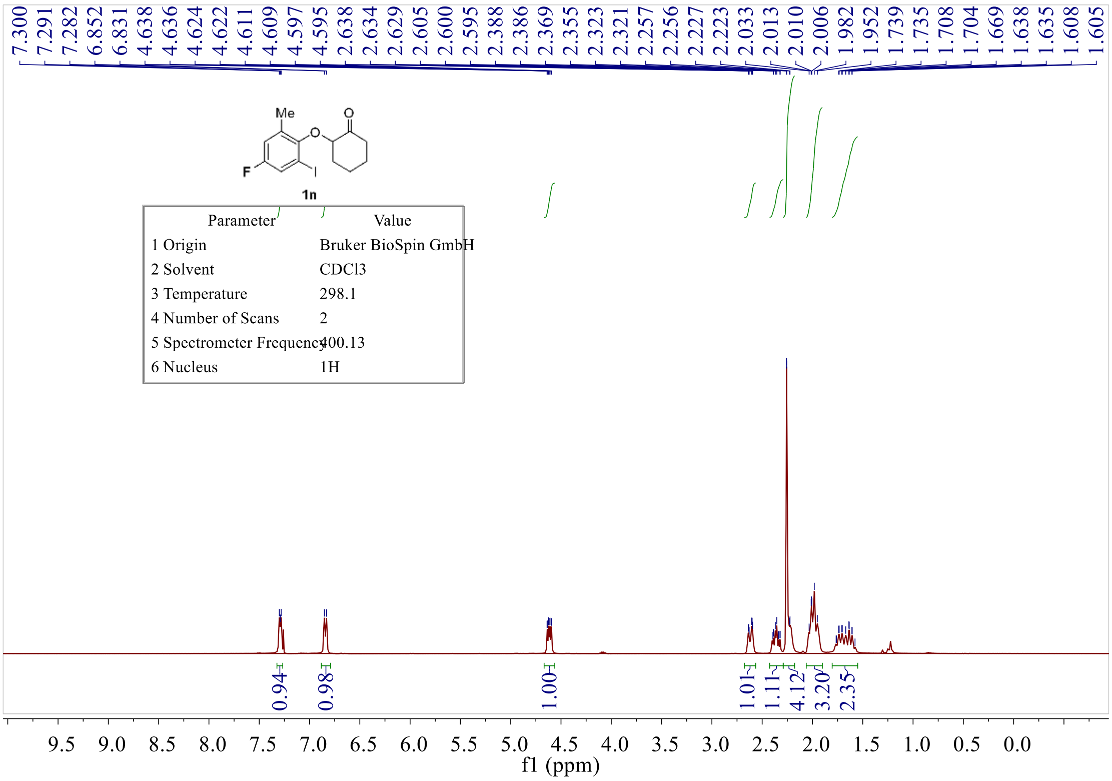

Supplementary Figure 41. ^{1}**H NMR (400 MHz, CDCl**$_3$**) spectra for compound 1n**

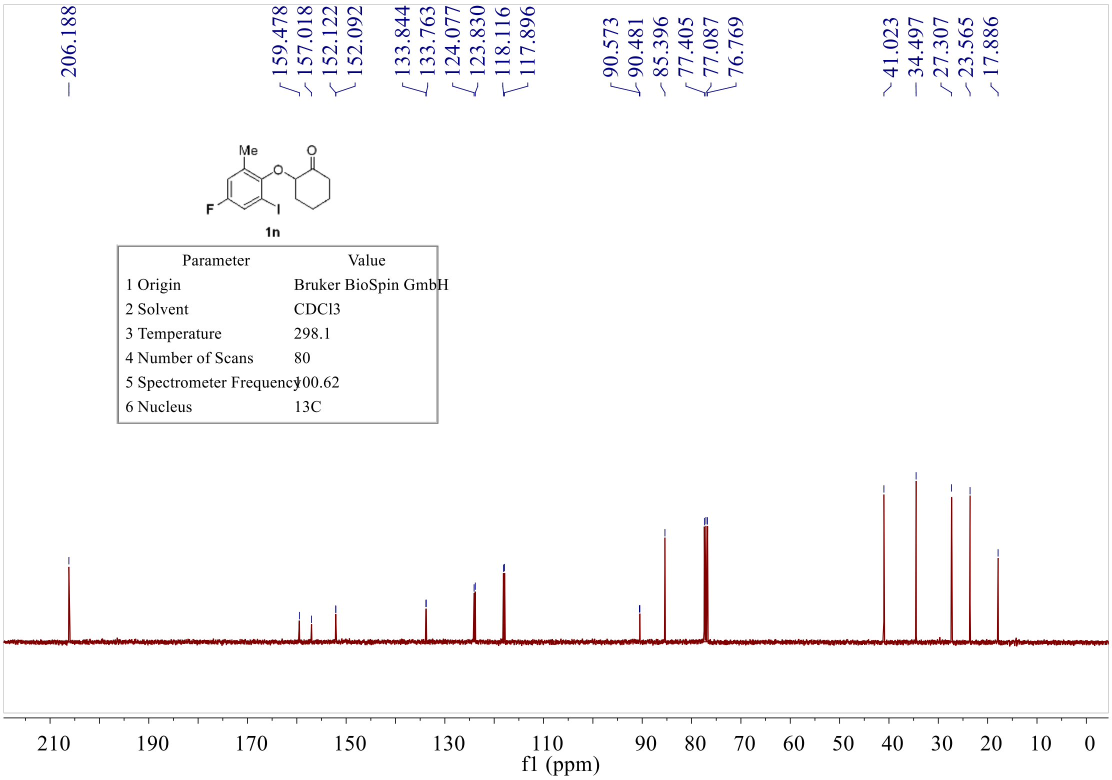

Supplementary Figure 42. ^{13}C NMR (100 MHz, $CDCl_3$) spectra for compound 1n

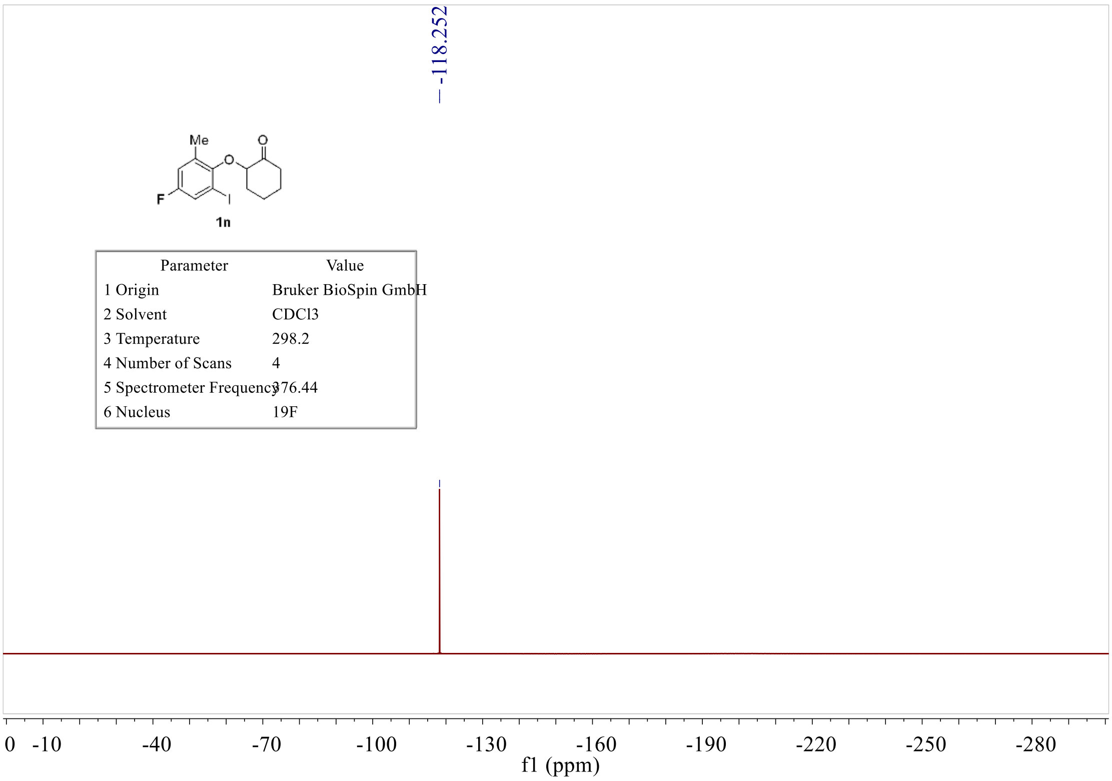

Supplementary Figure 43. ^{19}F NMR (376 MHz, $CDCl_3$) spectra for compound 1n

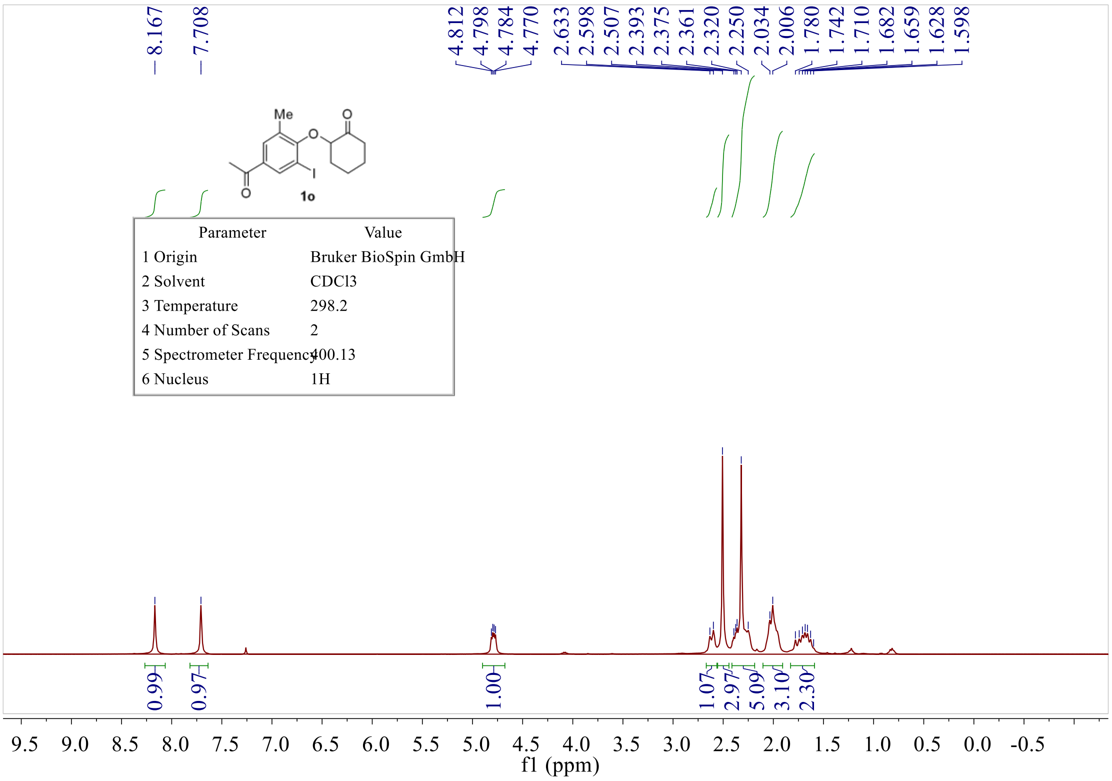

Supplementary Figure 44. ^{1}H NMR (400 MHz, $CDCl_3$) spectra for compound 1o

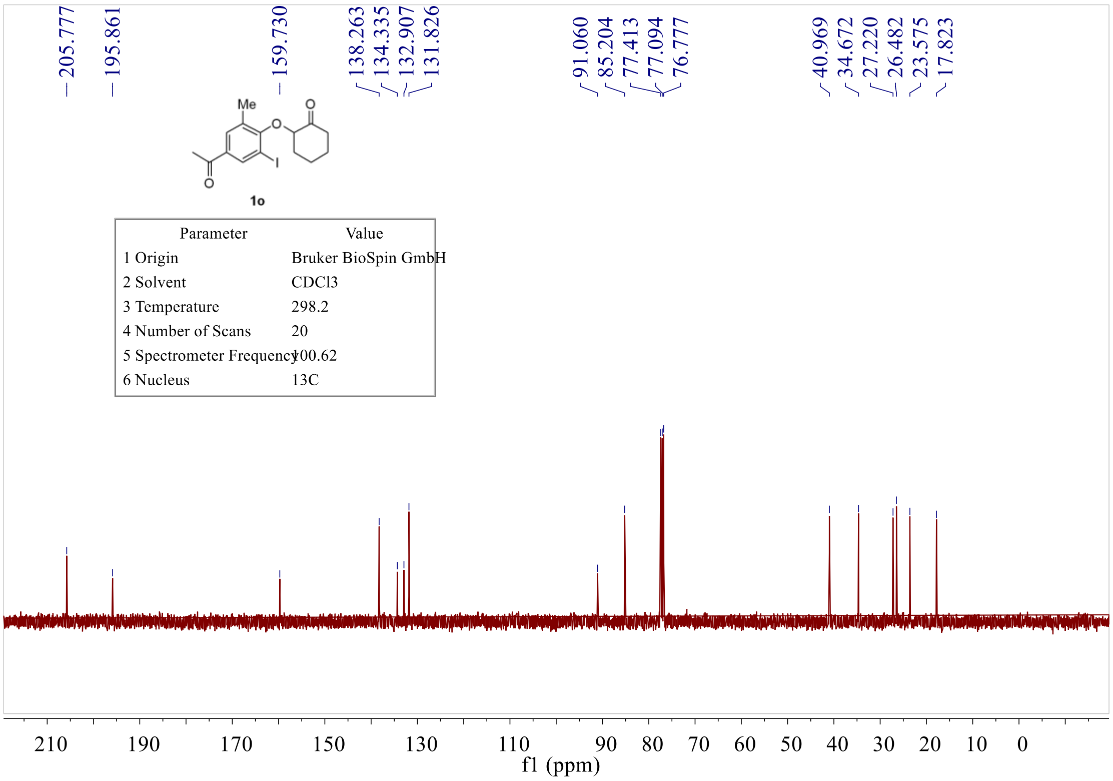

Supplementary Figure 45. ^{13}C NMR (100 MHz, $CDCl_3$) spectra for compound 1o

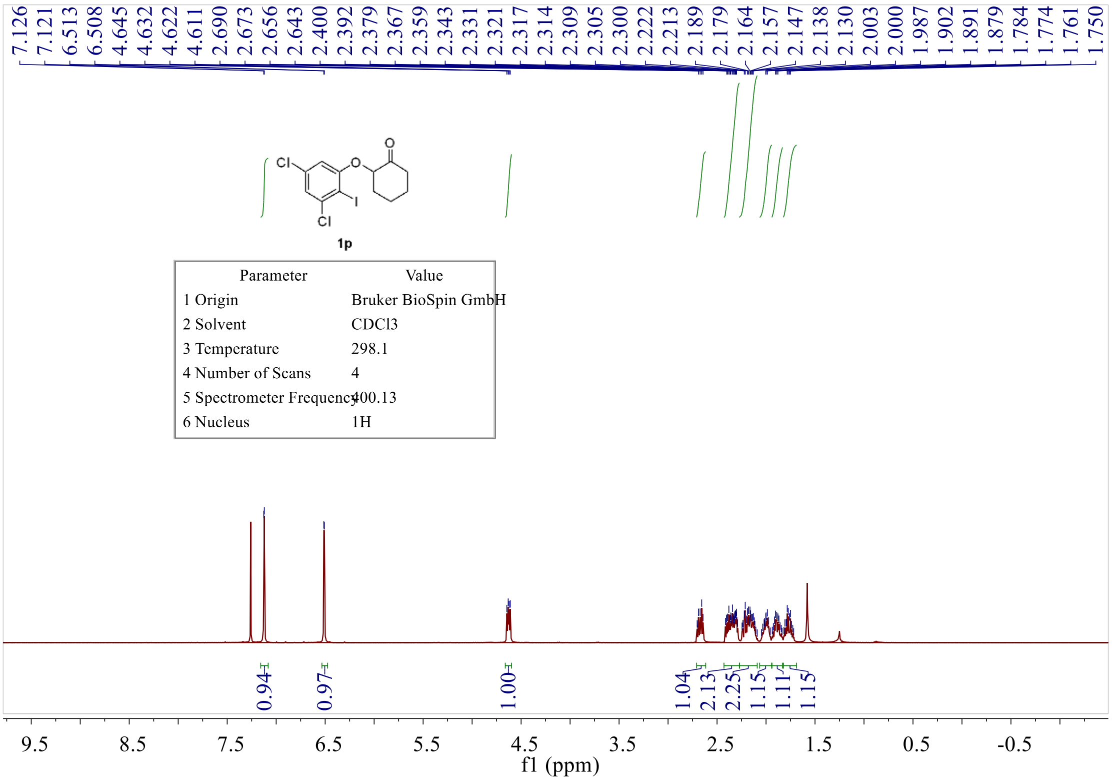

Supplementary Figure 46. ^{1}H NMR (400 MHz, CDCl$_3$) spectra for compound 1p

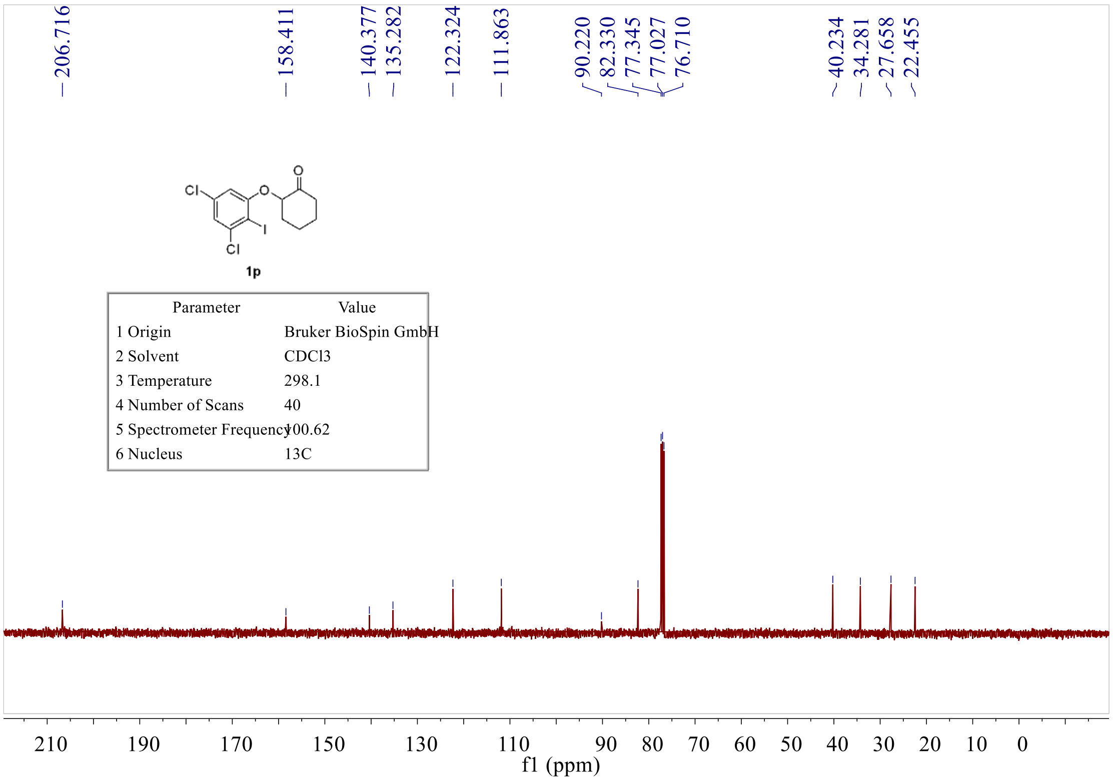

Supplementary Figure 47. ^{13}C NMR (100 MHz, CDCl$_3$) spectra for compound 1p

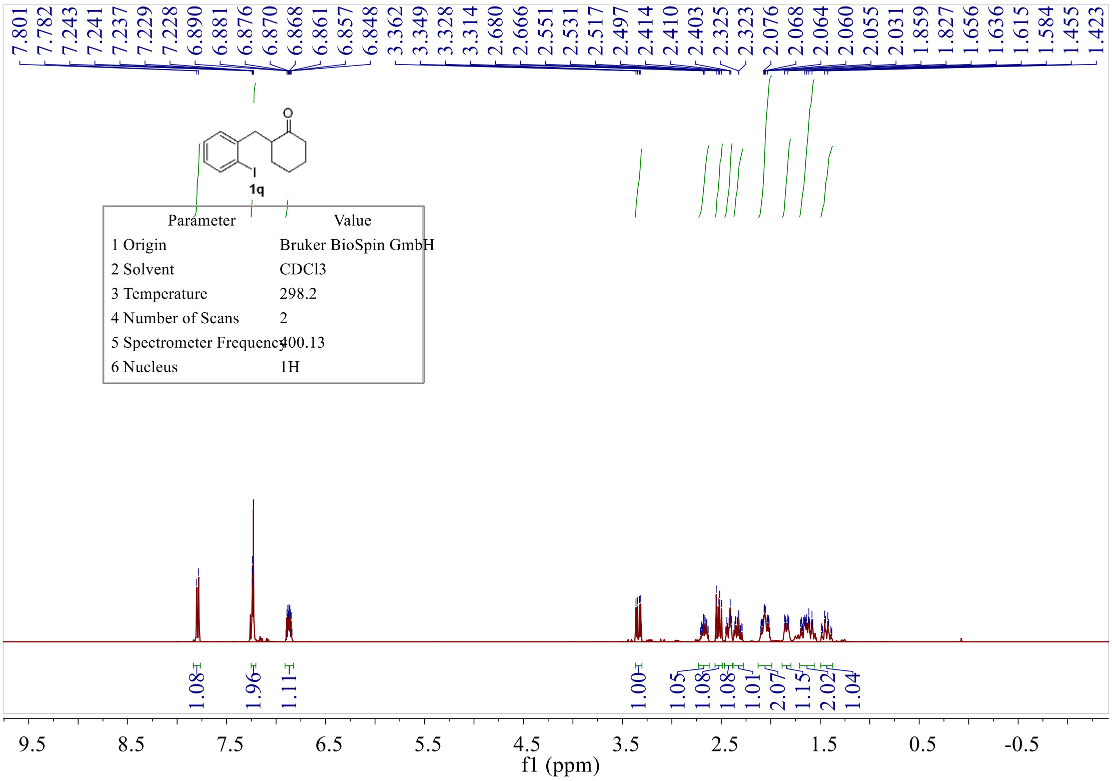

Supplementary Figure 48. ^{1}**H NMR (400 MHz,** $CDCl_3$**) spectra for compound 1q**

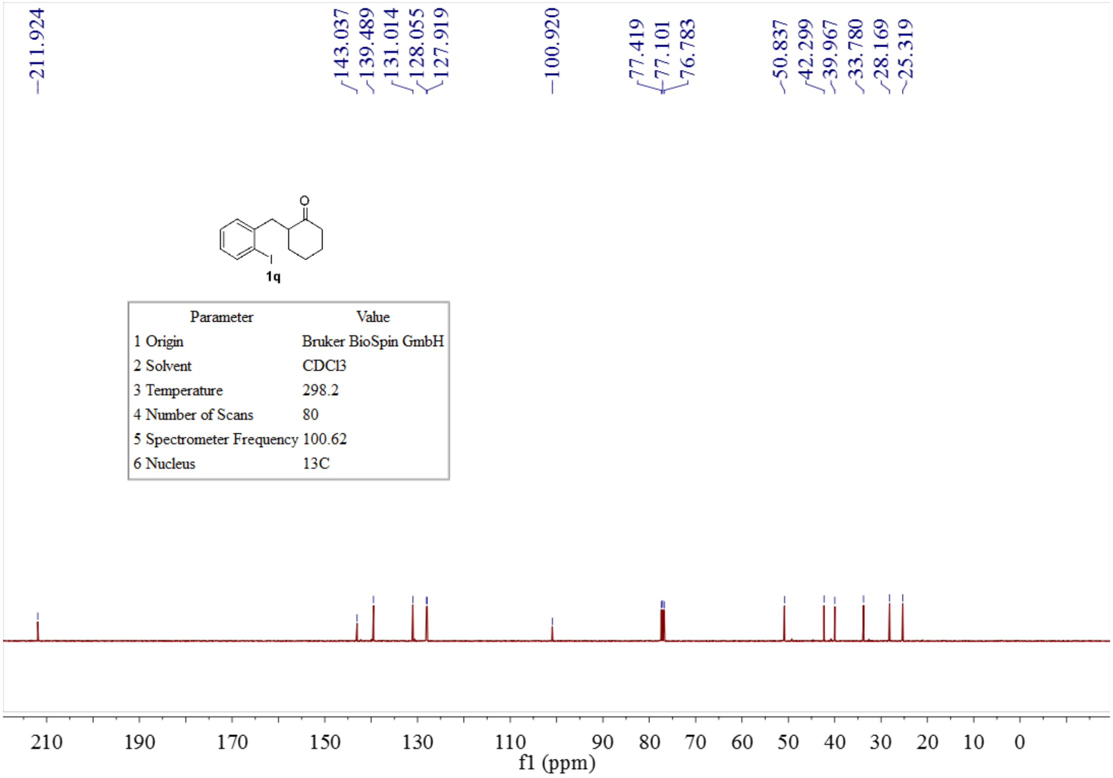

Supplementary Figure 49. ^{13}**C NMR (100 MHz,** $CDCl_3$**) spectra for compound 1q**

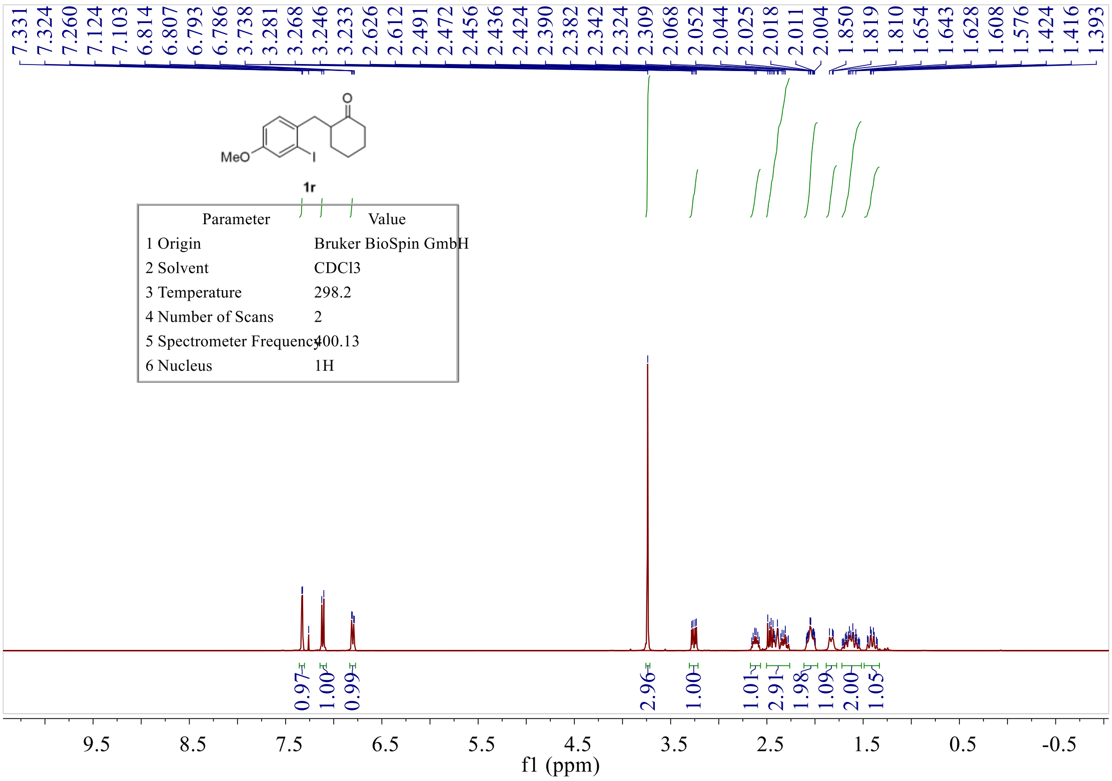

Supplementary Figure 50. ^{1}H **NMR (400 MHz, $CDCl_3$) spectra for compound 1r**

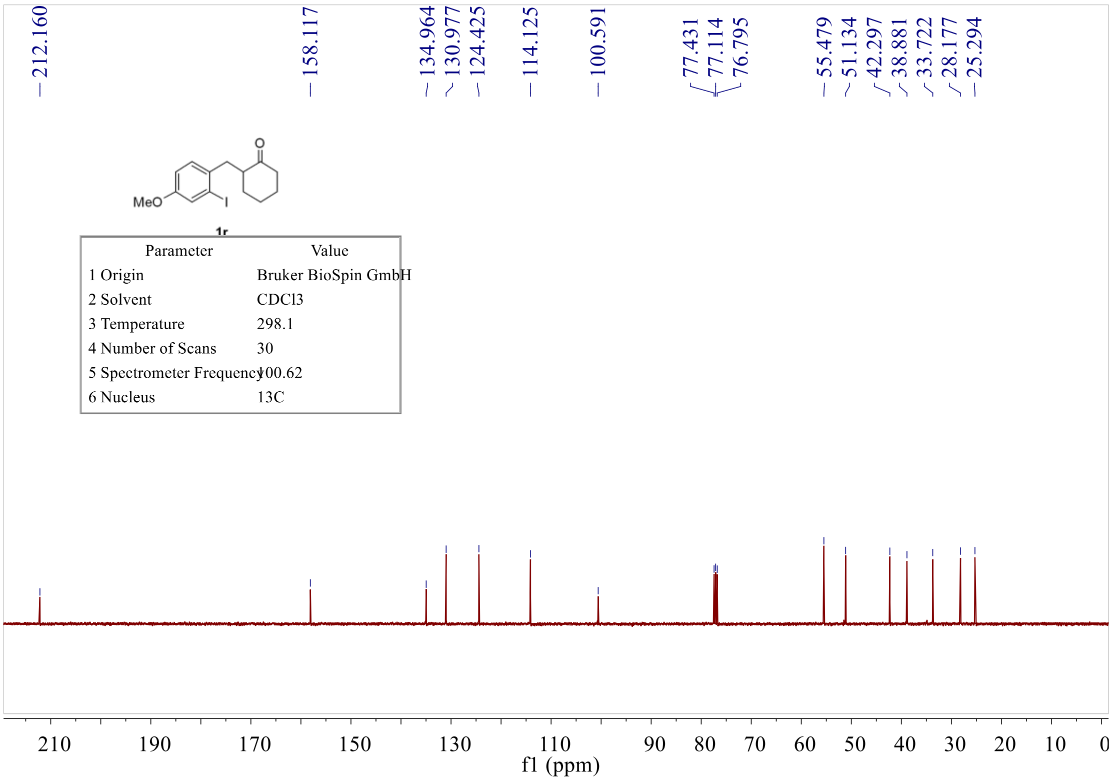

Supplementary Figure 51. ^{13}C **NMR (100 MHz, $CDCl_3$) spectra for compound 1r**

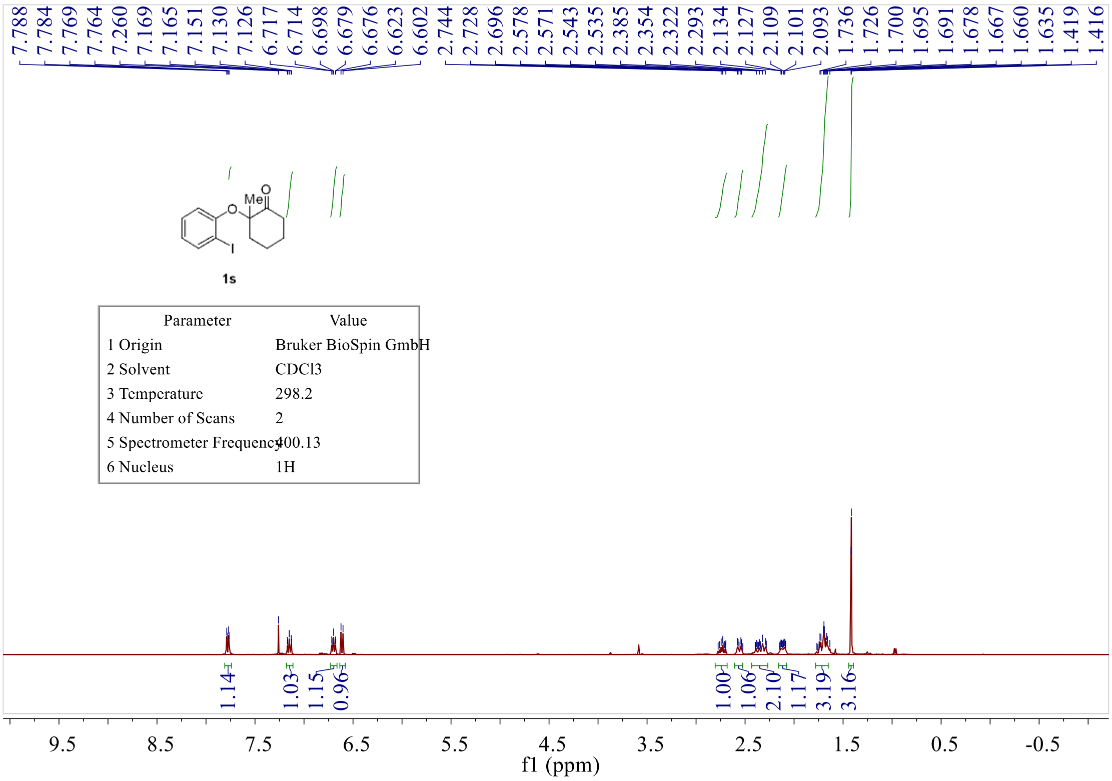

Supplementary Figure 52. ^{1}H NMR (400 MHz, $CDCl_3$) spectra for compound 1s

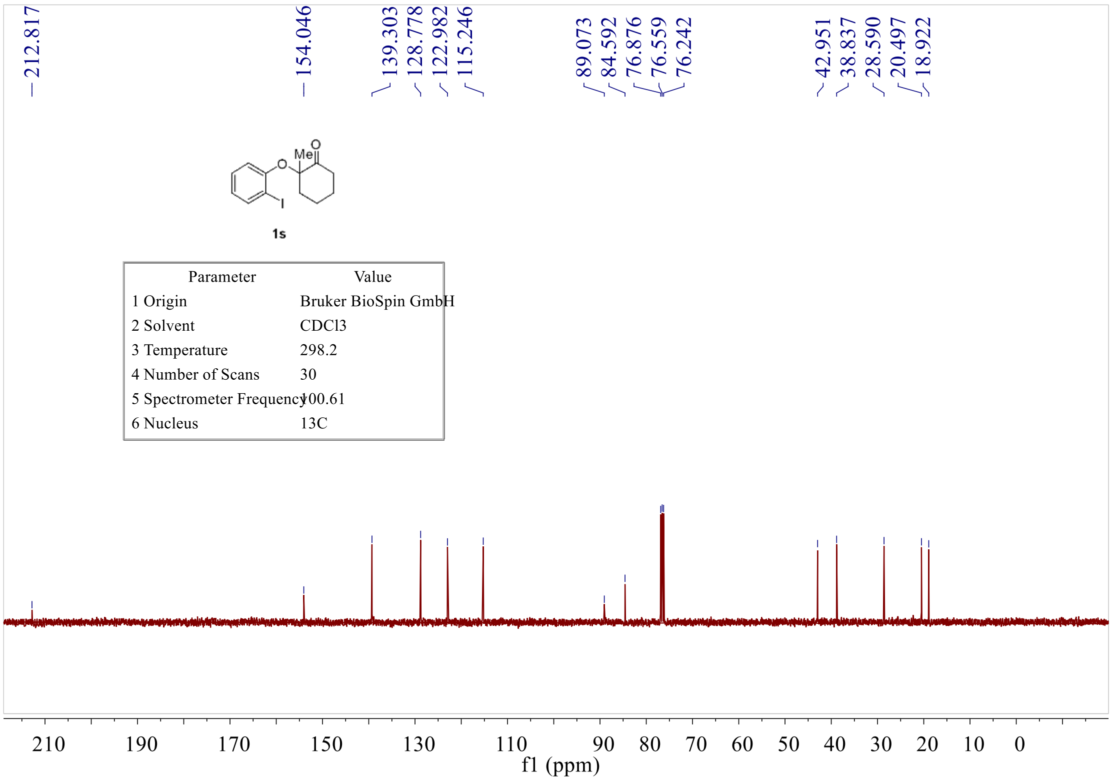

Supplementary Figure 53. ^{13}C NMR (100 MHz, $CDCl_3$) spectra for compound 1s

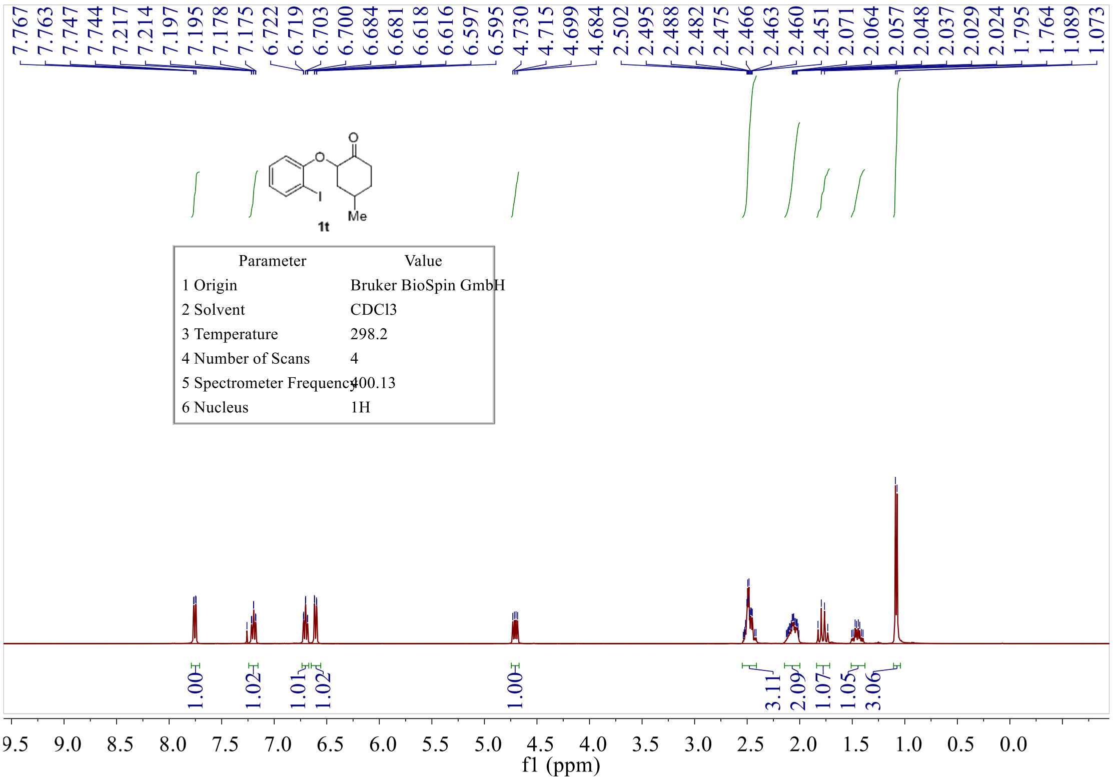

Supplementary Figure 54. ^{1}**H NMR (400 MHz, CDCl**$_{3}$**) spectra for compound 1t**

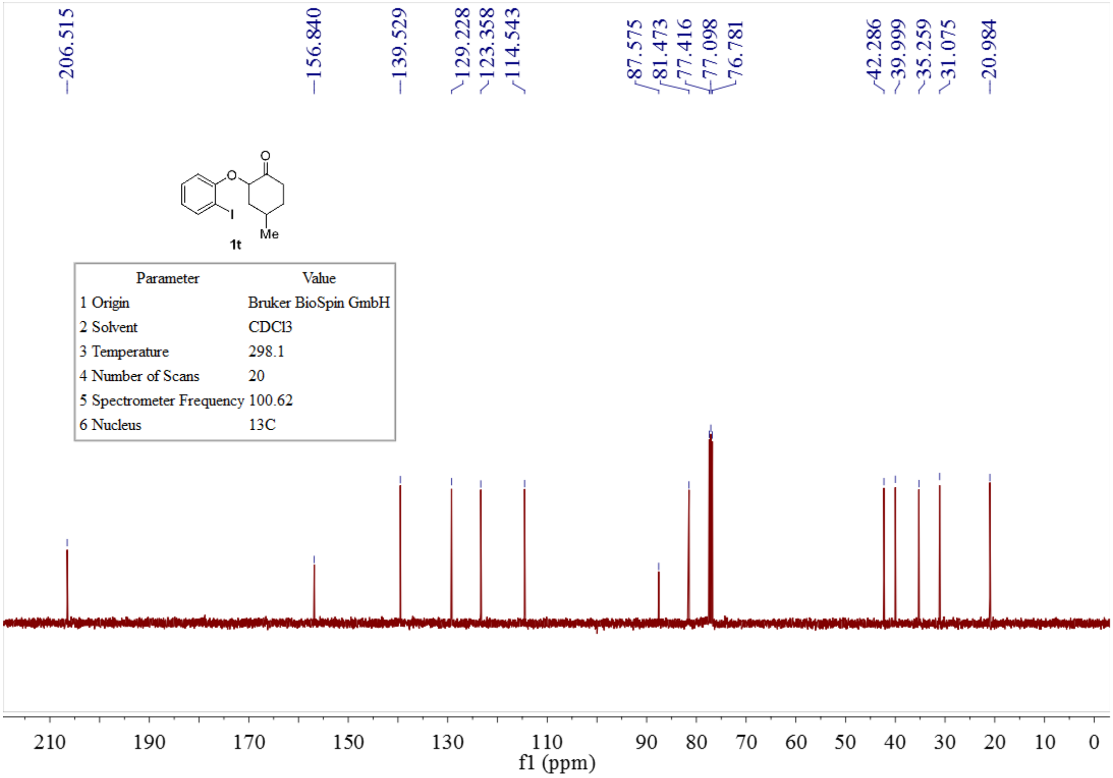

Supplementary Figure 55. ^{13}**C NMR (100 MHz, CDCl**$_{3}$**) spectra for compound 1t**

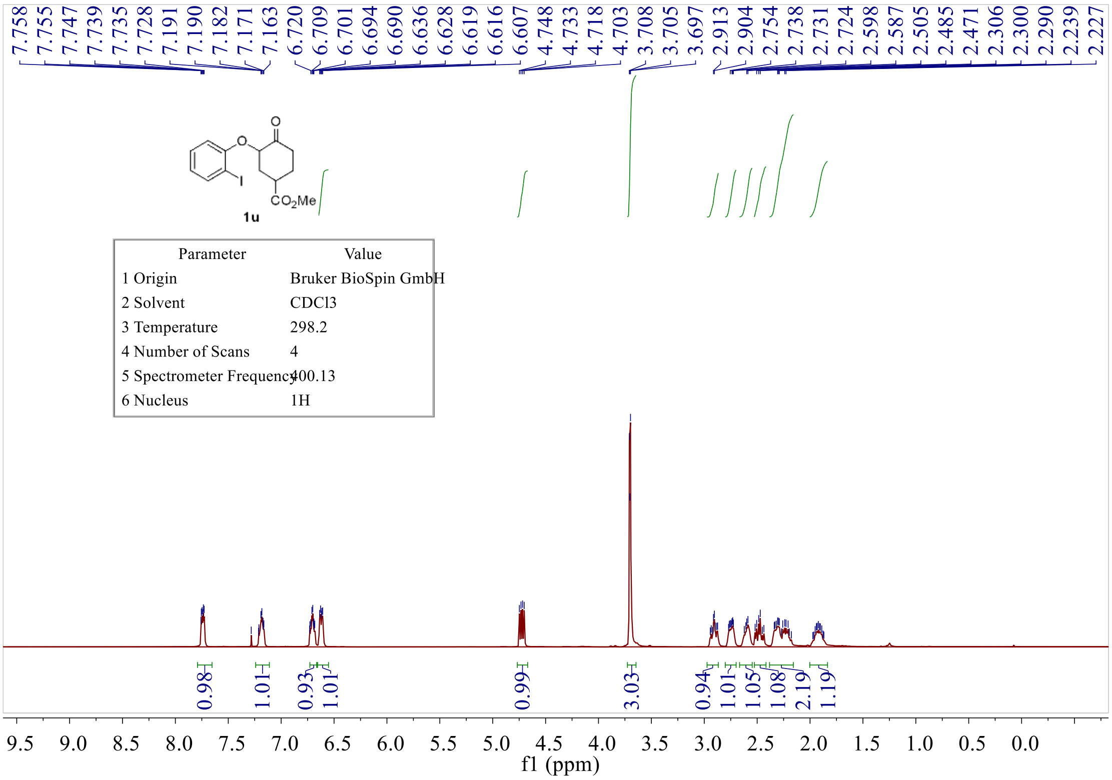

Supplementary Figure 56. ^{1}**H NMR (400 MHz,** $CDCl_3$**) spectra for compound 1u**

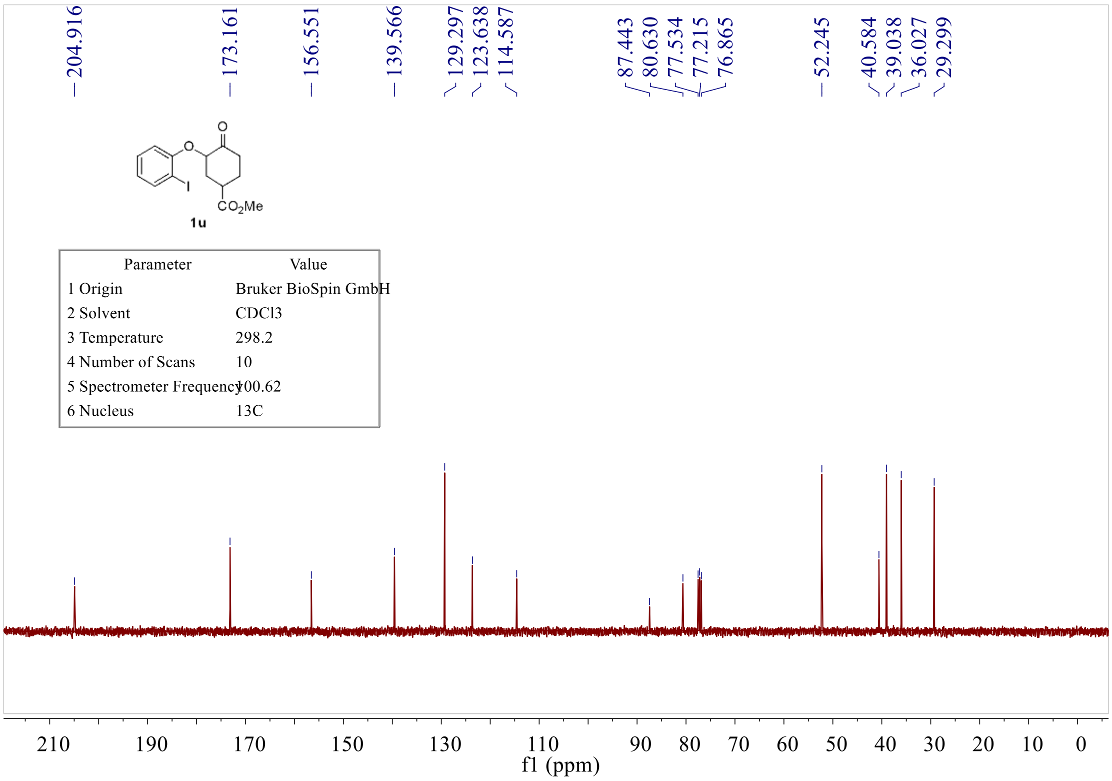

Supplementary Figure 57. ^{13}**C NMR (100 MHz,** $CDCl_3$**) spectra for compound 1u**

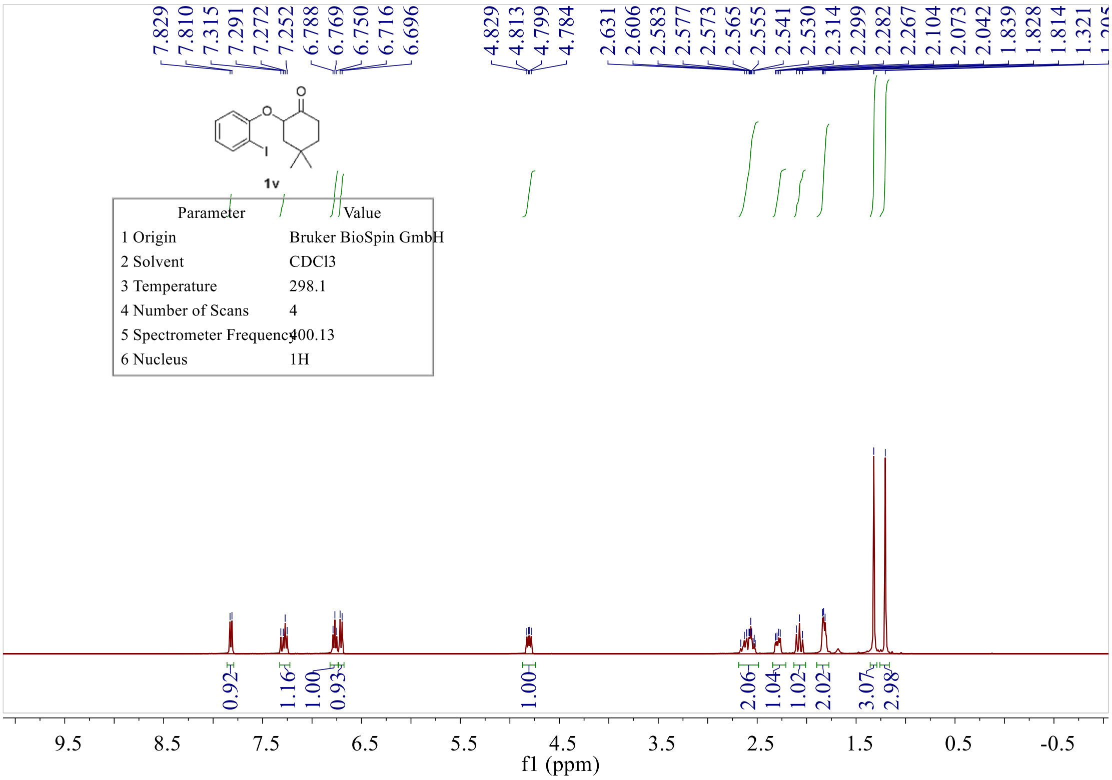

Supplementary Figure 58. ^{1}**H NMR (400 MHz, CDCl$_3$) spectra for compound 1v**

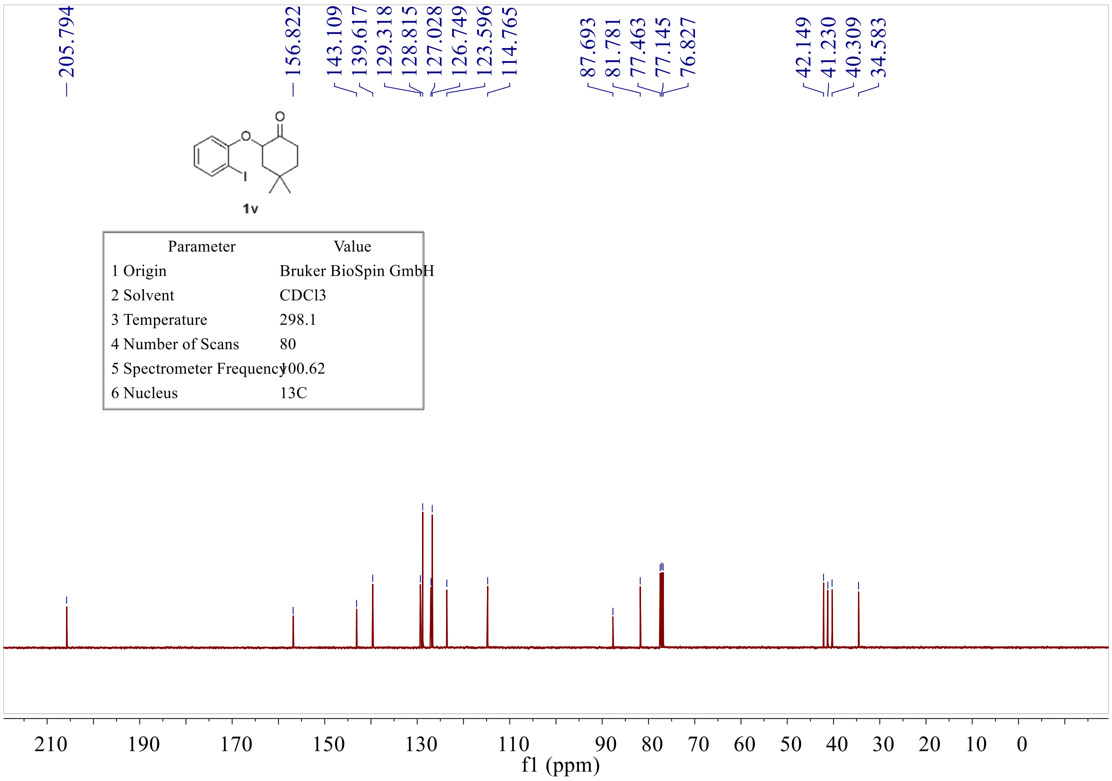

Supplementary Figure 59. ^{13}**C NMR (100 MHz, CDCl$_3$) spectra for compound 1v**

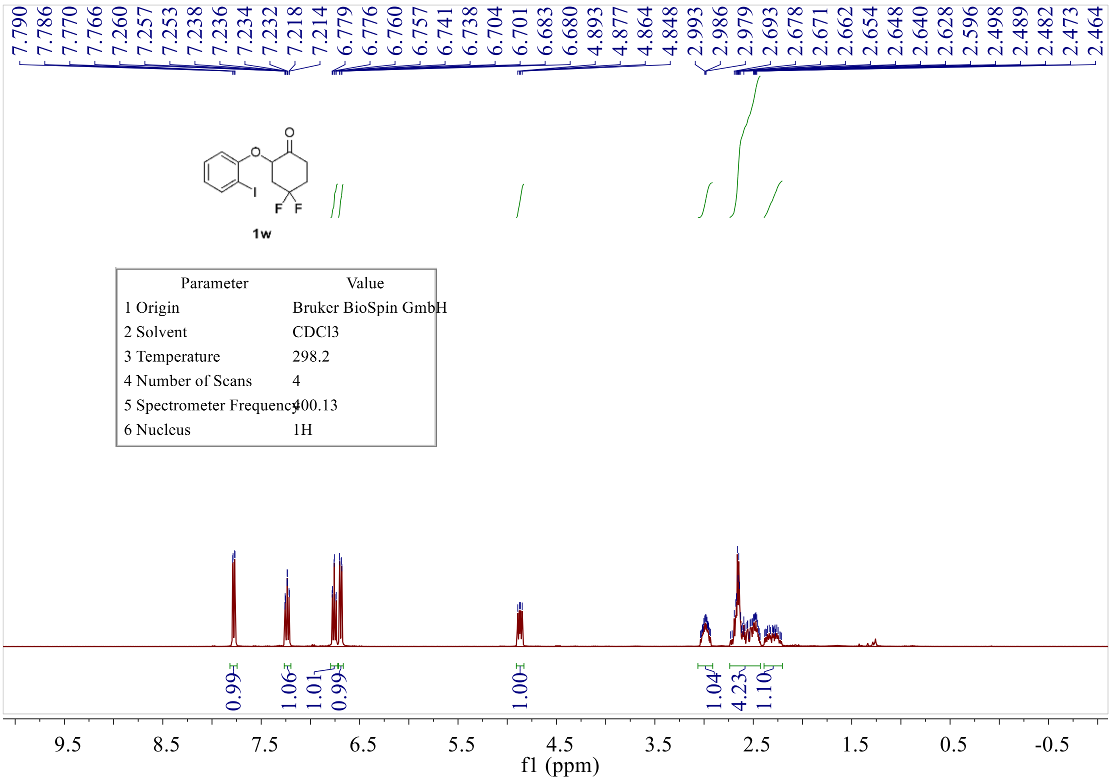

Supplementary Figure 60. ^{1}**H NMR (400 MHz, CDCl$_3$) spectra for compound 1w**

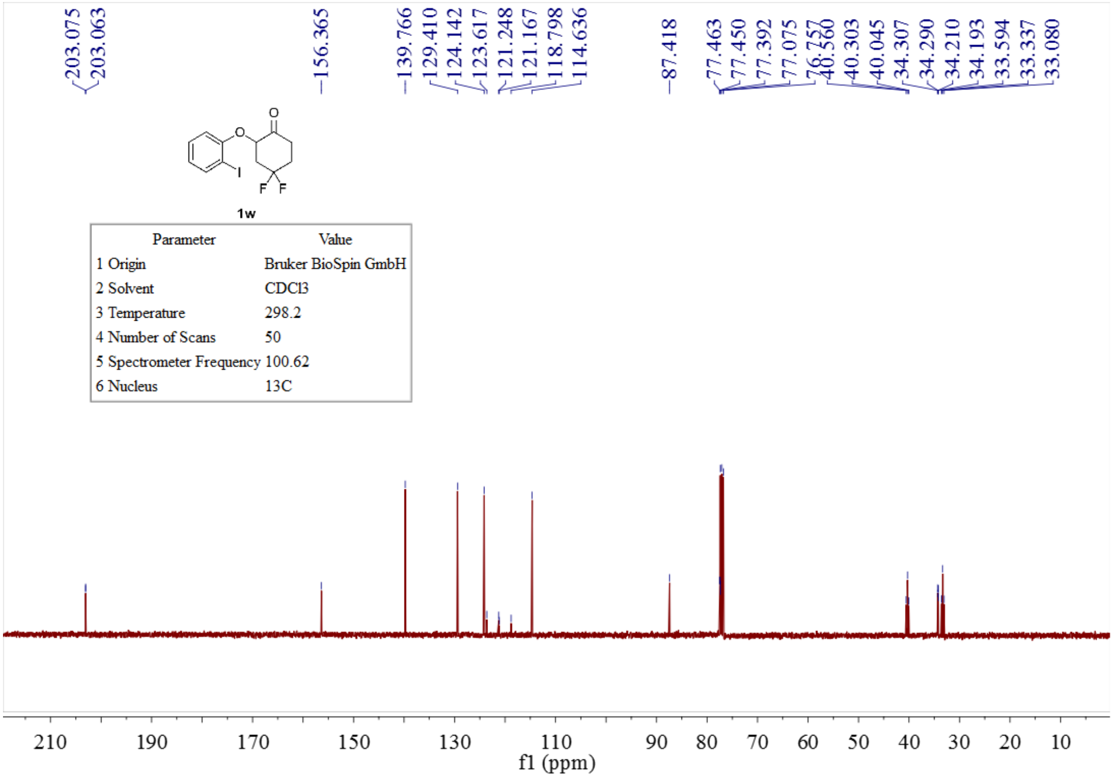

Supplementary Figure 61. ^{13}**C NMR (100 MHz, CDCl$_3$) spectra for compound 1w**

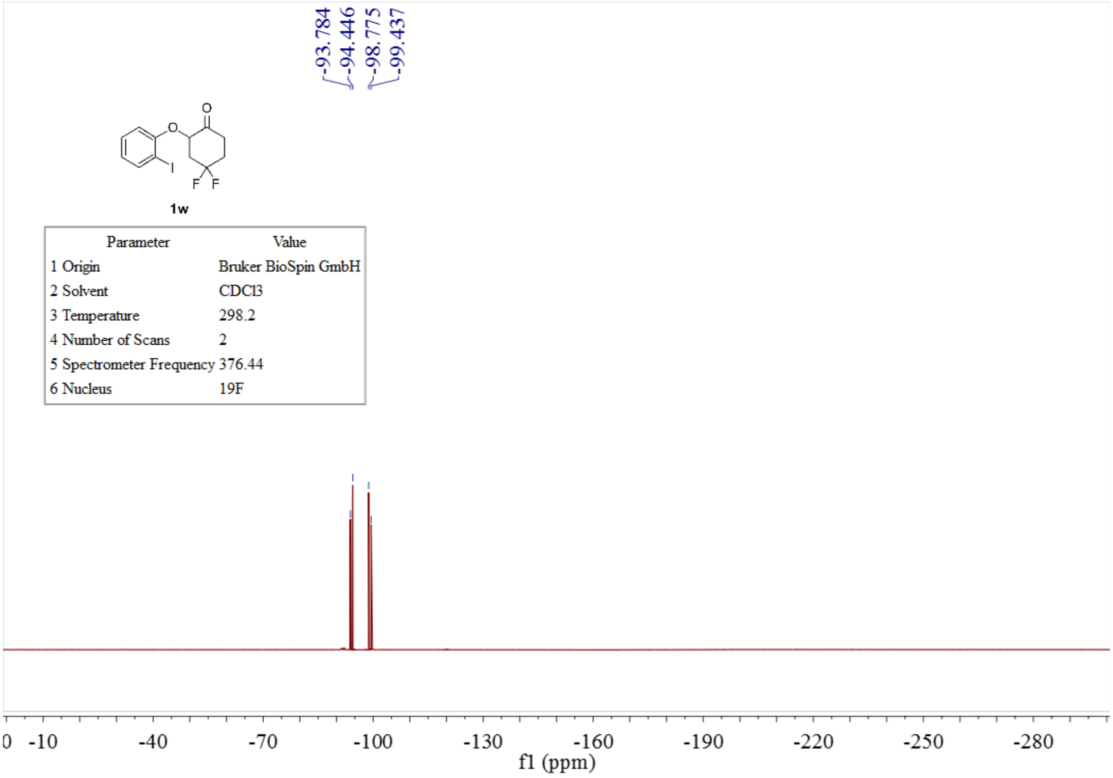

Supplementary Figure 62. ^{19}**F NMR (376 MHz, CDCl$_3$) spectra for compound 1w**

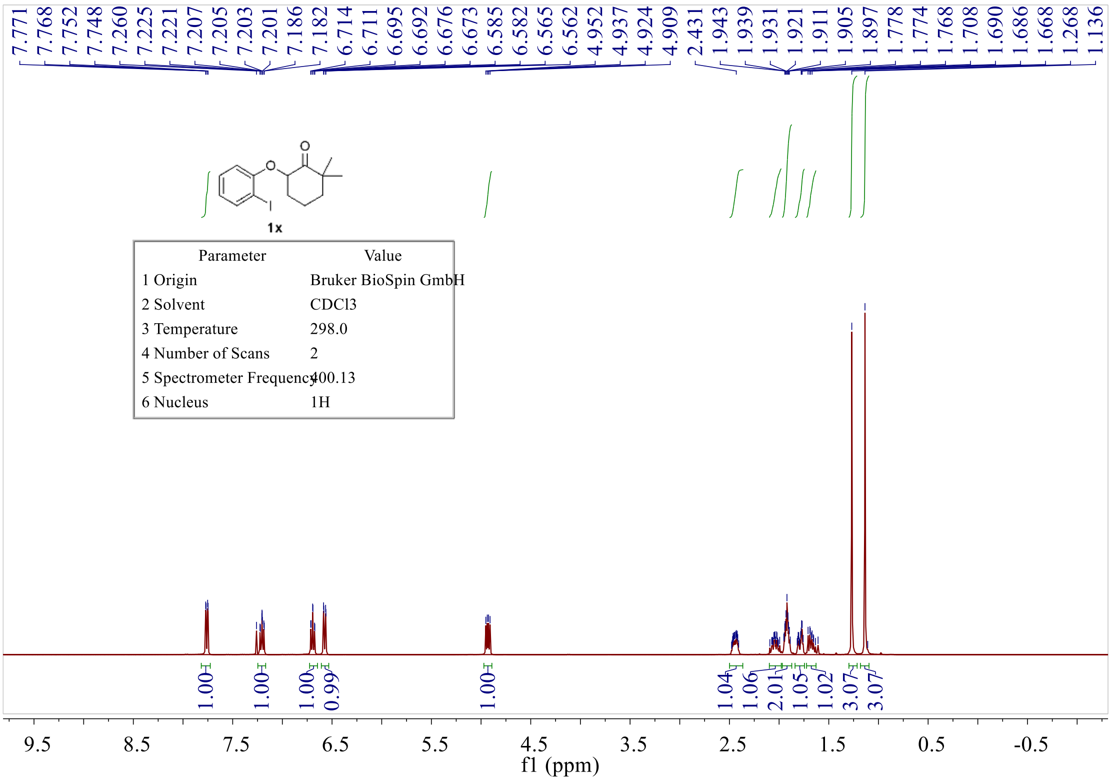

Supplementary Figure 63. ^{1}**H NMR (400 MHz, CDCl$_3$) spectra for compound 1x**

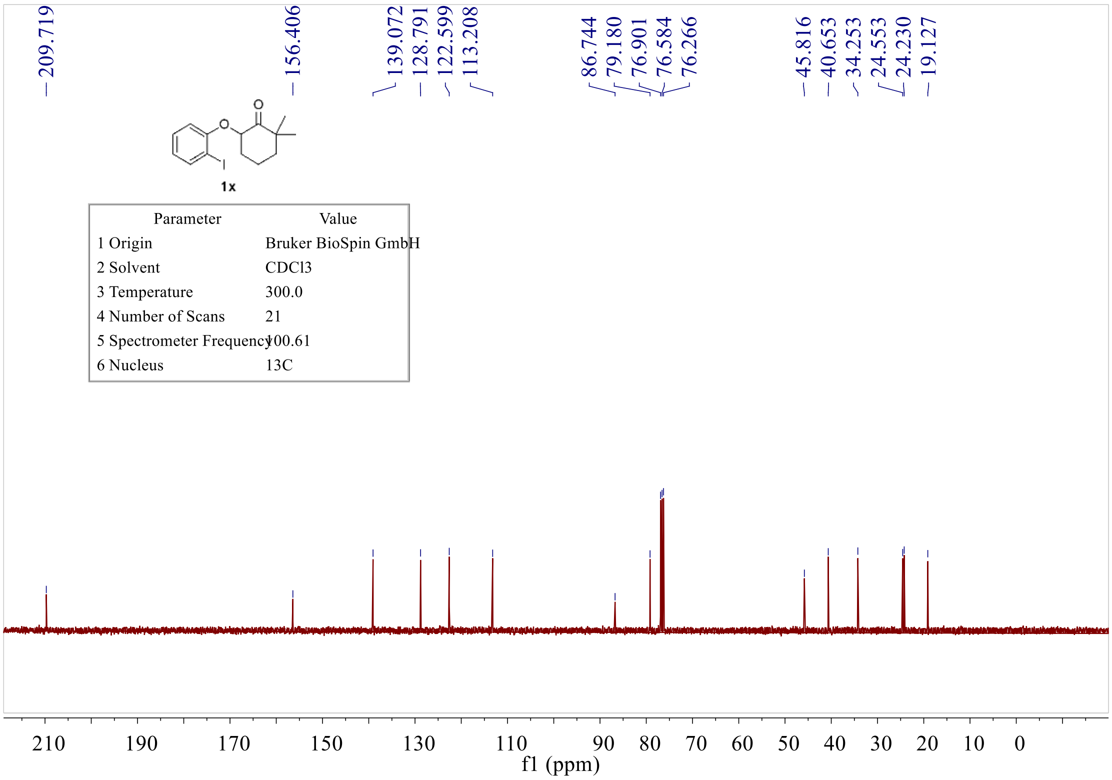

Supplementary Figure 64. ^{13}**C NMR (100 MHz, CDCl**$_3$**) spectra for compound 1x**

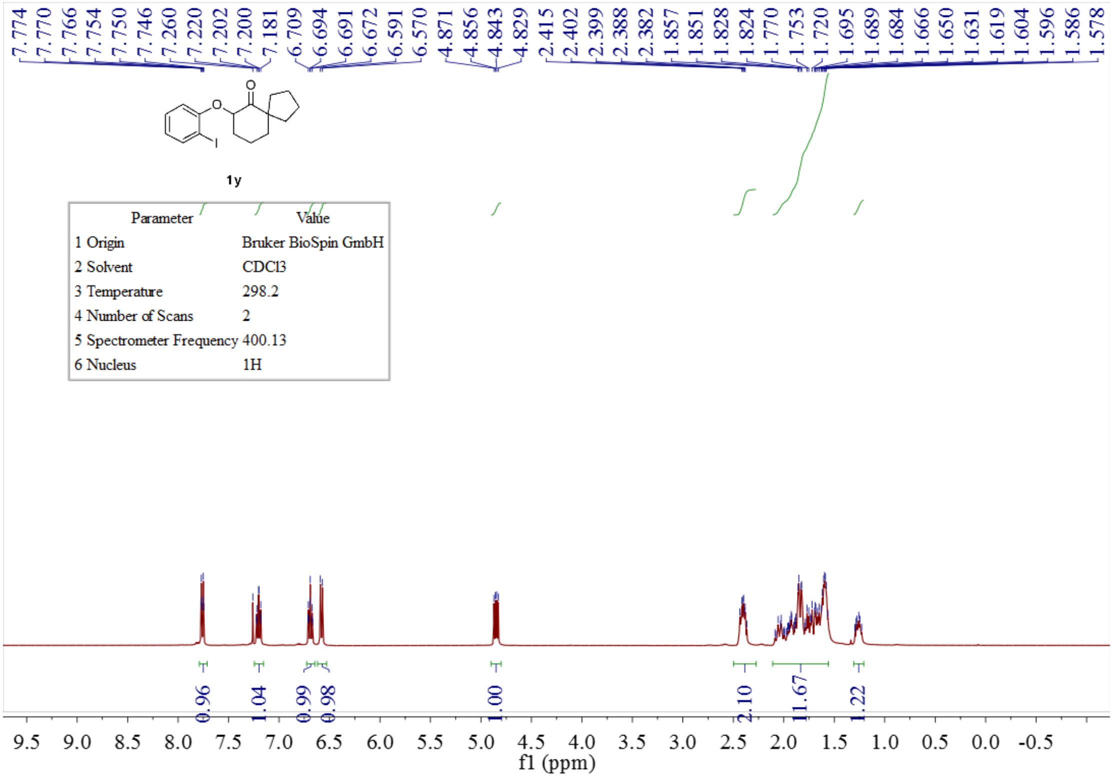

Supplementary Figure 65. ^{1}**H NMR (400 MHz, CDCl**$_3$**) spectra for compound 1y**

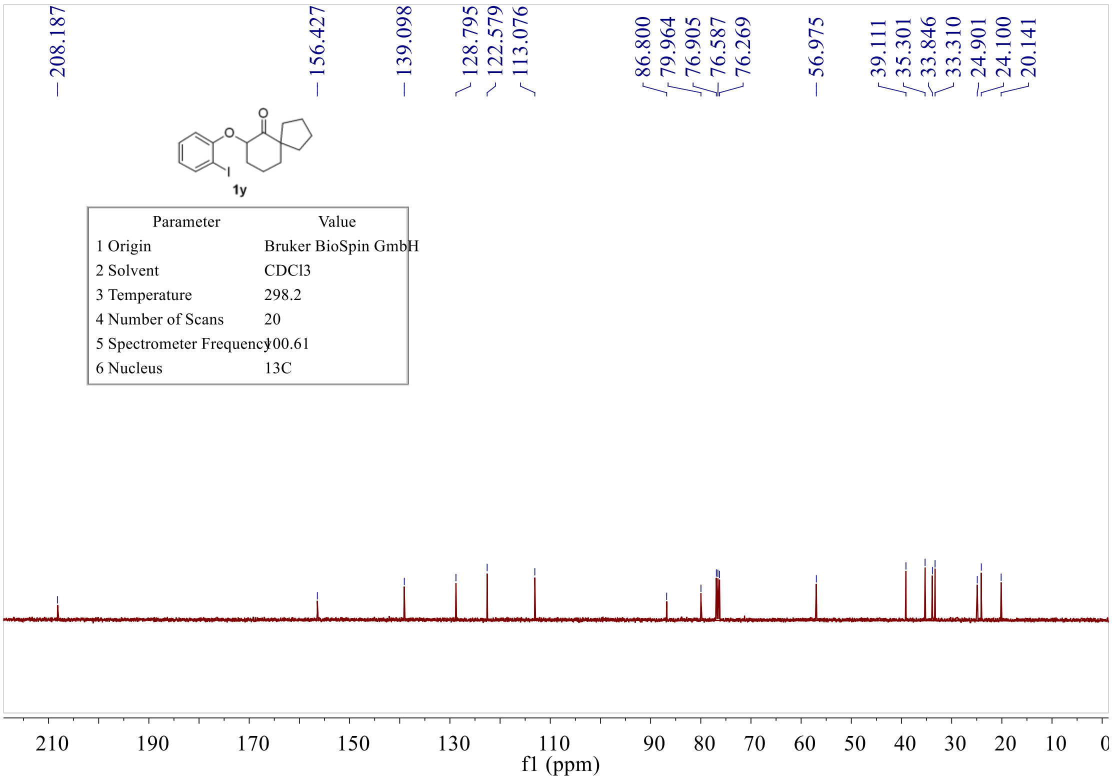

Supplementary Figure 66. ^{13}**C NMR (100 MHz, CDCl**$_3$**) spectra for compound 1y**

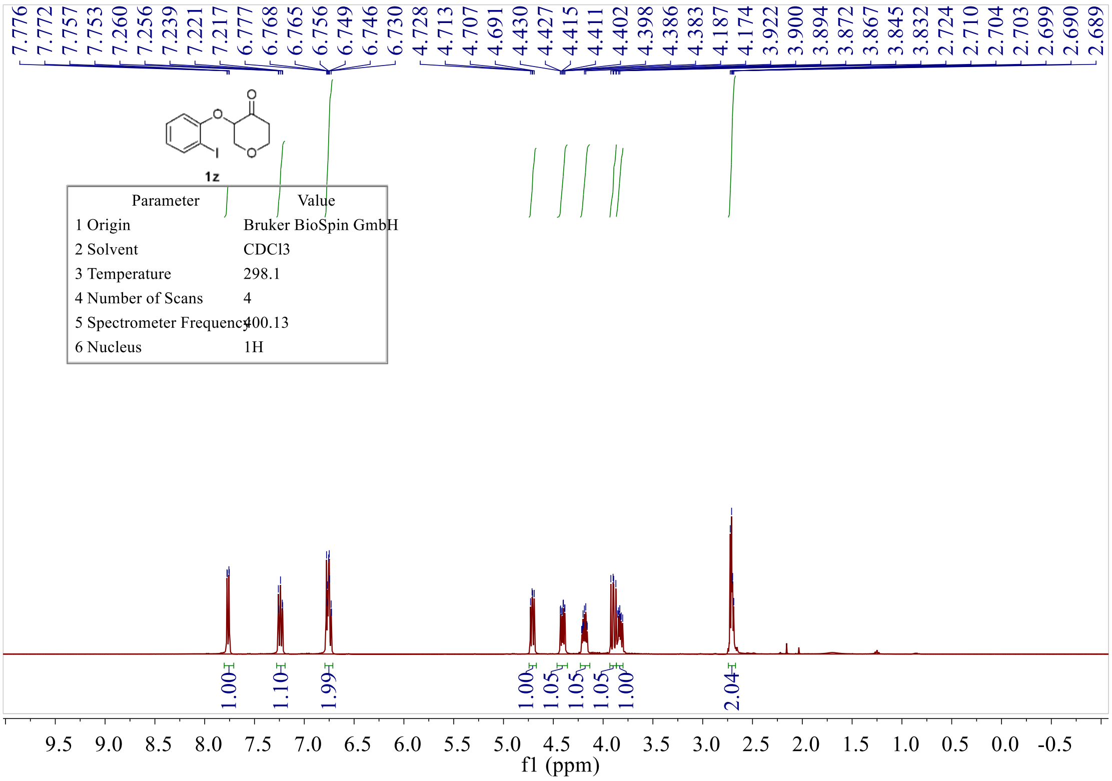

Supplementary Figure 67. ^{1}**H NMR (400 MHz, CDCl**$_3$**) spectra for compound 1z**

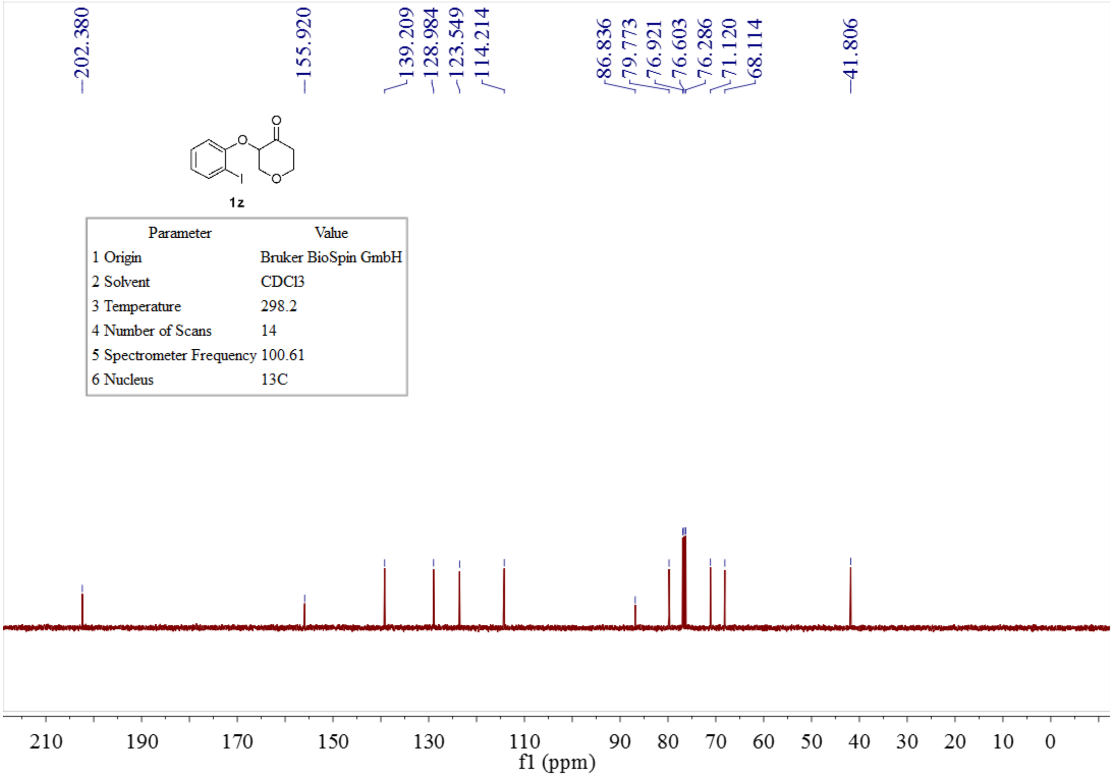

Supplementary Figure 68. **^{13}C NMR (100 MHz, $CDCl_3$) spectra for compound 1z**

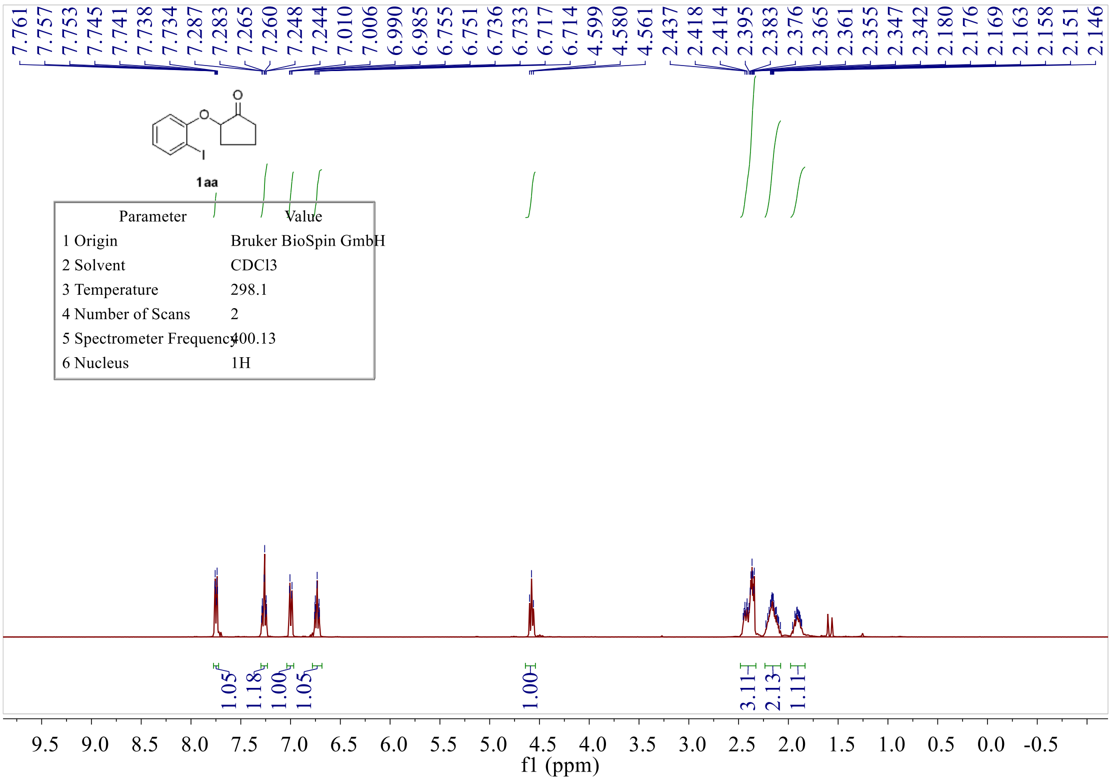

Supplementary Figure 69. **^{1}H NMR (400 MHz, $CDCl_3$) spectra for compound 1aa**

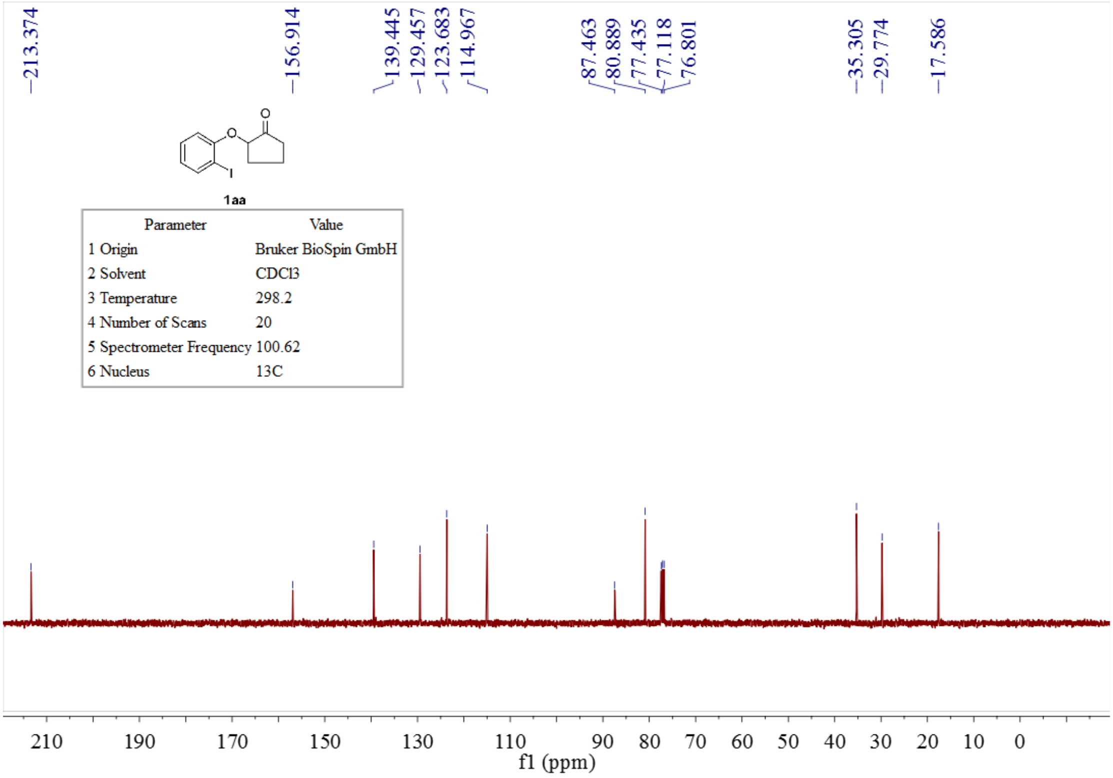

Supplementary Figure 70. ^{13}**C NMR (100 MHz, CDCl**$_3$**) spectra for compound 1aa**

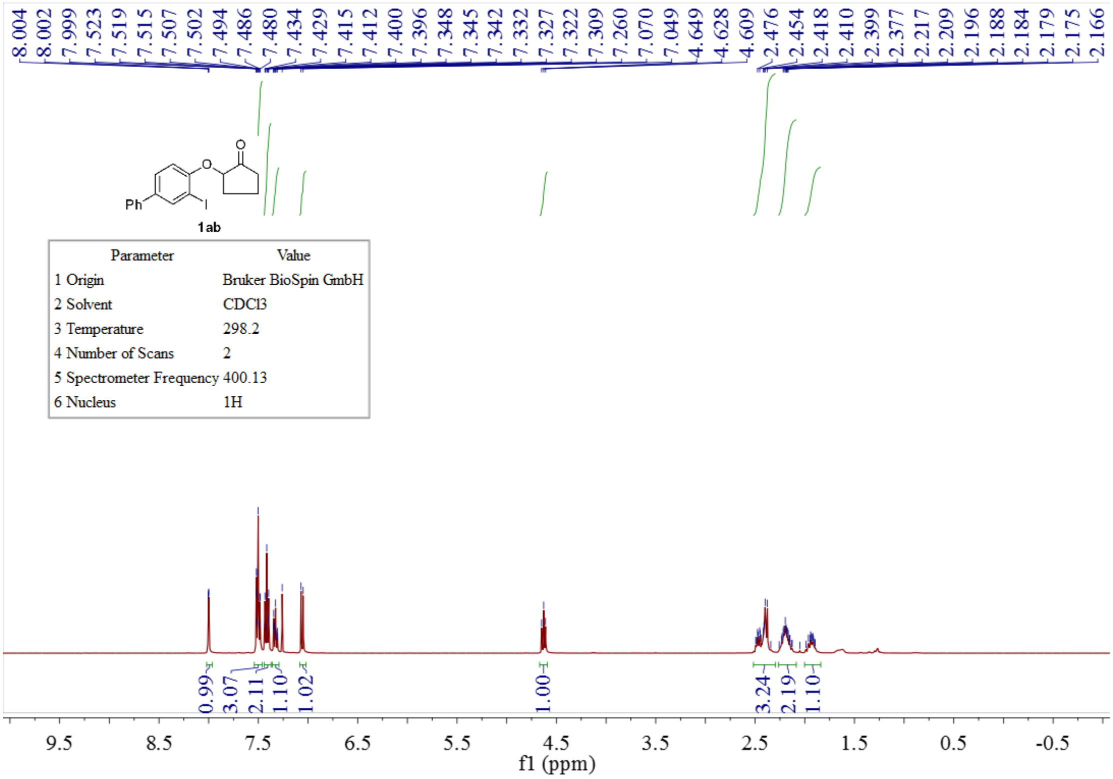

Supplementary Figure 71. ^{1}**H NMR (400 MHz, CDCl**$_3$**) spectra for compound 1ab**

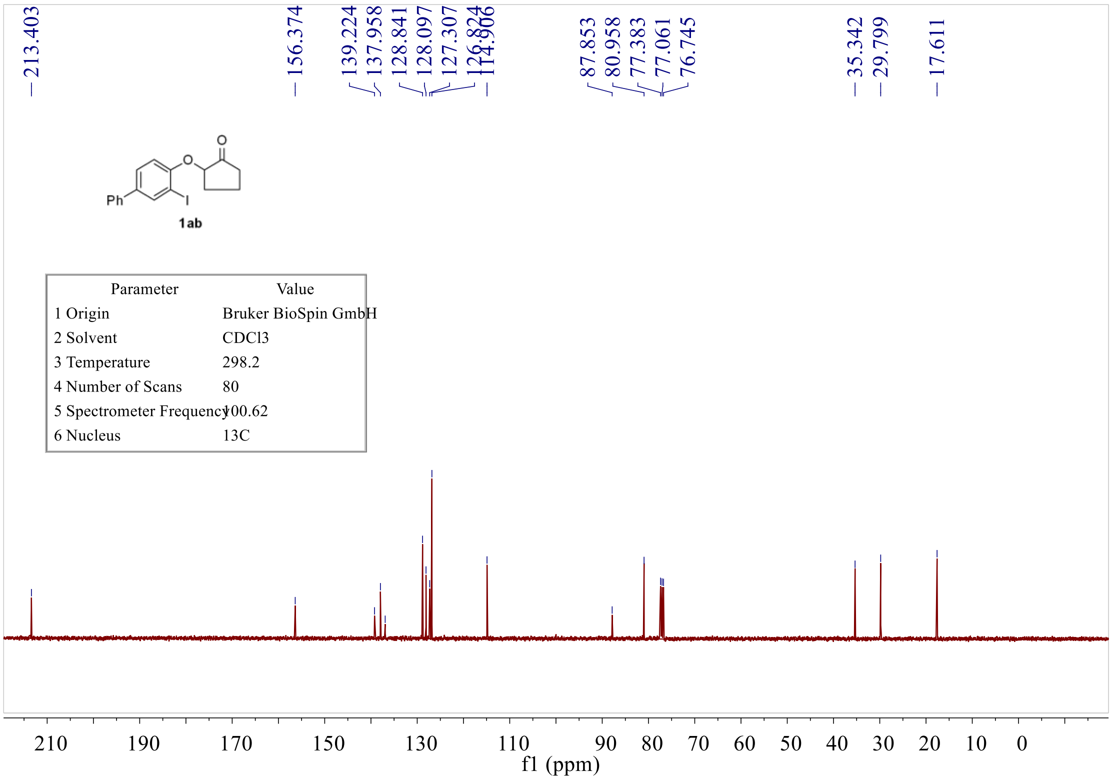

Supplementary Figure 72. ^{13}**C NMR (100 MHz, CDCl$_3$) spectra for compound 1ab**

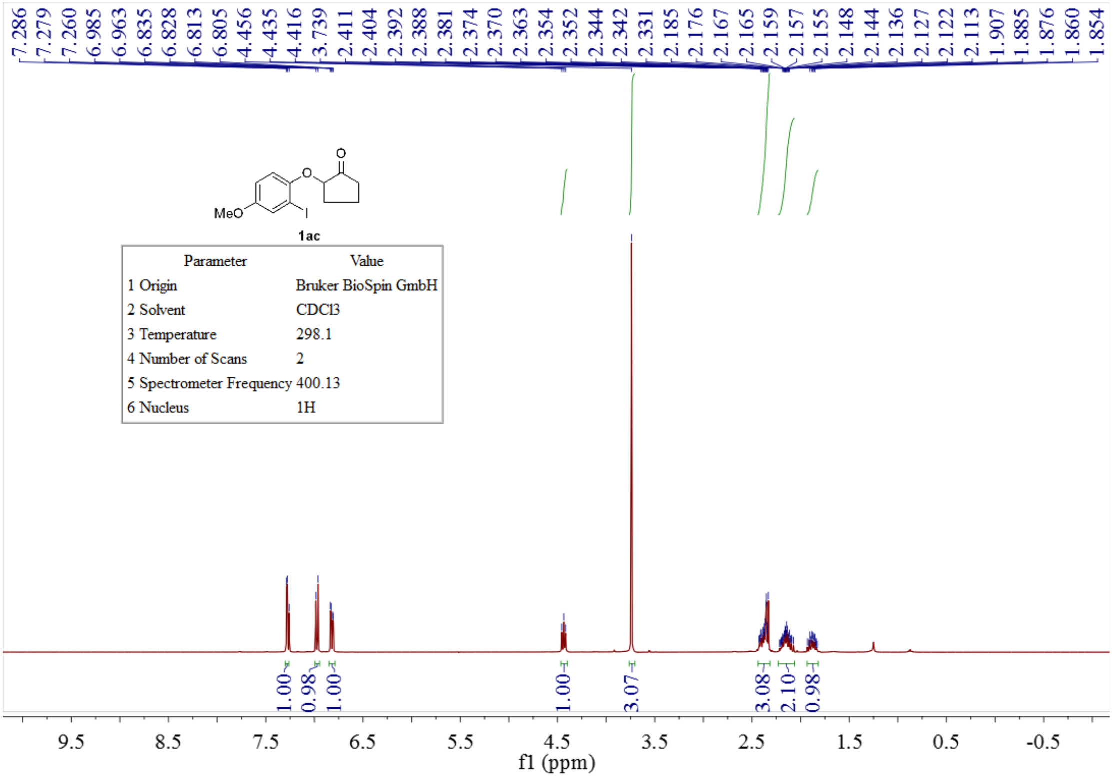

Supplementary Figure 73. ^{1}**H NMR (400 MHz, CDCl$_3$) spectra for compound 1ac**

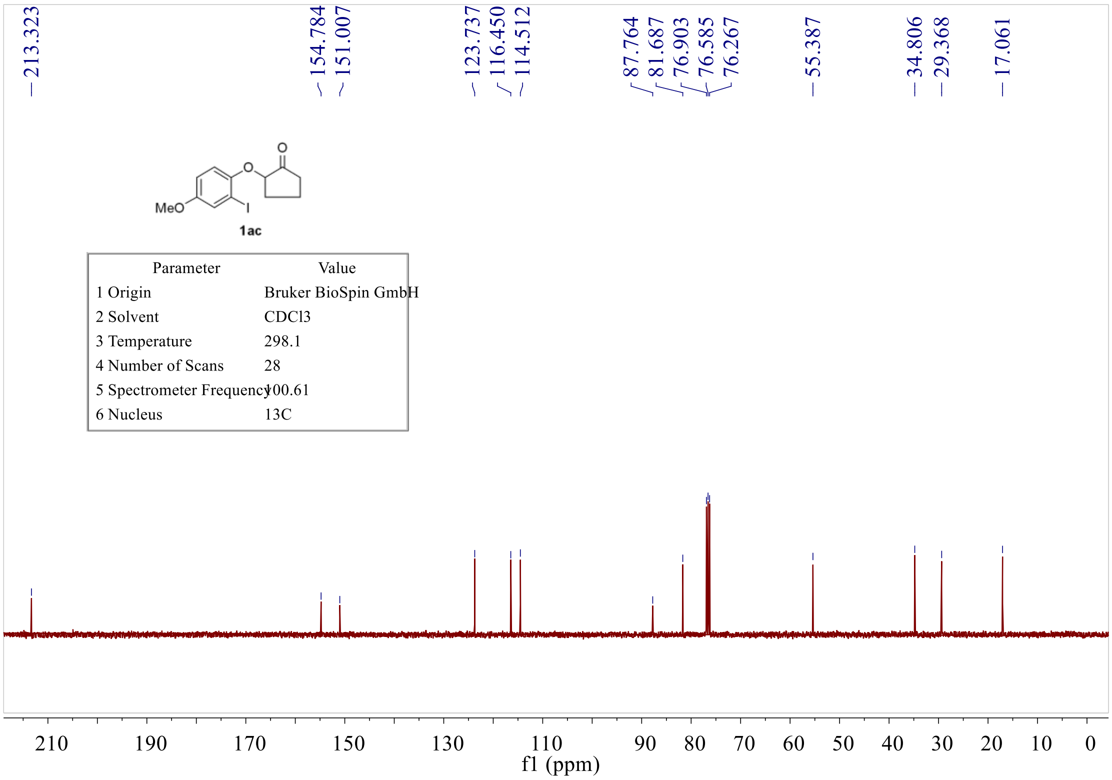

Supplementary Figure 74. ^{13}**C NMR (100 MHz, CDCl$_3$) spectra for compound 1ac**

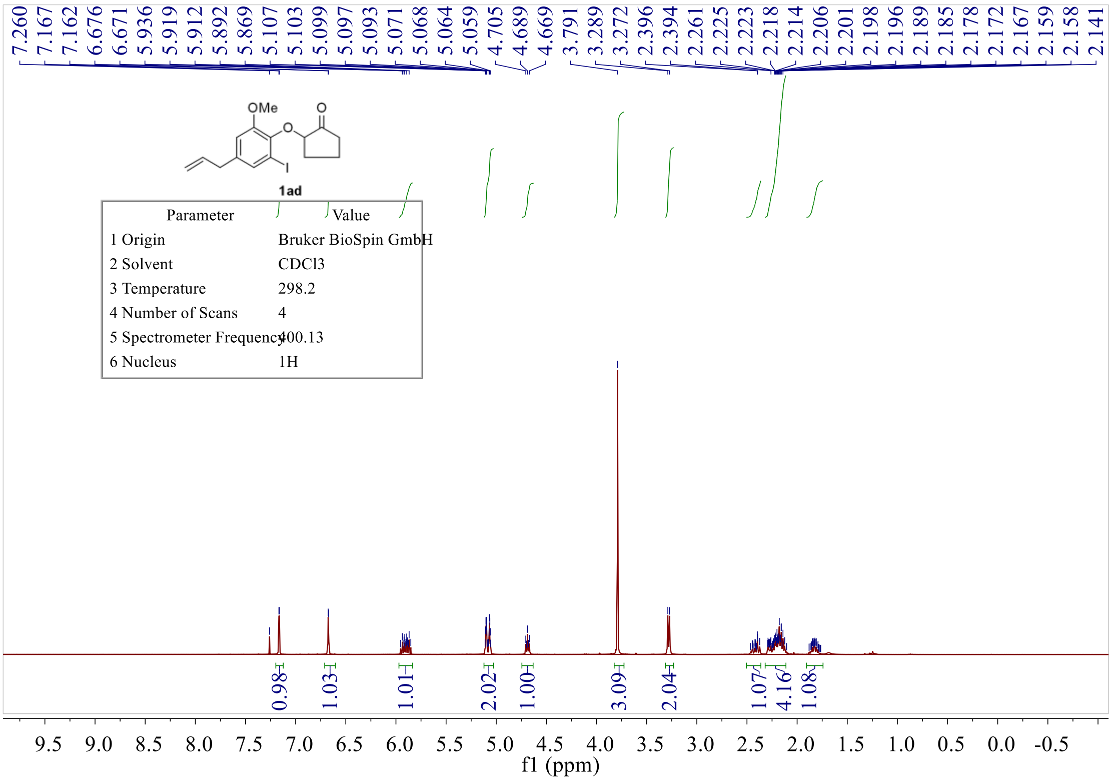

Supplementary Figure 75. ^{1}**H NMR (400 MHz, CDCl$_3$) spectra for compound 1ad**

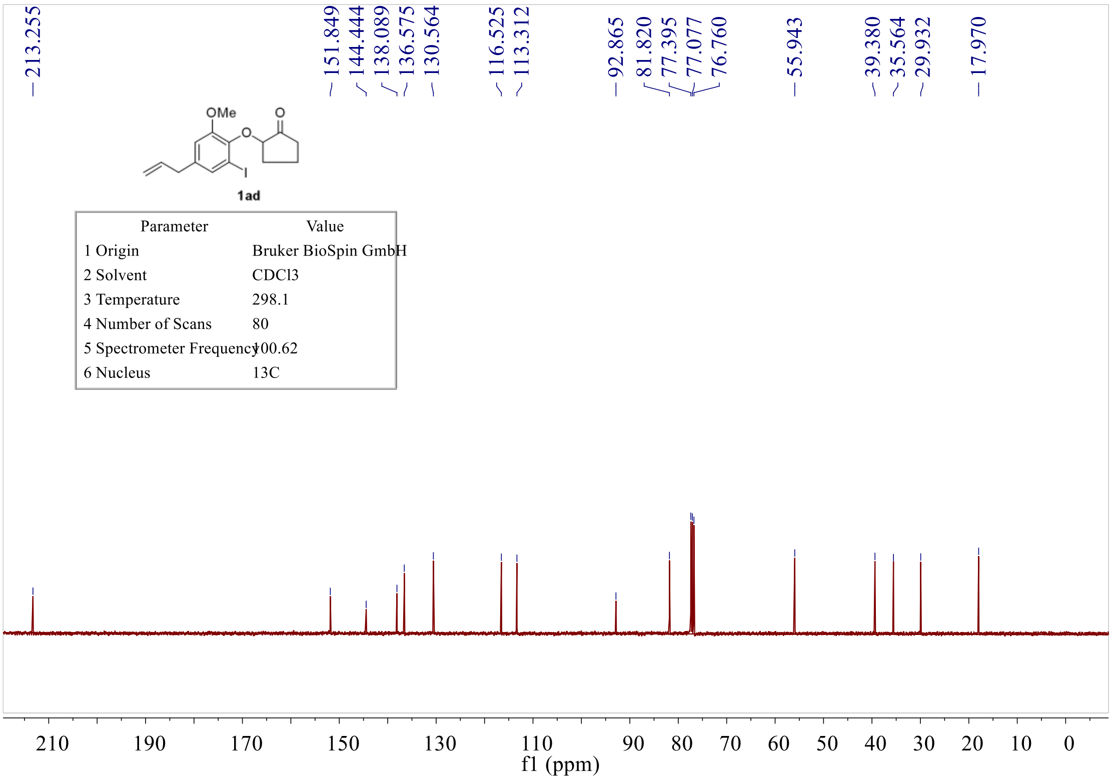

Supplementary Figure 76. ^{13}**C NMR (100 MHz,** $CDCl_3$**) spectra for compound 1ad**

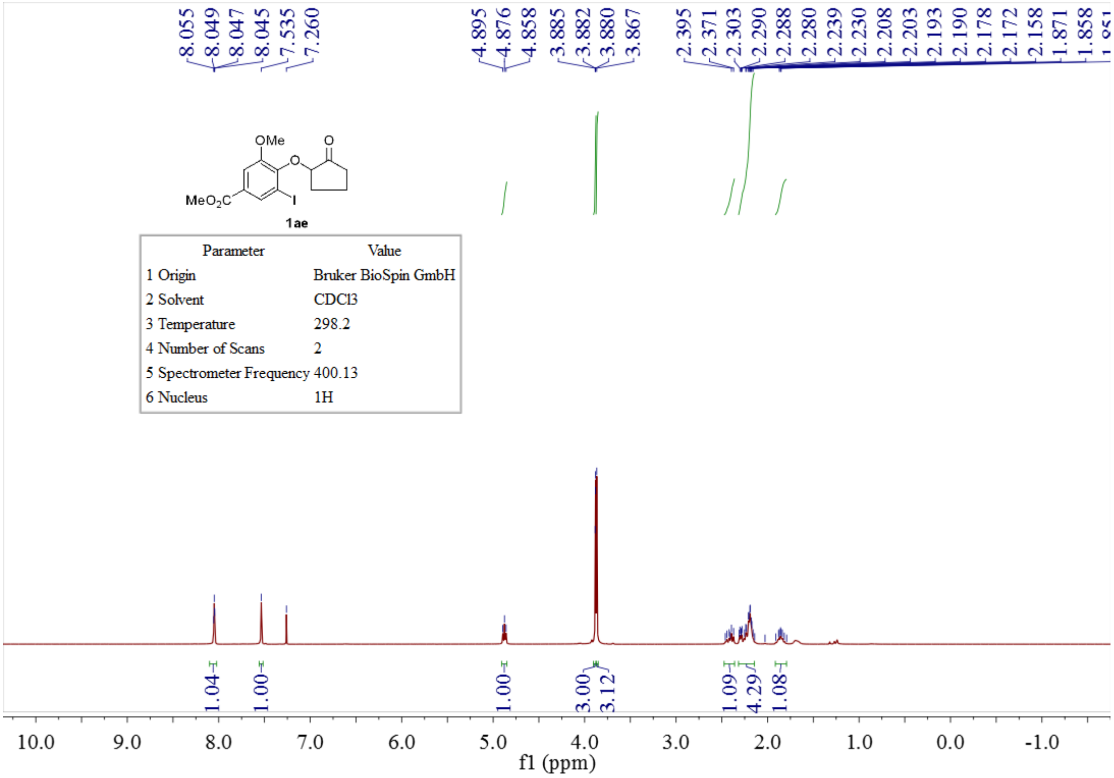

Supplementary Figure 77. ^{1}**H NMR (400 MHz,** $CDCl_3$**) spectra for compound 1ae**

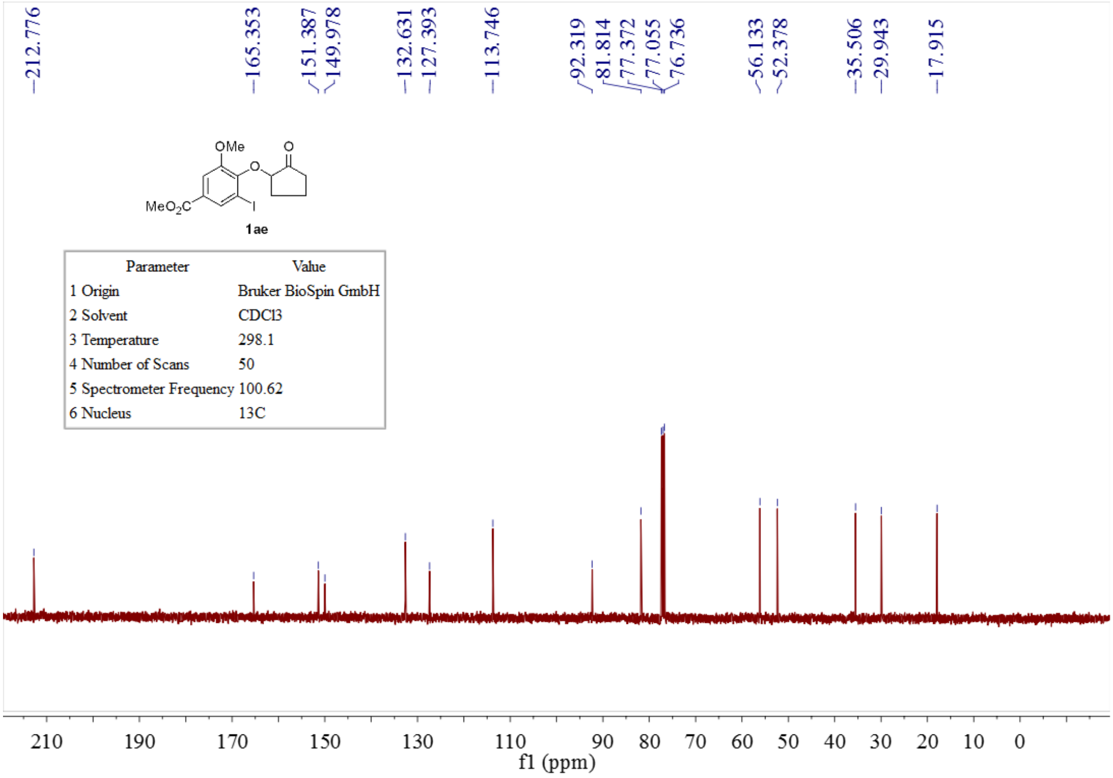

Supplementary Figure 78. ^{13}**C NMR (100 MHz, CDCl**$_3$**) spectra for compound 1ae**

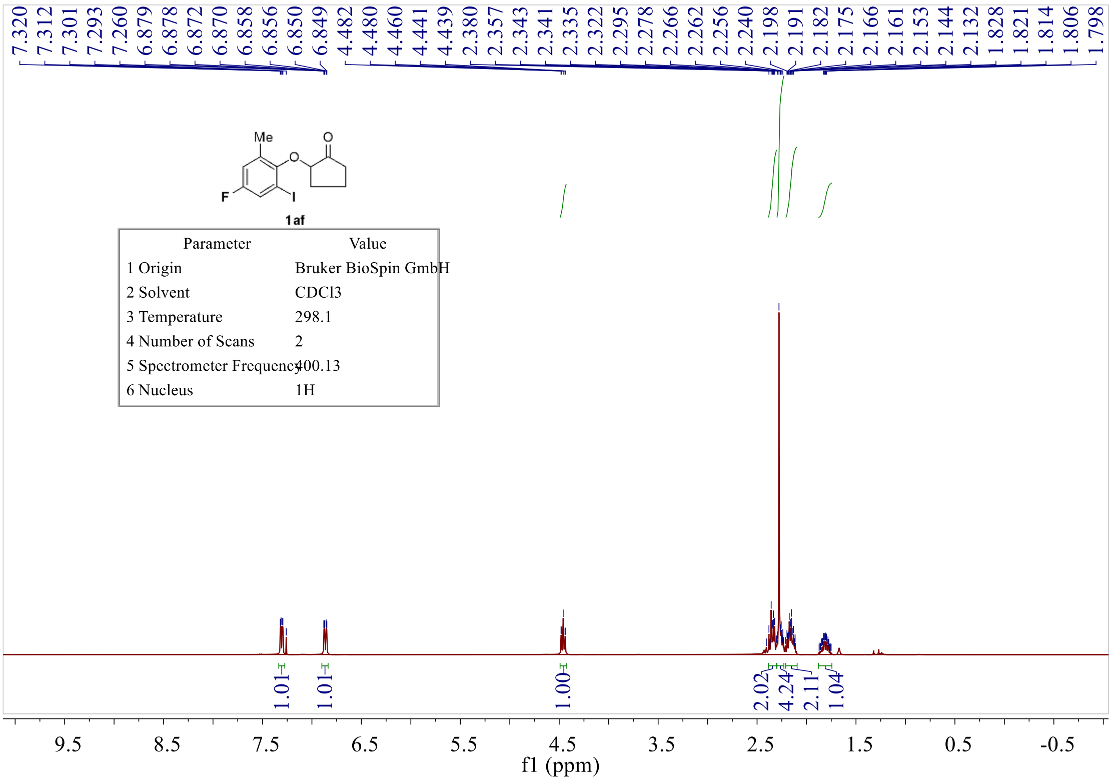

Supplementary Figure 79. ^{1}**H NMR (400 MHz, CDCl**$_3$**) spectra for compound 1af**

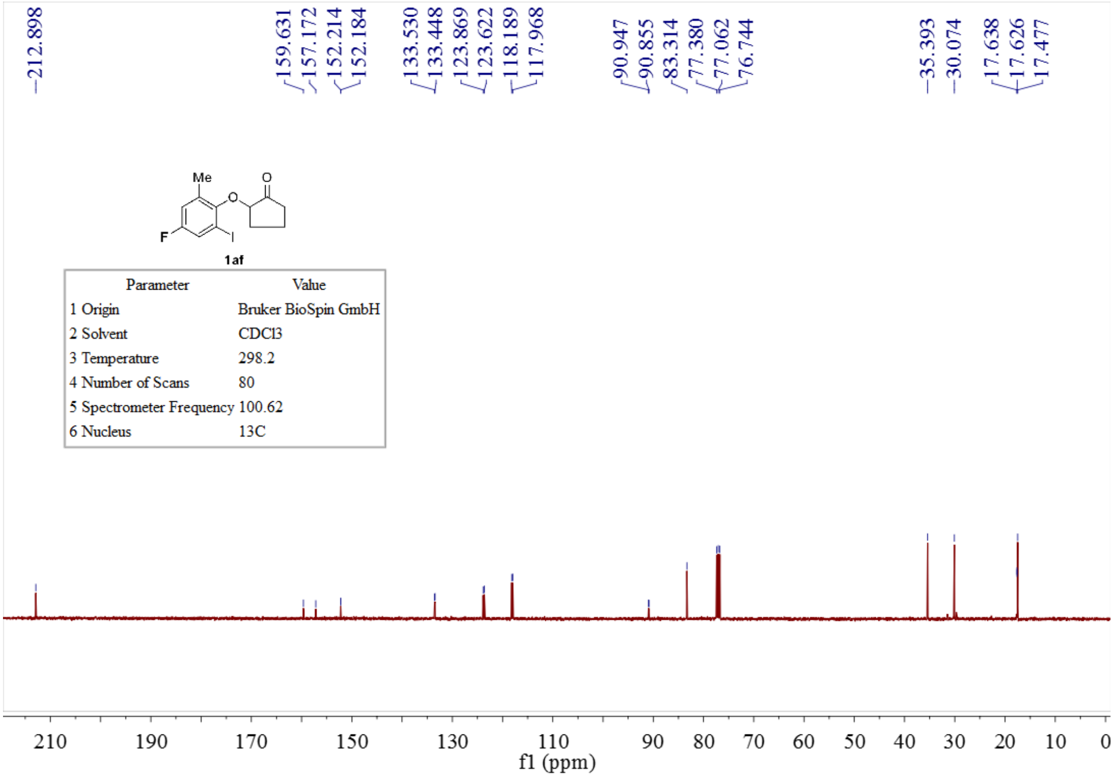

Supplementary Figure 80. ^{13}**C NMR (100 MHz, CDCl$_3$) spectra for compound 1af**

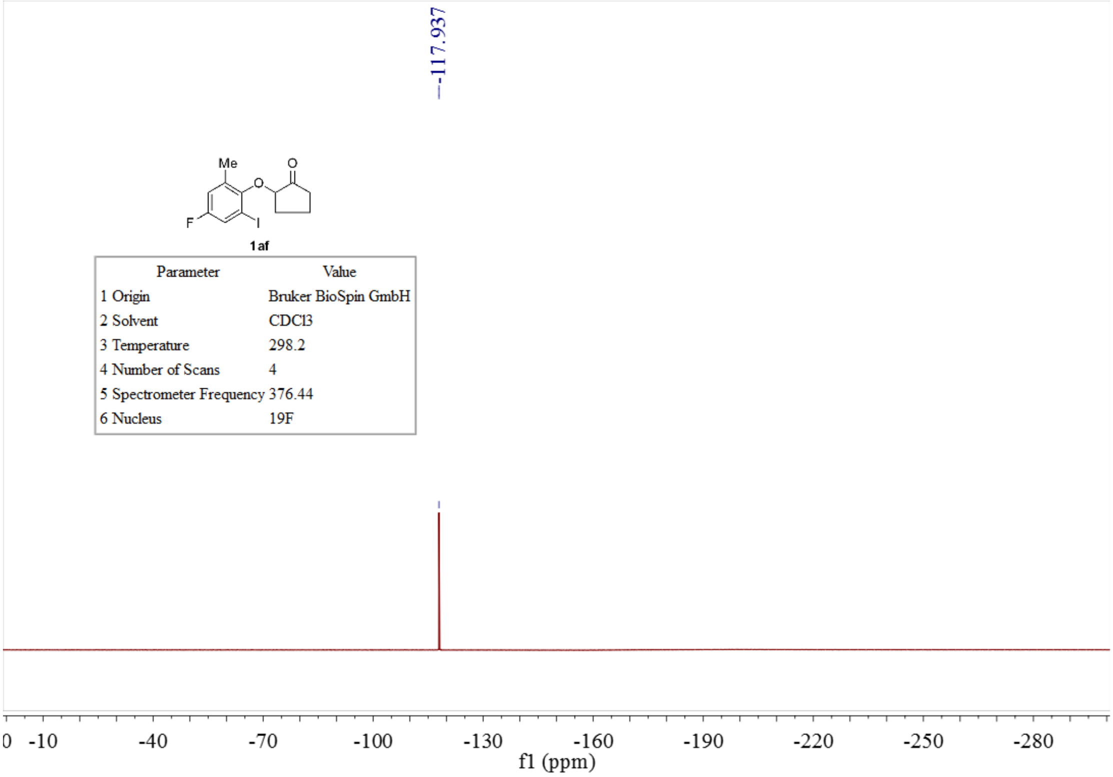

Supplementary Figure 81. ^{19}**F NMR (376 MHz, CDCl$_3$) spectra for compound 1af**

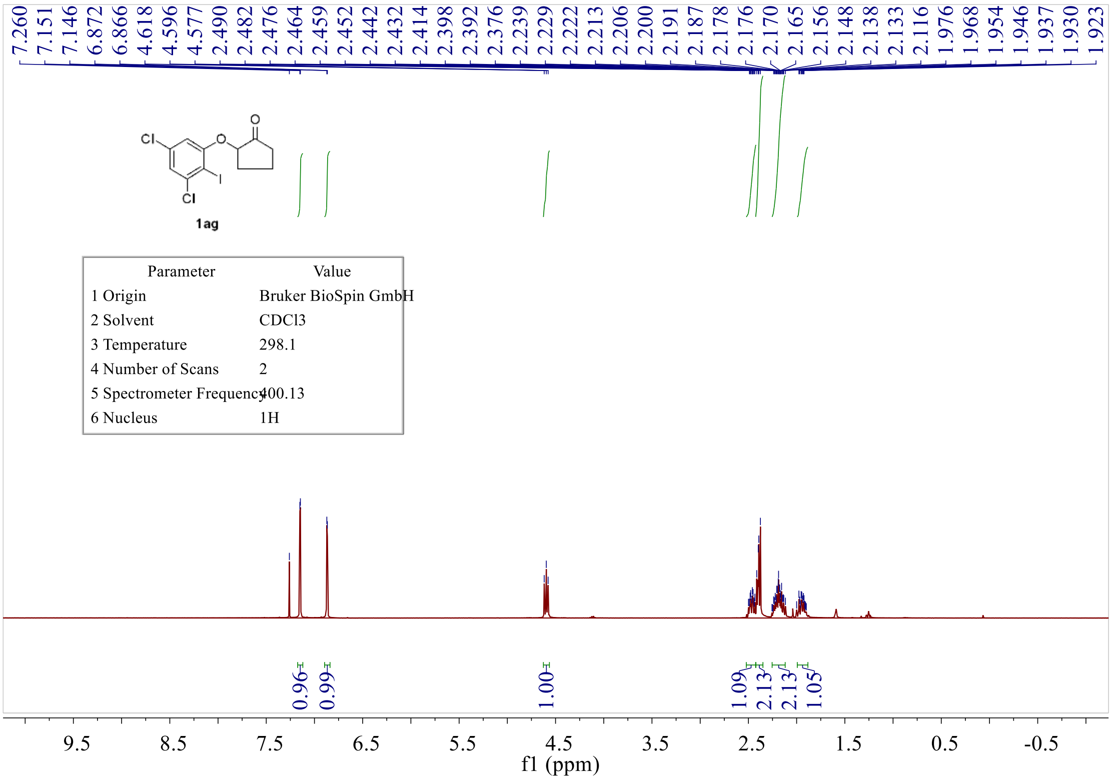

Supplementary Figure 82. ^{1}H NMR (400 MHz, $CDCl_3$) spectra for compound 1ag

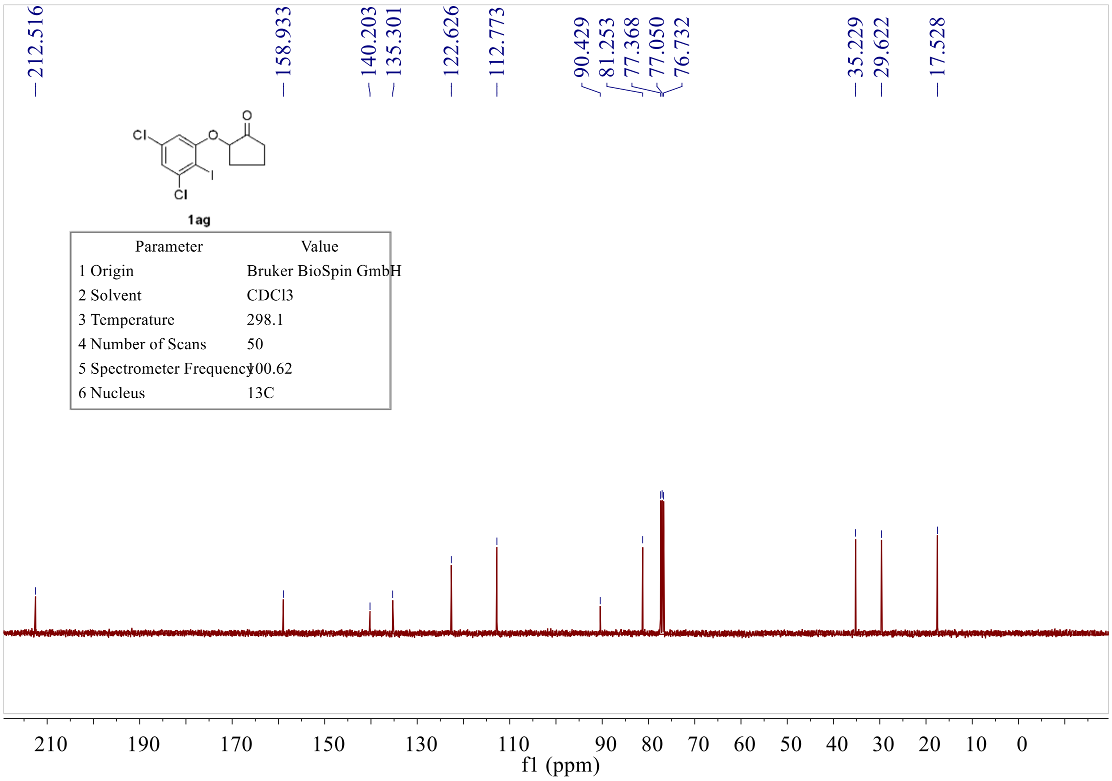

Supplementary Figure 83. ^{13}C NMR (100 MHz, $CDCl_3$) spectra for compound 1ag

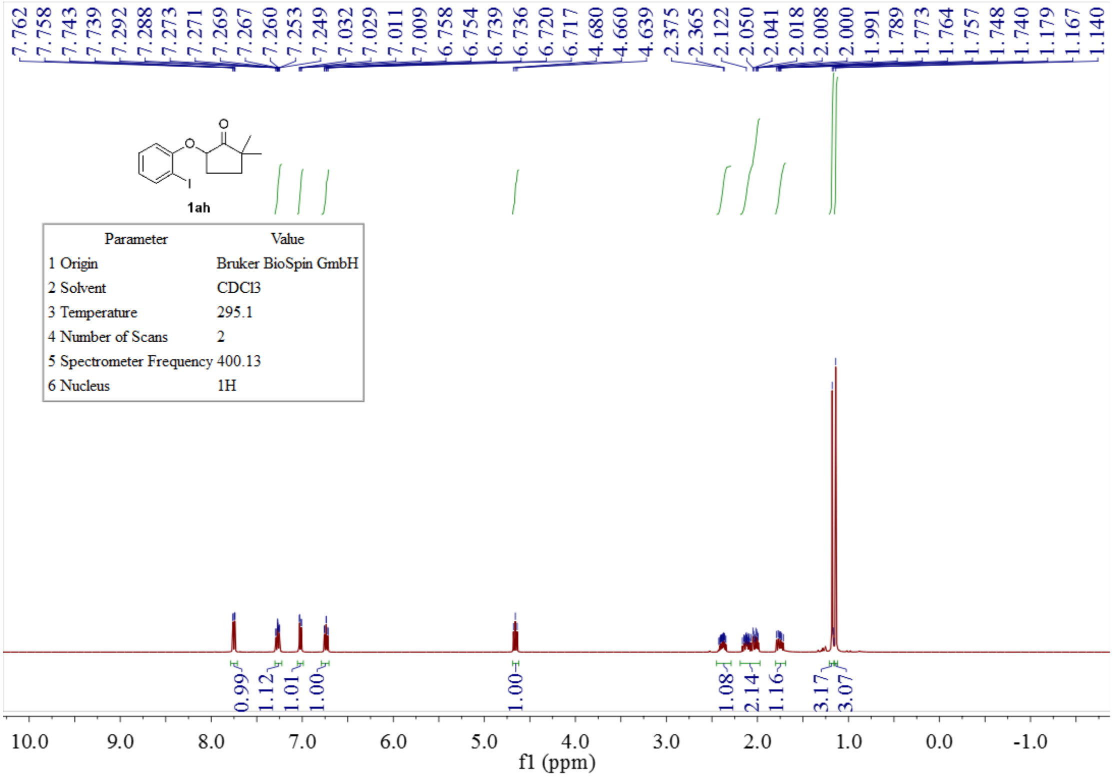

Supplementary Figure 84. **^{1}H NMR (400 MHz, $CDCl_3$) spectra for compound 1ah**

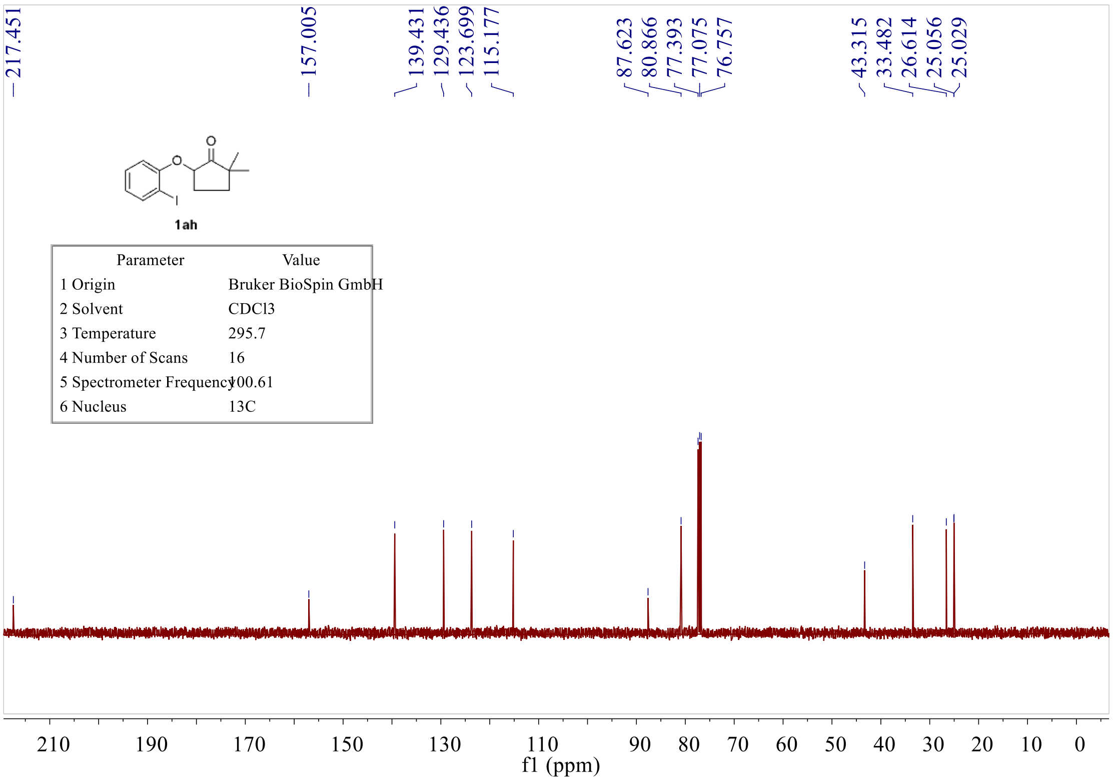

Supplementary Figure 85. **^{13}C NMR (100 MHz, $CDCl_3$) spectra for compound 1ah**

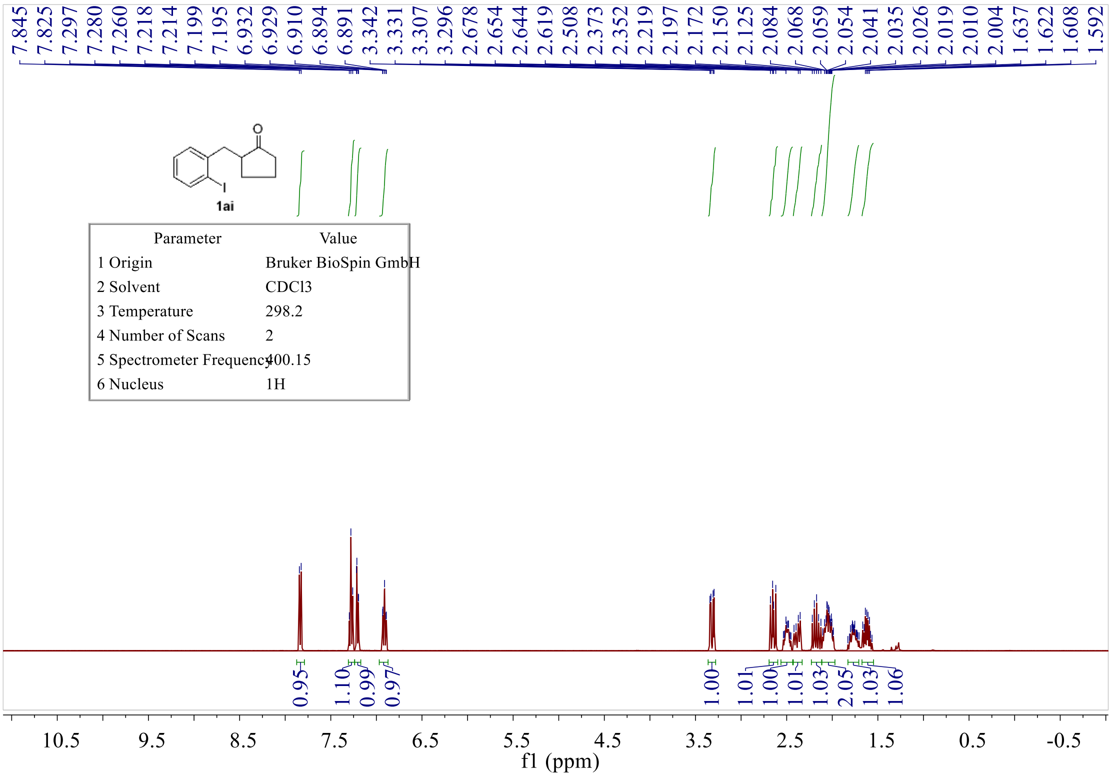

Supplementary Figure 86. ^{1}H **NMR (400 MHz, CDCl$_3$) spectra for compound 1ai**

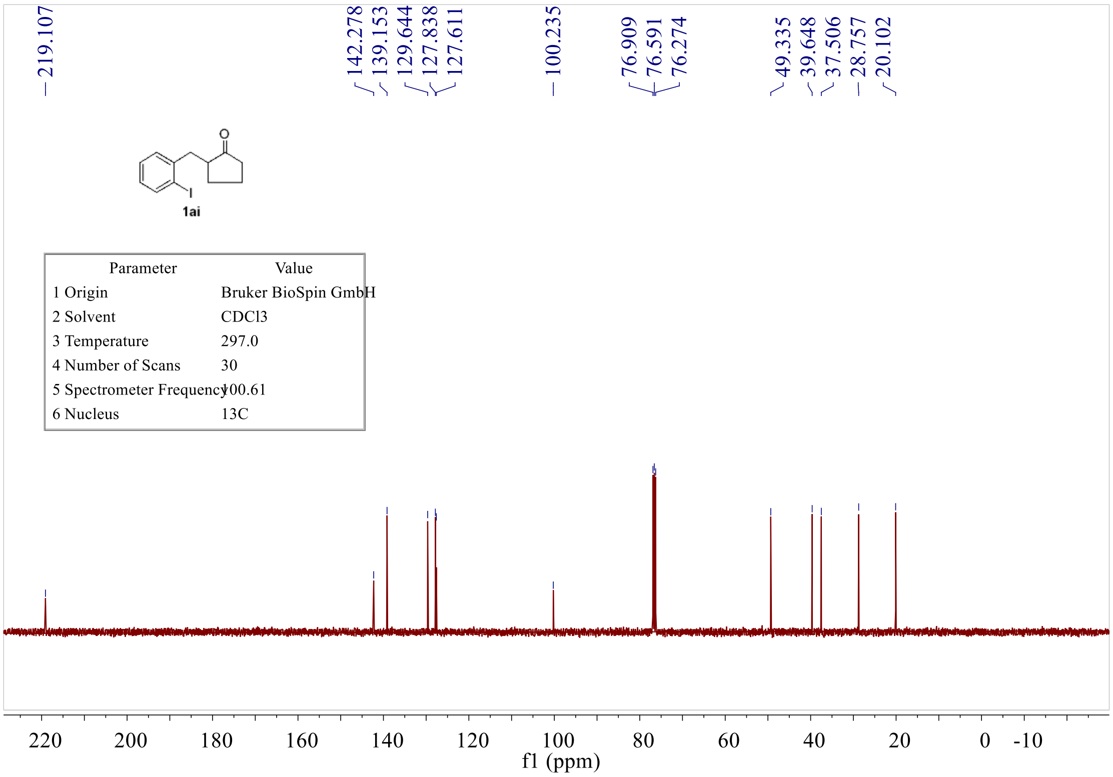

Supplementary Figure 87. ^{13}C **NMR (100 MHz, CDCl$_3$) spectra for compound 1ai**

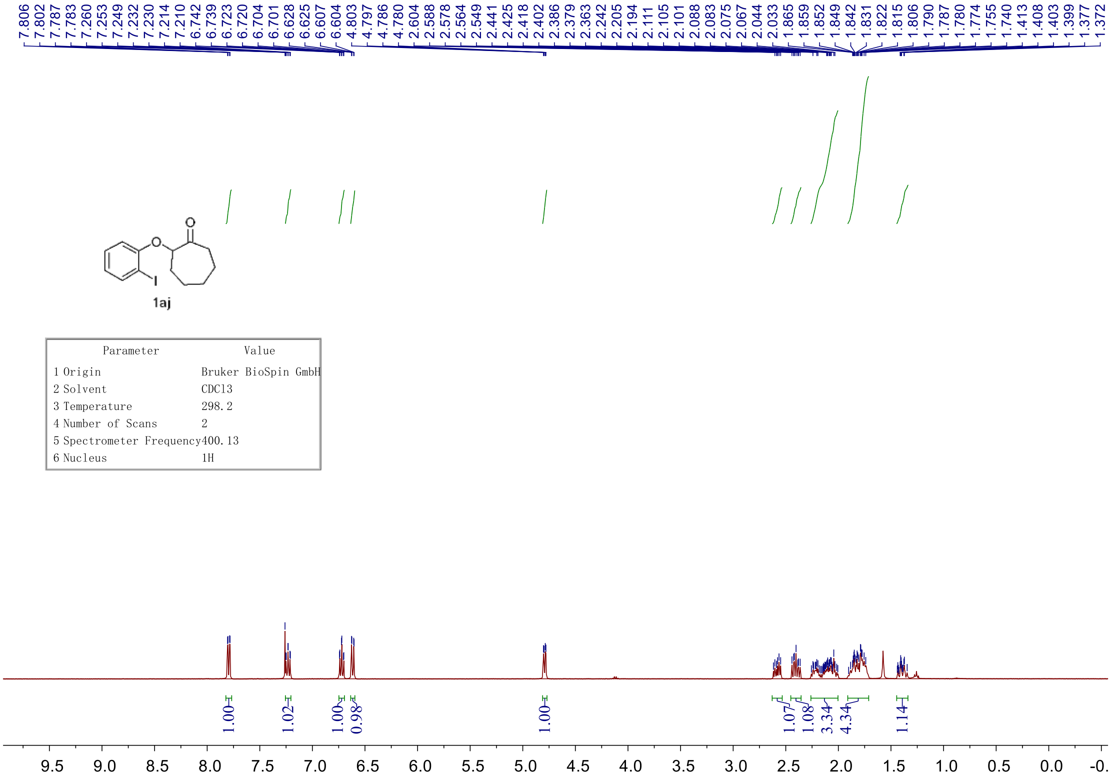

Supplementary Figure 88. ^{1}H NMR (400 MHz, $CDCl_3$) spectra for compound 1aj

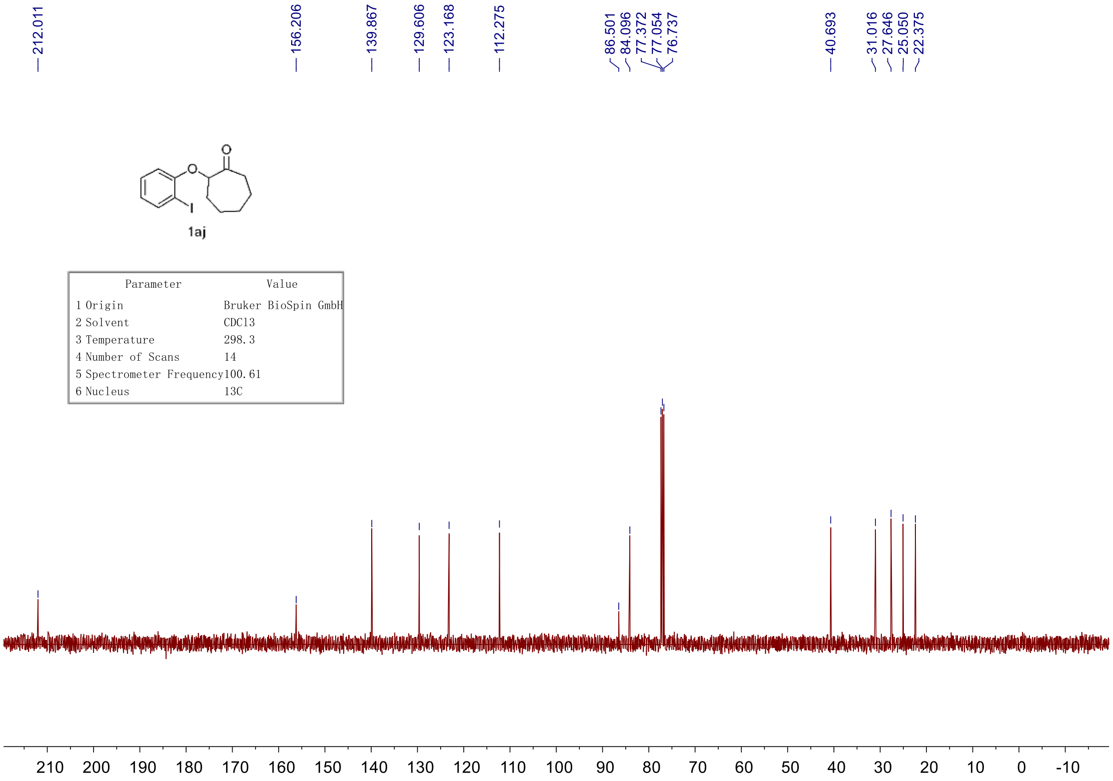

Supplementary Figure 89. ^{13}C NMR (100 MHz, $CDCl_3$) spectra for compound 1aj

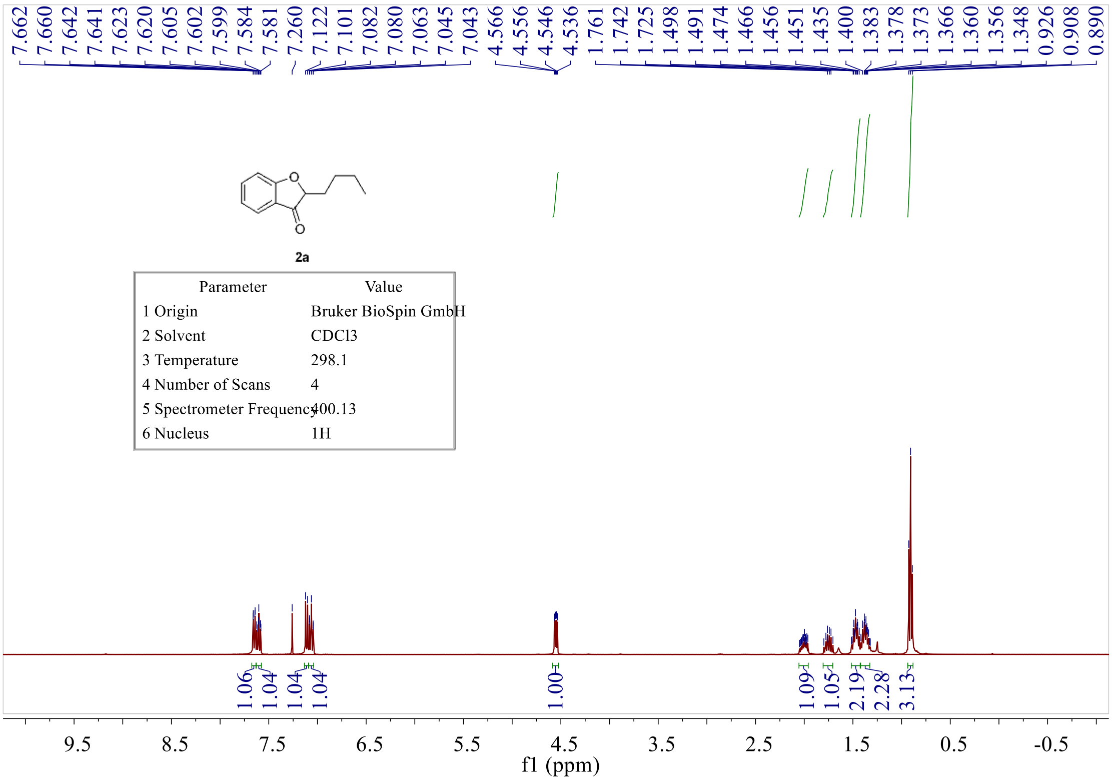

Supplementary Figure 90. ^{1}**H NMR (400 MHz, CDCl**$_3$**) spectra for compound 2a**

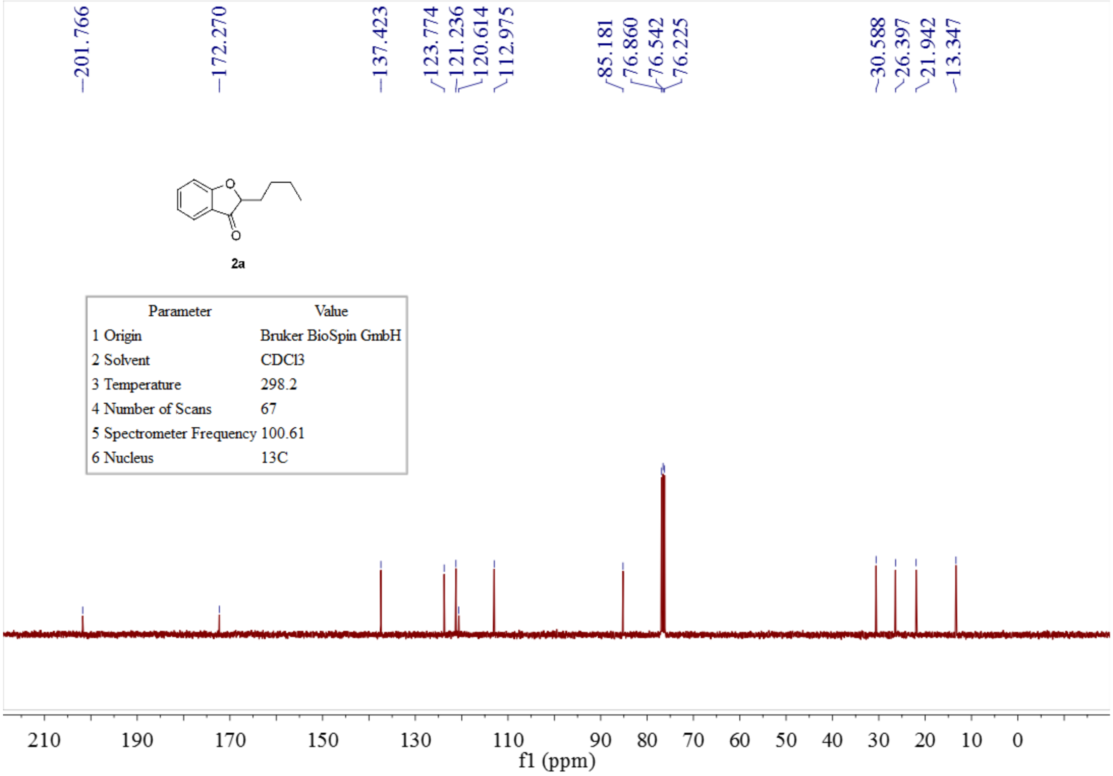

Supplementary Figure 91. ^{13}**C NMR (100 MHz, CDCl**$_3$**) spectra for compound 2a**

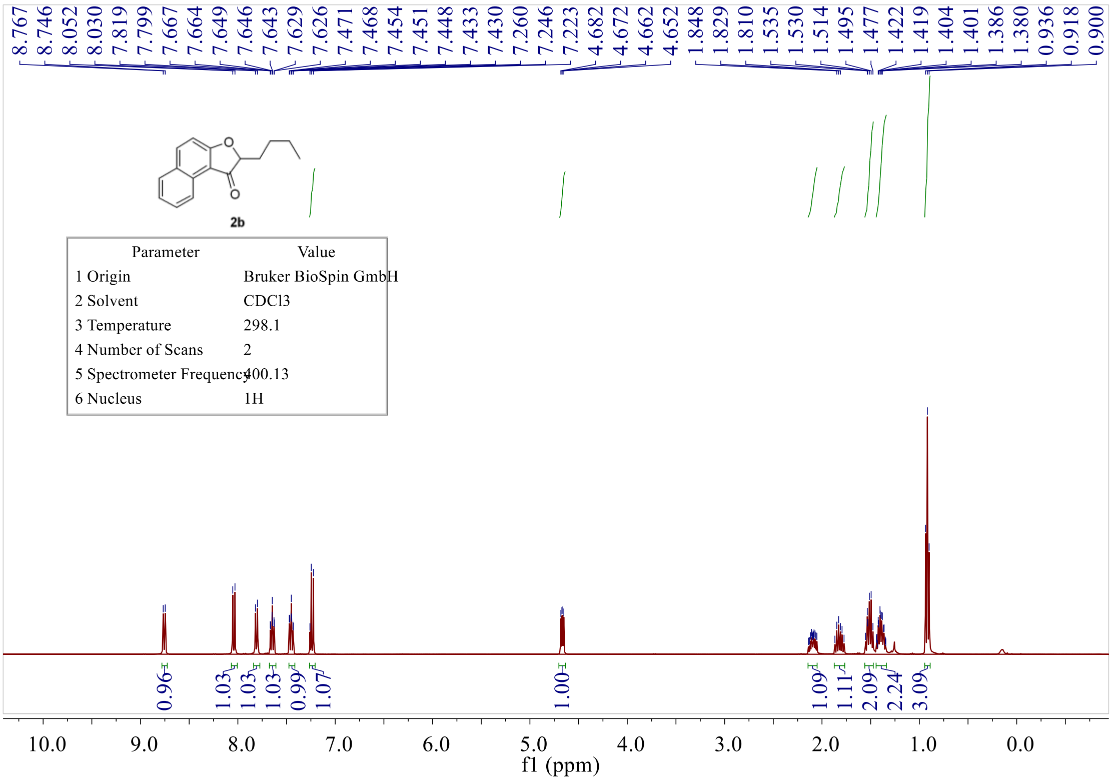

Supplementary Figure 92. ^{1}H NMR (400 MHz, $CDCl_3$) spectra for compound 2b

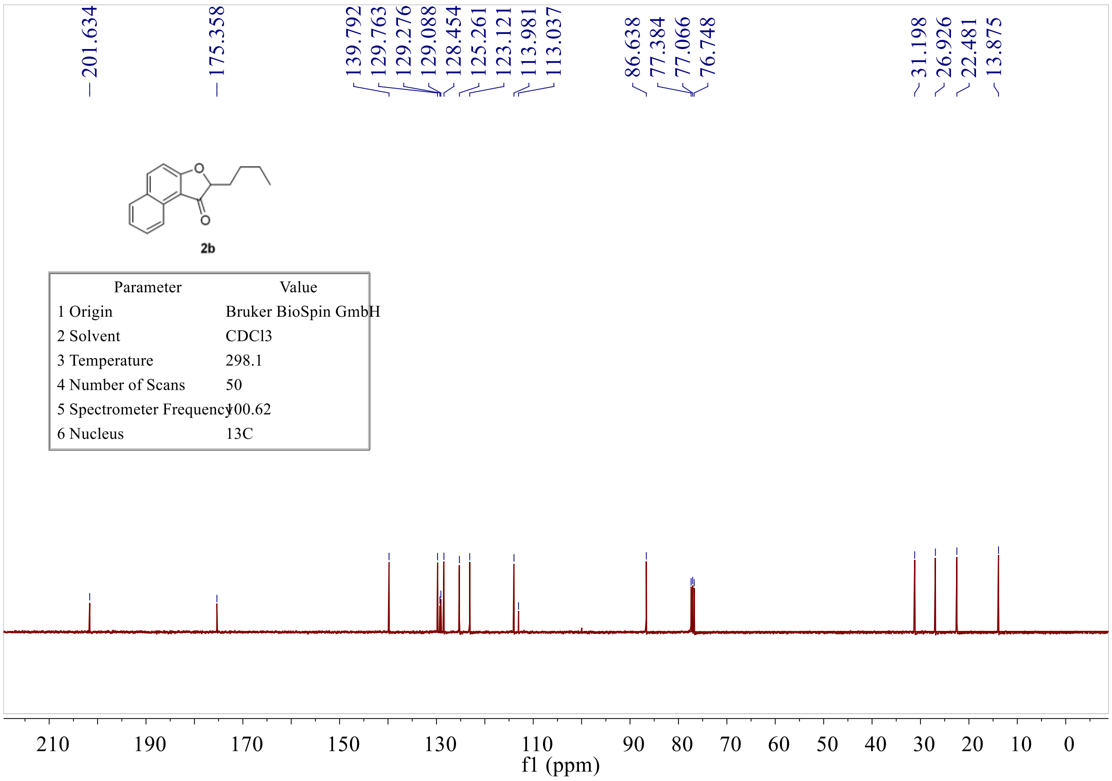

Supplementary Figure 93. ^{13}C NMR (100 MHz, $CDCl_3$) spectra for compound 2b

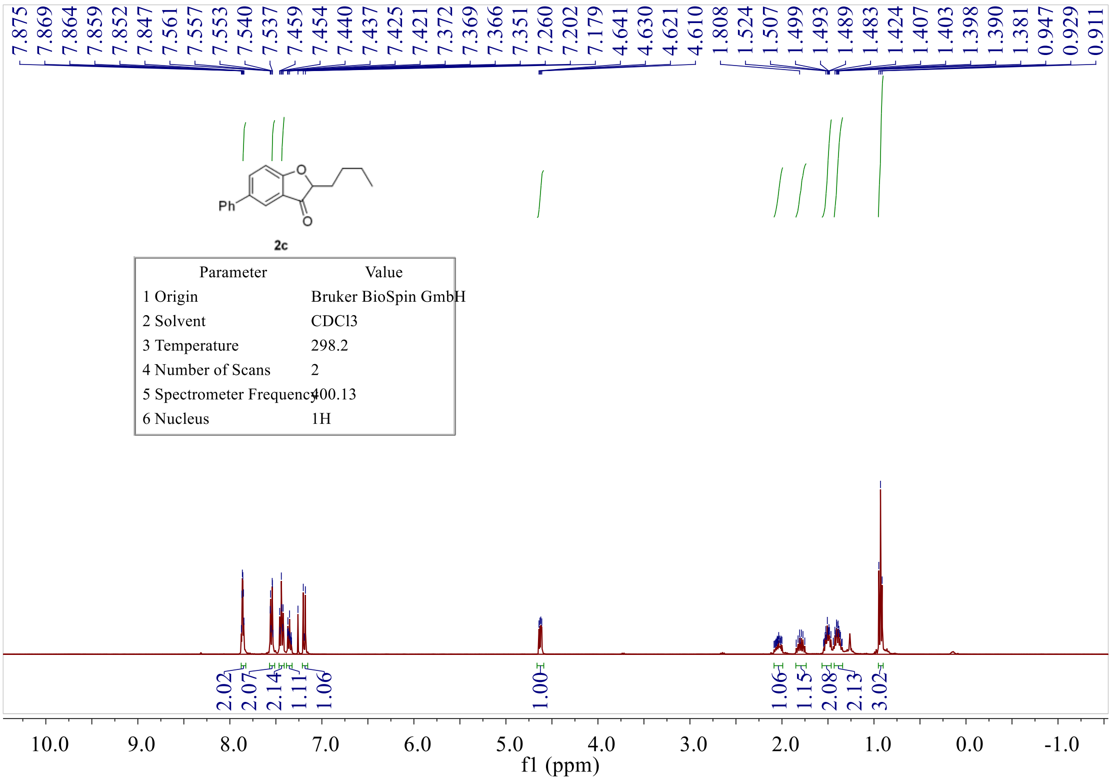

Supplementary Figure 94. ^{1}H NMR (400 MHz, $CDCl_3$) spectra for compound 2c

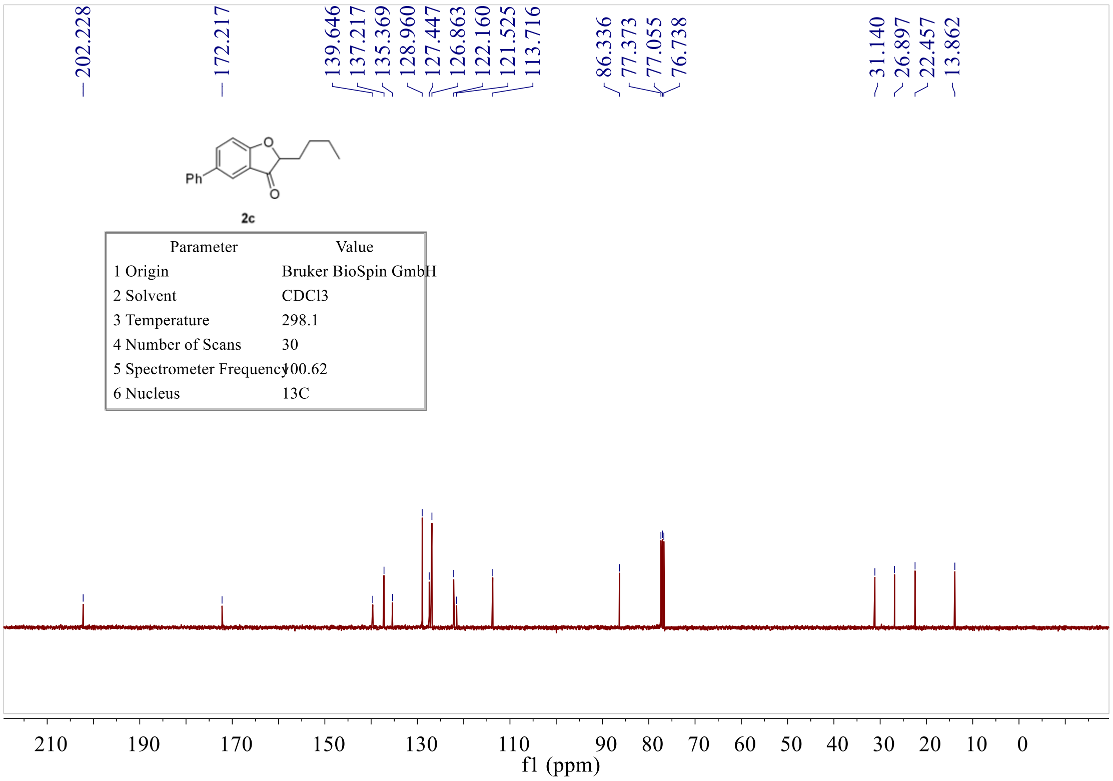

Supplementary Figure 95. ^{13}C NMR (100 MHz, $CDCl_3$) spectra for compound 2c

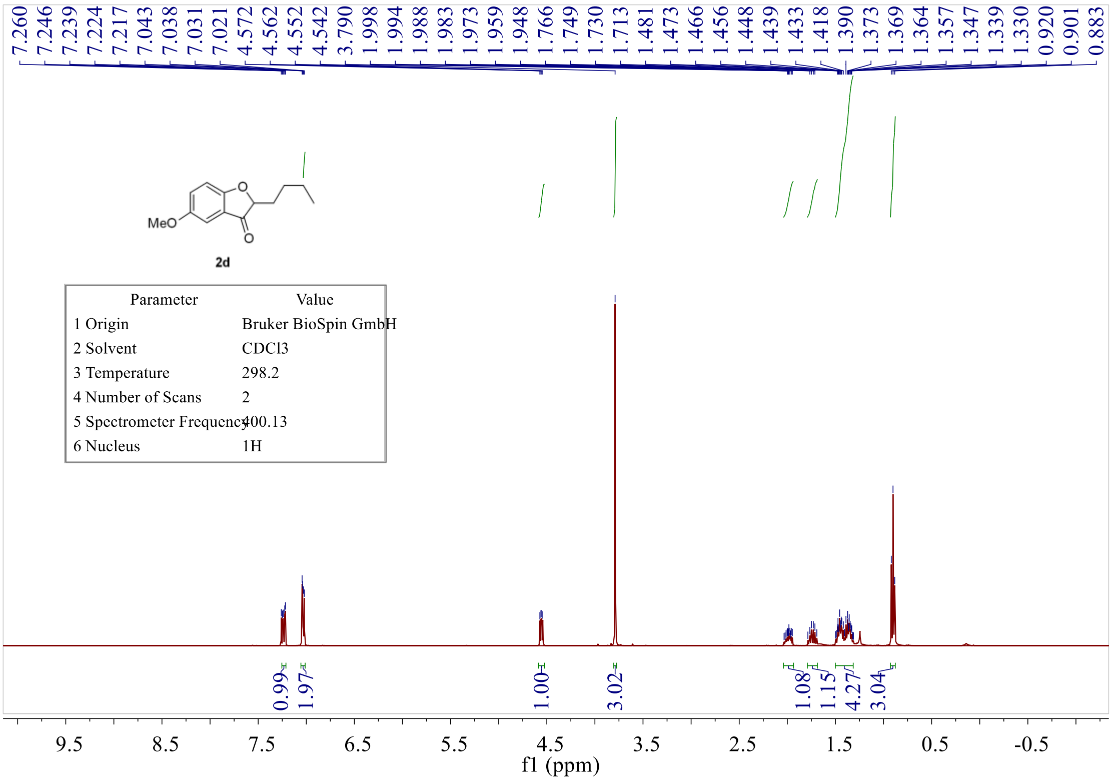

Supplementary Figure 96. ^{1}H NMR (400 MHz, $CDCl_3$) spectra for compound 2d

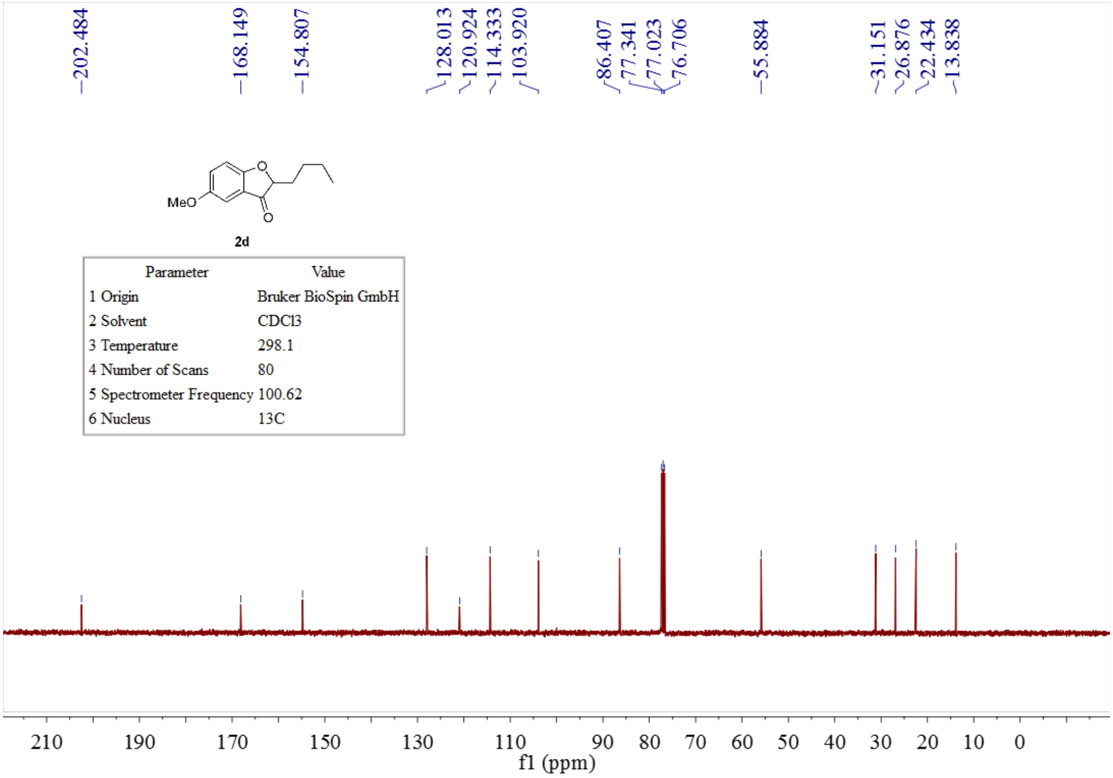

Supplementary Figure 97. ^{13}C NMR (100 MHz, $CDCl_3$) spectra for compound 2d

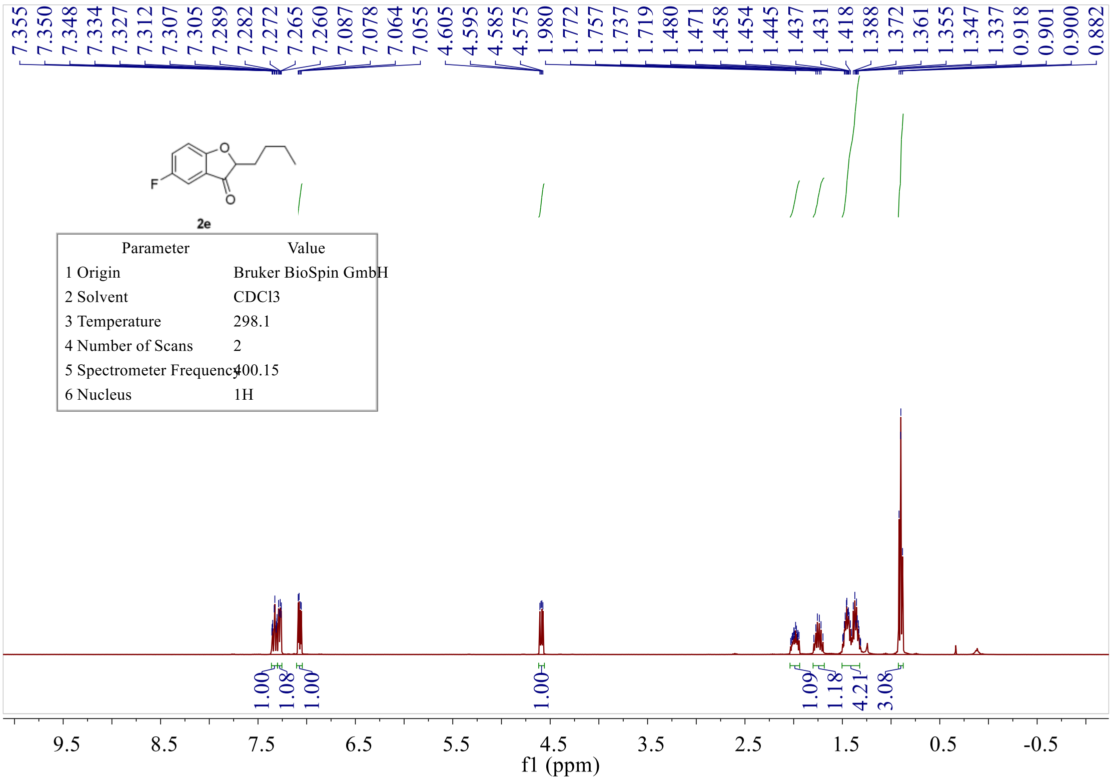

Supplementary Figure 98. ^{1}H NMR (400 MHz, $CDCl_3$) spectra for compound 2e

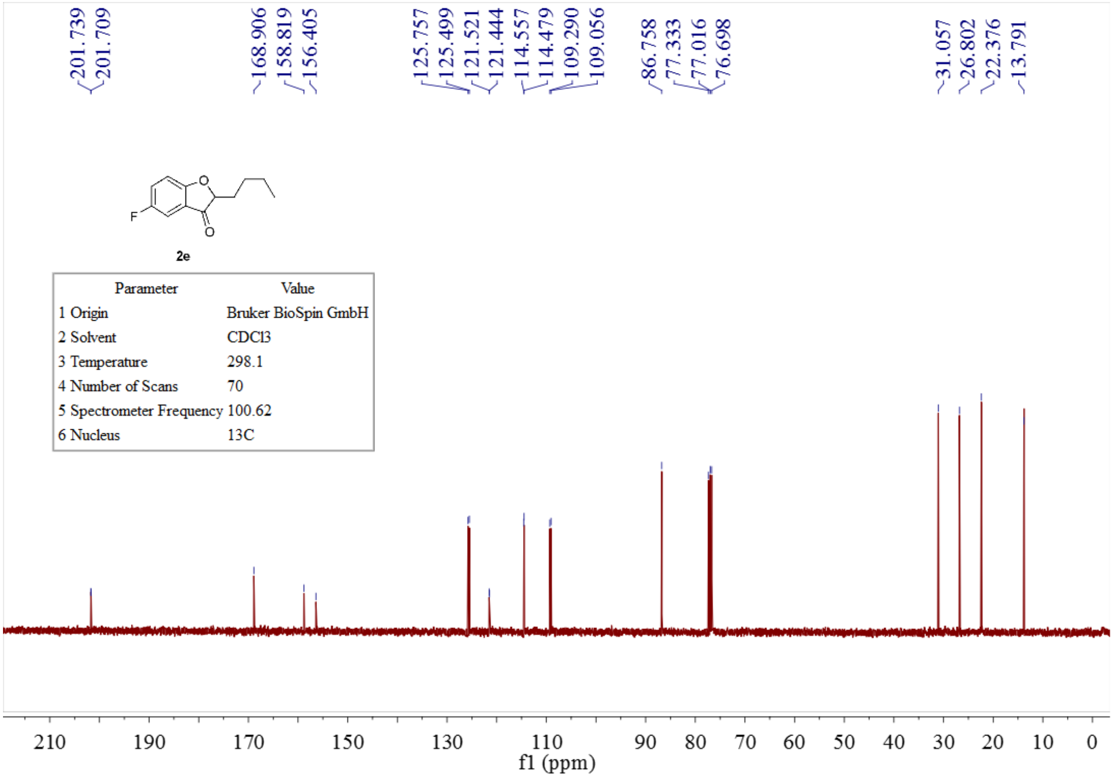

Supplementary Figure 99. ^{13}C NMR (100 MHz, $CDCl_3$) spectra for compound 2e

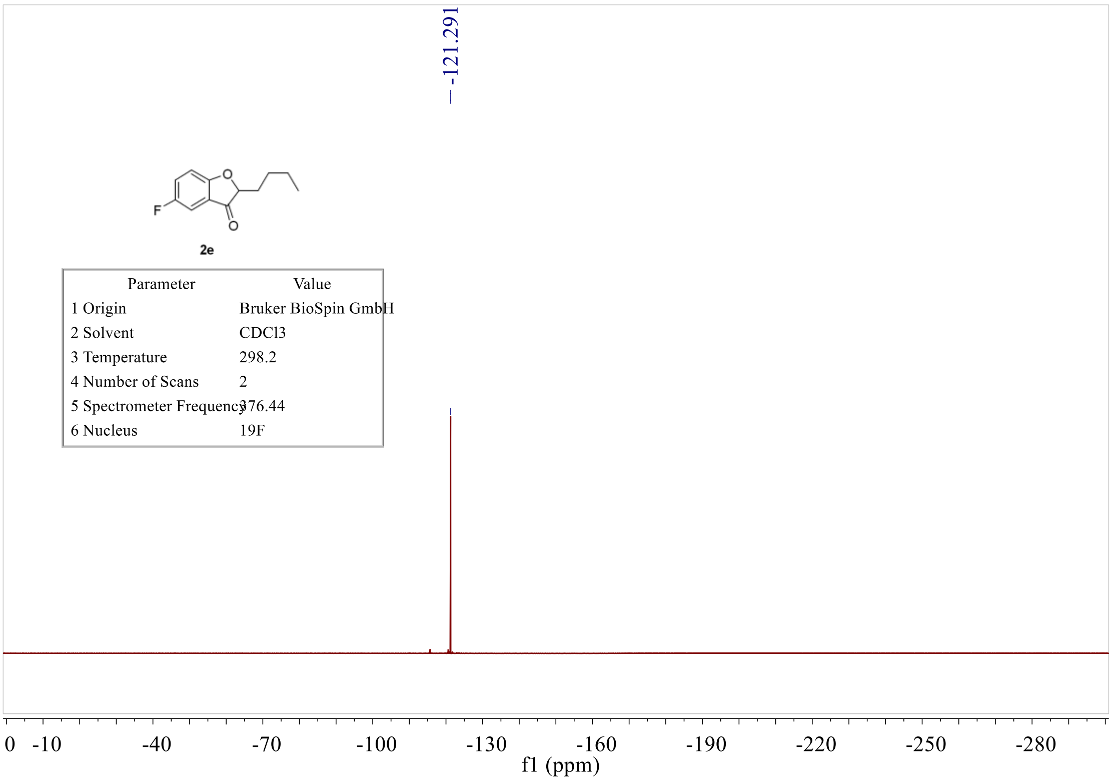

Supplementary Figure 100. ^{19}**F NMR (376 MHz, CDCl**$_3$**) spectra for compound 2e**

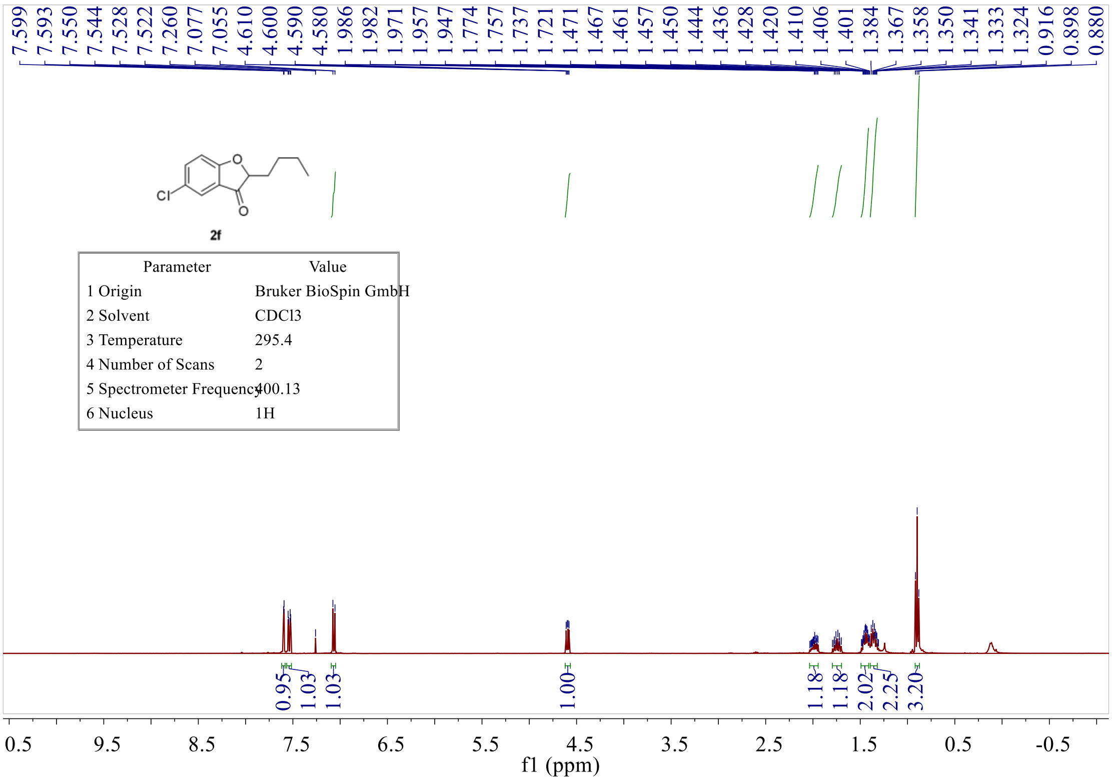

Supplementary Figure 101. ^{1}**H NMR (400 MHz, CDCl**$_3$**) spectra for compound 2f**

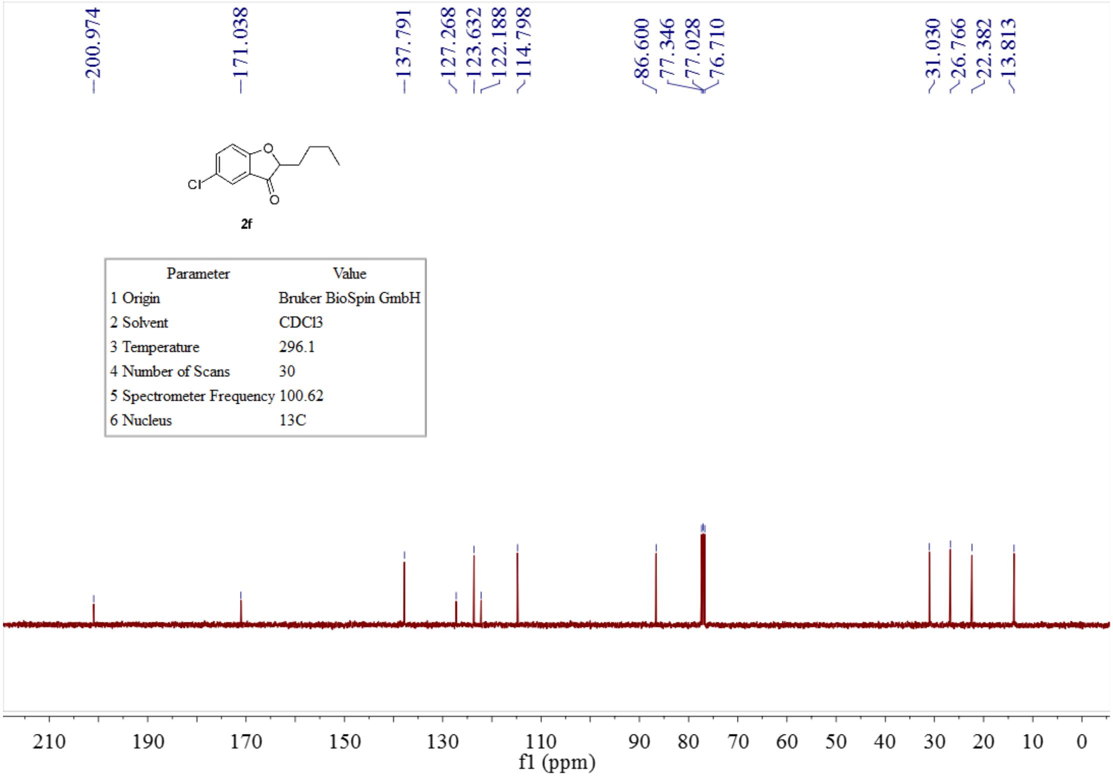

Supplementary Figure 102. ^{13}**C NMR (100 MHz, CDCl$_3$) spectra for compound 2f**

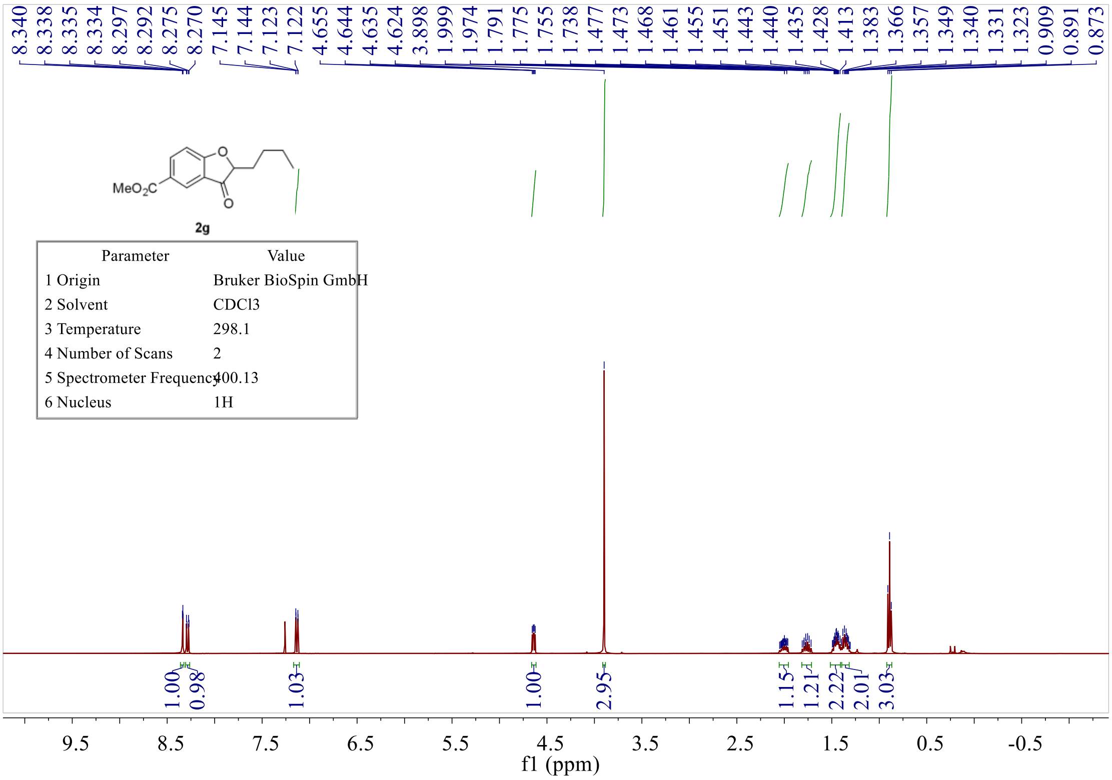

Supplementary Figure 103. ^{1}**H NMR (400 MHz, CDCl$_3$) spectra for compound 2g**

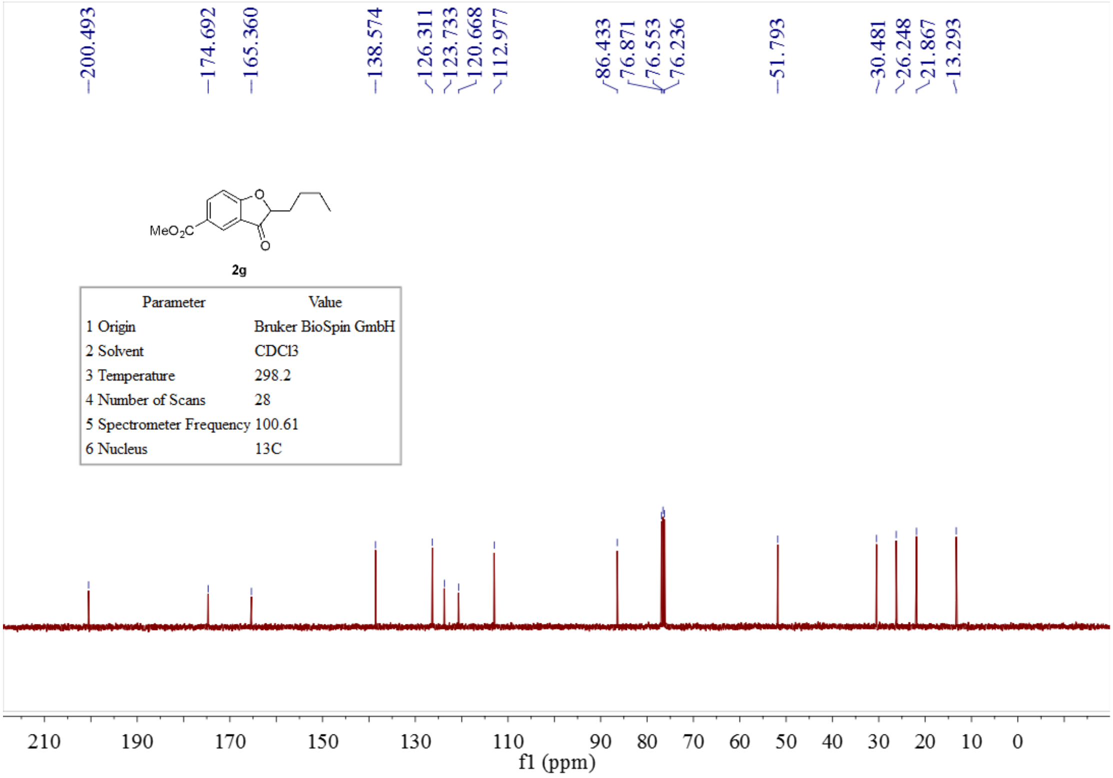

Supplementary Figure 104. ^{13}**C NMR (100 MHz, CDCl**$_3$**) spectra for compound 2g**

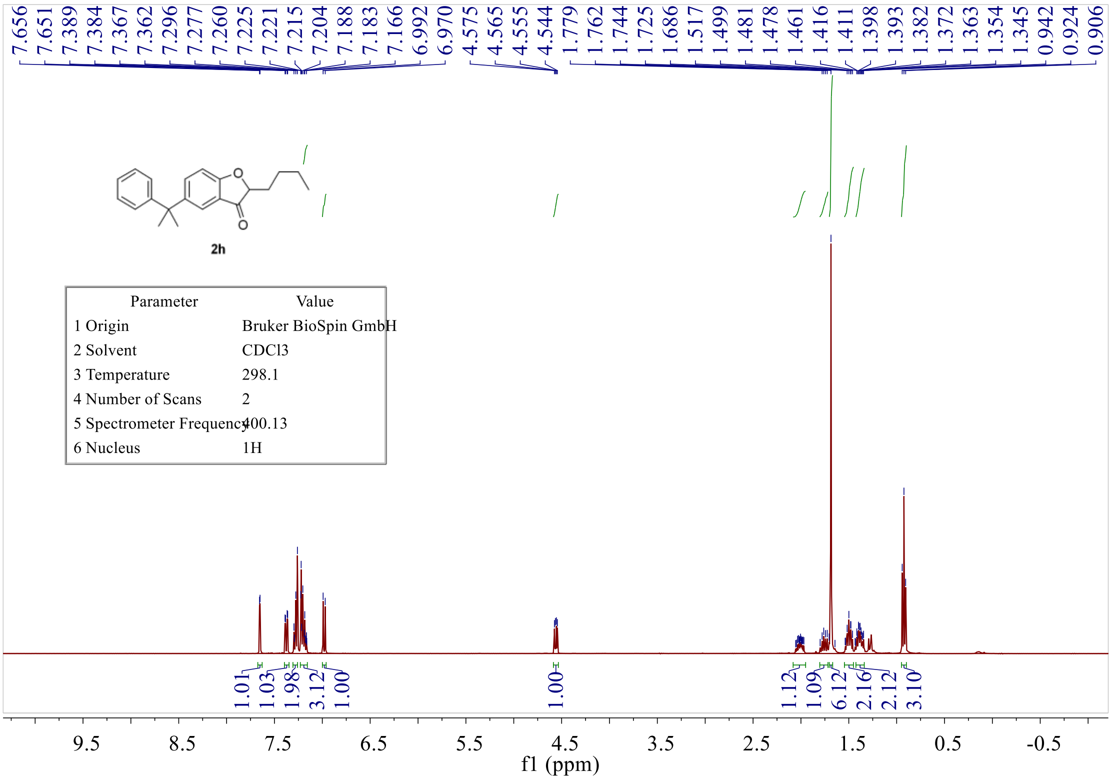

Supplementary Figure 105. ^{1}**H NMR (400 MHz, CDCl**$_3$**) spectra for compound 2h**

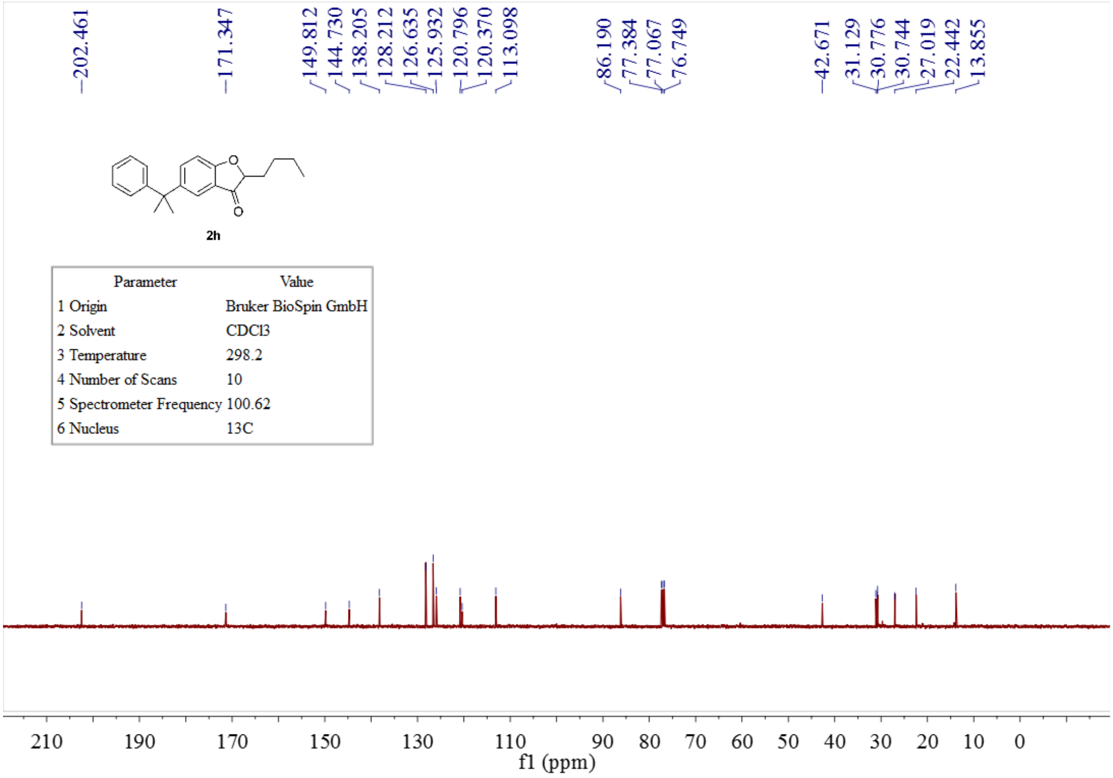

Supplementary Figure 106. **^{13}C NMR (100 MHz, $CDCl_3$) spectra for compound 2h**

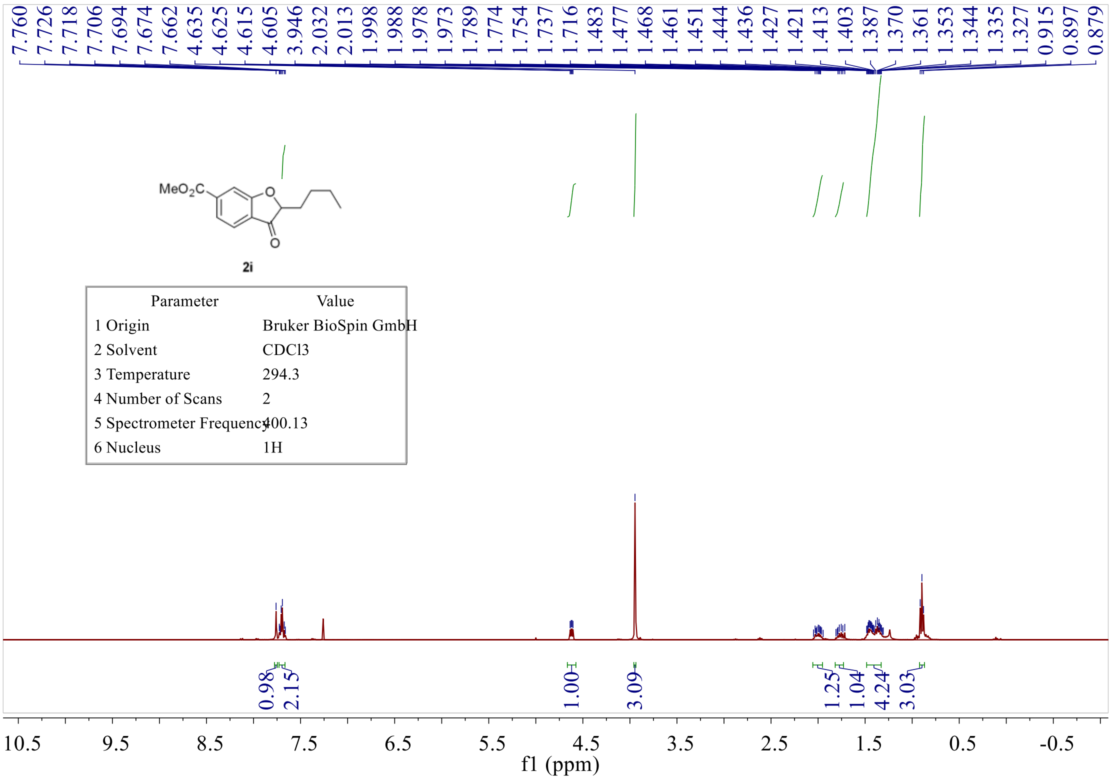

Supplementary Figure 107. **^{1}H NMR (400 MHz, $CDCl_3$) spectra for compound 2i**

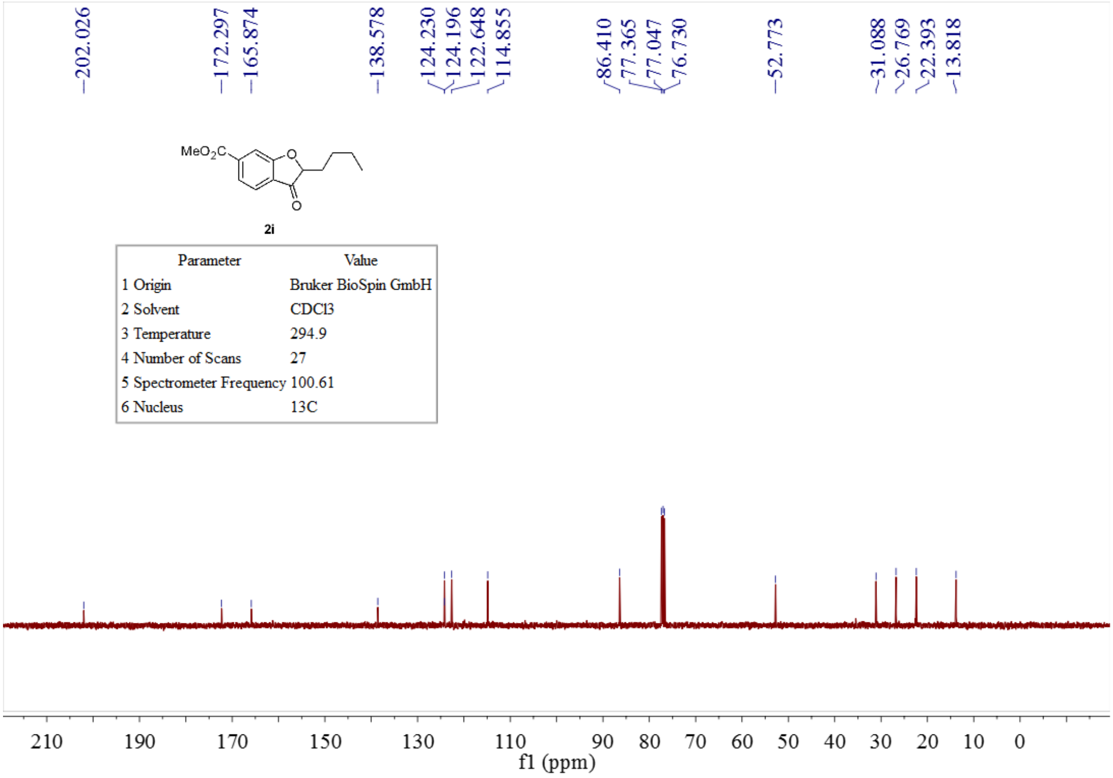

Supplementary Figure 108. ^{13}**C NMR (100 MHz, CDCl$_3$) spectra for compound 2i**

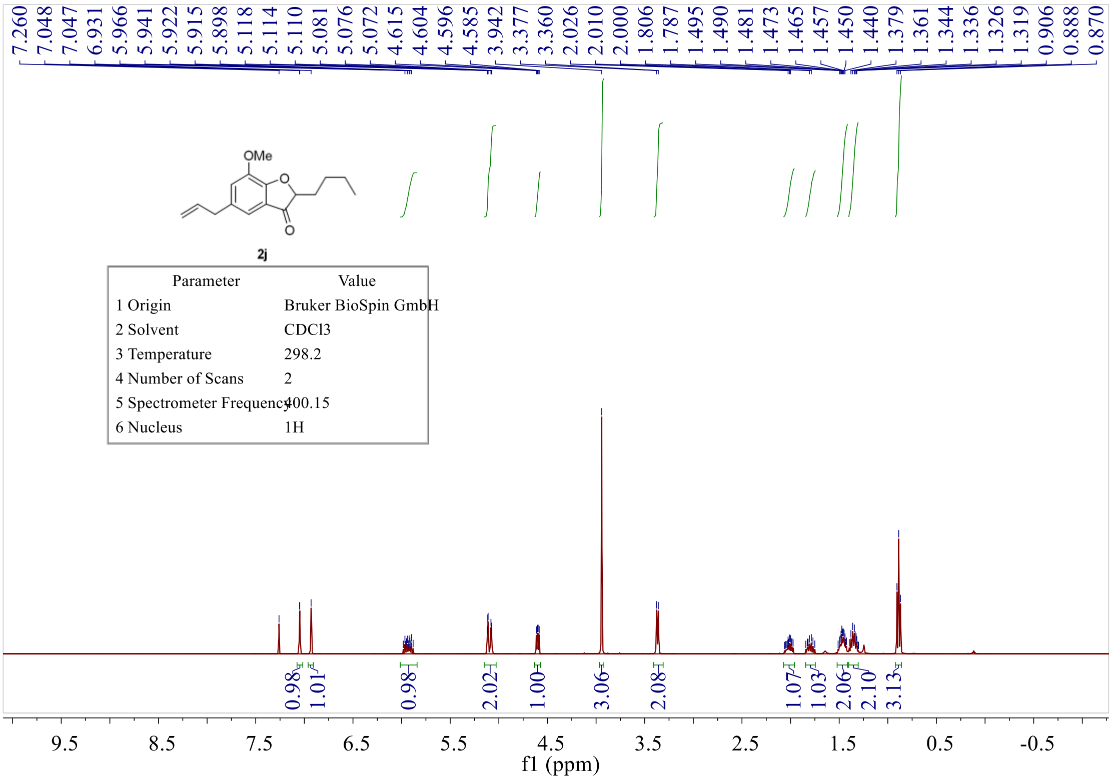

Supplementary Figure 109. ^{1}**H NMR (400 MHz, CDCl$_3$) spectra for compound 2j**

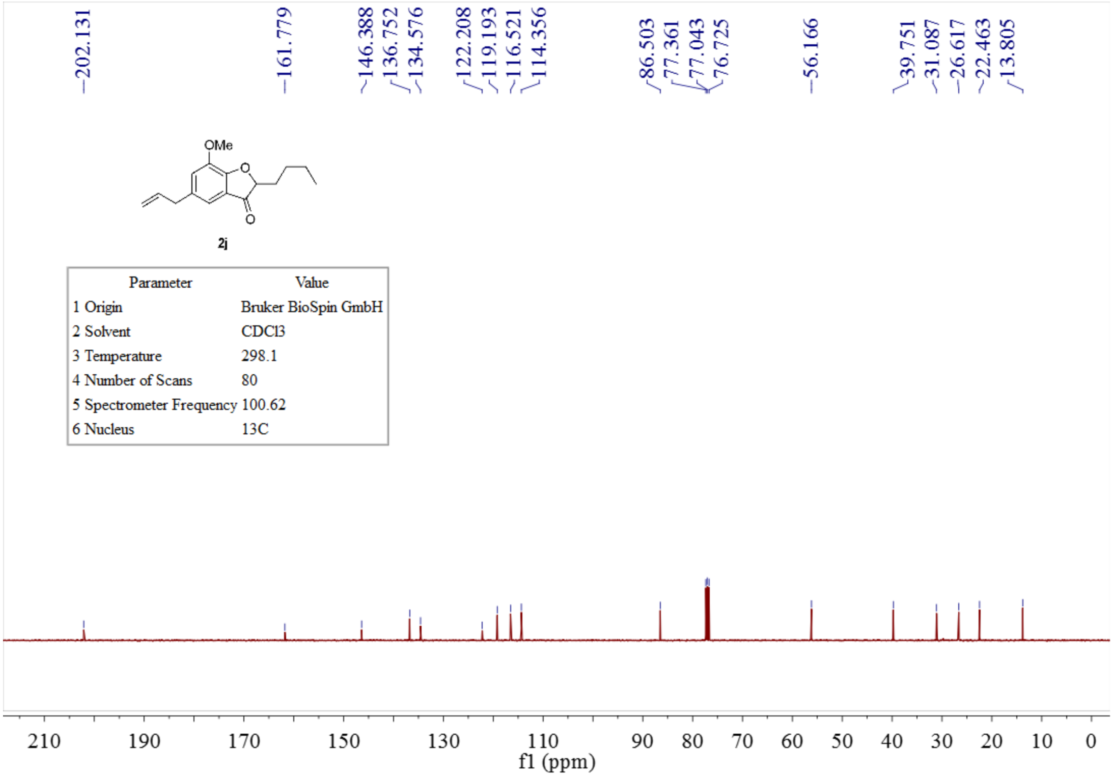

Supplementary Figure 110. **^{13}C NMR (100 MHz, $CDCl_3$) spectra for compound 2j**

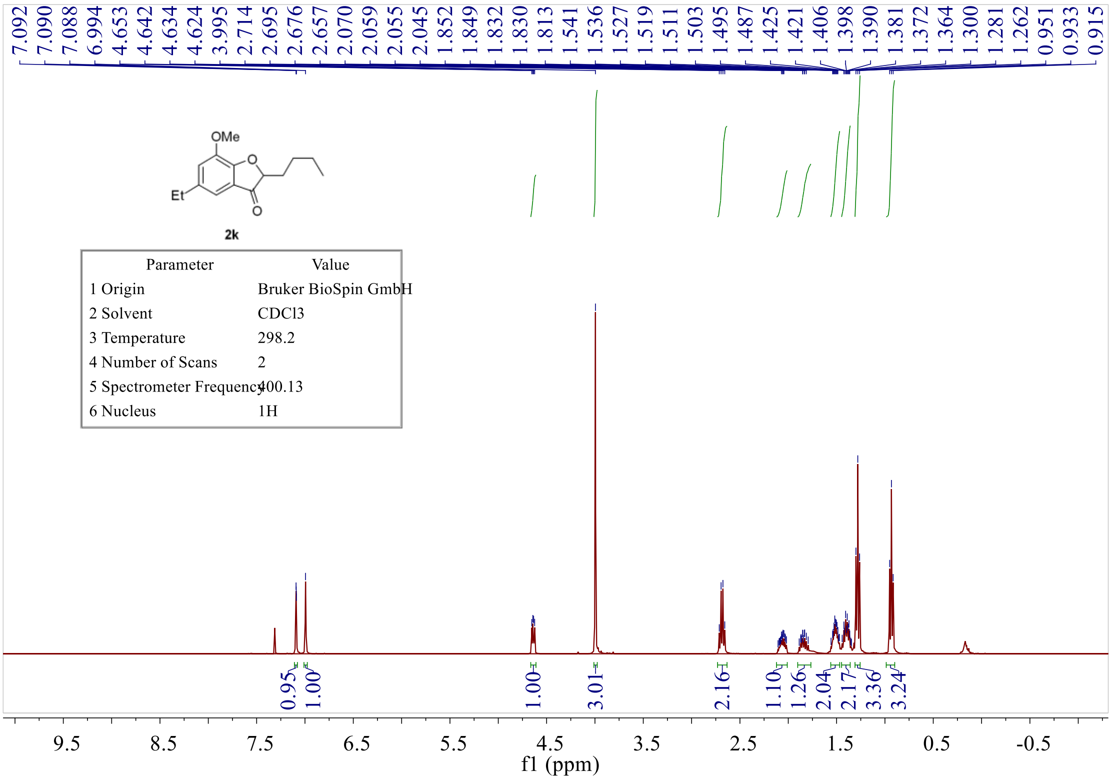

Supplementary Figure 111. **^{1}H NMR (400 MHz, $CDCl_3$) spectra for compound 2k**

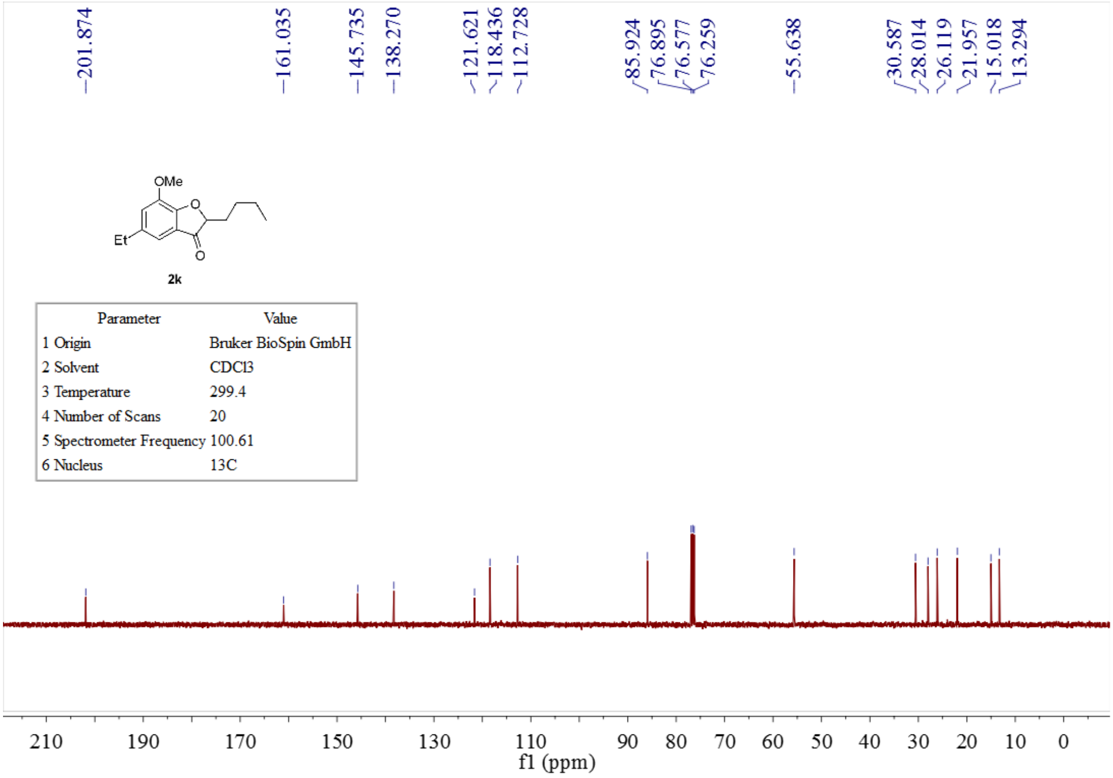

Supplementary Figure 112. ^{13}C NMR (100 MHz, $CDCl_3$) spectra for compound 2k

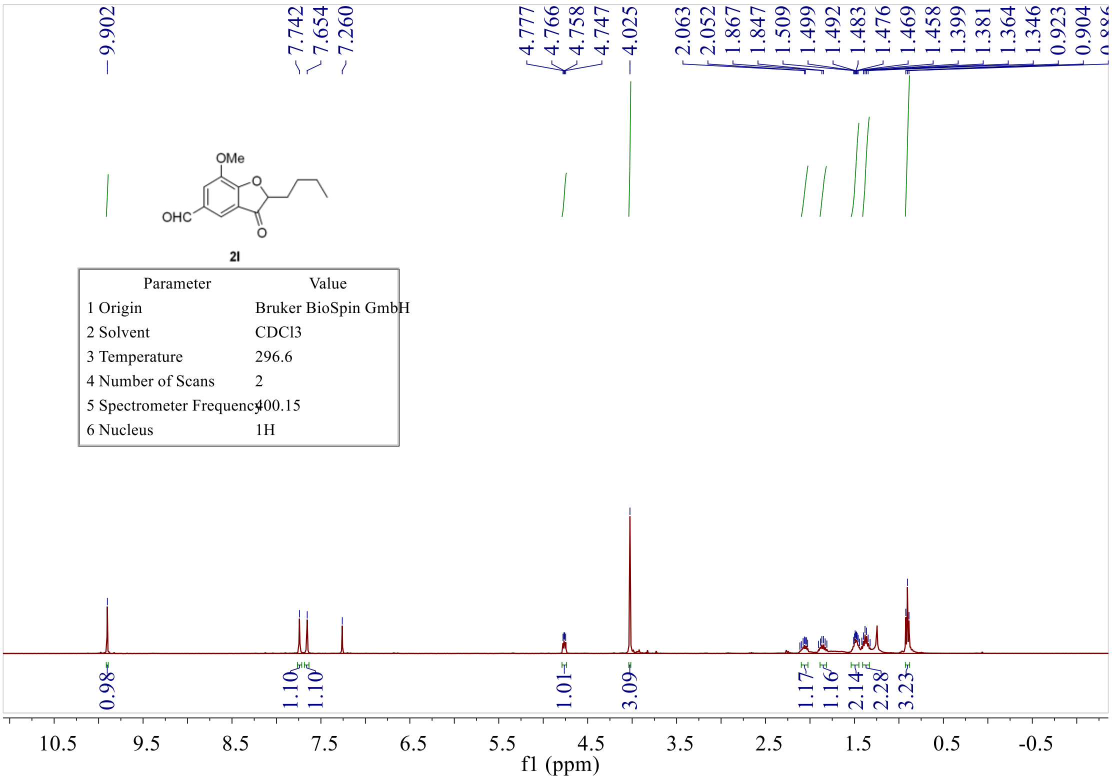

Supplementary Figure 113. ^{1}H NMR (400 MHz, $CDCl_3$) spectra for compound 2l

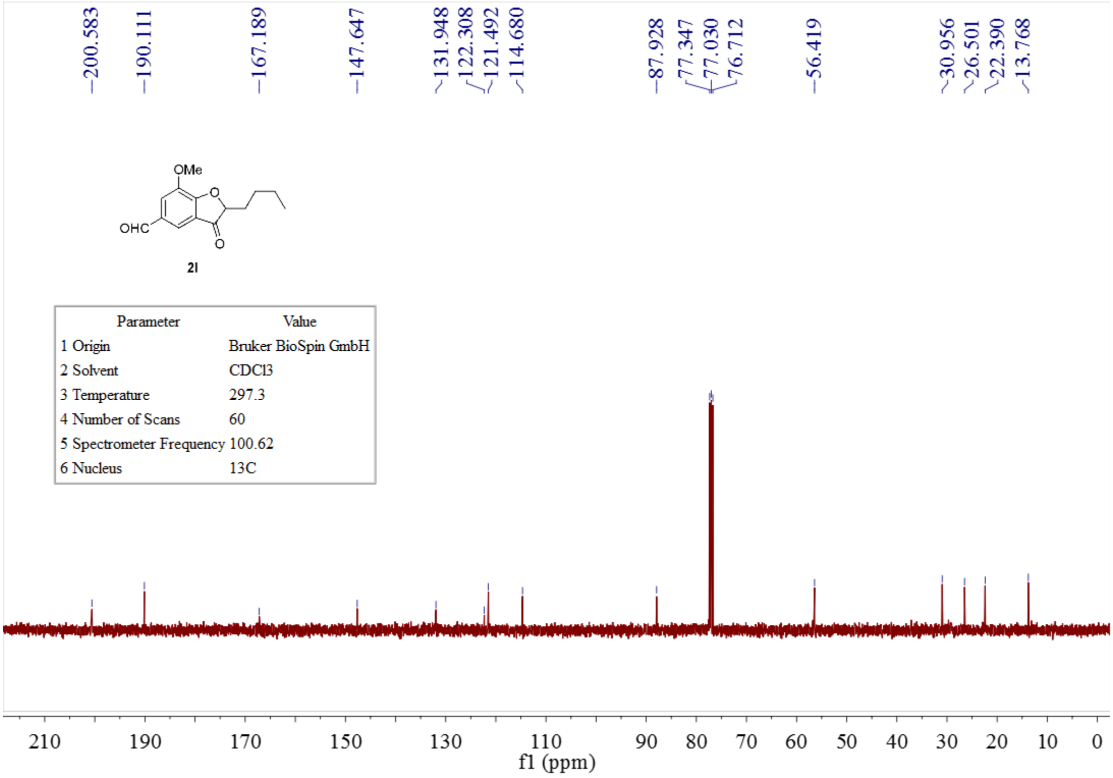

Supplementary Figure 114. **^{13}C NMR (100 MHz, CDCl$_3$) spectra for compound 2l**

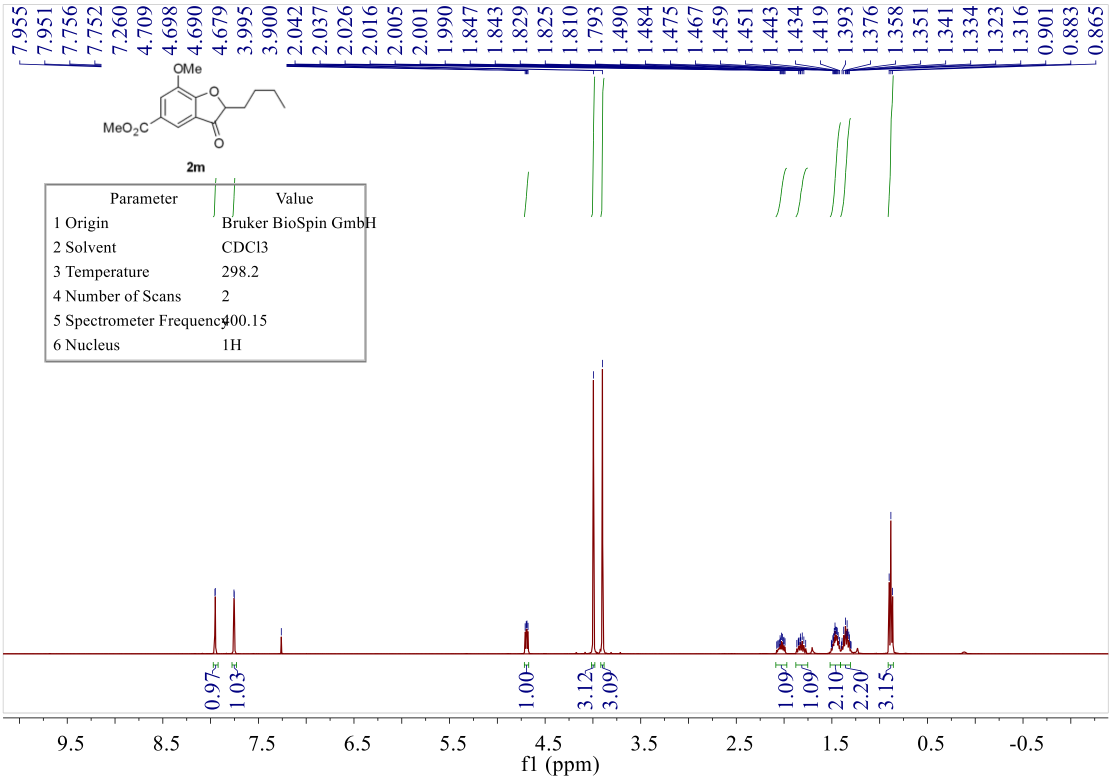

Supplementary Figure 115. **^{1}H NMR (400 MHz, CDCl$_3$) spectra for compound 2m**

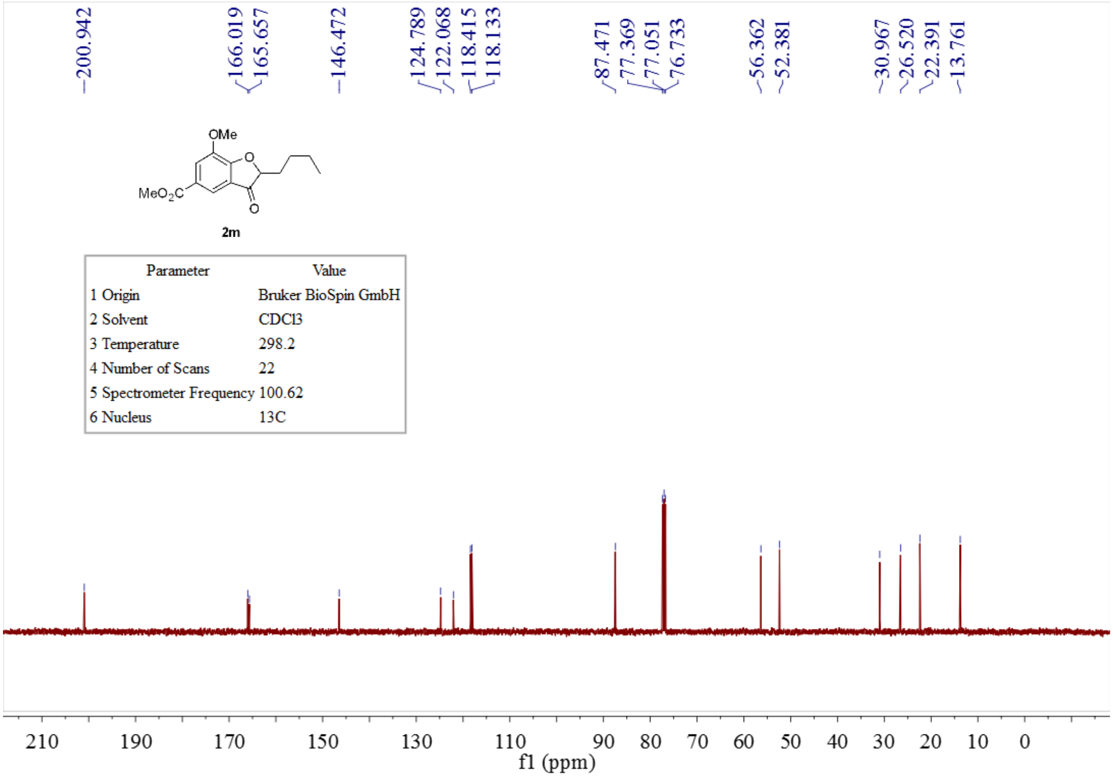

Supplementary Figure 116. ^{13}**C NMR (100 MHz, CDCl$_3$) spectra for compound 2m**

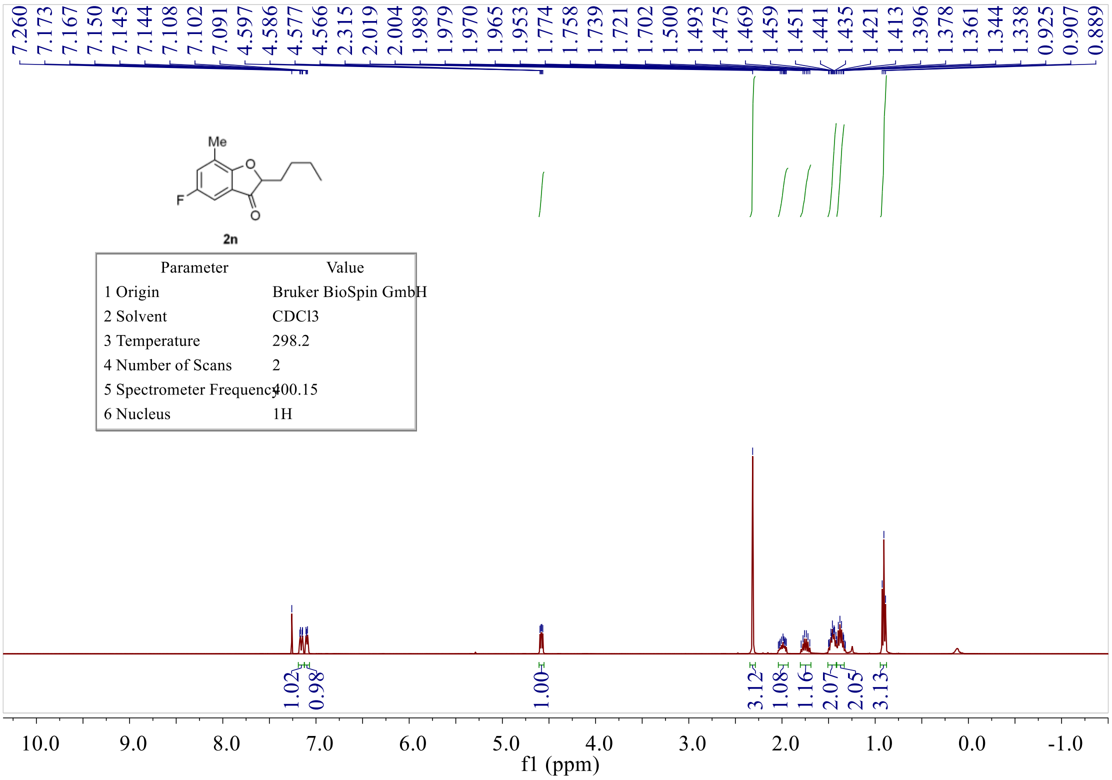

Supplementary Figure 117. ^{1}**H NMR (400 MHz, CDCl$_3$) spectra for compound 2n**

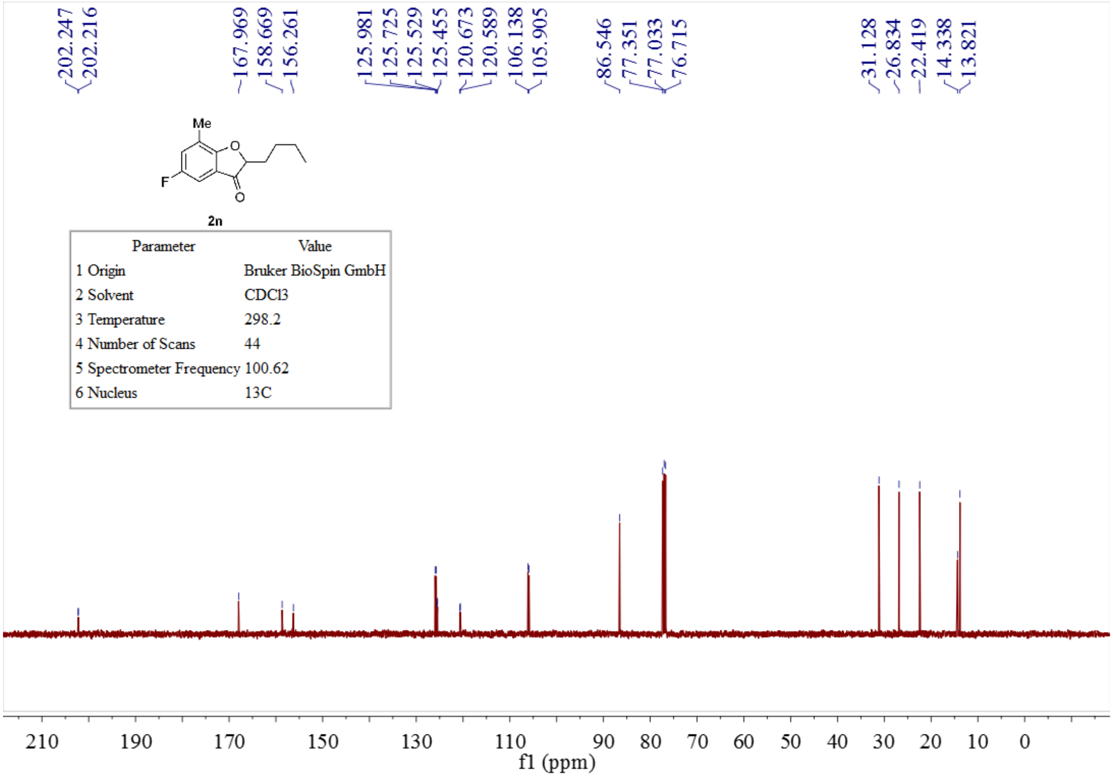

Supplementary Figure 118. **^{13}C NMR (100 MHz, $CDCl_3$) spectra for compound 2n**

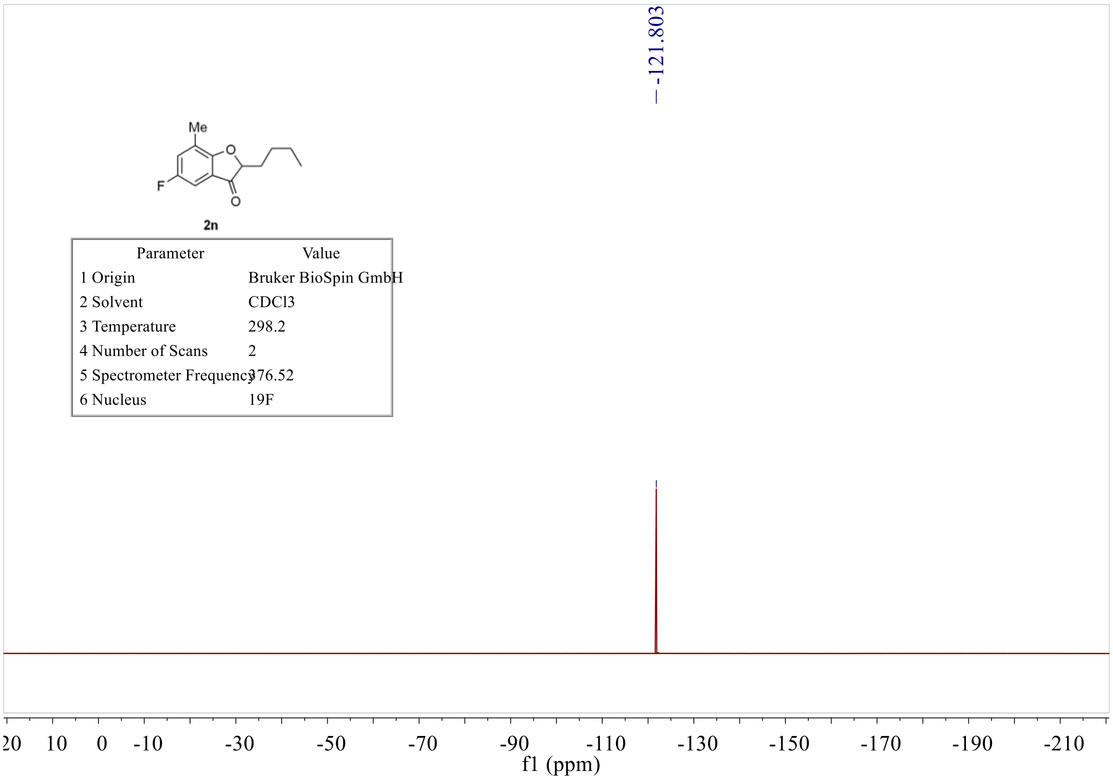

Supplementary Figure 119. **^{19}F NMR (376 MHz, $CDCl_3$) spectra for compound 2n**

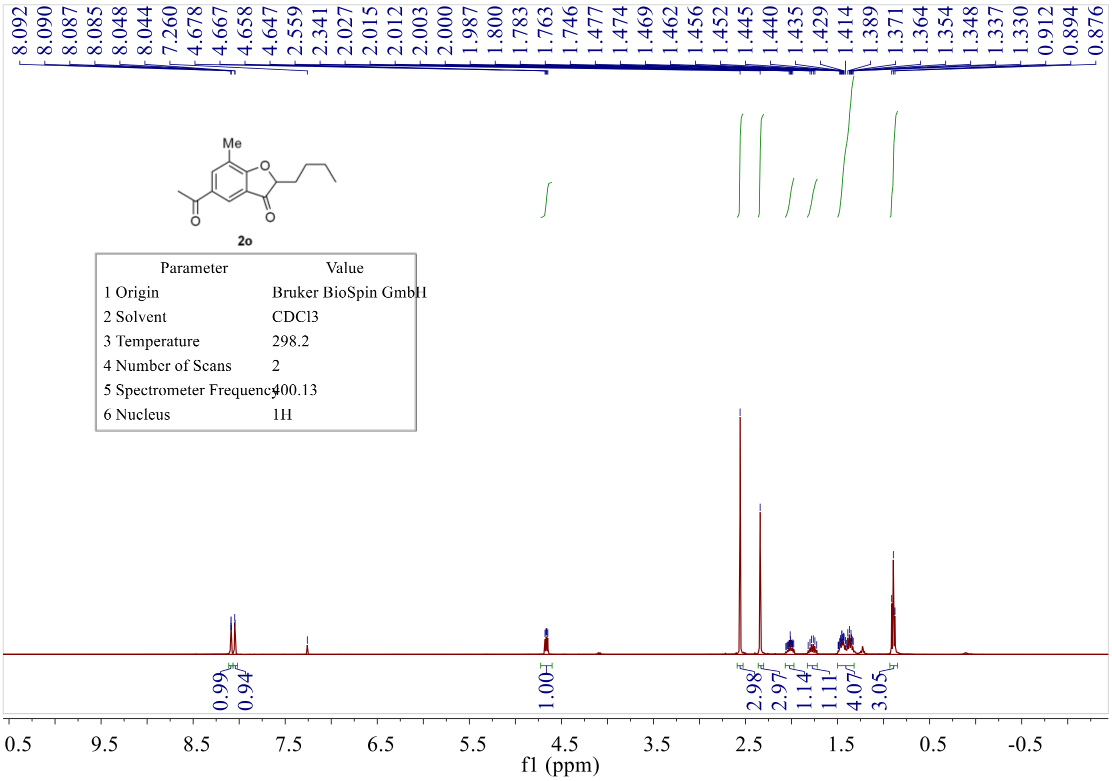

Supplementary Figure 120. **^{1}H NMR (400 MHz, $CDCl_3$) spectra for compound 2o**

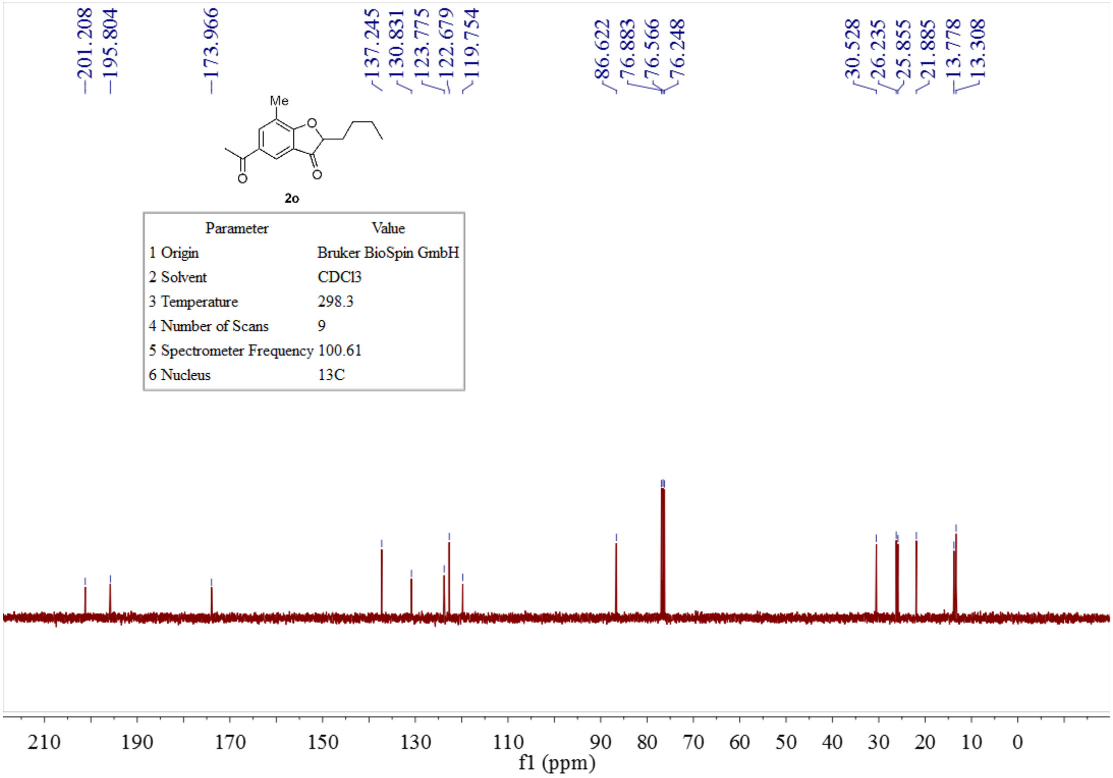

Supplementary Figure 121. **^{13}C NMR (100 MHz, $CDCl_3$) spectra for compound 2o**

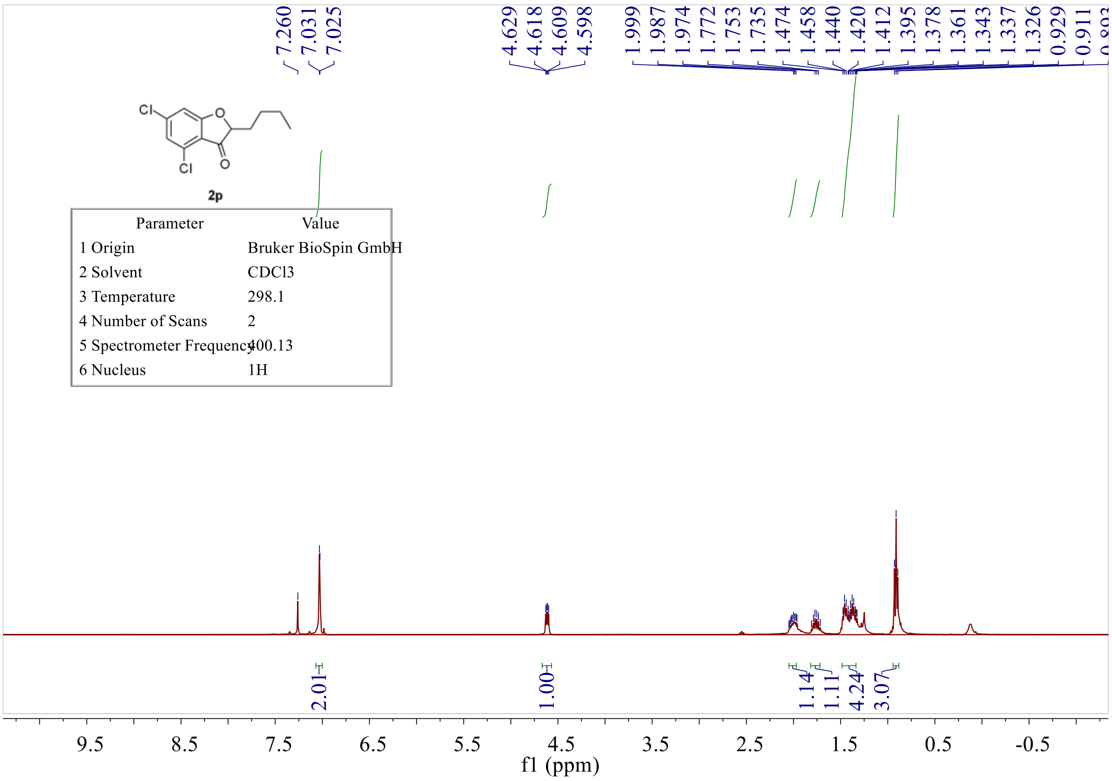

Supplementary Figure 122. **^{1}H NMR (400 MHz, $CDCl_3$) spectra for compound 2p**

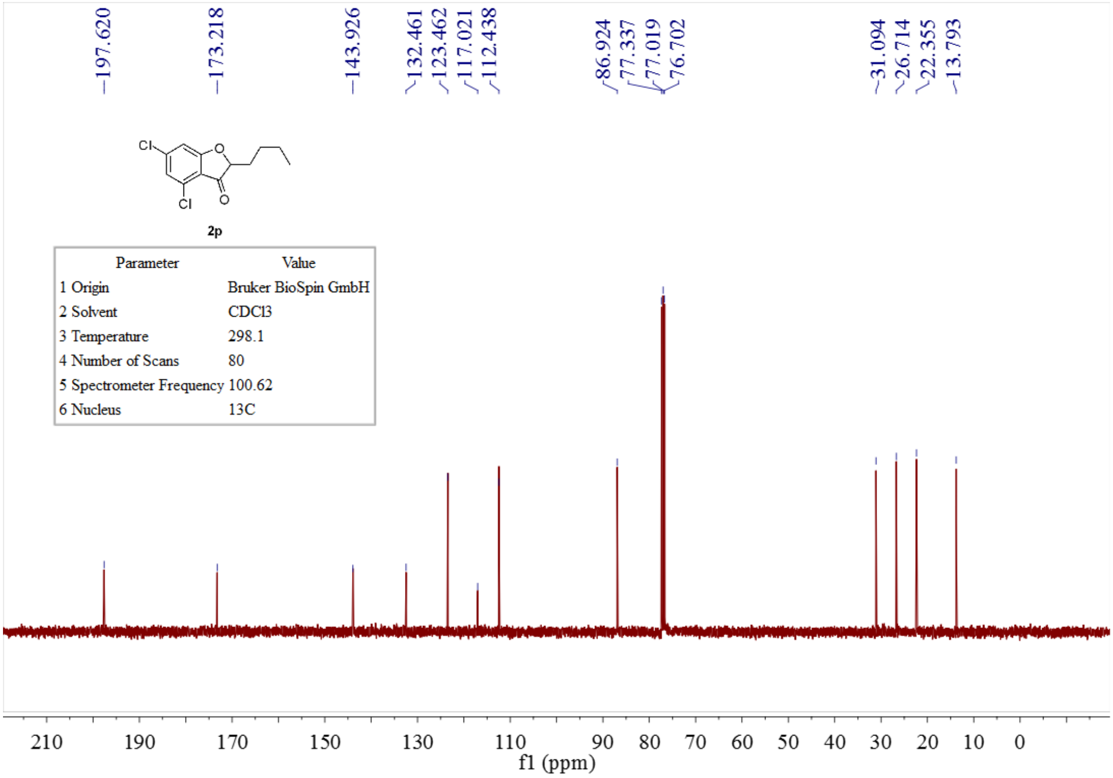

Supplementary Figure 123. **^{13}C NMR (100 MHz, $CDCl_3$) spectra for compound 2p**

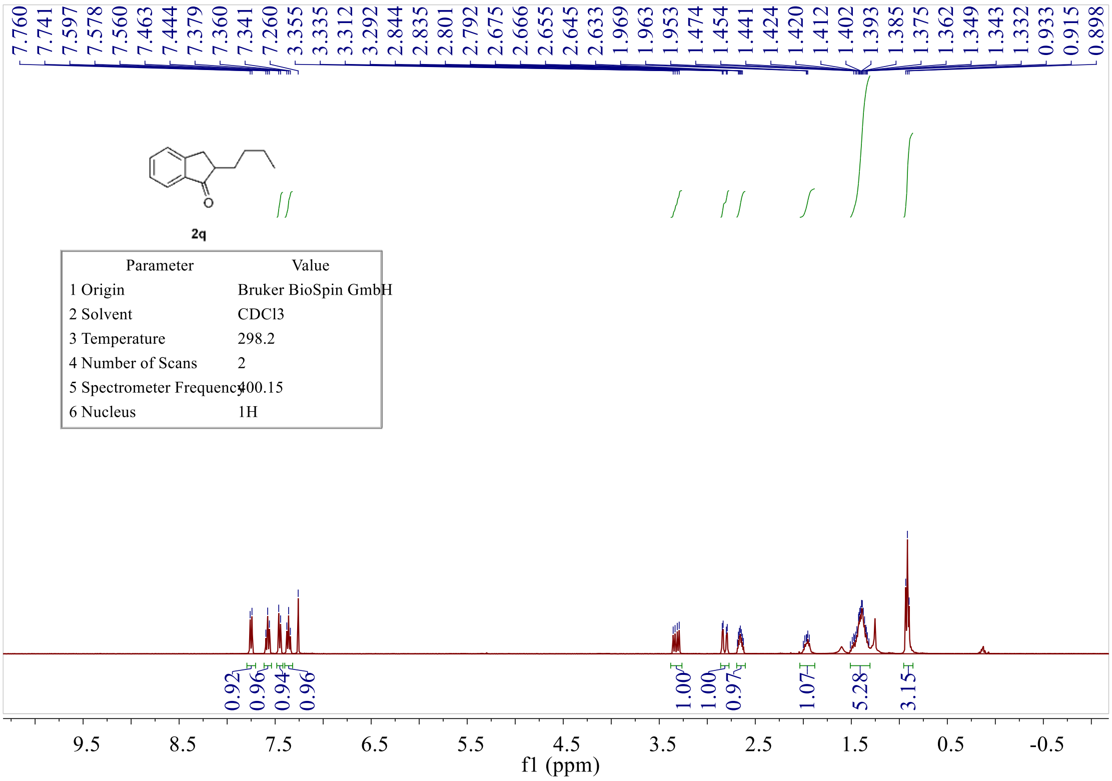

Supplementary Figure 124. ^{1}**H NMR (400 MHz, CDCl**$_3$**) spectra for compound 2q**

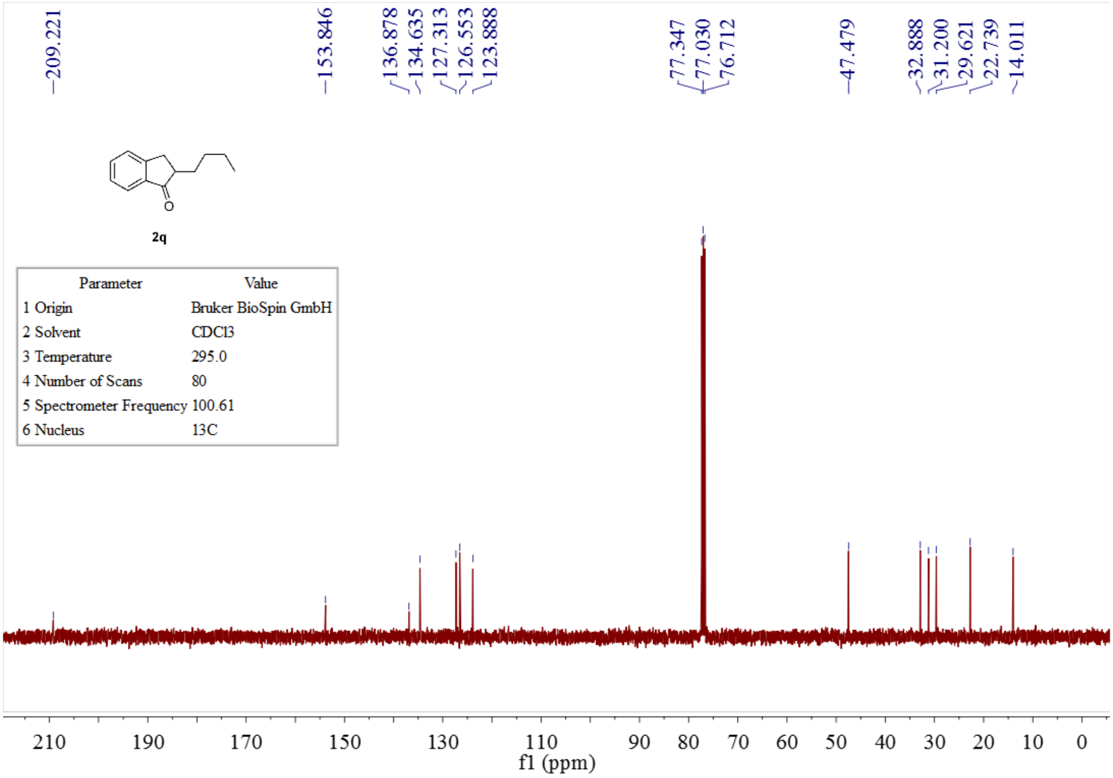

Supplementary Figure 125. ^{13}**C NMR (100 MHz, CDCl**$_3$**) spectra for compound 2q**

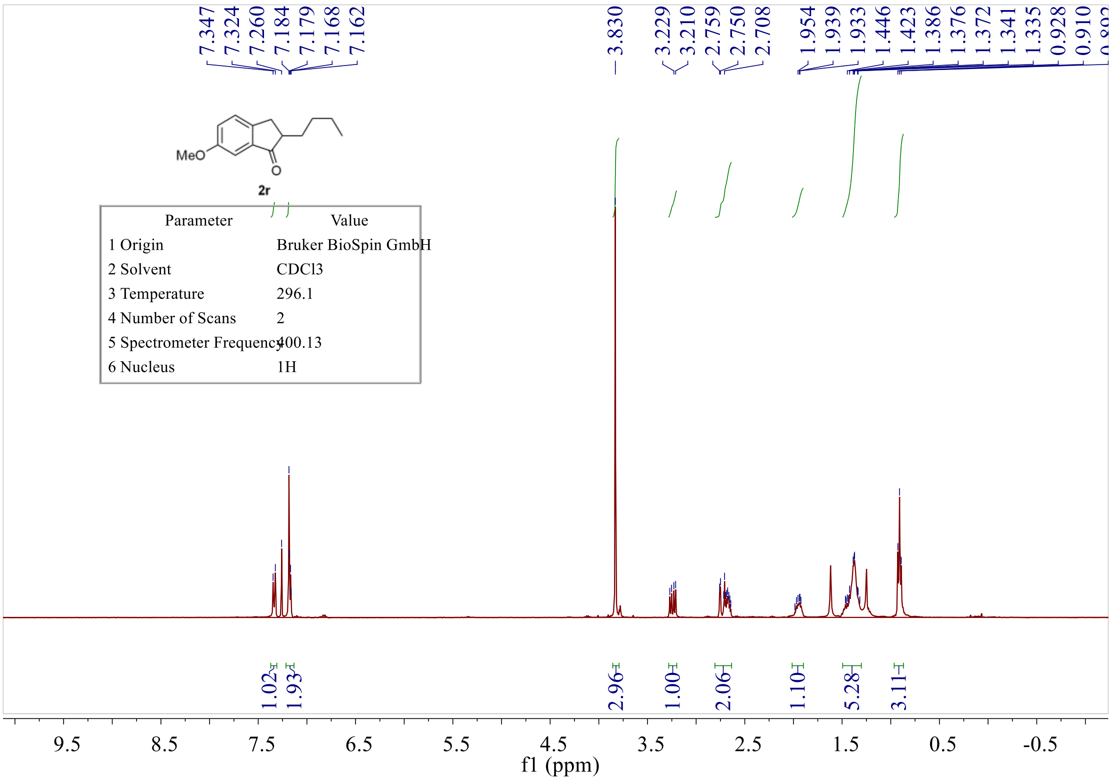

Supplementary Figure 126. ^{1}H NMR (400 MHz, $CDCl_3$) spectra for compound 2r

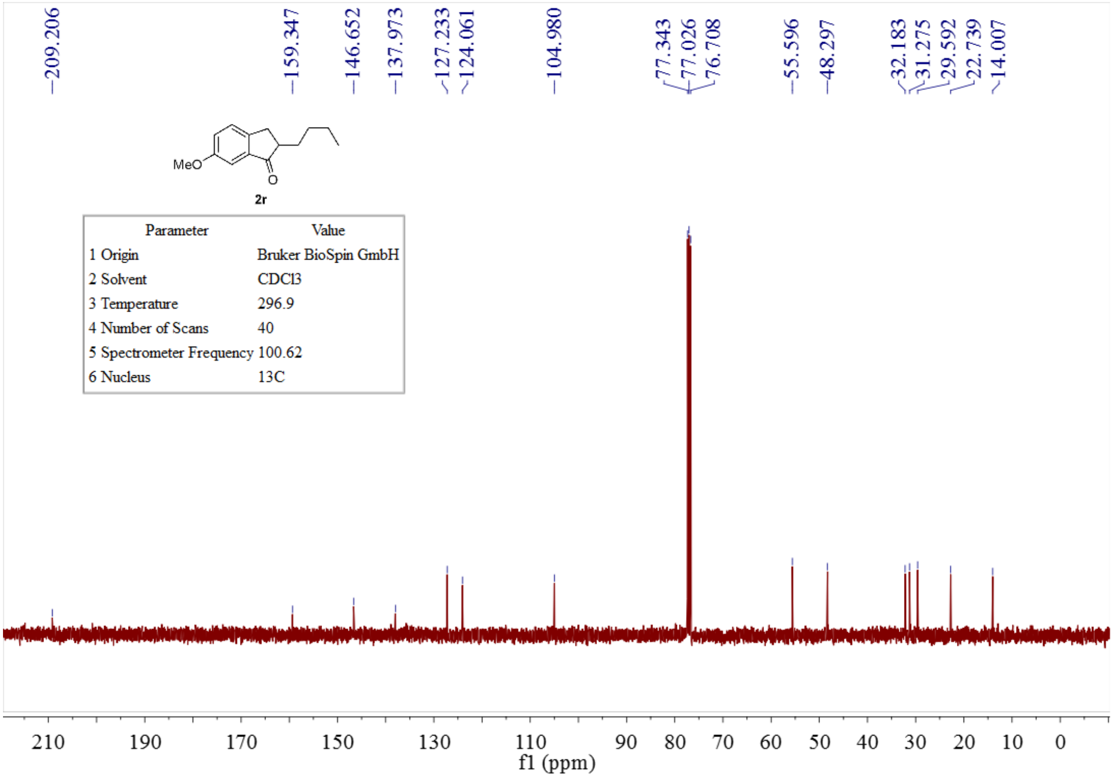

Supplementary Figure 127. ^{13}C NMR (100 MHz, $CDCl_3$) spectra for compound 2r

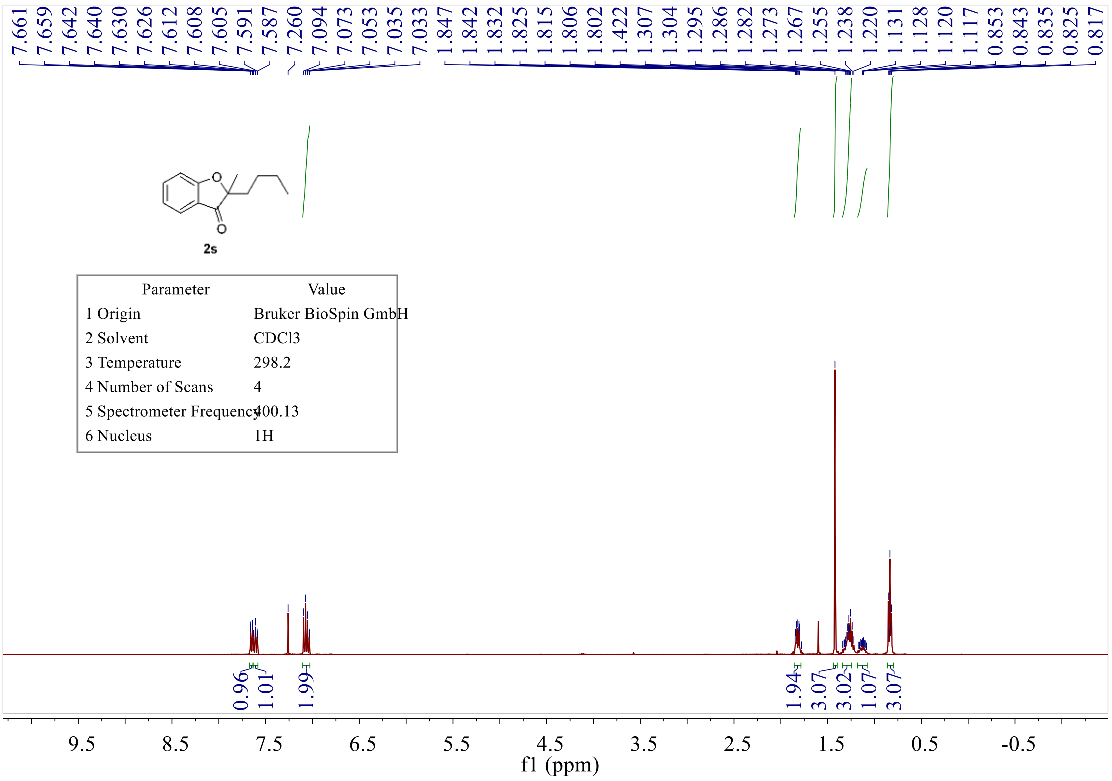

Supplementary Figure 128. ^{1}**H NMR (400 MHz, CDCl$_3$) spectra for compound 2s**

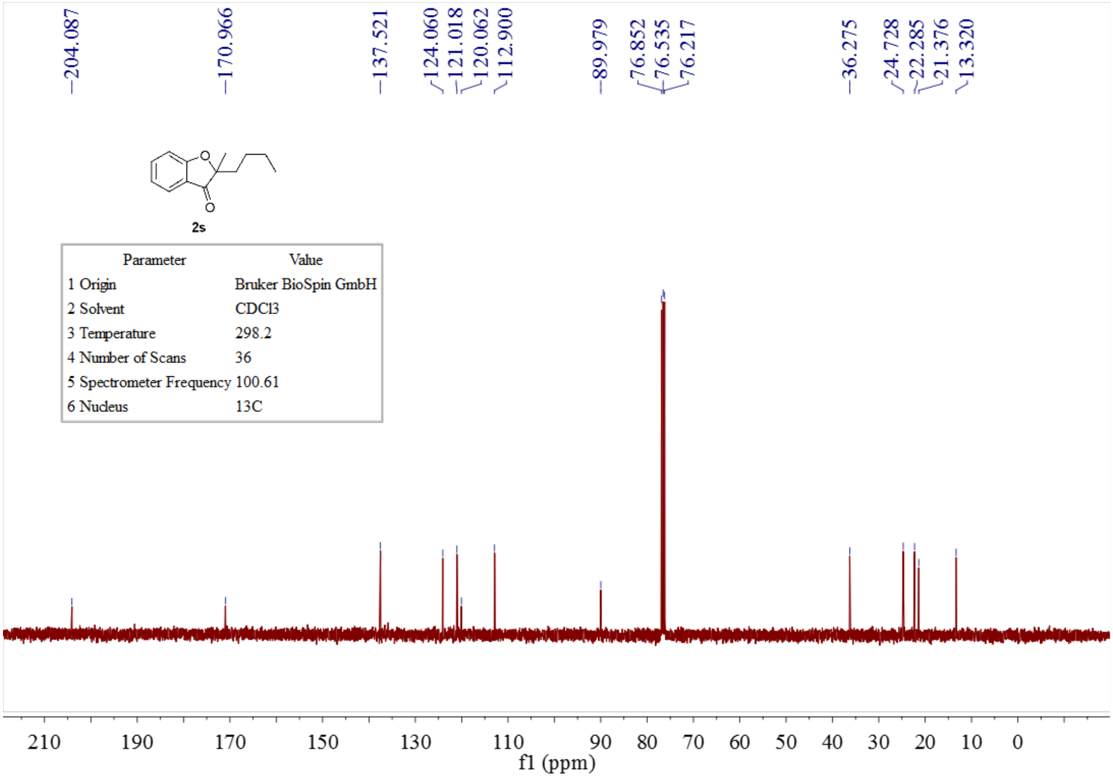

Supplementary Figure 129. ^{13}**C NMR (100 MHz, CDCl$_3$) spectra for compound 2s**

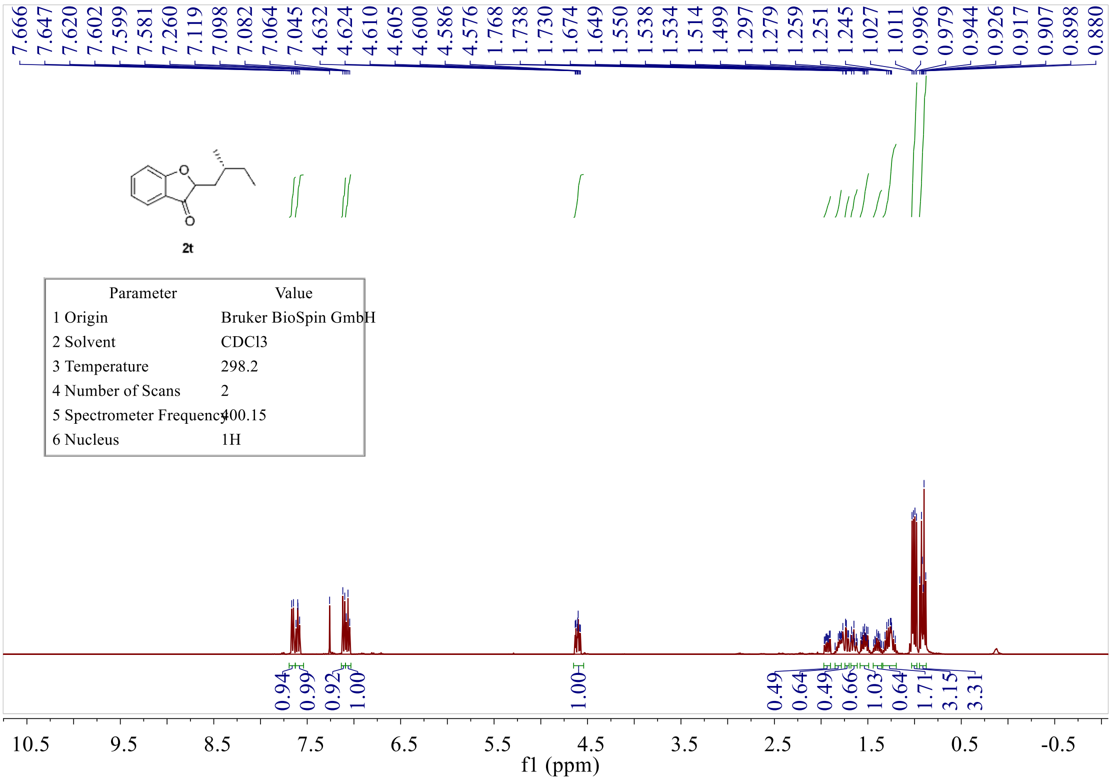

Supplementary Figure 130. **^{1}H NMR (400 MHz, $CDCl_3$) spectra for compound 2t**

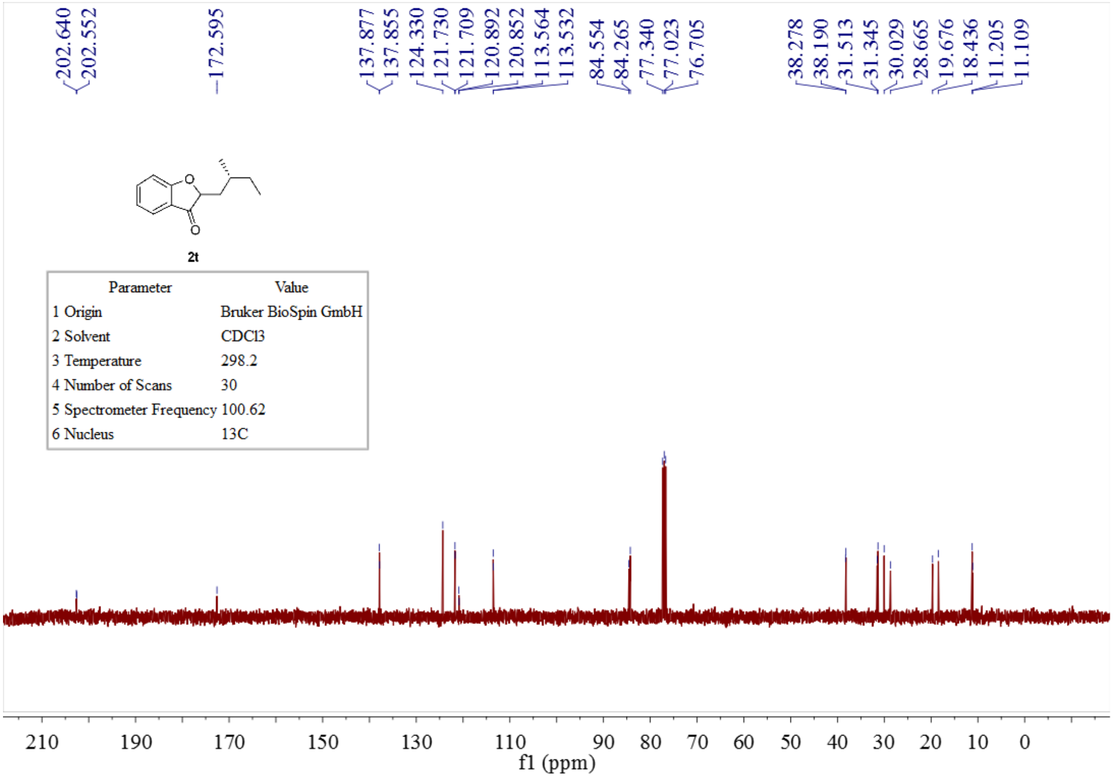

Supplementary Figure 131. **^{13}C NMR (100 MHz, $CDCl_3$) spectra for compound 2t**

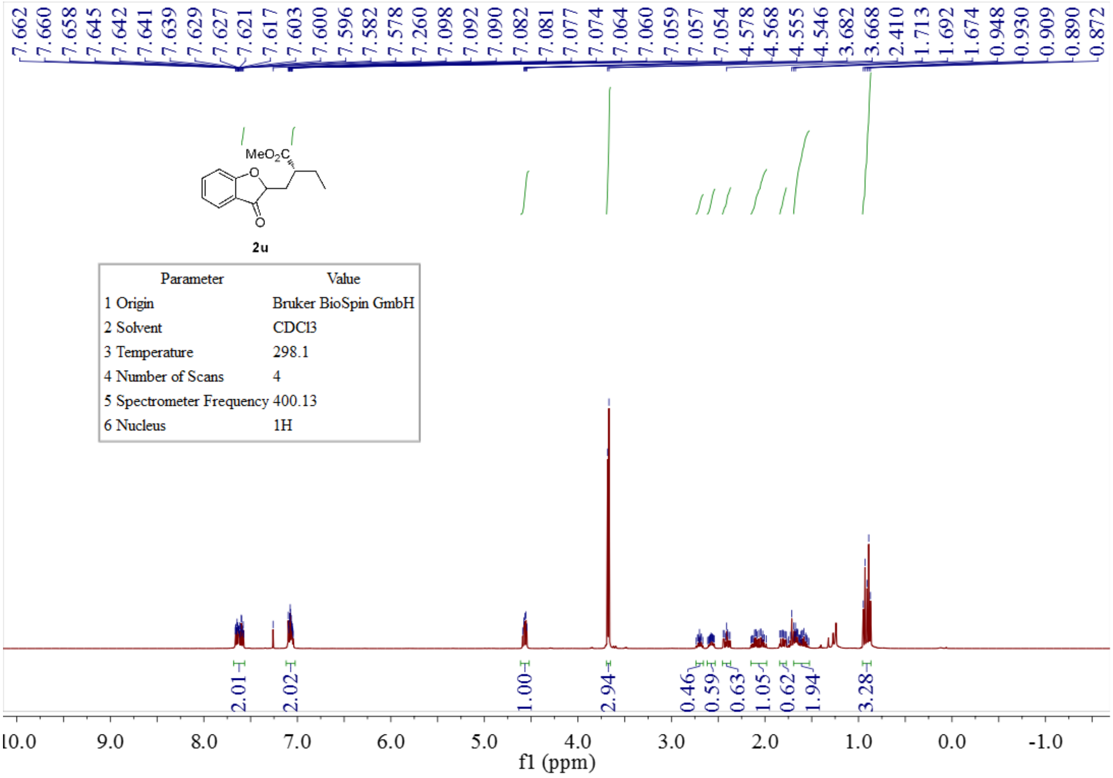

Supplementary Figure 132. ^{1}H **NMR (400 MHz,** $CDCl_3$**) spectra for compound 2u**

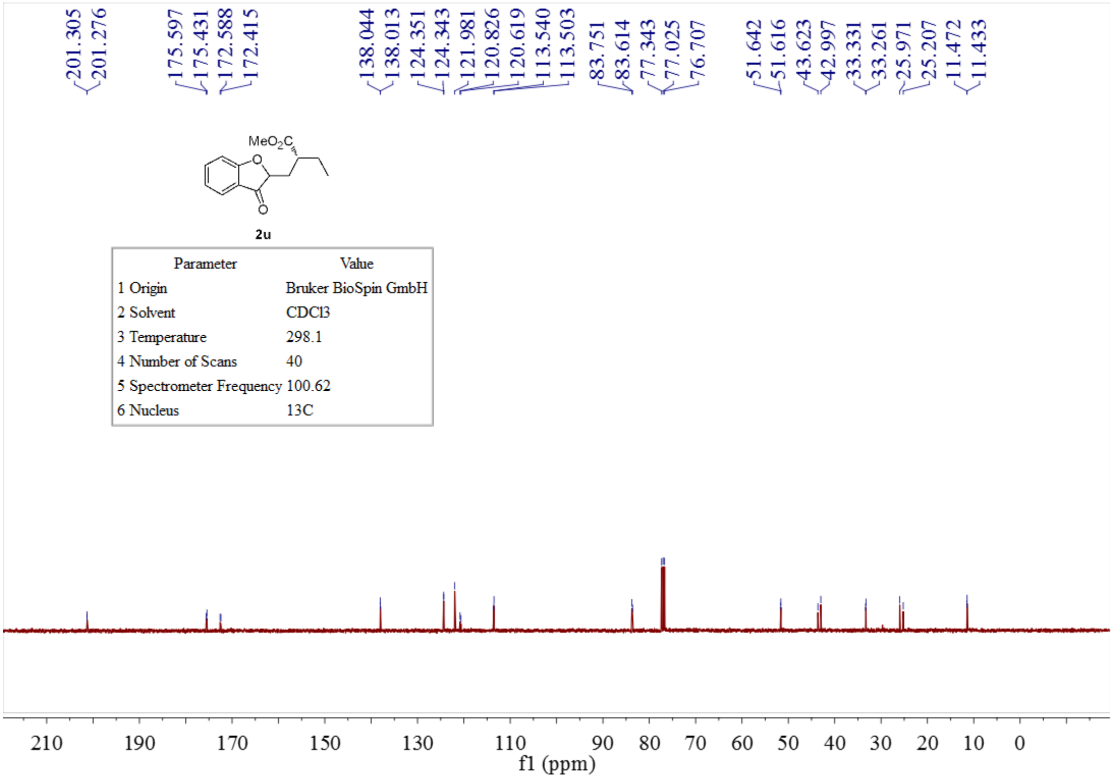

Supplementary Figure 133. ^{13}C **NMR (100 MHz,** $CDCl_3$**) spectra for compound 2u**

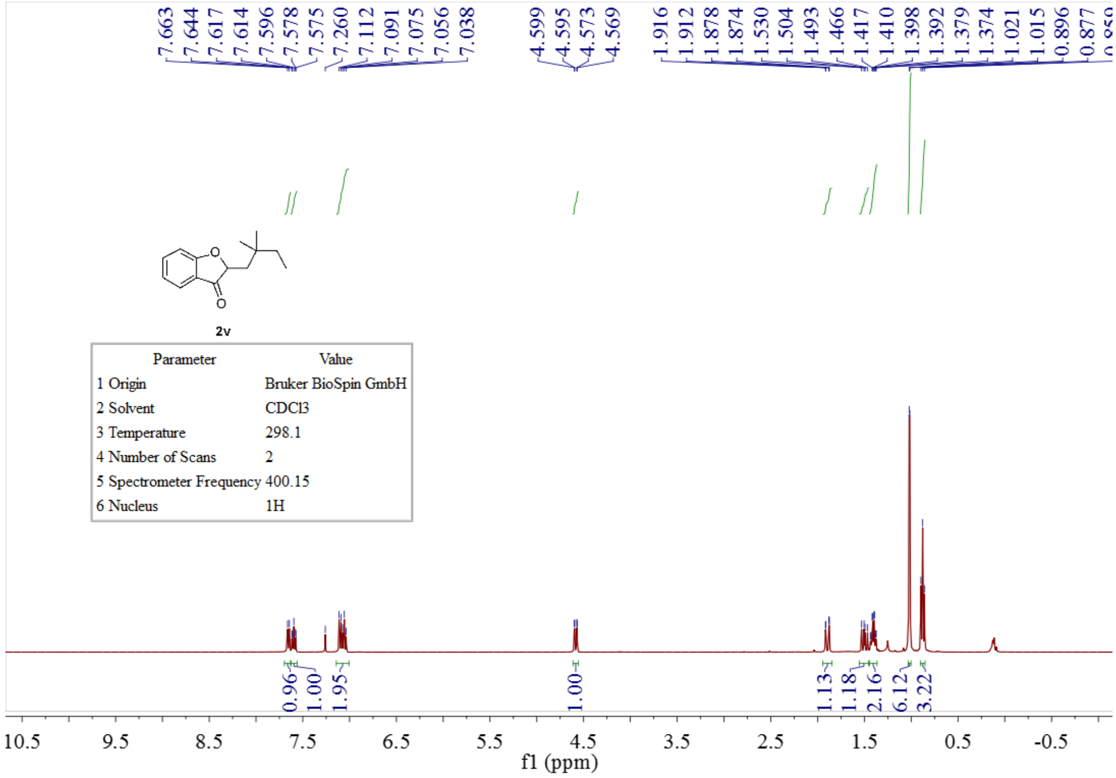

Supplementary Figure 134. ^{1}**H NMR (400 MHz, CDCl**$_3$**) spectra for compound 2v**

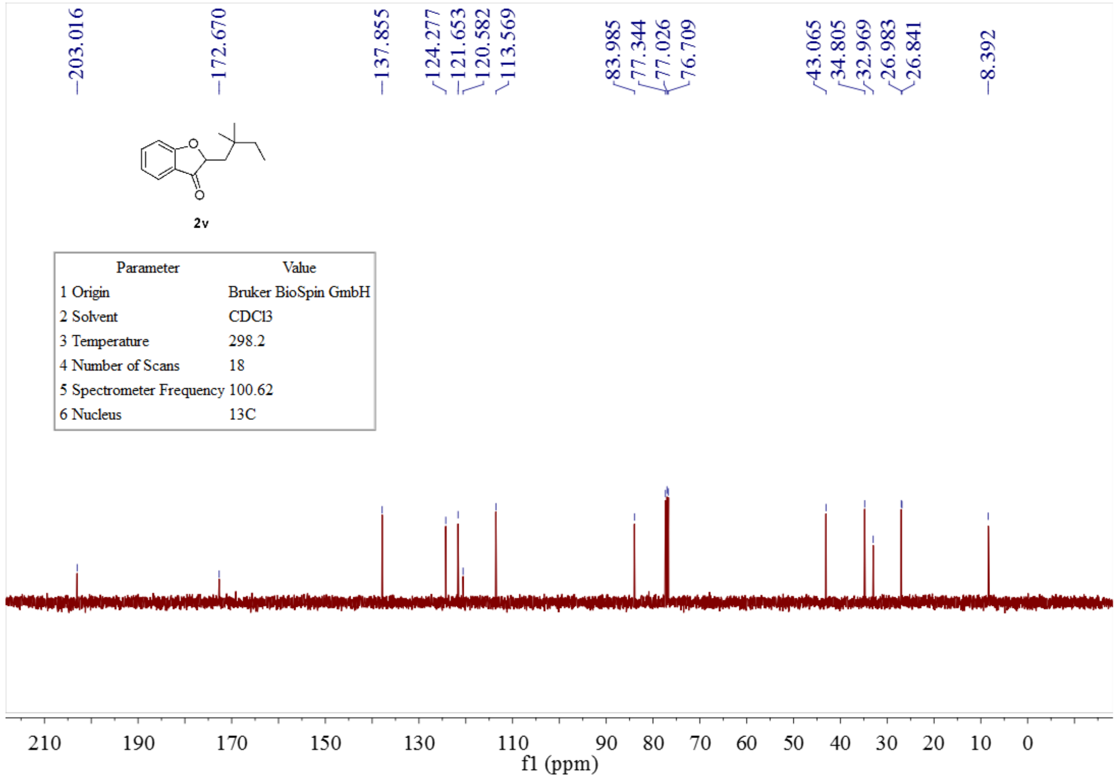

Supplementary Figure 135. ^{13}**C NMR (100 MHz, CDCl**$_3$**) spectra for compound 2v**

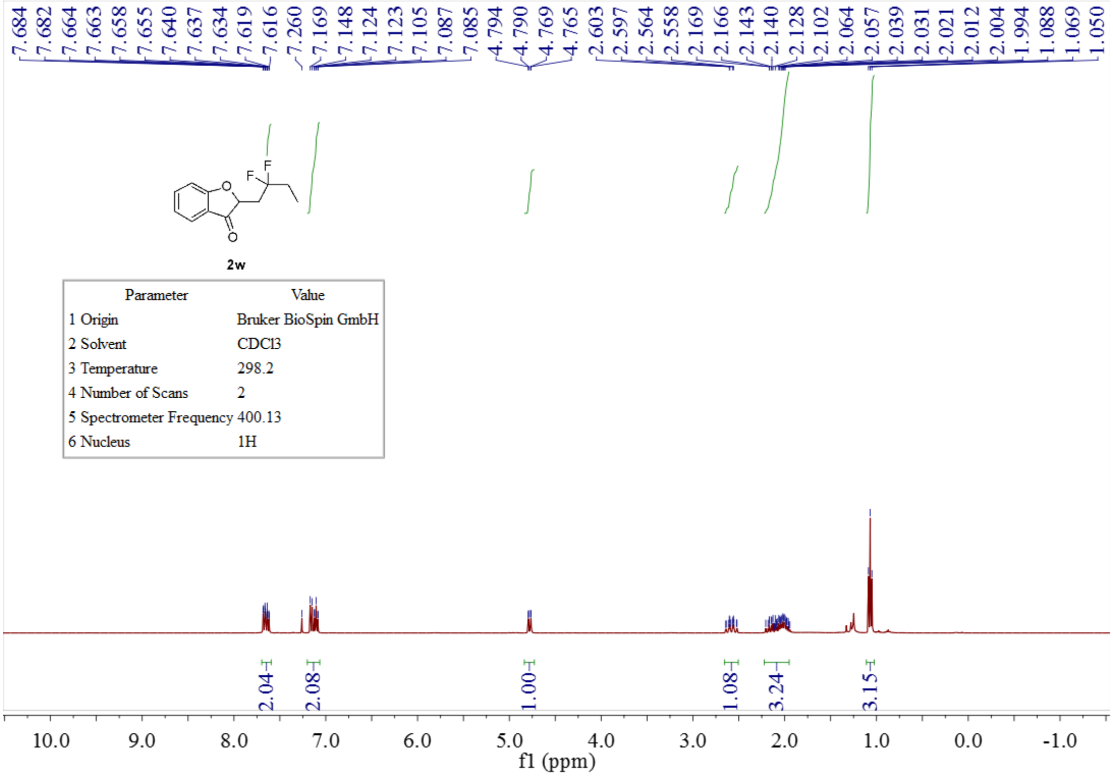

Supplementary Figure 136. ^{1}**H NMR (400 MHz,** $CDCl_3$**) spectra for compound 2w**

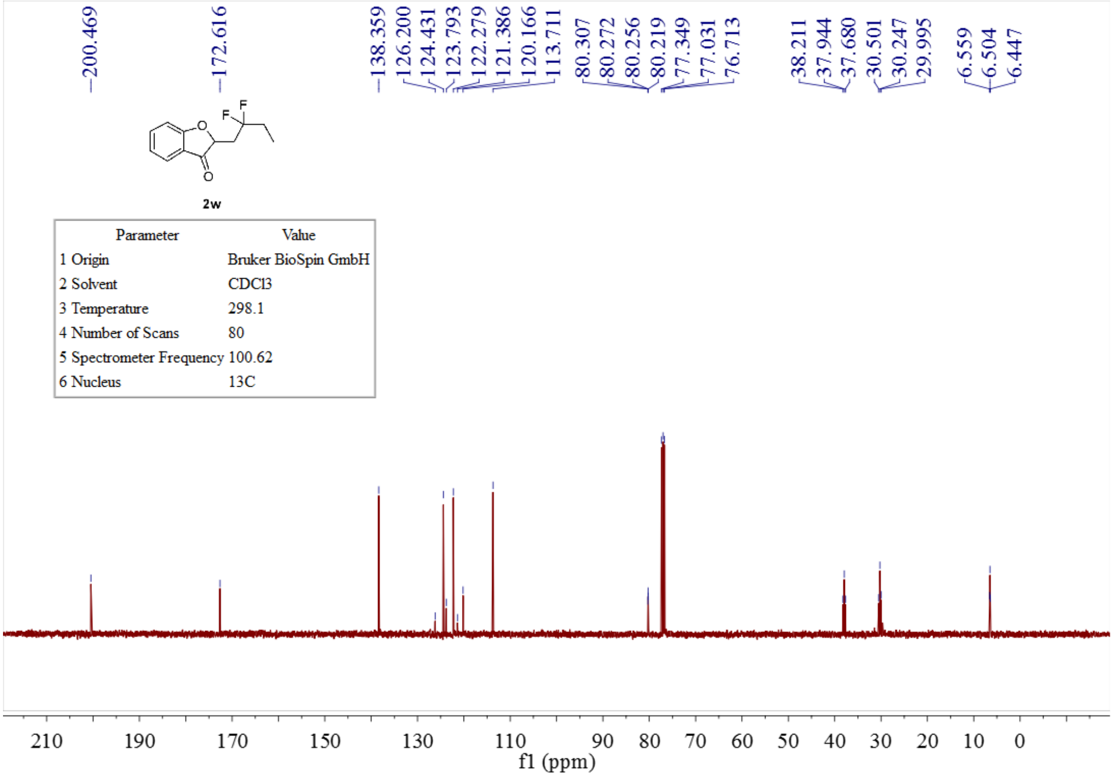

Supplementary Figure 137. ^{13}**C NMR (100 MHz,** $CDCl_3$**) spectra for compound 2w**

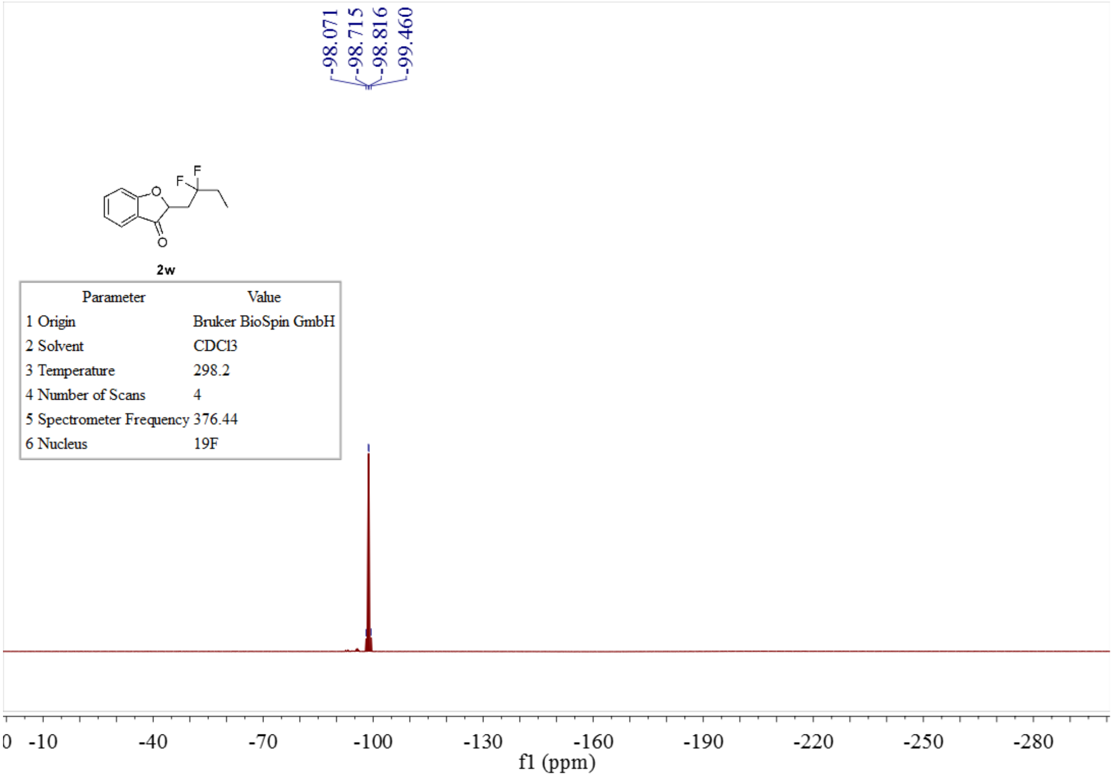

Parameter	Value
1 Origin	Bruker BioSpin GmbH
2 Solvent	CDCl3
3 Temperature	298.2
4 Number of Scans	4
5 Spectrometer Frequency	376.44
6 Nucleus	19F

Supplementary Figure 138. **^{19}F NMR (376 MHz, $CDCl_3$) spectra for compound 2w**

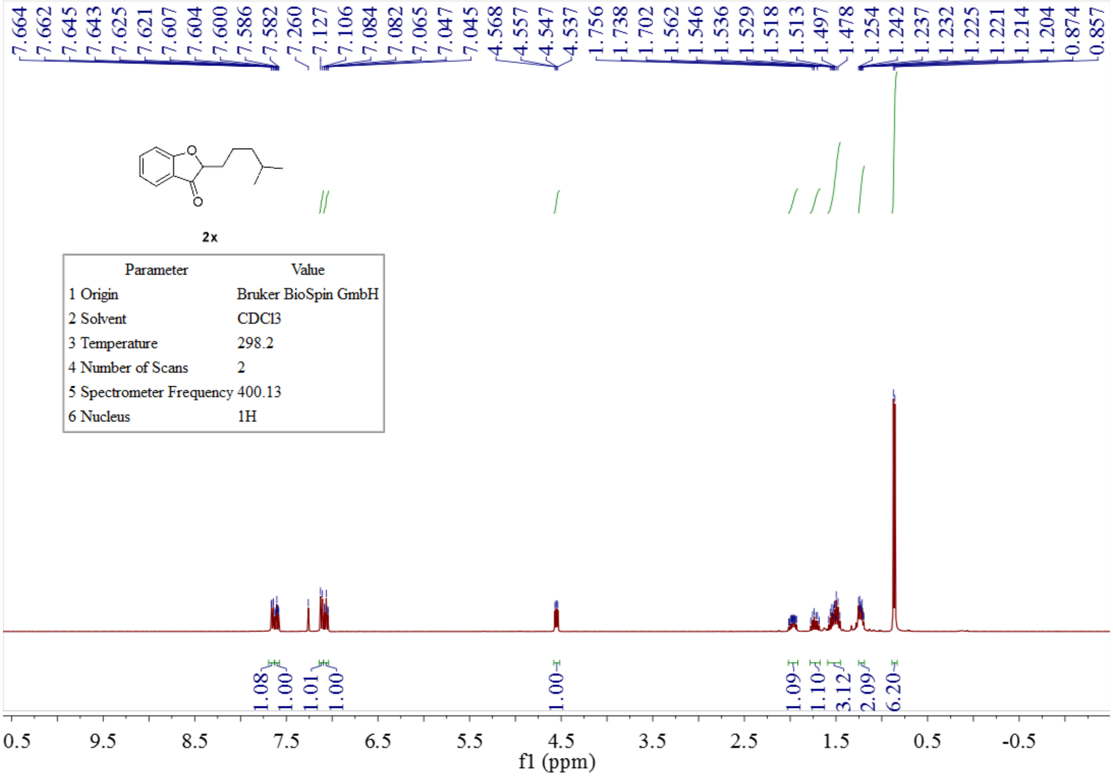

Parameter	Value
1 Origin	Bruker BioSpin GmbH
2 Solvent	CDCl3
3 Temperature	298.2
4 Number of Scans	2
5 Spectrometer Frequency	400.13
6 Nucleus	1H

Supplementary Figure 139. **^{1}H NMR (400 MHz, $CDCl_3$) spectra for compound 2x**

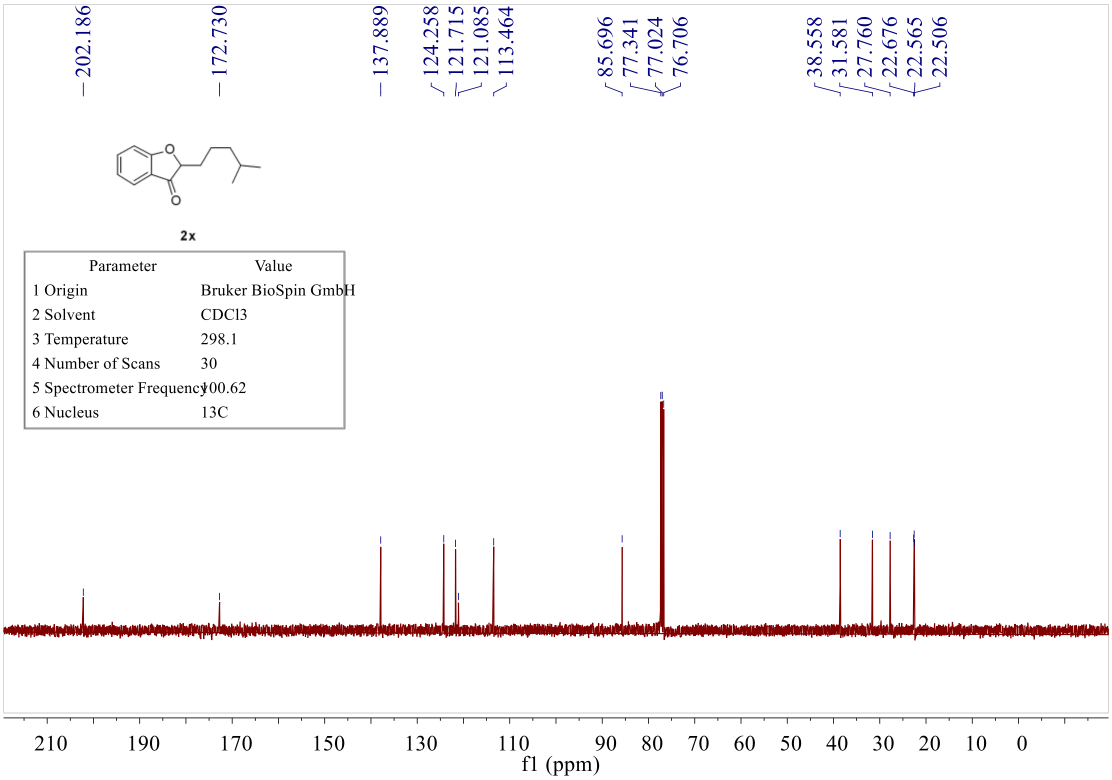

Supplementary Figure 140. ^{13}**C NMR (100 MHz, CDCl$_3$) spectra for compound 2x**

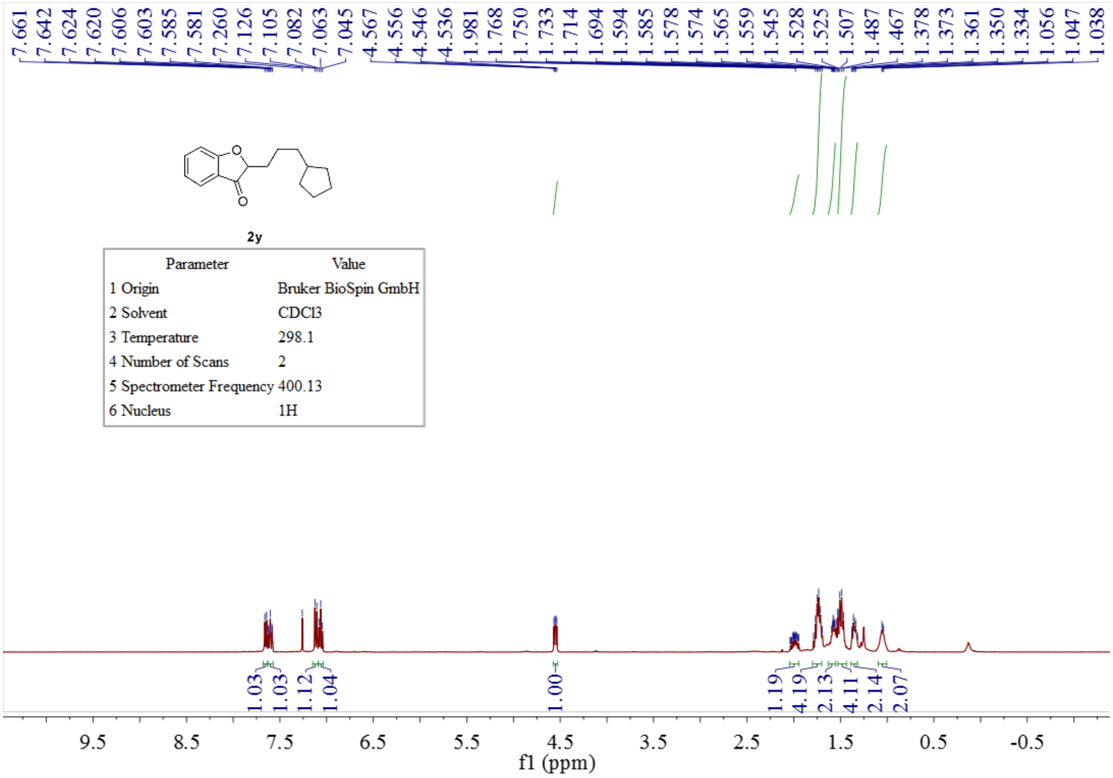

Supplementary Figure 141. ^{1}**H NMR (400 MHz, CDCl$_3$) spectra for compound 2y**

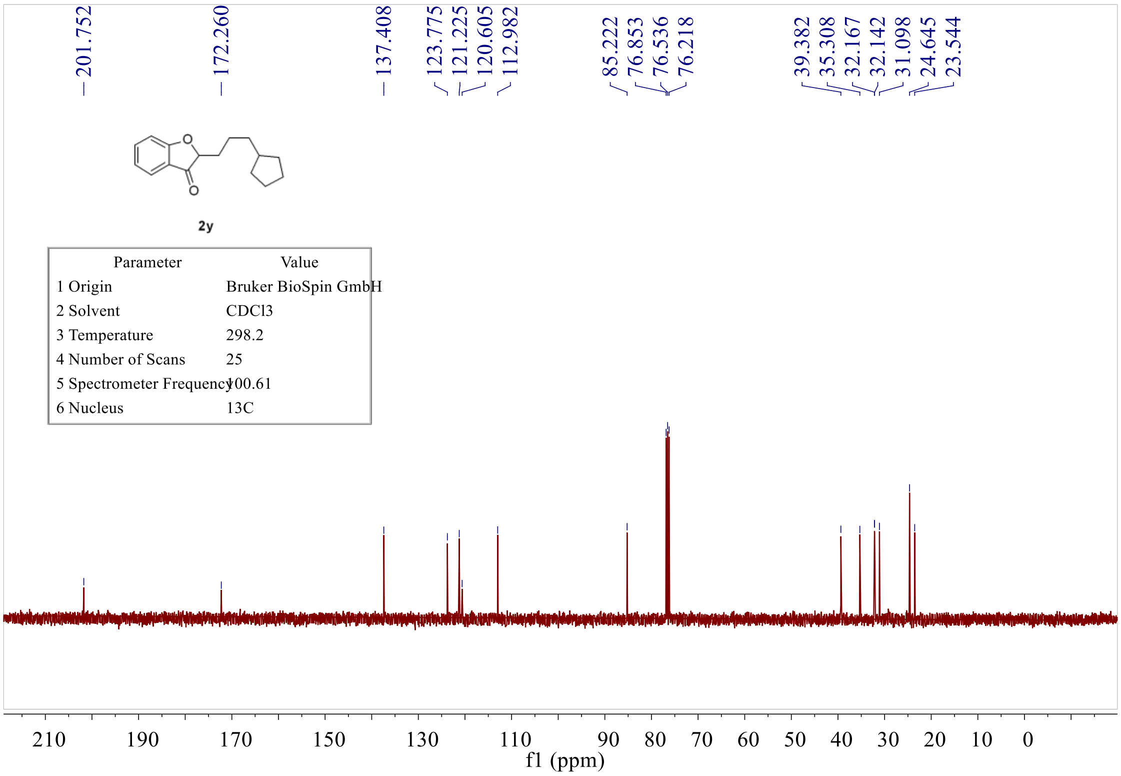

Supplementary Figure 142. ^{13}C NMR (100 MHz, $CDCl_3$) spectra for compound 2y

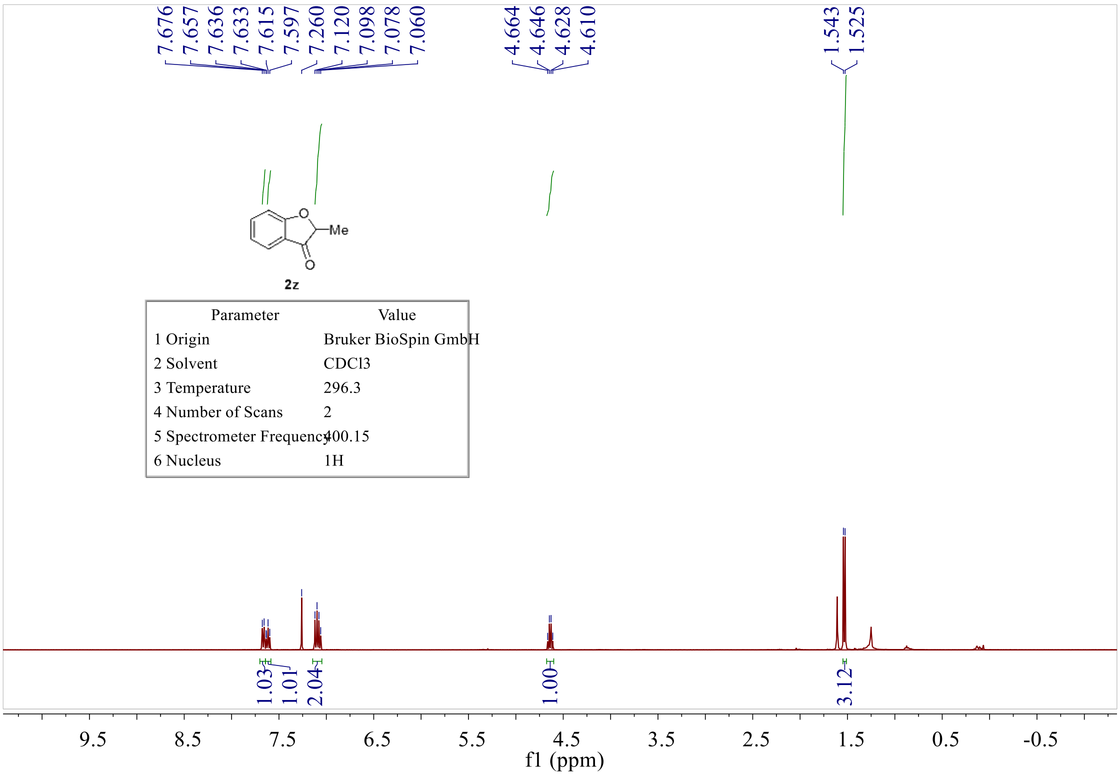

Supplementary Figure 143. ^{1}H NMR (400 MHz, $CDCl_3$) spectra for compound 2z

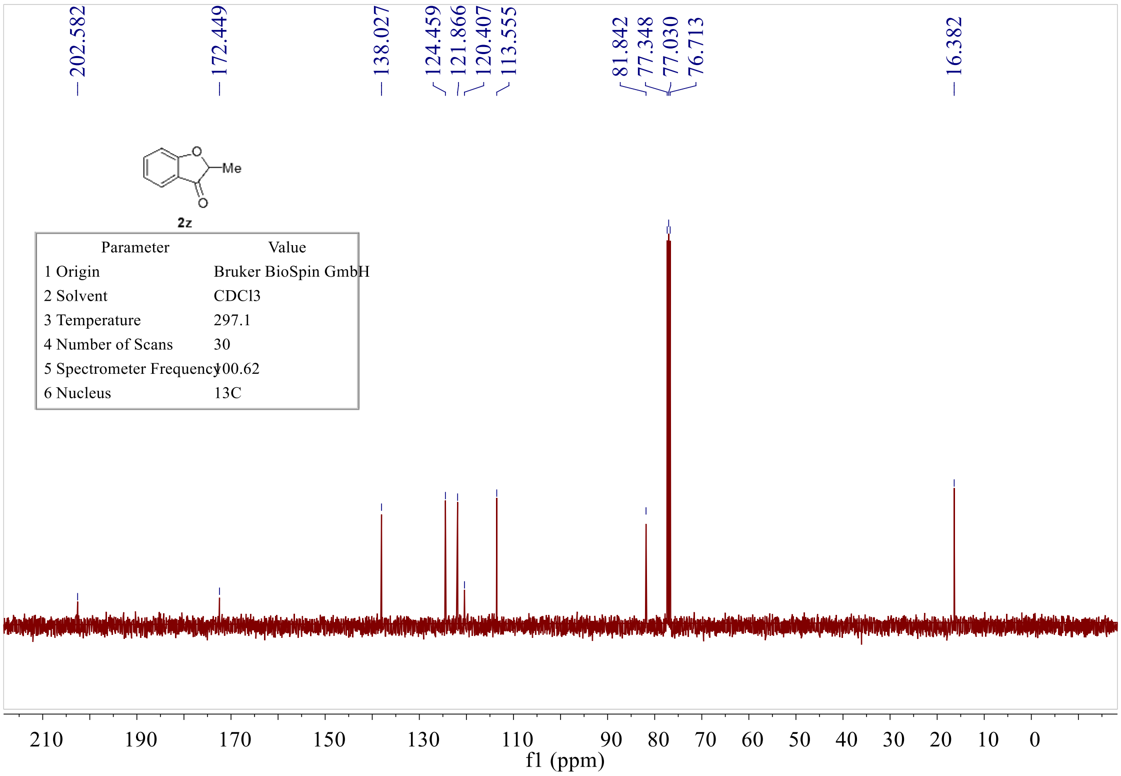

Supplementary Figure 144. ^{13}**C NMR (100 MHz, CDCl**$_3$**) spectra for compound 2z**

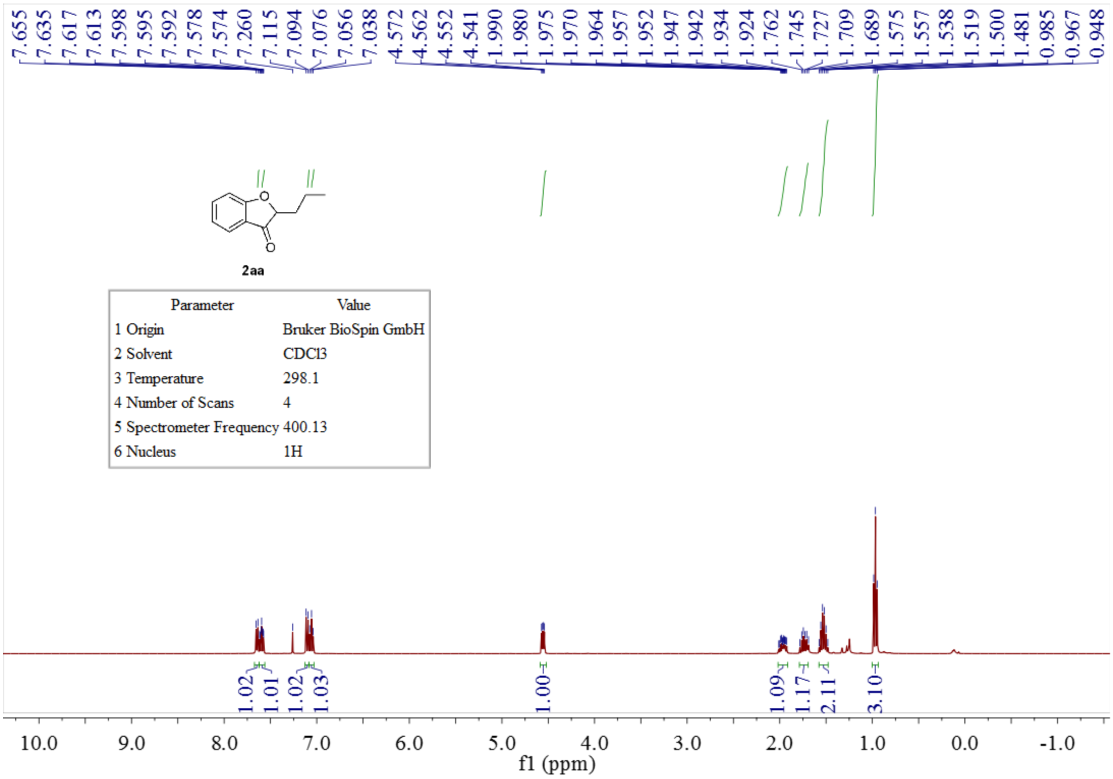

Supplementary Figure 145. ^{1}**H NMR (400 MHz, CDCl**$_3$**) spectra for compound 2aa**

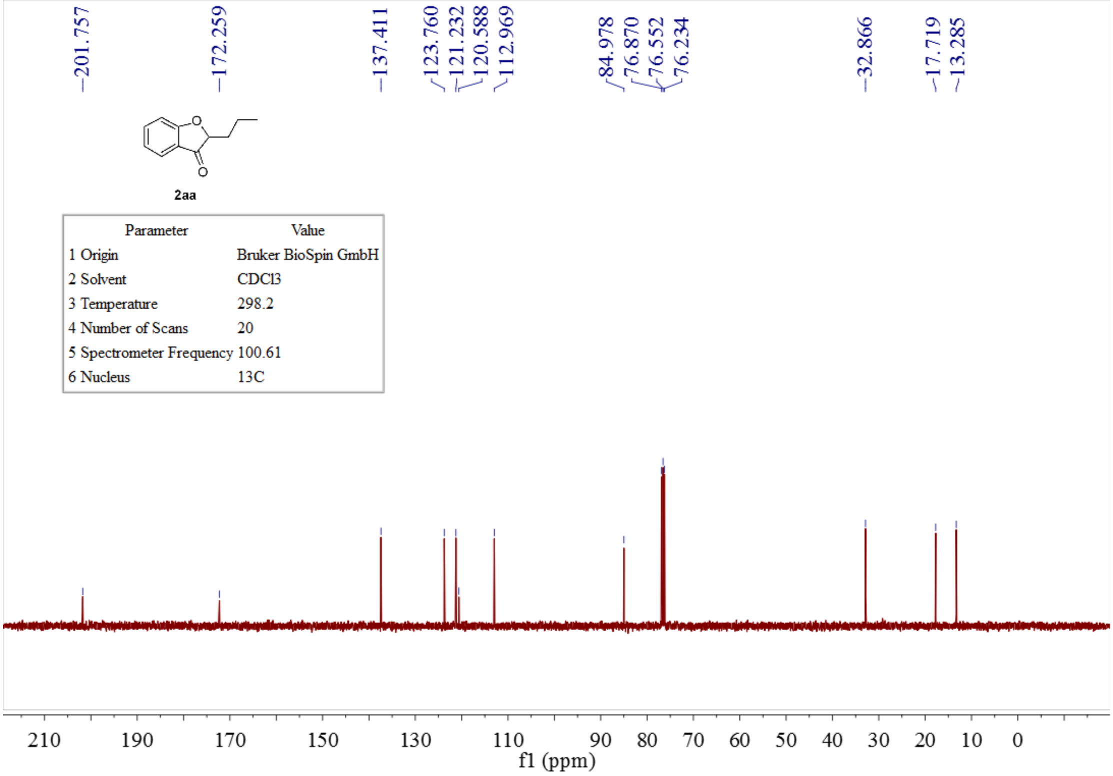

Supplementary Figure 146. ^{13}**C NMR (100 MHz, CDCl**$_3$**) spectra for compound 2aa**

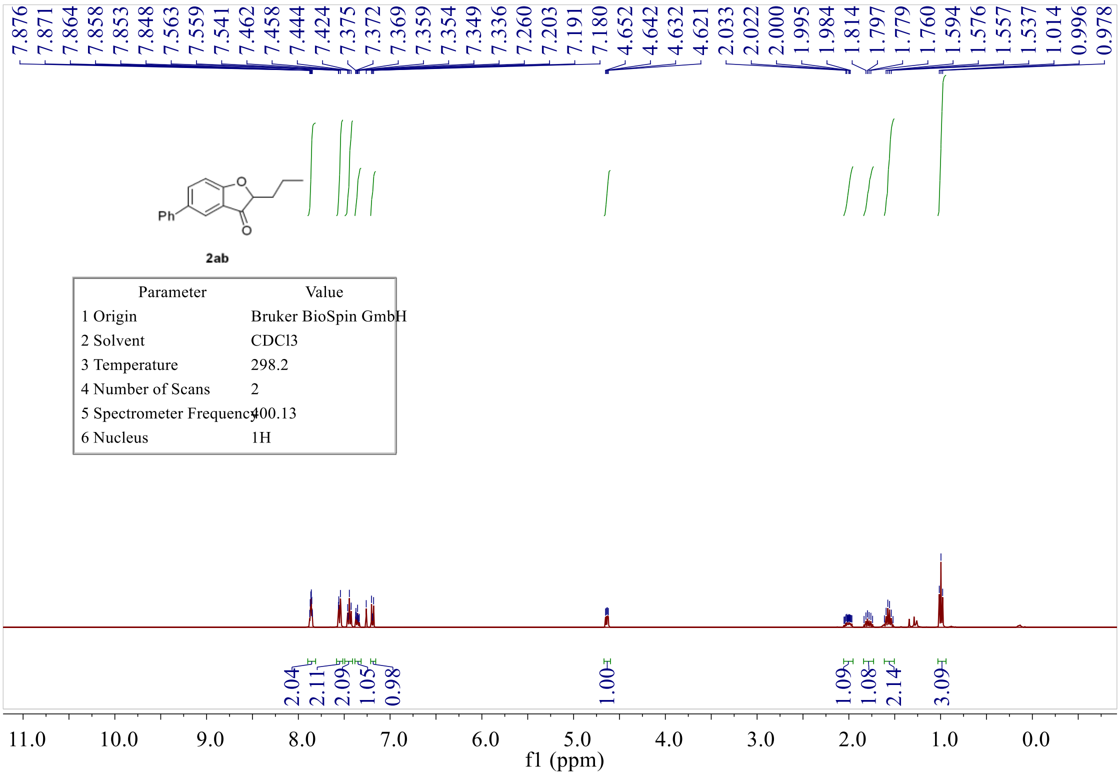

Supplementary Figure 147. ^{1}**H NMR (400 MHz, CDCl**$_3$**) spectra for compound 2ab**

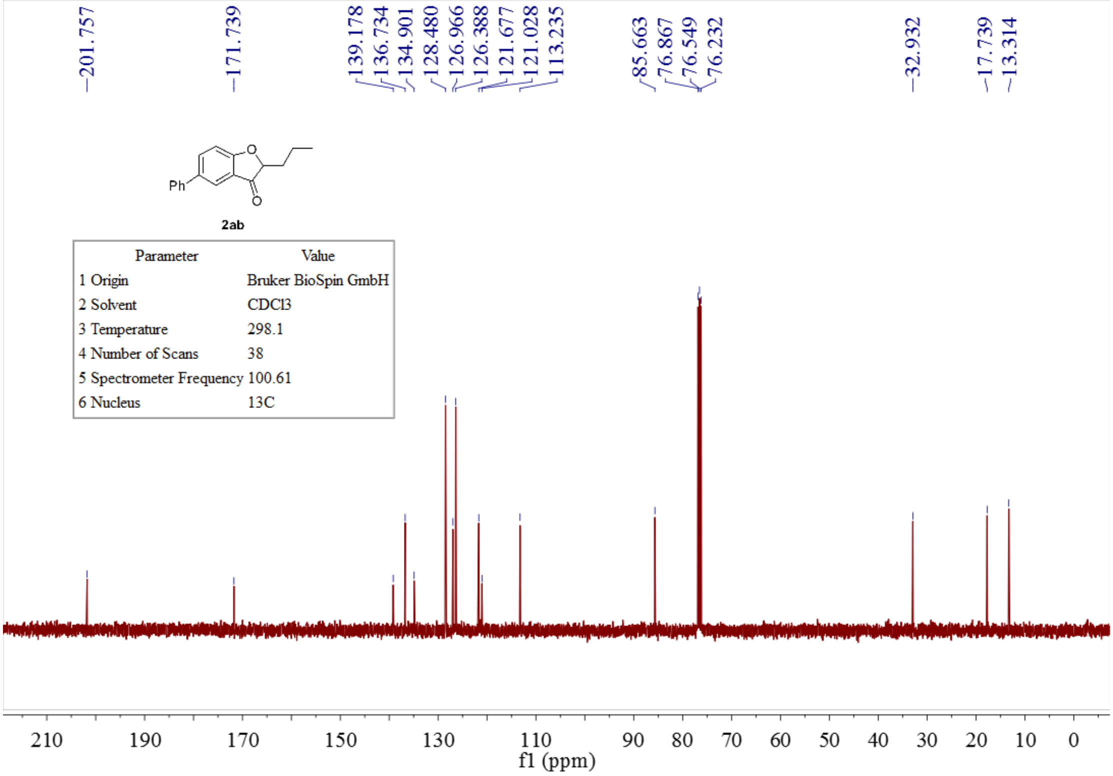

Supplementary Figure 148. ^{13}**C NMR (100 MHz, CDCl$_3$) spectra for compound 2ab**

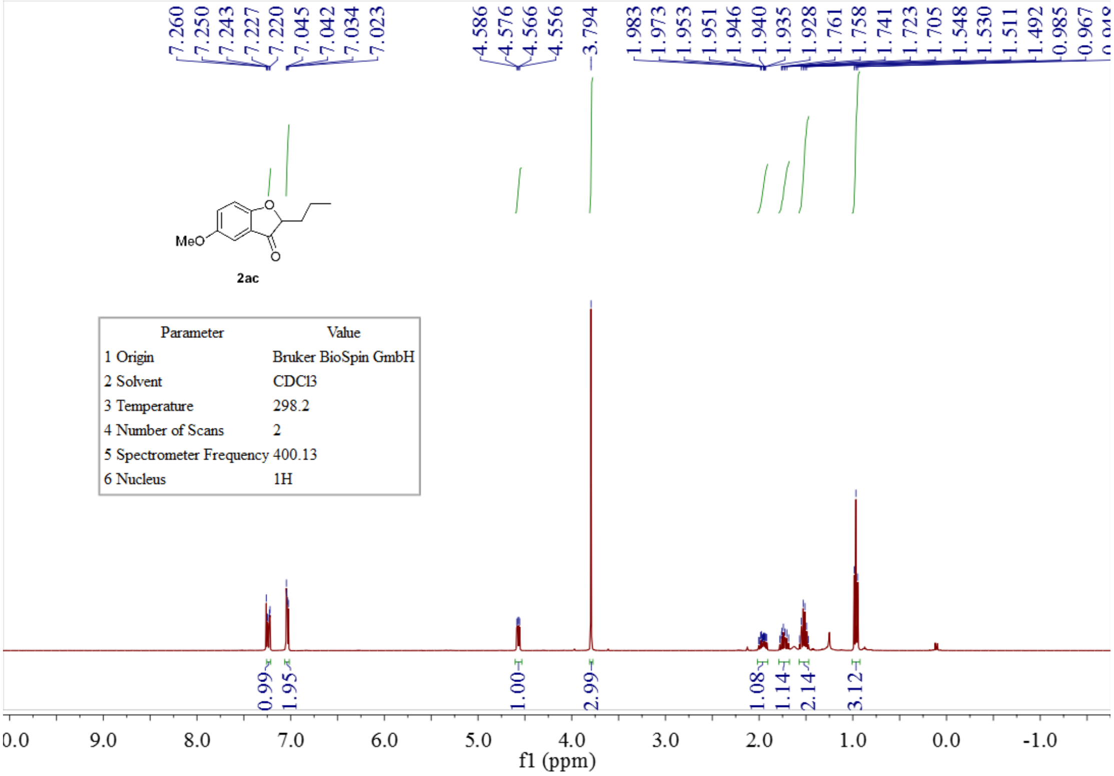

Supplementary Figure 149. ^{1}**H NMR (400 MHz, CDCl$_3$) spectra for compound 2ac**

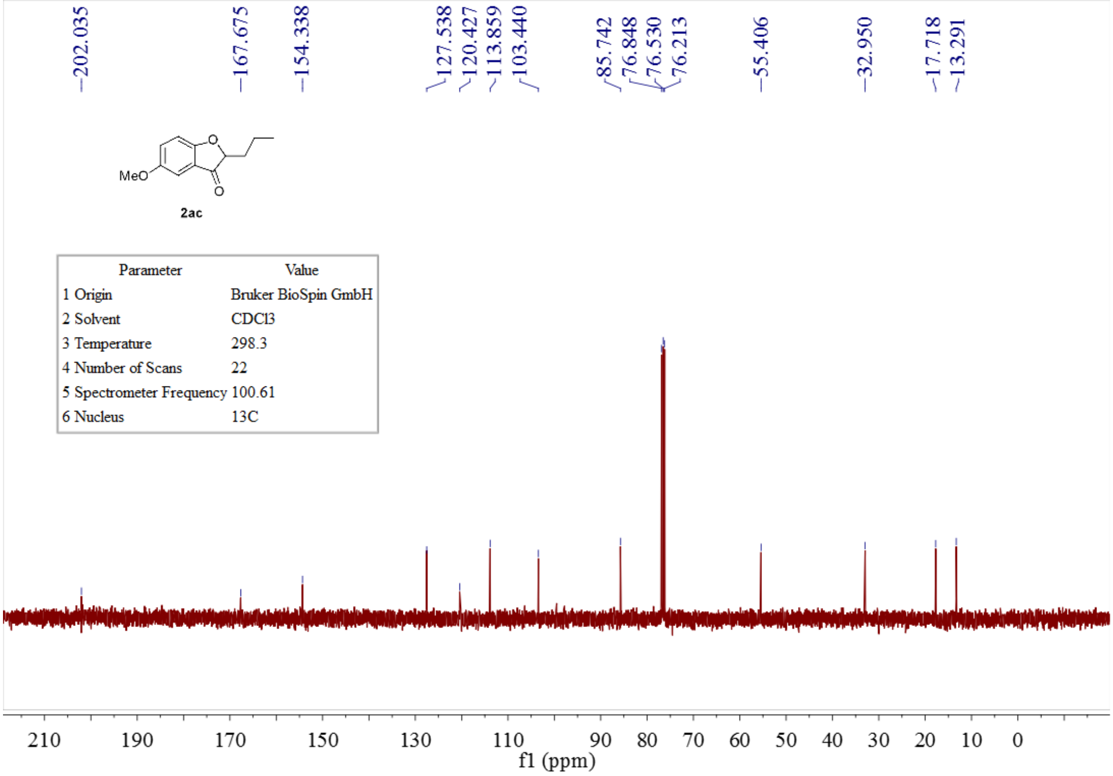

Supplementary Figure 150. ^{13}**C NMR (100 MHz, CDCl**$_3$**) spectra for compound 2ac**

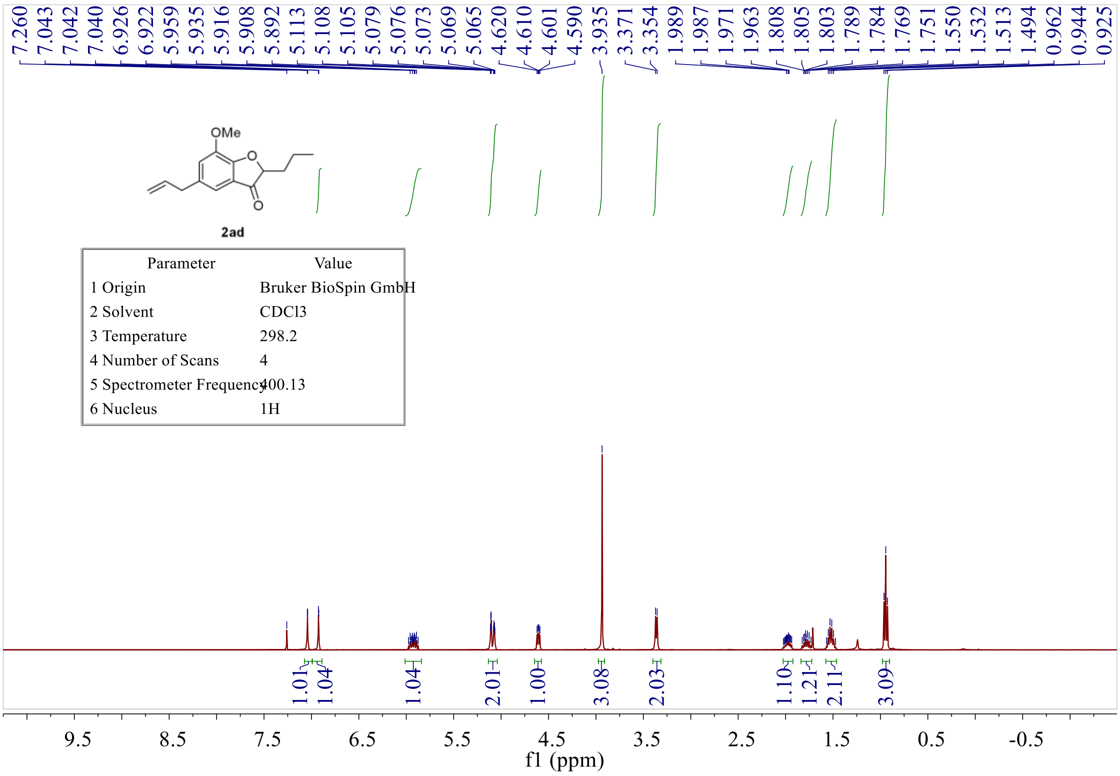

Supplementary Figure 151. ^{1}**H NMR (400 MHz, CDCl**$_3$**) spectra for compound 2ad**

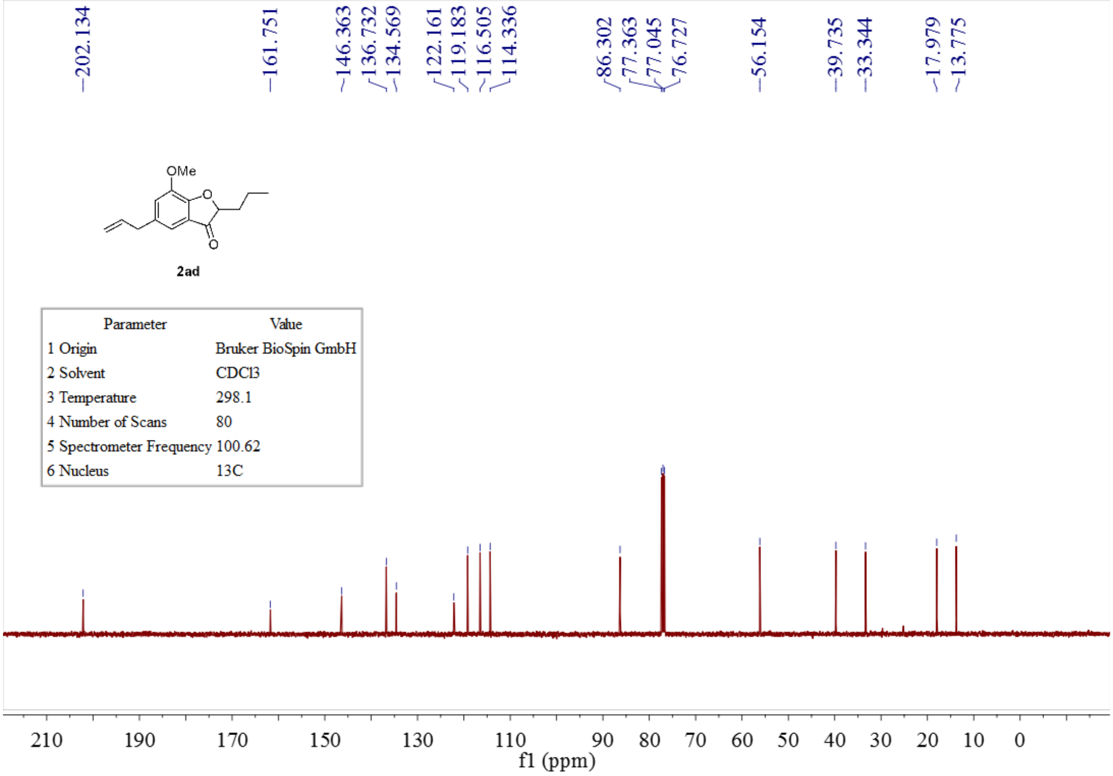

Supplementary Figure 152. ^{13}**C NMR (100 MHz,** $CDCl_3$**) spectra for compound 2ad**

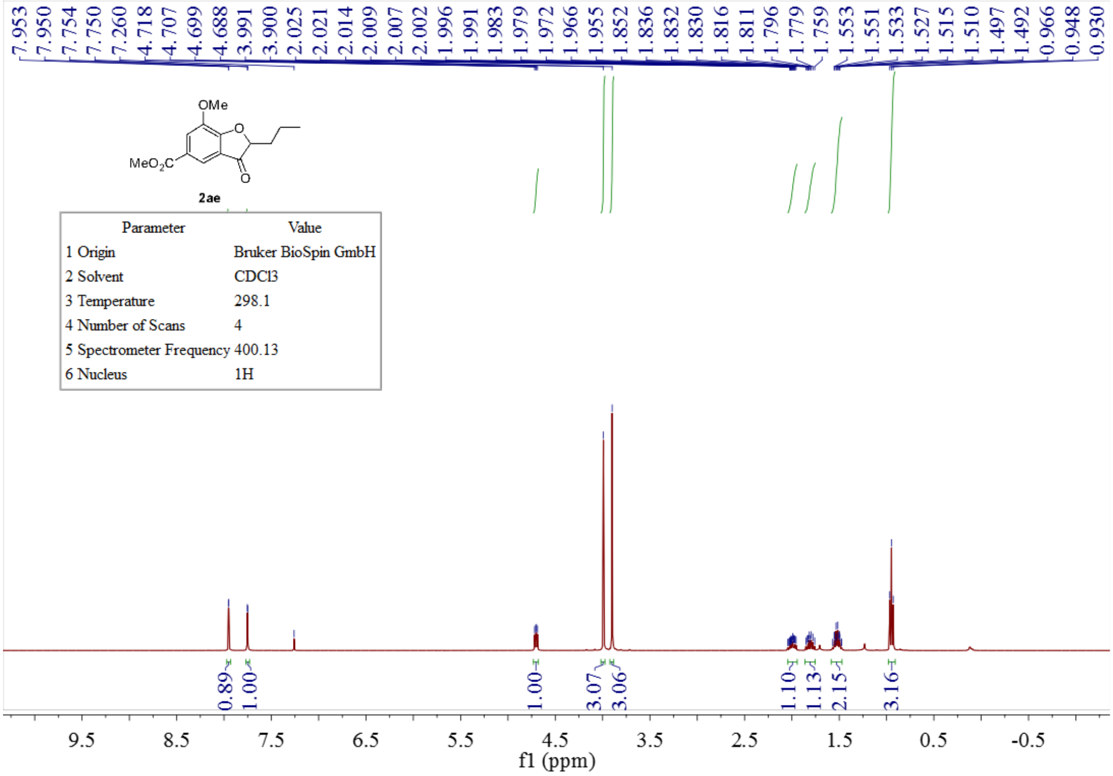

Supplementary Figure 153. ^{1}**H NMR (400 MHz,** $CDCl_3$**) spectra for compound 2ae**

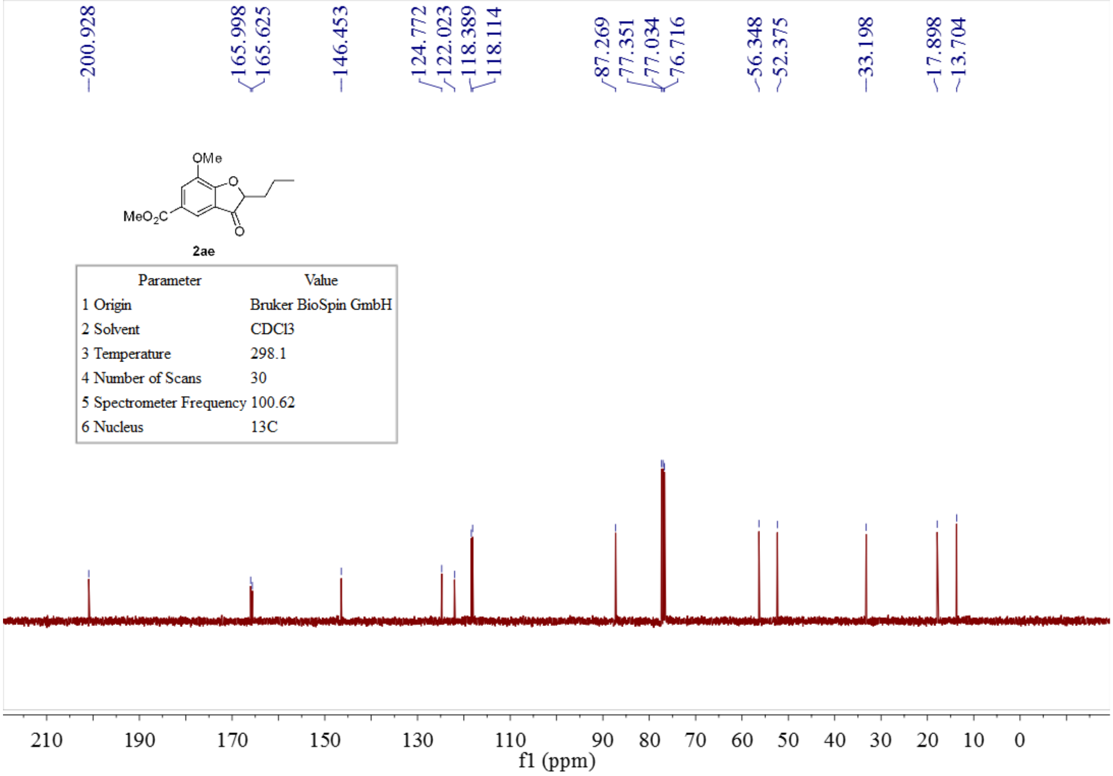

Supplementary Figure 154. **^{13}C NMR (100 MHz, CDCl$_3$) spectra for compound 2ae**

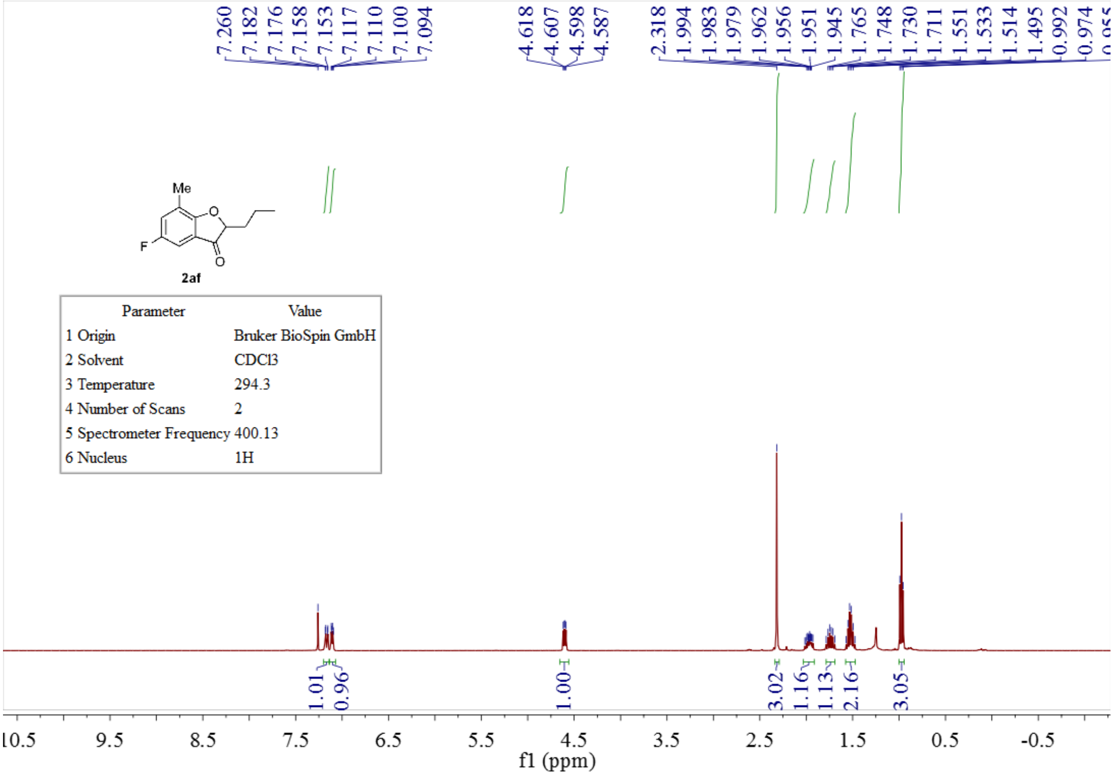

Supplementary Figure 155. **^{1}H NMR (400 MHz, CDCl$_3$) spectra for compound 2af**

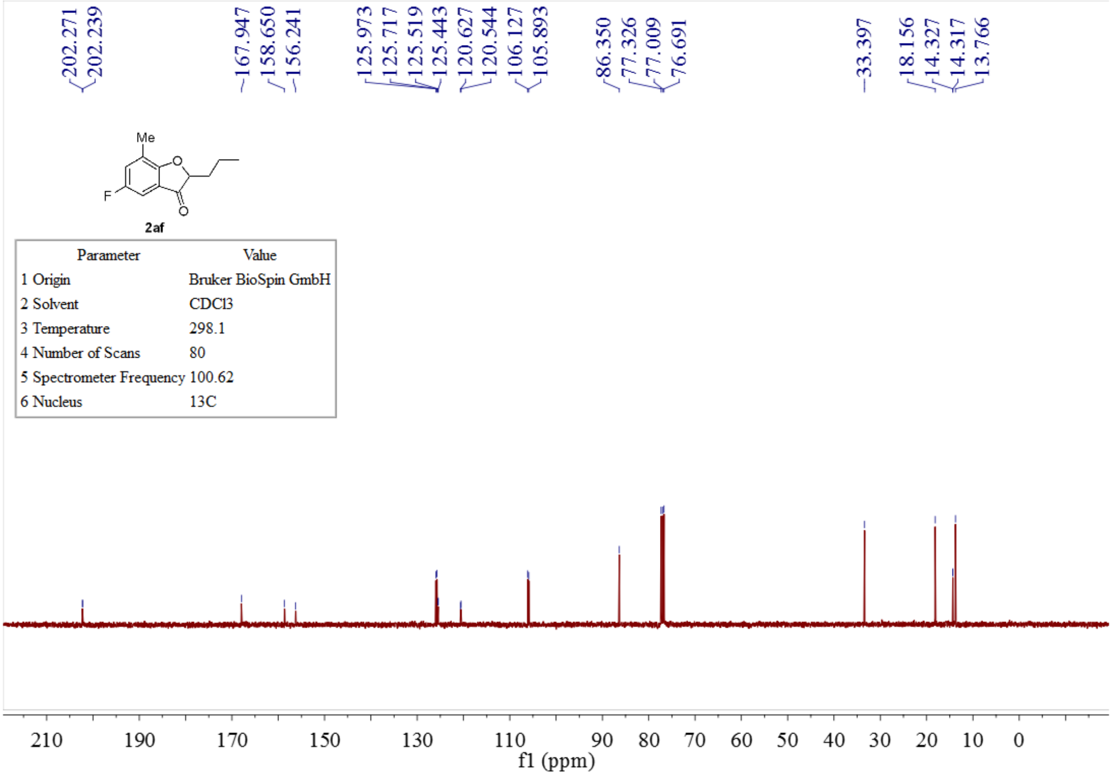

Supplementary Figure 156. ^{13}C **NMR (100 MHz,** $CDCl_3$**) spectra for compound 2af**

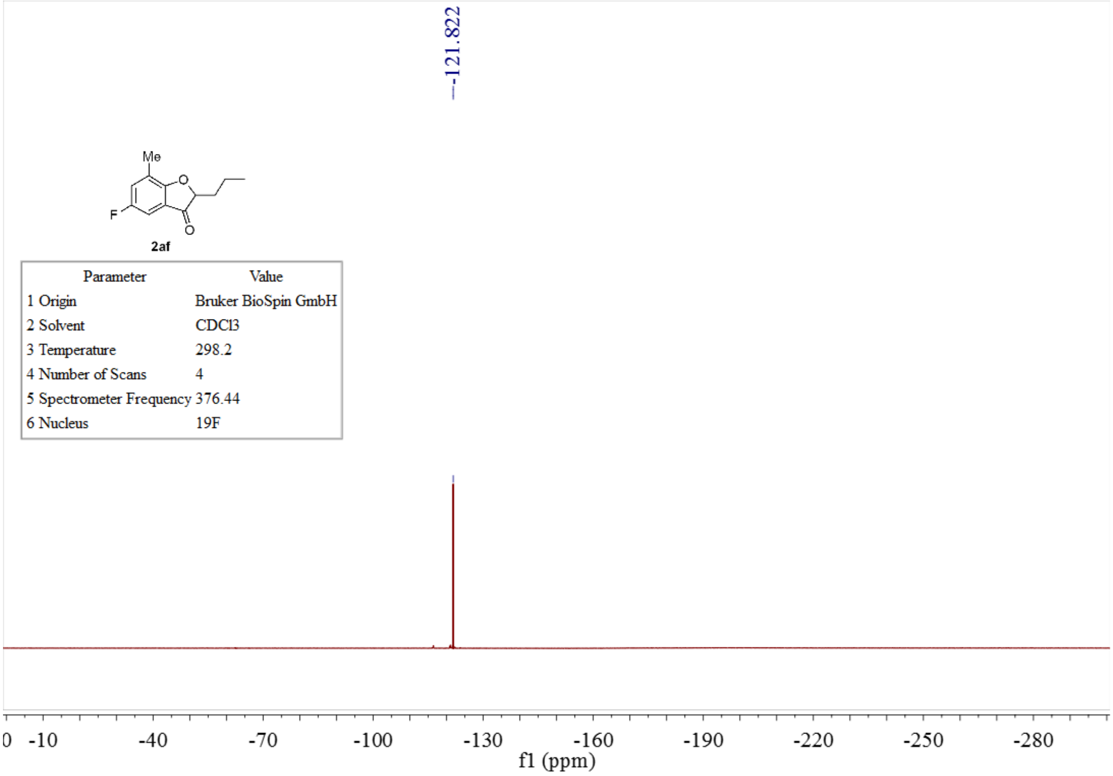

Supplementary Figure 157. ^{19}F **NMR (376 MHz,** $CDCl_3$**) spectra for compound 2af**

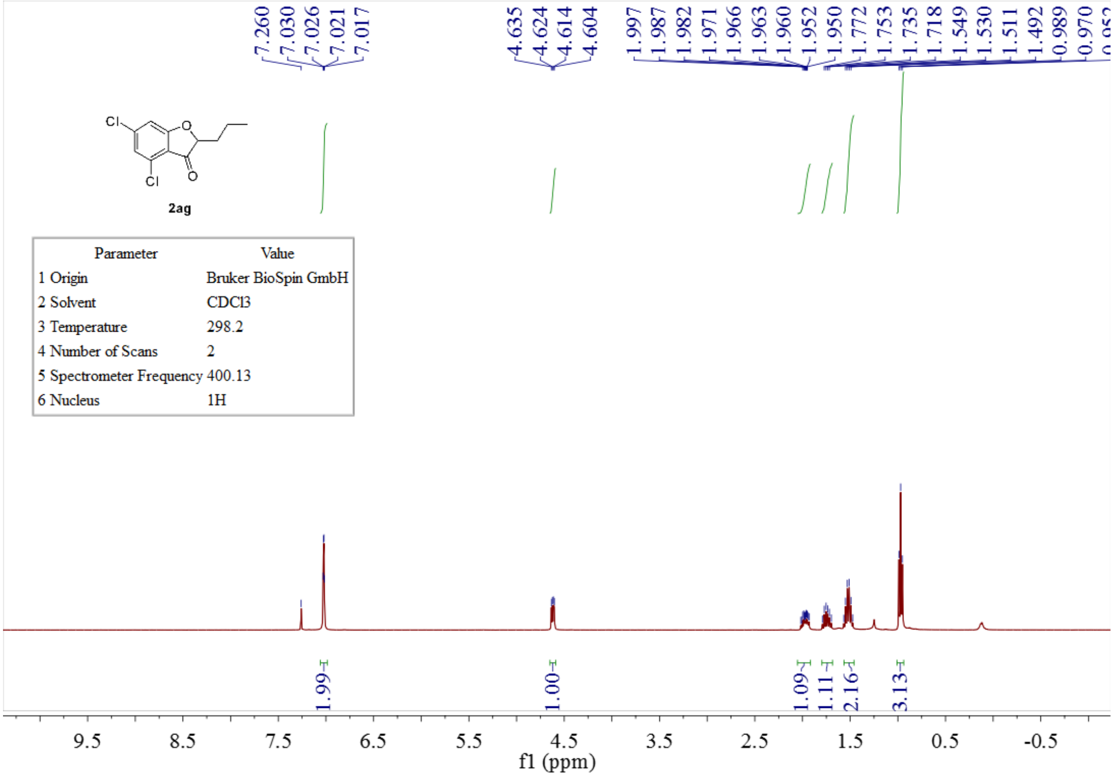

Supplementary Figure 158. ^{1}H NMR (400 MHz, $CDCl_3$) spectra for compound 2ag

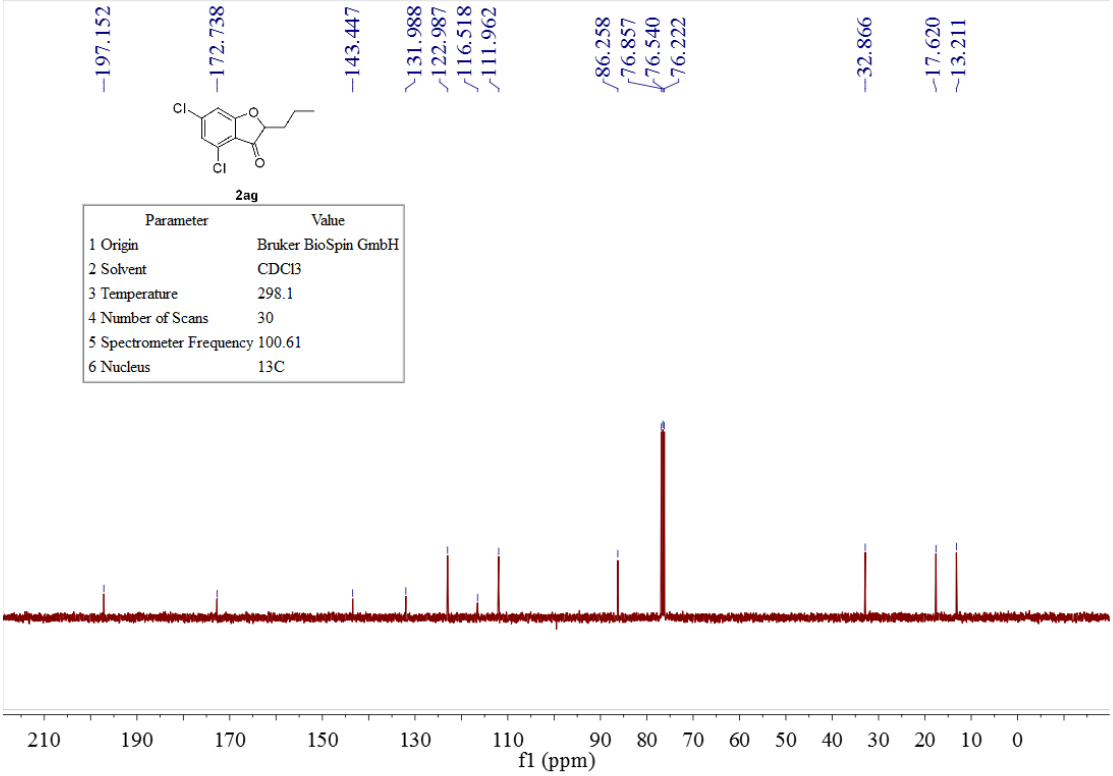

Supplementary Figure 159. ^{13}C NMR (100 MHz, $CDCl_3$) spectra for compound 2ag

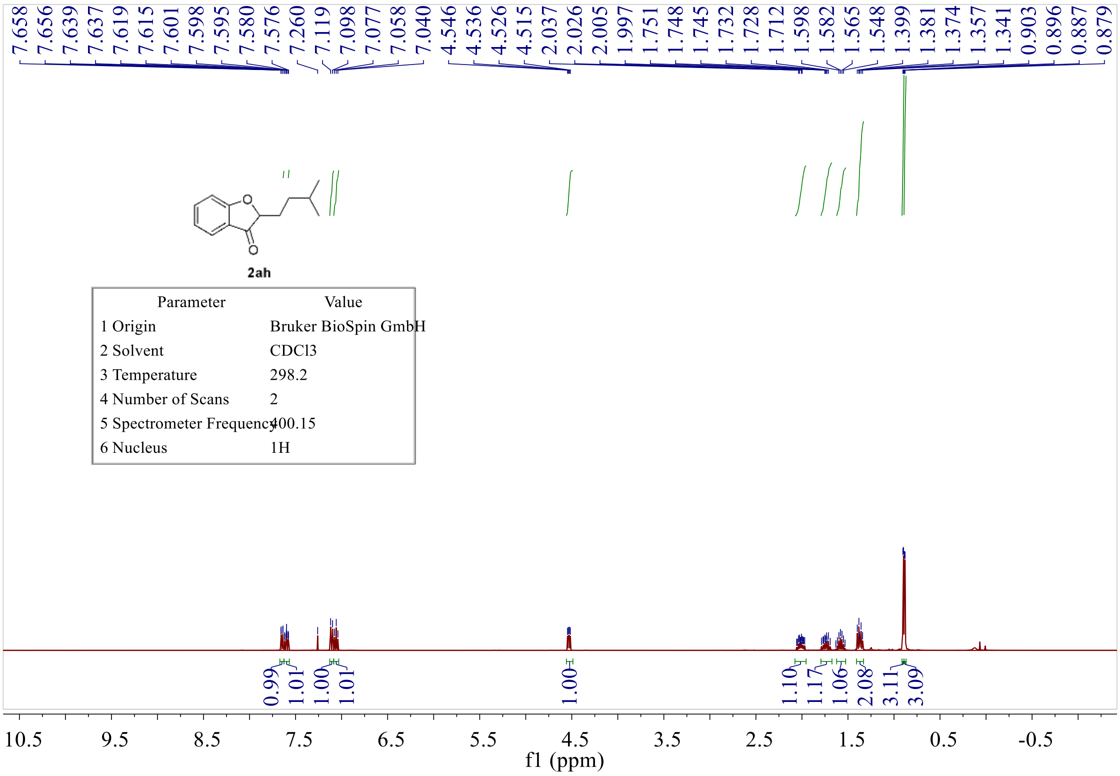

Supplementary Figure 160. ^{1}H **NMR (400 MHz,** $CDCl_3$**) spectra for compound 2ah**

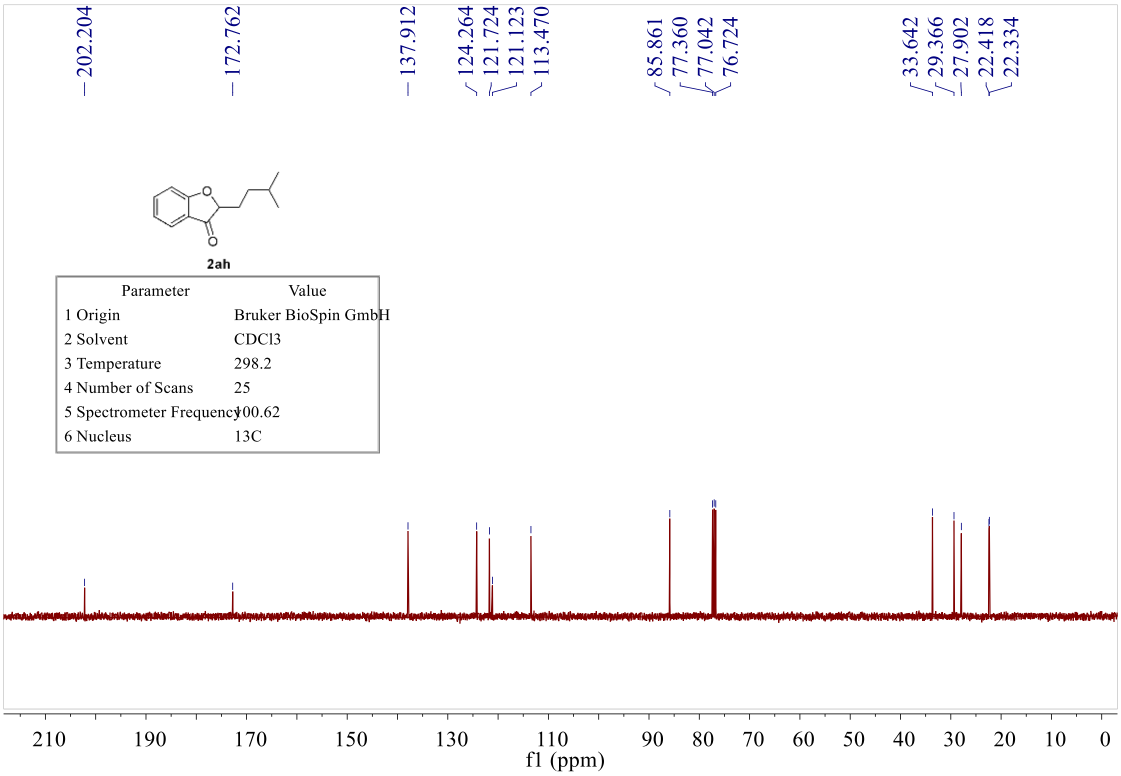

Supplementary Figure 161. ^{13}C **NMR (100 MHz,** $CDCl_3$**) spectra for compound 2ah**

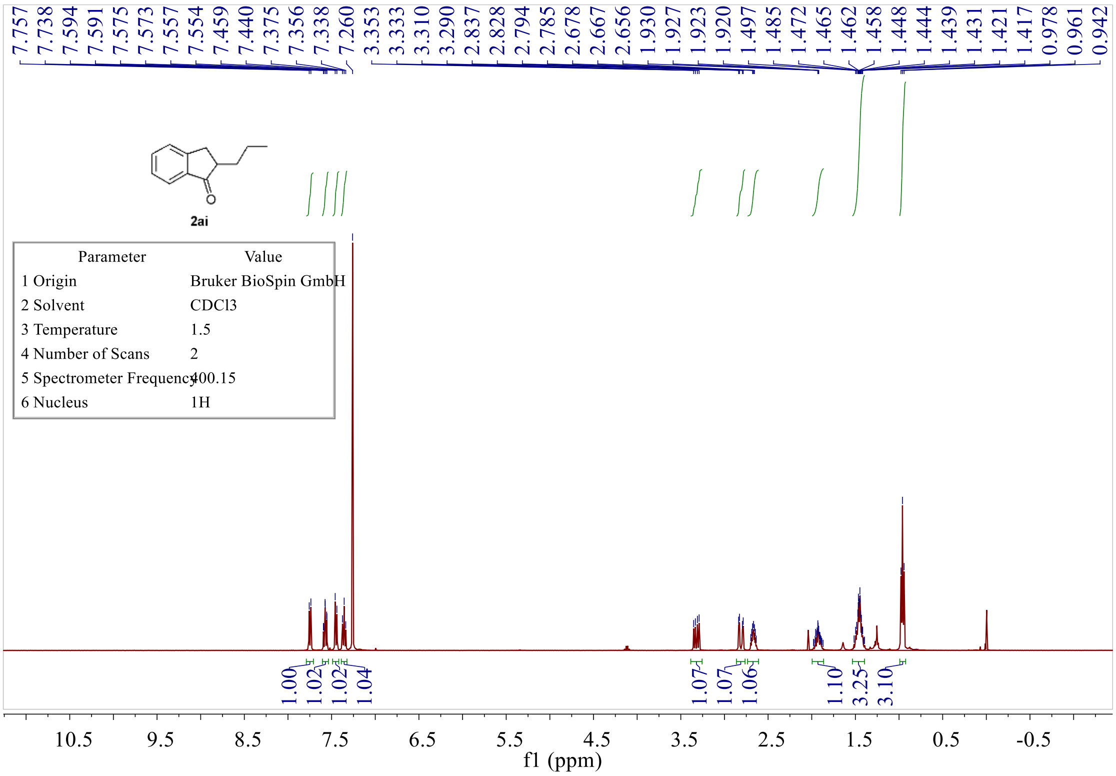

Supplementary Figure 162. ^{1}**H NMR (400 MHz, CDCl$_3$) spectra for compound 2ai**

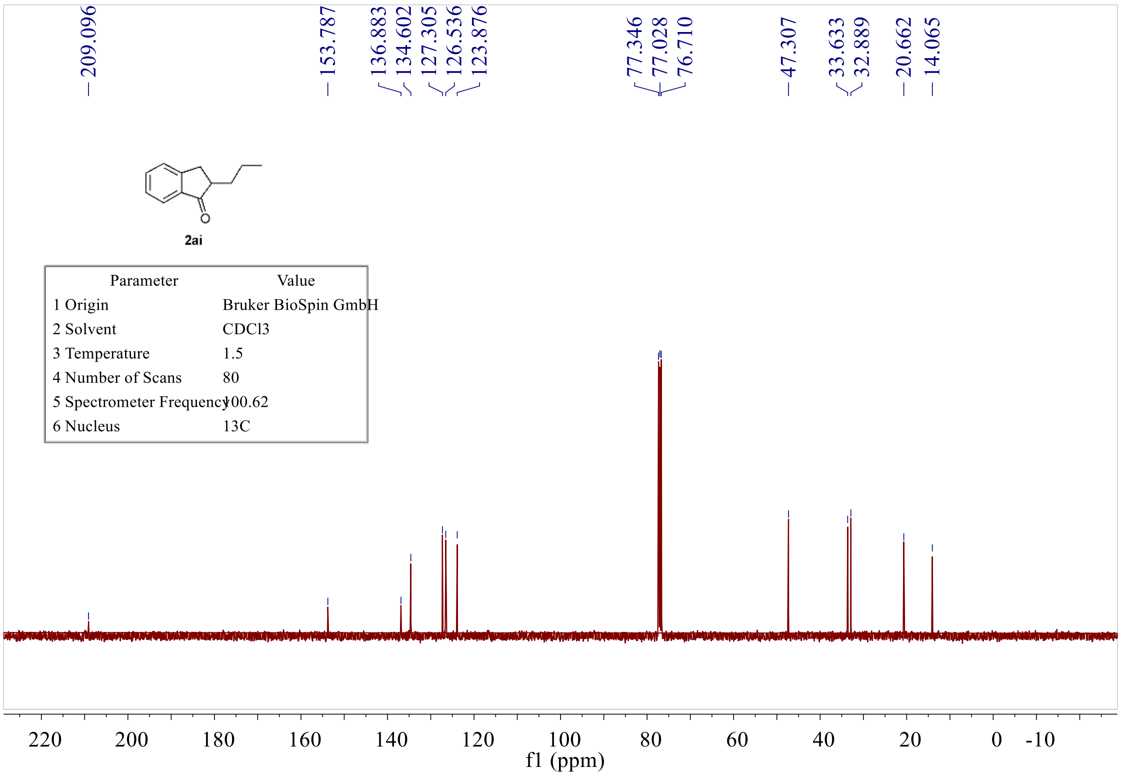

Supplementary Figure 163. ^{13}**C NMR (100 MHz, CDCl$_3$) spectra for compound 2ai**

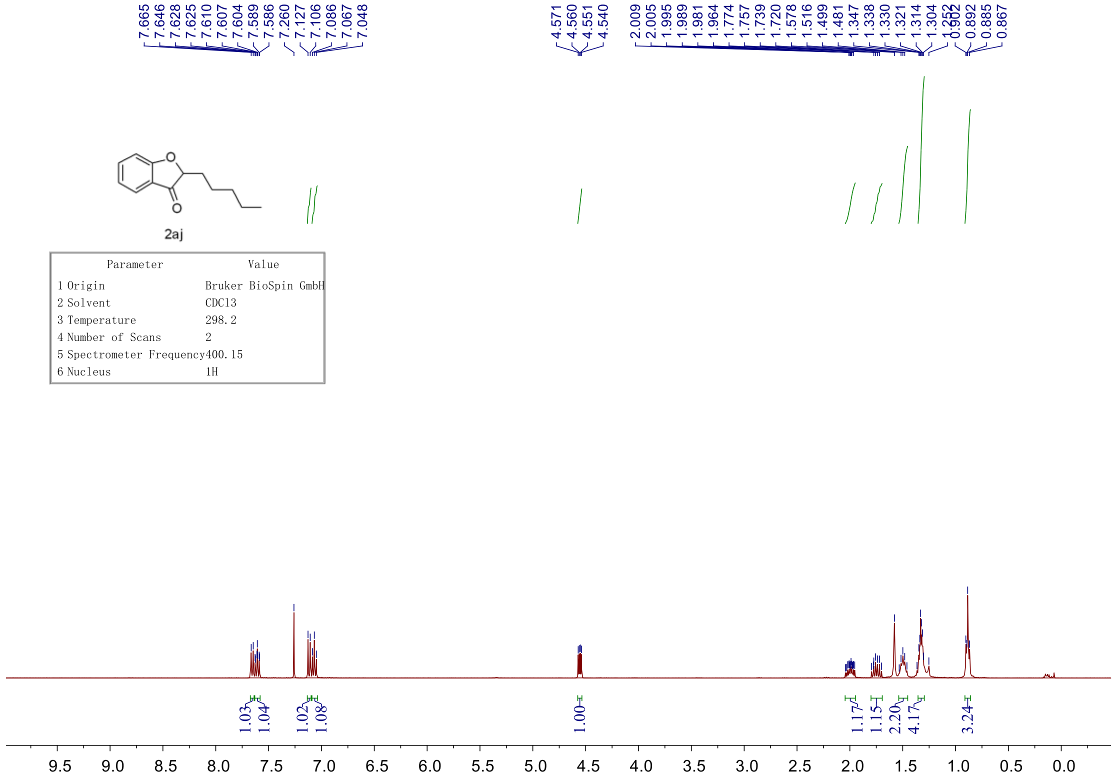

Supplementary Figure 164. ^{1}**H NMR (400 MHz, CDCl$_3$) spectra for compound 2aj**

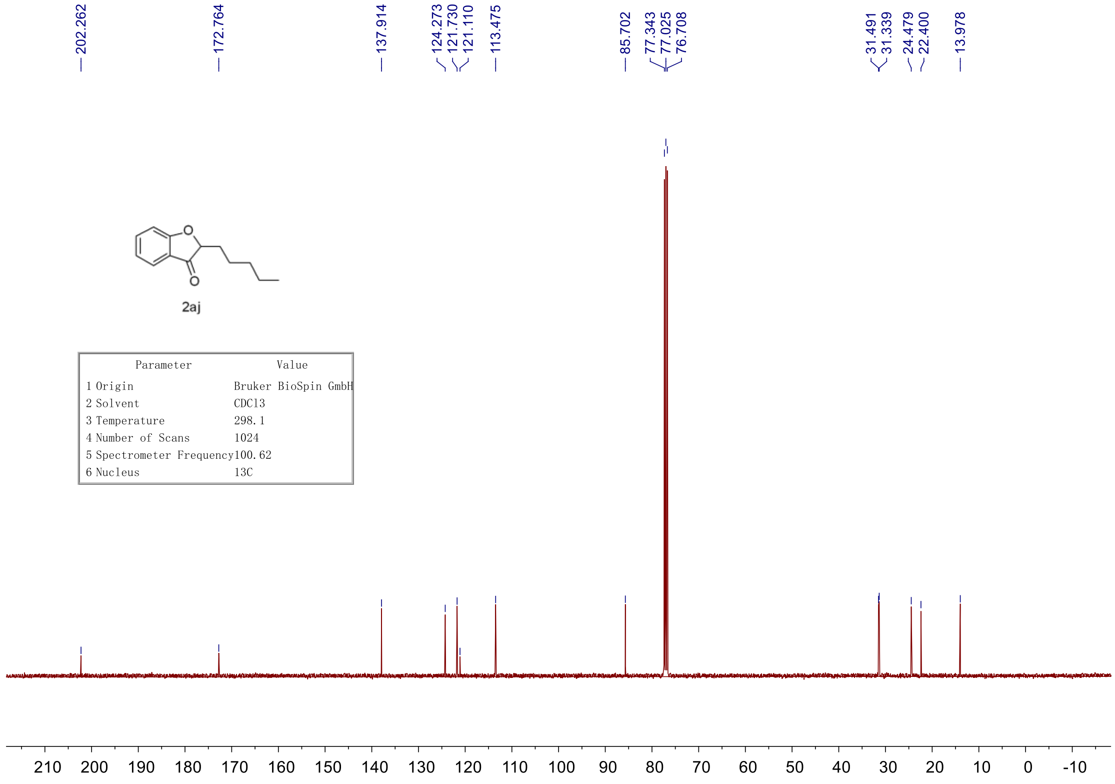

Supplementary Figure 165. ^{13}**C NMR (100 MHz, CDCl$_3$) spectra for compound 2aj**

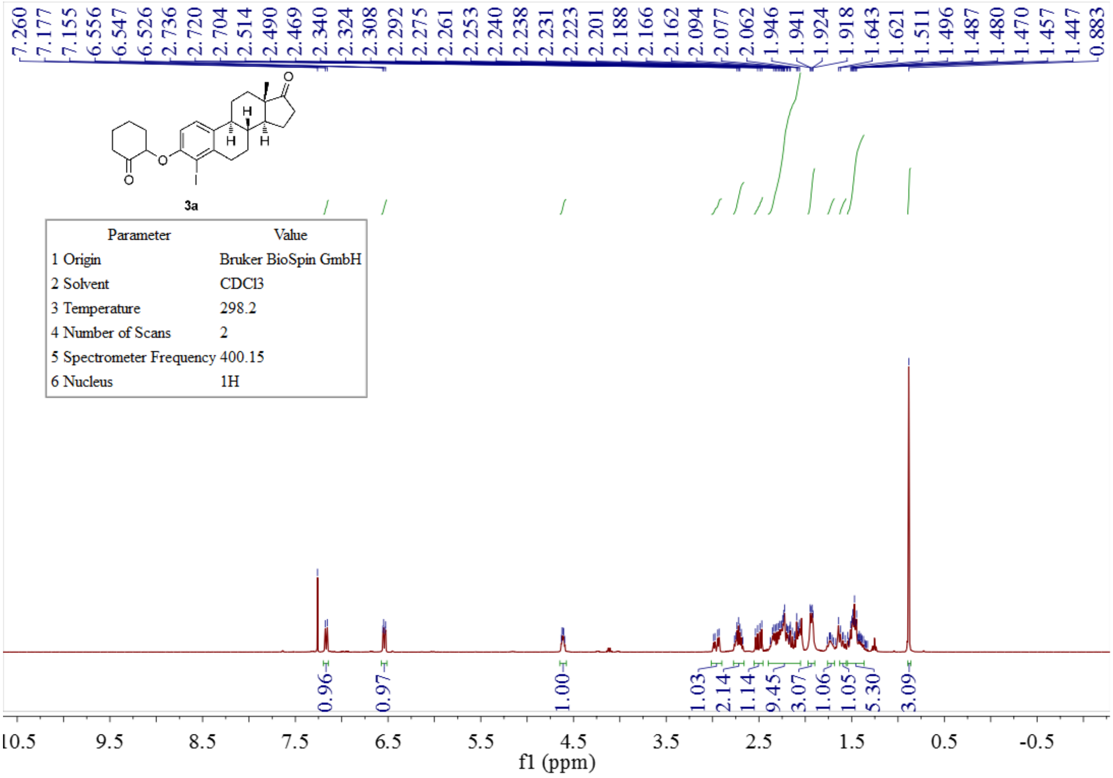

Supplementary Figure 166. ^{1}H NMR (400 MHz, CDCl$_3$) spectra for compound 3a

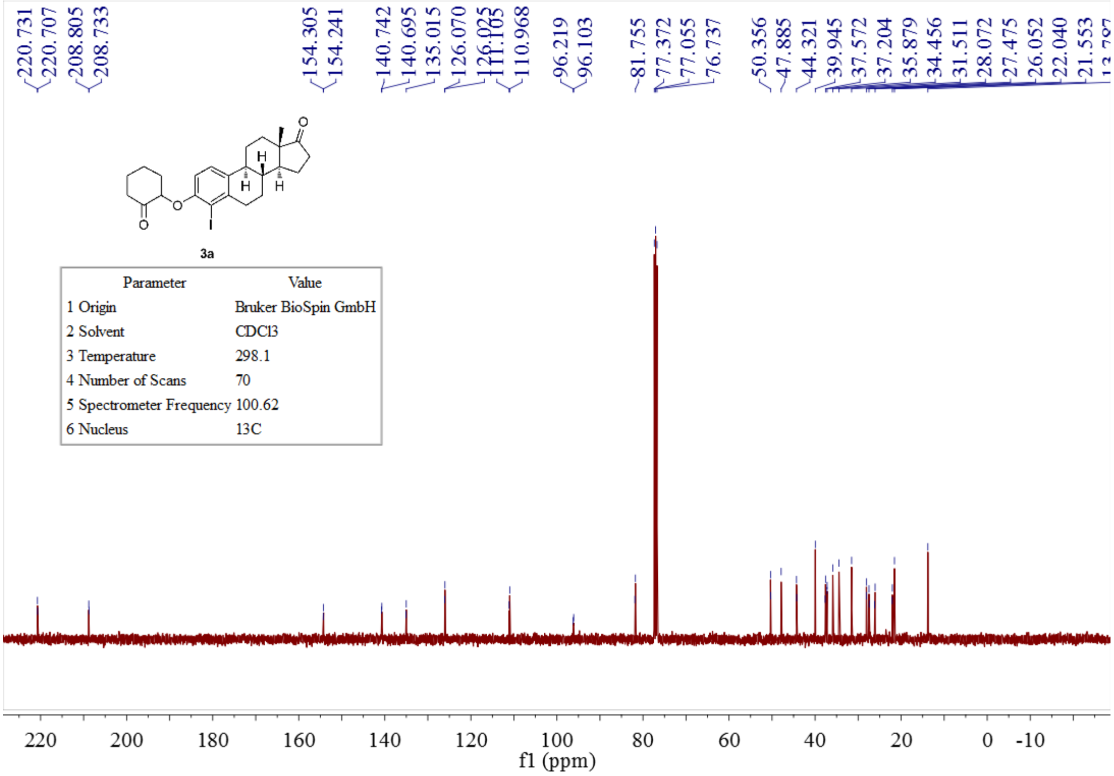

Supplementary Figure 167. ^{13}C NMR (100 MHz, CDCl$_3$) spectra for compound 3a

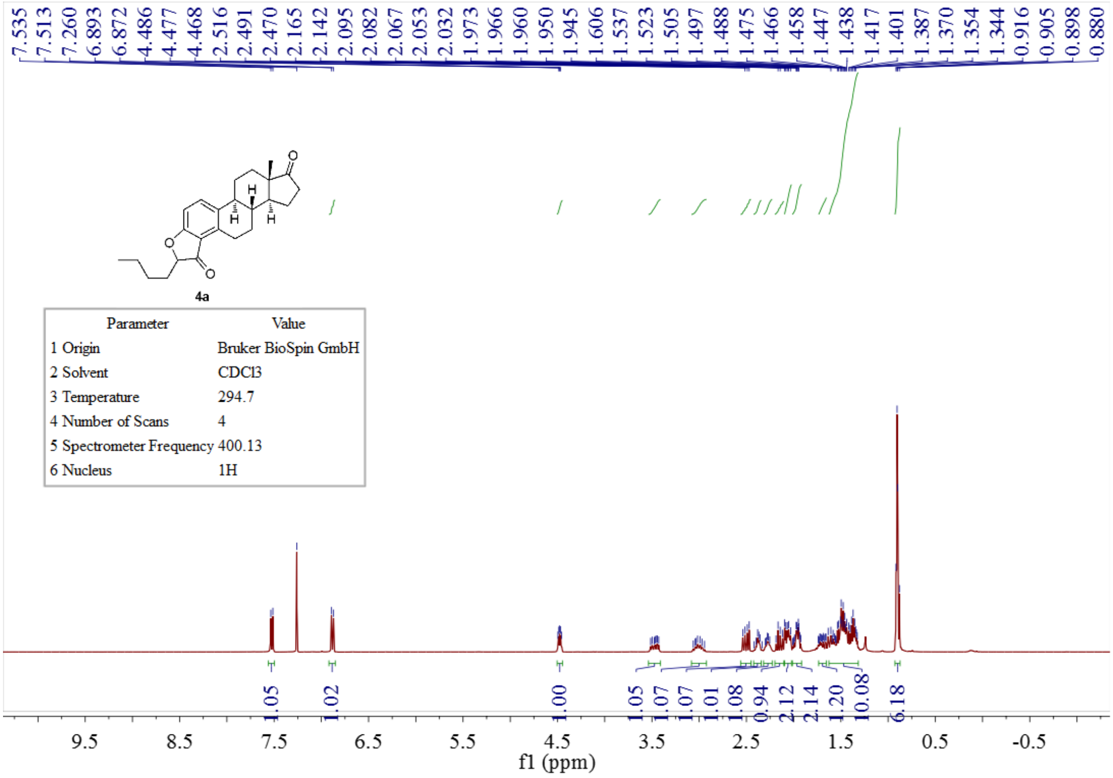

Supplementary Figure 168. ^{1}**H NMR (400 MHz, CDCl$_3$) spectra for compound 4a**

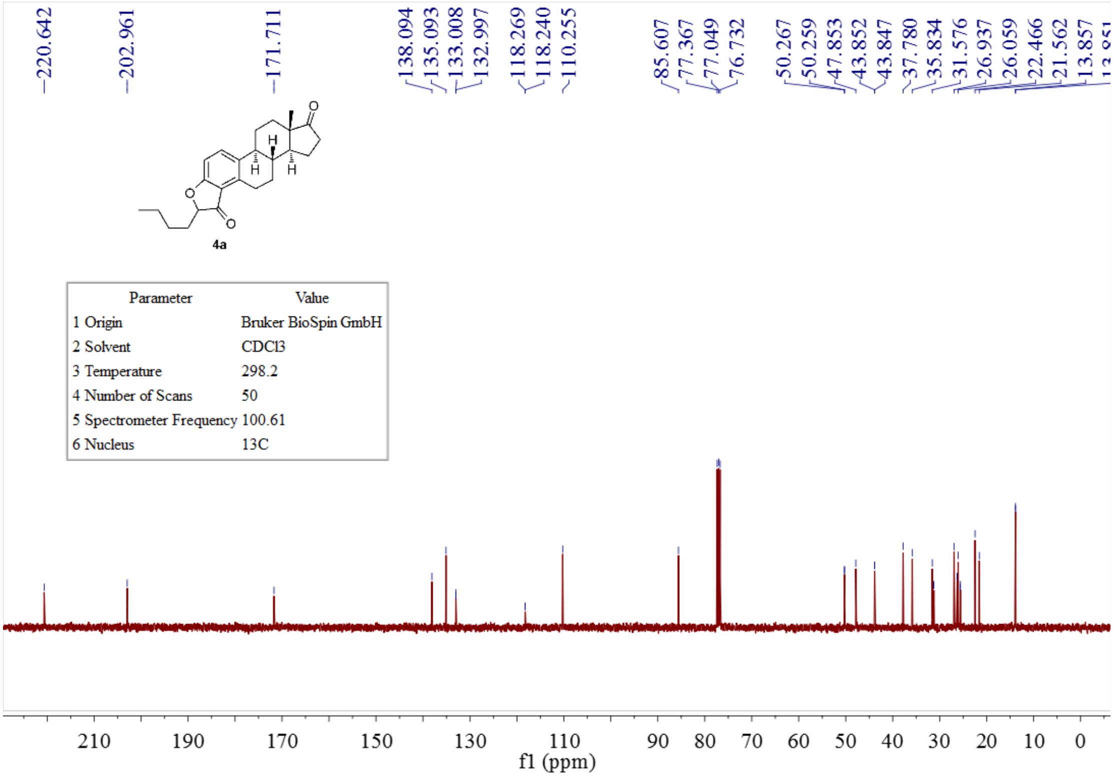

Supplementary Figure 169. ^{13}**C NMR (100 MHz, CDCl$_3$) spectra for compound 4a**

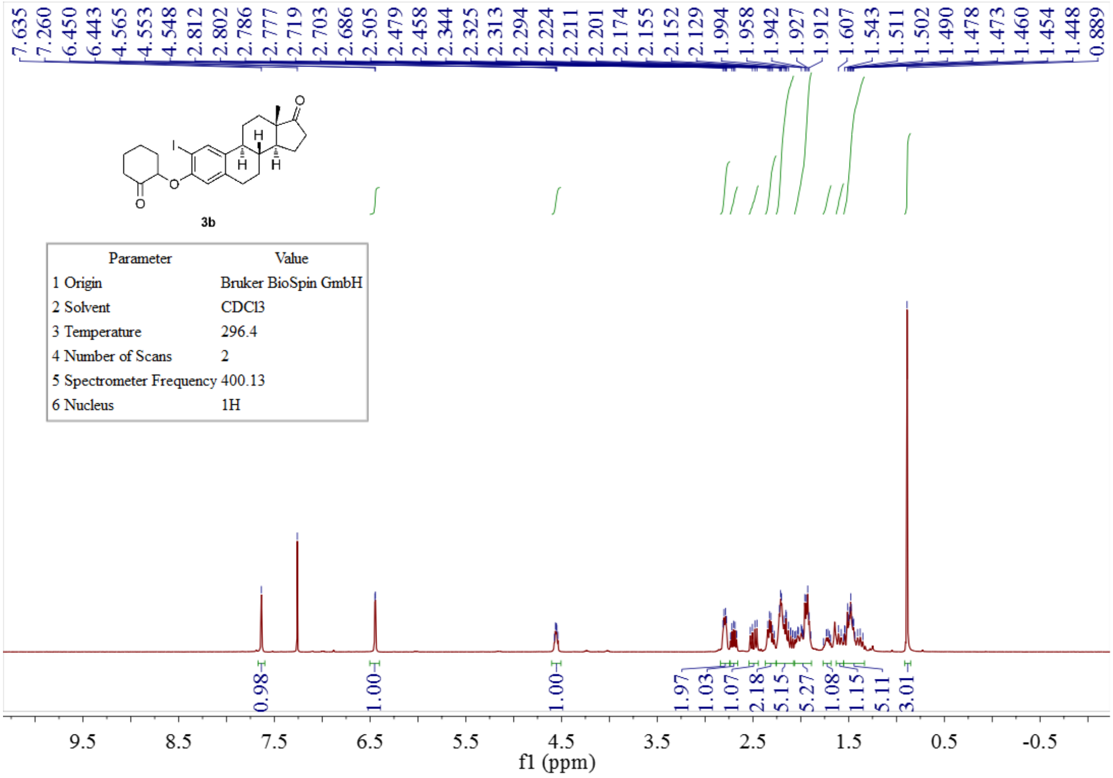

Parameter	Value
1 Origin	Bruker BioSpin GmbH
2 Solvent	CDCl3
3 Temperature	296.4
4 Number of Scans	2
5 Spectrometer Frequency	400.13
6 Nucleus	1H

Supplementary Figure 170. ^{1}H NMR (400 MHz, $CDCl_3$) spectra for compound 3b

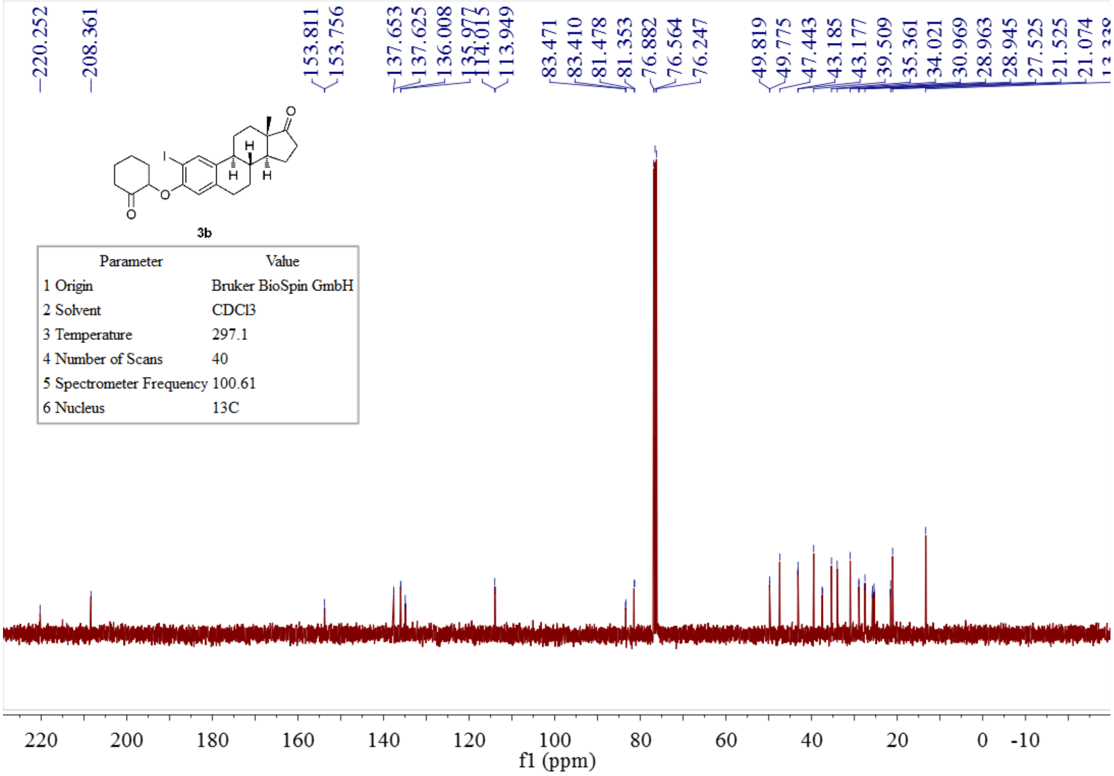

Parameter	Value
1 Origin	Bruker BioSpin GmbH
2 Solvent	CDCl3
3 Temperature	297.1
4 Number of Scans	40
5 Spectrometer Frequency	100.61
6 Nucleus	13C

Supplementary Figure 171. ^{13}C NMR (100 MHz, $CDCl_3$) spectra for compound 3b

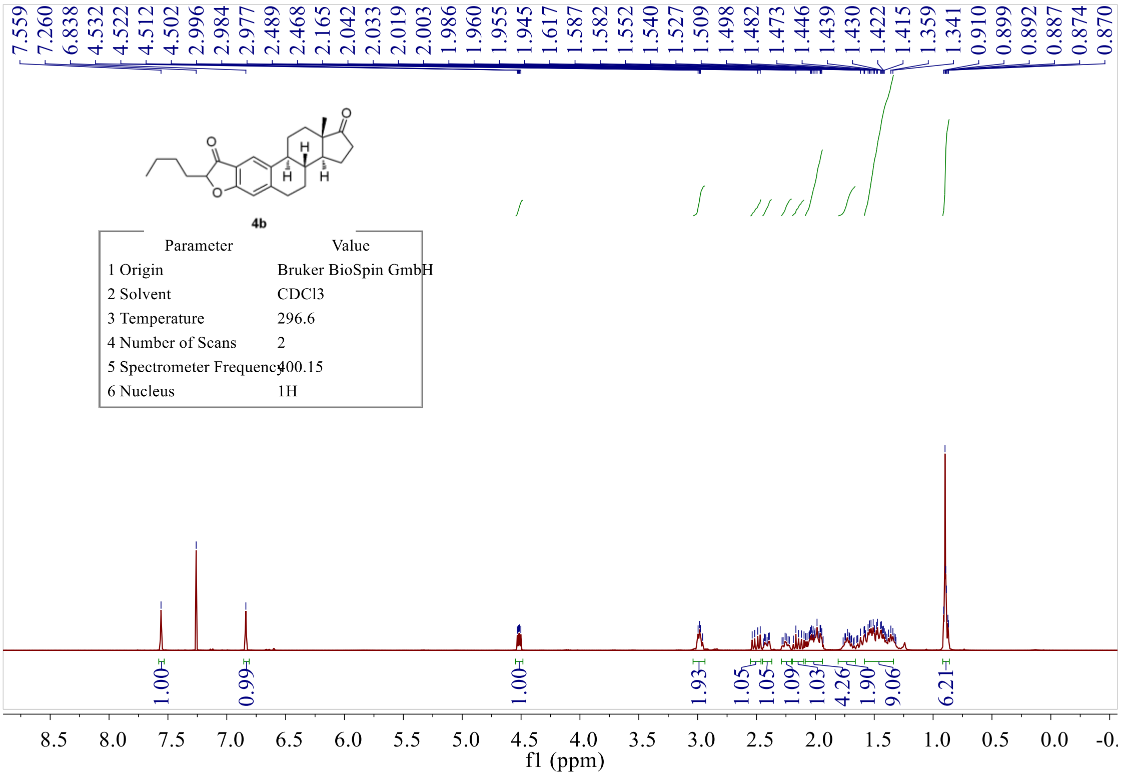

Supplementary Figure 172. ^{1}H NMR (400 MHz, $CDCl_3$) spectra for compound 4b

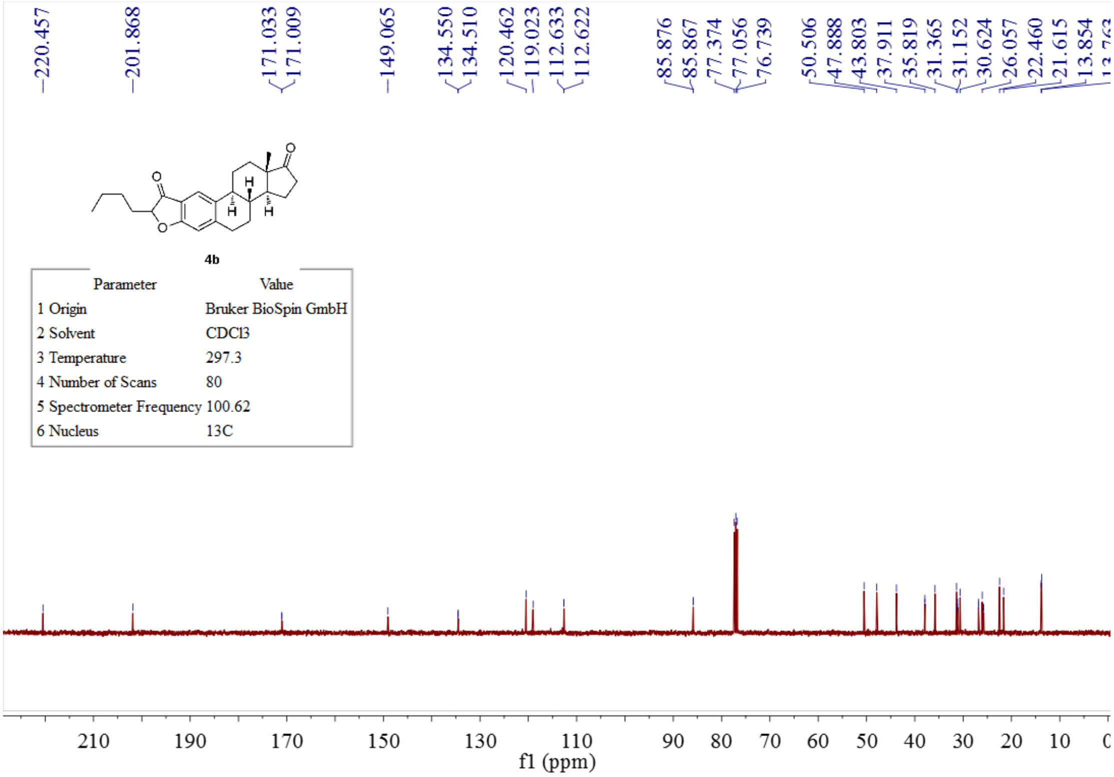

Supplementary Figure 173. ^{13}C NMR (100 MHz, $CDCl_3$) spectra for compound 4b

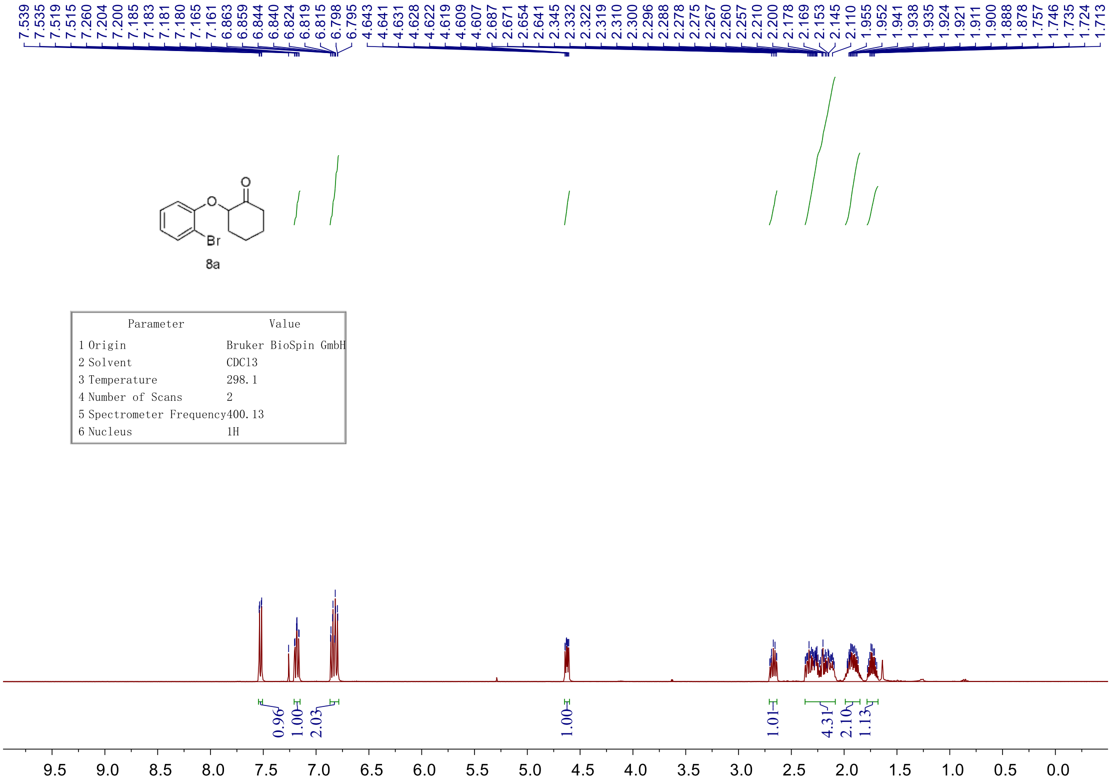

Supplementary Figure 174. ^{1}H NMR (400 MHz, $CDCl_3$) spectra for compound 8a

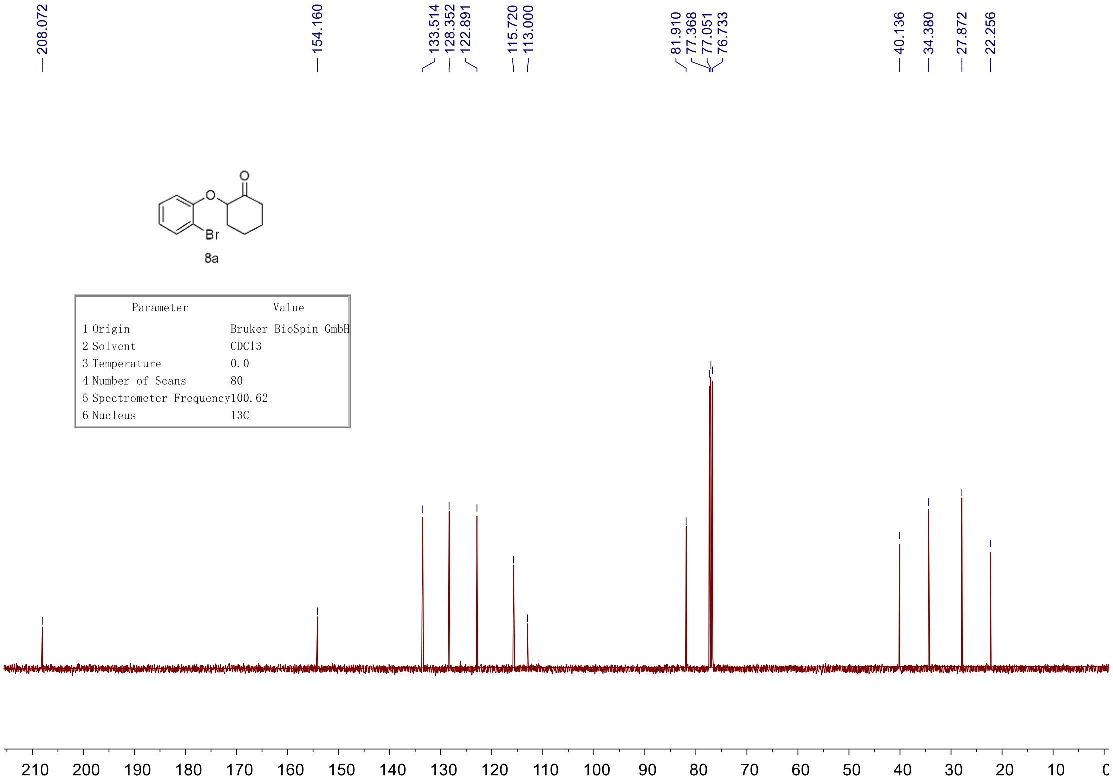

Supplementary Figure 175. ^{13}C NMR (100 MHz, $CDCl_3$) spectra for compound 8a

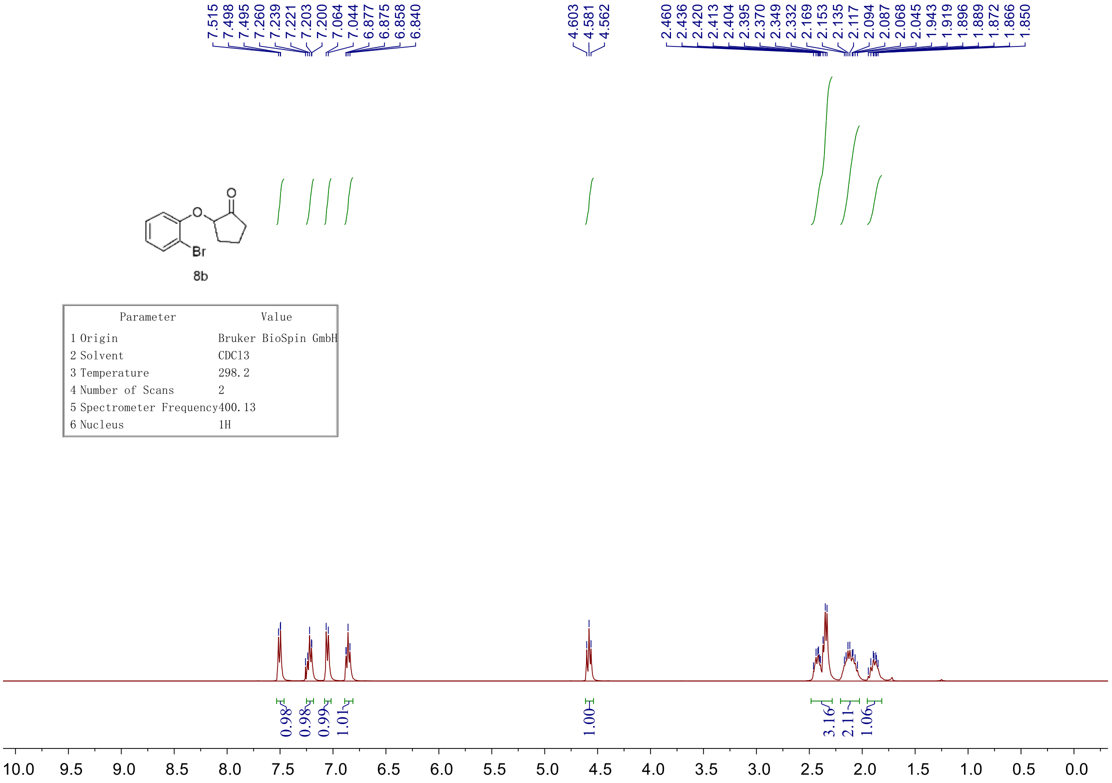

Supplementary Figure 176. ^{1}H NMR (400 MHz, $CDCl_3$) spectra for compound 8b

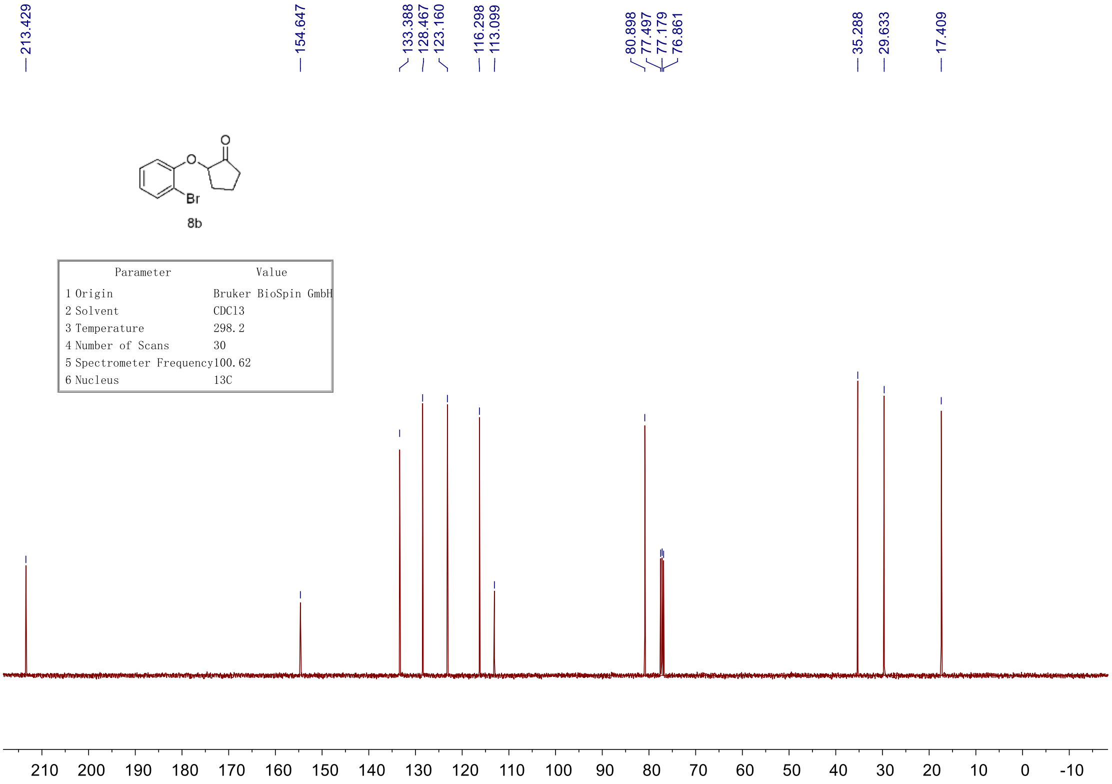

Supplementary Figure 177. ^{13}C NMR (100 MHz, $CDCl_3$) spectra for compound 8b

DFT Calculations

All theoretical calculations were performed with Gaussian 09[6]. Geometry optimizations and frequency computations were calculated by M06-2X[7,8] functional together with the 6-31G(d) basis set. Single-point energies were calculated the SMD-M06-2X/6-311++G(2d,p) method. The solvent effect of $PhCF_3$ was estimated by $PhCH_3$ with the SMD model.[9] All the optimized geometries were verified as minima or transition state structures by frequency calculations. A factor of RT ln (24.46) was added to free energy for each species to account for the 1 atm to 1 M standard state change. Structures were generated using CYLview[10]. All energies reported in the text are in kcal/mol, and bond lengths are in angstroms (Å) unless specified otherwise.

Supplementary Table 1. The natural bond orbital (NBO) analysis of Int.1 at the UM06-2X/6-311++G(2d, p) level of theory. Interaction energies are in kcal/mol.

Interaction	Lone-pair electrons		Unpaired electron	In total
	α spin	β spin	α spin	
$nO_4 \longrightarrow \sigma^*\ C_1{-}C_2$	**2.2**	**5.1**	**4.8**	**12.1**
$nO_4 \longrightarrow \sigma^*\ C_1{-}C_3$	**5.1**	**--** [a]	**--** [a]	**5.1**

[a] Below the threshold of 0.5 kcal/mol.

Supplementary Table 2. The natural bond orbital (NBO) analysis of Int.1 at different theoretical calculation levels of theory. Interaction energies are in kcal/mol.

Interaction	Lone-pair electrons		Unpaired electron	In total
	α spin	β spin	α spin	
$nO_4 \longrightarrow \sigma^*\ C_1{-}C_2$				
**UM06-2X/6-311++G(2d, p)**	**2.2**	**5.1**	**4.8**	**12.1**
**UPBE0-D3(BJ)/6-311++G(2d, p)**	**2.2**	**4.1**	**4.2**	**10.6**
**UB3LYP-D3(BJ)/6-311++G(2d, p)**	**2.3**	**4.0**	**3.6**	**9.9**
$nO_4 \longrightarrow \sigma^*\ C_1{-}C_3$				
**UM06-2X/6-311++G(2d, p)**	**5.1**	**--** [a]	**--** [a]	**5.1**
**UPBE0-D3(BJ)/6-311++G(2d, p)**	**4.5**			**4.5**
**UB3LYP-D3(BJ)/6-311++G(2d, p)**	**4.4**			**4.4**

[a] Below the threshold of 0.5 kcal/mol.

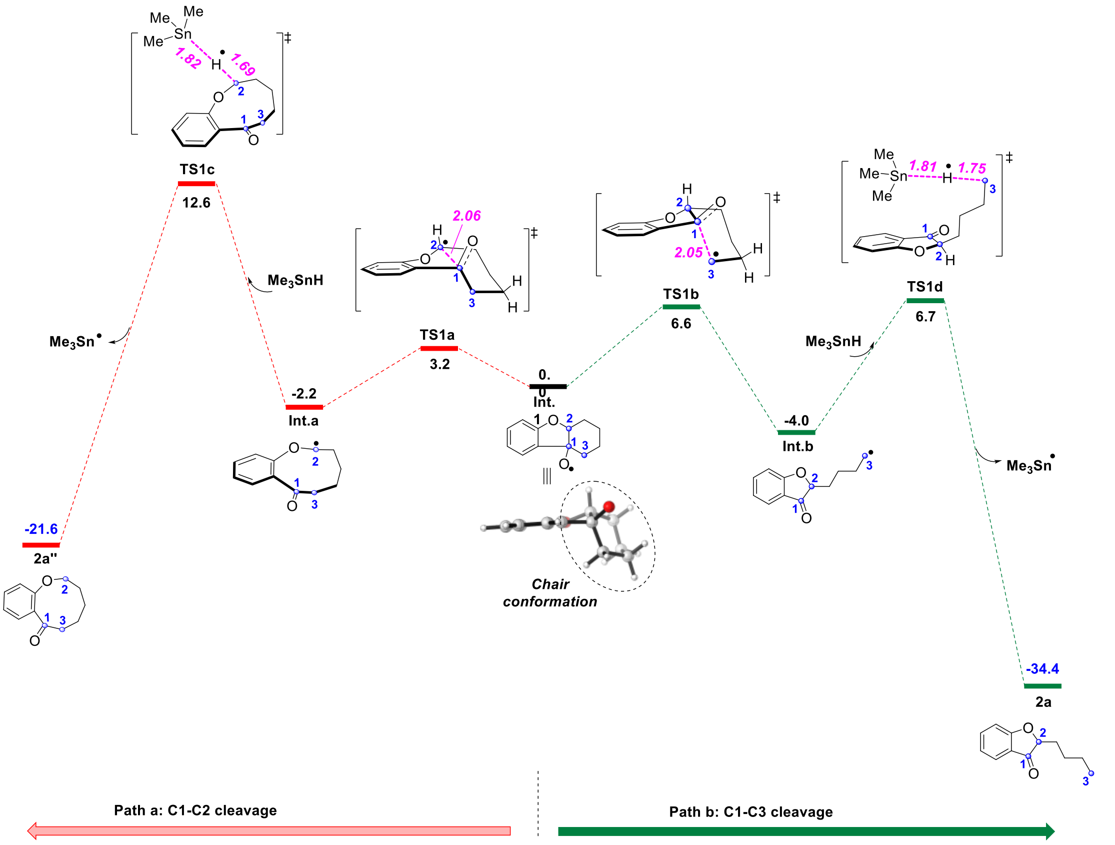

Supplementary Figure 178. Free energy diagram (kcal/mol) calculated at the (SMD)-M06-2X/6-311++G(2d,p)//M06-2X/6-31G(d) level of theory (pink: bond length in Å), wherein the trimethyltin hydride is used as a hydrogen atom donor. ΔG calculated at 298.15 K.

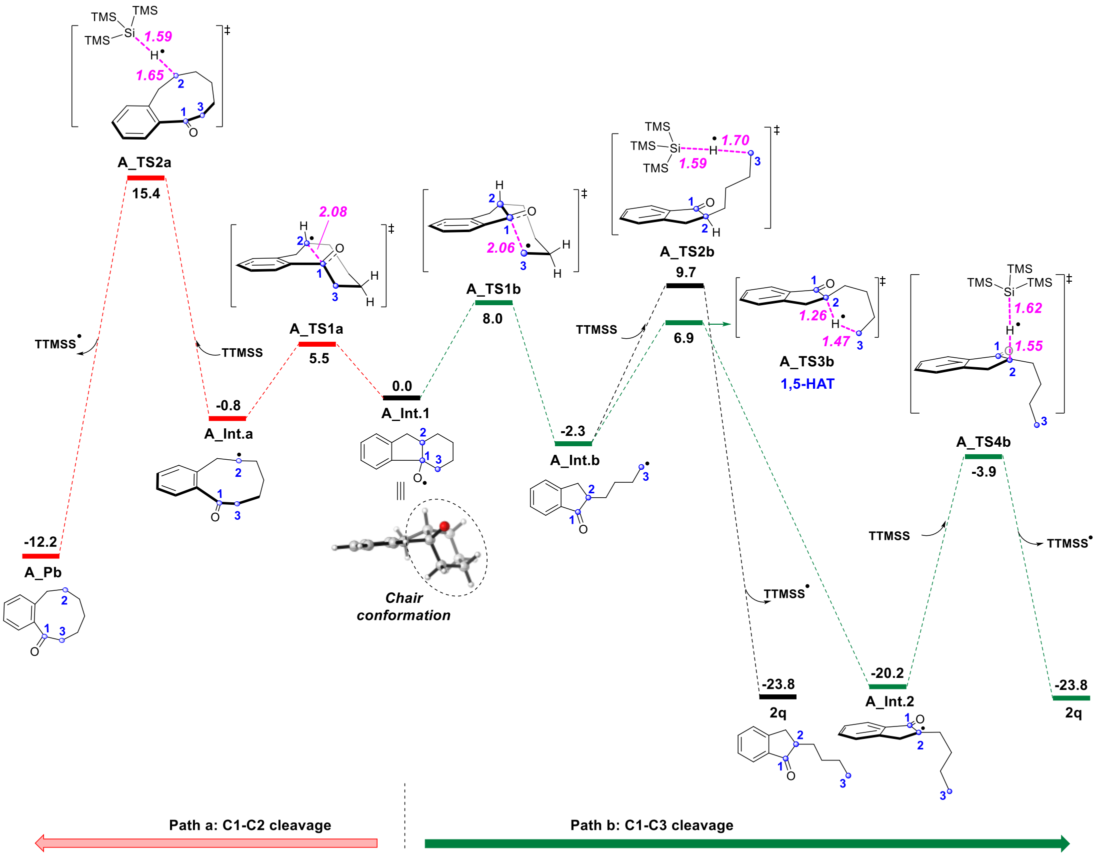

Supplementary Figure 179. Free energy diagram (kcal/mol) of carbon radical analogue (A_int.1) leading to indanone (2a) calculated at the (SMD)-M06-2X/6-311++G(2d,p)//M06-2X/6-31G(d) level of theory (pink: bond length in Å). ΔG calculated at 298.15 K.

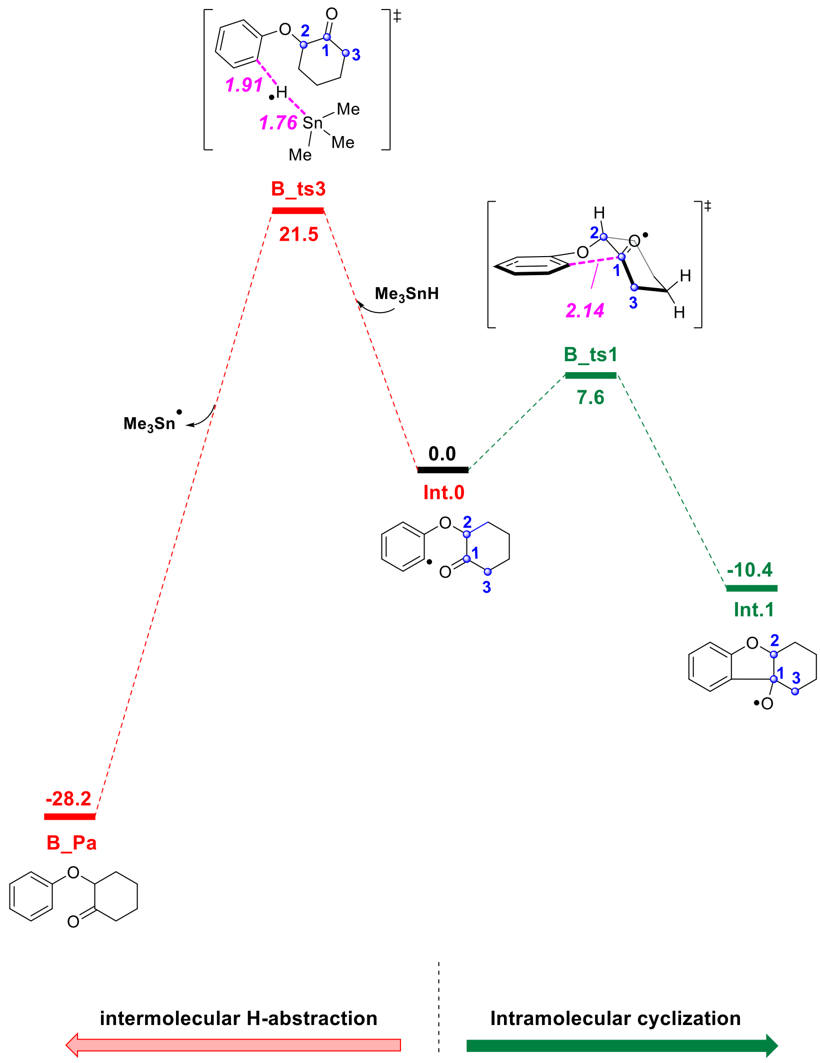

Supplementary Figure 180. Free energy diagram (kcal/mol) of intramolecular cyclization *vs* intermolecular H-abstraction calculated at the (SMD)-M06-2X/6-311++G(2d,p)//M06-2X/6-31G(d) level of theory (pink: bond length in Å). ΔG calculated at 298.15 K.

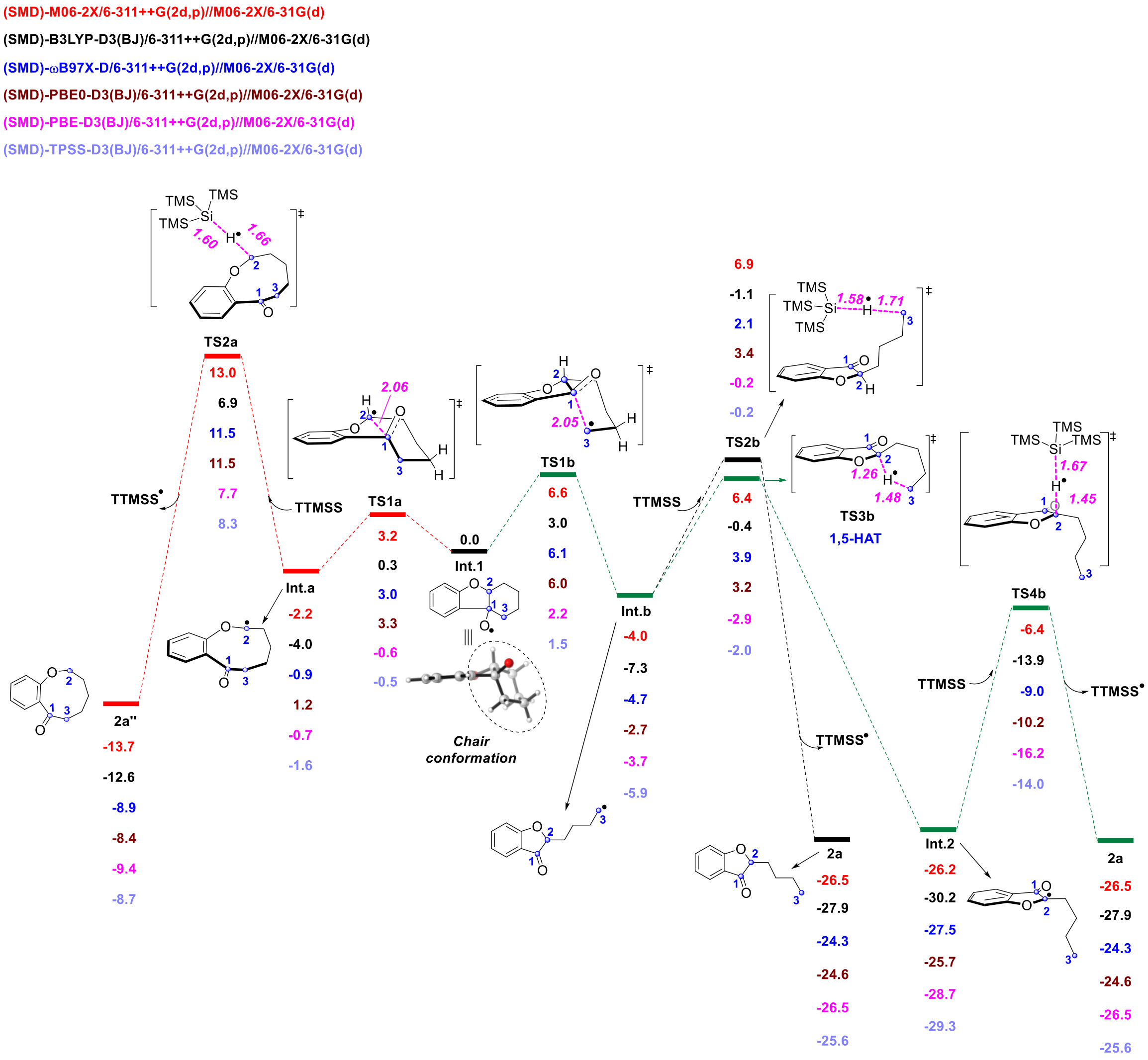

Supplementary Figure 181. Single-point calculations with different types of functionals including B3LYP-D3(BJ), ωB97X-D, PBE0-D3(BJ), PBE-D3(BJ), and TPSS-D3(BJ).

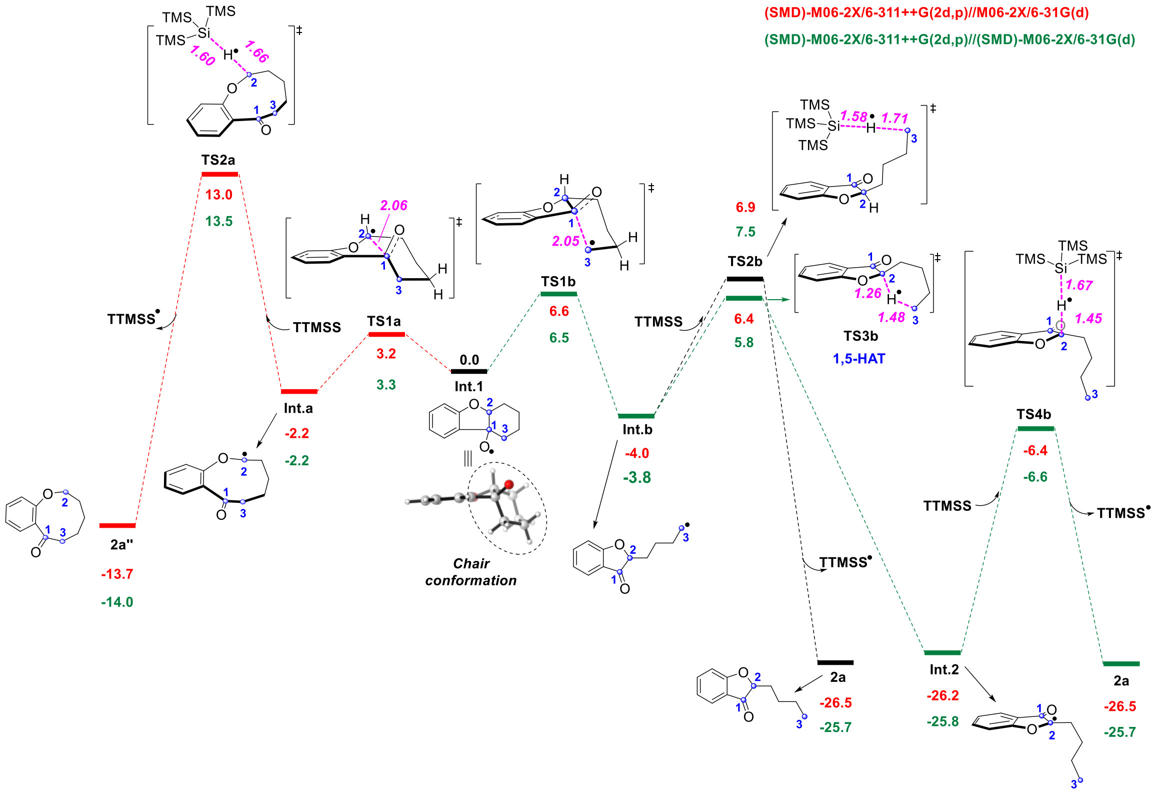

Supplementary Figure 182. Single-point calculations with solvent effects

Supplementary references:

1. Zhou, X., Fu, W., Jiang, H., Wang, C., Ju, C., Chu, W. & Sun, Z. Synthesis of 8-azaprotosappanin A derivatives via intramolecular palladium-catalyzed ortho C–H activation/C–C cyclization and their antibacterial activity. *Org. Biomol. Chem.*, **15**, 1956-1960 (2017).
2. Toda, Y., Komiyama, Y., Kikuchi, A. & Suga, H. Tetraarylphosphonium salt-catalyzed carbon dioxide fixation at atmospheric pressure for the synthesis of cyclic carbonates. *ACS Catal.*, **6**, 6906-6910 (2016).
3. Vyas, V. K. & Bhanage, B. M. Kinetic resolution driven diastereo- and enantioselective synthesis of cis-β-heteroaryl amino cycloalkanols by ruthenium-catalyzed asymmetric transfer hydrogenation. *Org. Lett.*, **18**, 6436-6439 (2016).
4. Berkowitz, W. F. & Wilson, P. J. Vinyl radical cyclizations: synthesis of substituted bicyclooctanols. *J. Org. Chem.*, **56**, 3097-3102 (1991).
5. Zheng, H., Zhu, Y. & Shi,Y. Palladium(0)-catalyzed Heck reaction/C-H activation/amination sequence with diaziridinone: A facile approach to indolines. *Angew. Chem. Int. Ed.*, **53**, 11280-11284 (2014).
6. Frisch, M. J., Trucks, G. W., Schlegel, H. B., Scuseria, G. E., Robb, M. A., Cheeseman, J. R., Scalmani, G., Barone, V., Mennucci, B., Petersson, G. A., Nakatsuji, H., Caricato, M., Li, X., Hratchian, H. P.; Izmaylov, A. F.; Bloino, J.; Zheng, G.; Sonnenberg, J. L.; Hada, M.; Ehara, M., Toyota, K., Fukuda, R., Hasegawa, J., Ishida, M., Nakajima, T., Honda, Y., Kitao, O., Nakai, H., Vreven, T., Montgomery, J. A., Jr., J. E. P., Ogliaro, F., Bearpark, M., Heyd, J. J., Brothers, E., Kudin, K. N., Staroverov, V. N., Keith, T., Kobayashi, R., Normand, J.,

Raghavachari, K., Rendell, A., Burant, J. C., Iyengar, S. S., Tomasi, J., Cossi, M., Rega, N., Millam, J. M., Klene, M., Knox, J. E., Cross, J. B., Bakken, V., Adamo, C., Jaramillo, J., Gomperts, R., Stratmann, R. E., Yazyev, O., Austin, A. J., Cammi, R., Pomelli, C., Ochterski, J. W., Martin, R. L., Morokuma, K., Zakrzewski, V. G., Voth, G. A., Salvador, P., Dannenberg, J. J., Dapprich, S., Daniels, A. D., Farkas, O., Foresman, J. B., Ortiz, J. V., Cioslowski, J. & Fox, D. J., Gaussian 09, Revision D.01, Gaussian, Inc., Wallingford CT, 2013.

7. Zhao, Y. & Truhlar, D. G. Density functionals with broad applicability in chemistry. *Acc. Chem. Res.* **41**, 157 (2008).
8. Zhao, Y. & Truhlar, D. G. Applications and validations of the Minnesota density functionals. *Chem. Phys. Lett.* 502, 1 (2011).
9. Marenich, A. V., Cramer, C. J. & Truhlar, D. G. Universal solvation model based on solute electron density and on a continuum model of the solvent defined by the bulk dielectric constant and atomic surface tensions. *J. Phys. Chem. B* **113**, 6378 (2009).
10. Legault, C. Y. CYLview, 1.0b; Université de Sherbrooke, Sherbrooke, Canada, **2009**; http://www.cylview.org.
