# Supplementary material for: Radical-mediated C-C cleavage of unstrained cycloketones and DFT study for unusual regioselectivity
Source: Nat Commun. 2020 Feb 3;11:672. doi: 10.1038/s41467-020-14435-5 (PMC6997357; doi:10.1038/s41467-020-14435-5)
Supplement: Supplementary file 3 — Description of Additional Supplementary Files [file 41467_2020_14435_MOESM3_ESM.pdf]

Description of Additional Supplementary files

File name: Supplementary Data 1

Description: Calculated Cartesian coordinates and energies by M06-2X/ 6-31G(d)
