# Supplementary material for: Radical-mediated C-C cleavage of unstrained cycloketones and DFT study for unusual regioselectivity
Source: Nat Commun. 2020 Feb 3;11:672. doi: 10.1038/s41467-020-14435-5 (PMC6997357; doi:10.1038/s41467-020-14435-5)
Supplement: Supplementary file 4 — Supplementary Data 1 [file 41467_2020_14435_MOESM4_ESM.pdf]

Calculated Cartesian coordinates and energies by M06-2X/ 6-31G(d)

Int.1

C	-3.42984	-0.5566	0.40526
C	-3.27967	0.81736	0.21716
C	-2.02633	1.35531	-0.08991
C	-0.95547	0.49019	-0.21752
C	-1.12076	-0.87998	-0.03418
C	-2.34978	-1.43478	0.28068
O	0.04548	-1.58434	-0.1909
H	-4.40985	-0.95825	0.64474
H	-4.14113	1.47067	0.30968
H	-1.88968	2.42345	-0.23515
H	-2.46	-2.50515	0.41528
C	0.52629	0.71619	-0.4373
C	0.95274	-0.71191	-0.88873
H	0.72224	-0.78735	-1.9615
O	0.90996	1.69205	-1.3051
C	1.19096	1.1167	0.92914
C	2.67718	0.78552	0.98901
C	2.88828	-0.71055	0.7553
C	2.39571	-1.09915	-0.63677
H	0.98719	2.17882	1.09716
H	3.22301	1.36299	0.2331
H	3.06797	1.08164	1.96856
H	3.94604	-0.97271	0.85839
H	2.33677	-1.28136	1.51311
H	3.00114	-0.58196	-1.39183
H	2.50233	-2.17408	-0.81654
H	0.65453	0.55178	1.70005

Zero-point correction= 0.227811 (Hartree/Particle)
Thermal correction to Energy= 0.238565
Thermal correction to Enthalpy= 0.239510
Thermal correction to Gibbs Free Energy= 0.190999
Sum of electronic and zero-point Energies= -614.998101
Sum of electronic and thermal Energies= -614.987346
Sum of electronic and thermal Enthalpies= -614.986402
Sum of electronic and thermal Free Energies= -615.034913
SCF Done: E(UM062X) = -615.425699262

TS1a

C	3.43784	-0.65166	-0.33211
C	3.3505	0.72247	-0.11386
C	2.11278	1.32309	0.12334
C	0.96858	0.53756	0.13768
C	1.0865	-0.83855	-0.0553
C	2.29906	-1.45861	-0.29759
O	-0.10363	-1.53627	0.03724
H	4.40585	-1.10835	-0.51303
H	4.25076	1.32794	-0.12886
H	2.02967	2.39264	0.29231
H	2.349	-2.53348	-0.43282
C	-0.45762	1.04134	0.27493
C	-0.93509	-0.86354	0.90218
H	-0.51275	-0.74235	1.90095
O	-0.80035	1.80794	1.19122
C	-1.23645	1.04122	-1.05336
C	-2.72755	0.76861	-0.87347
C	-2.99392	-0.70566	-0.57001
C	-2.39532	-1.13321	0.77497
H	-1.07321	2.03732	-1.48374
H	-3.10239	1.39953	-0.05786
H	-3.26548	1.05691	-1.78249
H	-4.07095	-0.90136	-0.54794
H	-2.57162	-1.32597	-1.37047

H	-2.88516	-0.58386	1.58696
H	-2.57932	-2.20367	0.95016
H	-0.79837	0.31298	-1.74526

Zero-point correction= 0.226391 (Hartree/Particle)
Thermal correction to Energy= 0.237060
Thermal correction to Enthalpy= 0.238004
Thermal correction to Gibbs Free Energy= 0.189745
Sum of electronic and zero-point Energies= -614.989539
Sum of electronic and thermal Energies= -614.978870
Sum of electronic and thermal Enthalpies= -614.977926
Sum of electronic and thermal Free Energies= -615.026185
SCF Done: E(UM062X) = -615.419278315

Int.a

C	3.37602	-0.85972	-0.26396
C	3.41621	0.51201	-0.0222
C	2.22983	1.21988	0.14917
C	1.00051	0.56888	0.0657
C	0.98355	-0.8153	-0.13654
C	2.15822	-1.53429	-0.30752
O	-0.2195	-1.48843	-0.13728
H	4.29941	-1.41454	-0.3982
H	4.36925	1.02794	0.0317
H	2.23607	2.29005	0.33455
H	2.10205	-2.6078	-0.45276
C	-0.27445	1.36617	0.13414
C	-1.03405	-1.14662	0.91789
H	-0.54981	-1.12871	1.89097
O	-0.42597	2.23237	0.96905
C	-1.28803	1.17274	-0.99058
C	-2.72556	0.86251	-0.53257
C	-3.05997	-0.63328	-0.48545
C	-2.48573	-1.37692	0.73026
H	-1.2797	2.13506	-1.51745
H	-2.89343	1.33142	0.44534
H	-3.4178	1.34232	-1.23173
H	-4.14808	-0.7625	-0.47736
H	-2.6966	-1.10901	-1.4046
H	-2.98837	-1.03481	1.64228
H	-2.70999	-2.45175	0.63056
H	-0.93854	0.41447	-1.69741

Zero-point correction= 0.226087 (Hartree/Particle)
Thermal correction to Energy= 0.237864
Thermal correction to Enthalpy= 0.238808
Thermal correction to Gibbs Free Energy= 0.187935
Sum of electronic and zero-point Energies= -614.996878
Sum of electronic and thermal Energies= -614.985102
Sum of electronic and thermal Enthalpies= -614.984158
Sum of electronic and thermal Free Energies= -615.035030
SCF Done: E(UM062X) = -615.426107202

TS2a

C	2.43359	3.18181	0.91129
C	3.4901	3.22346	0.003
C	4.05022	2.03694	-0.46044
C	3.57136	0.80457	-0.01473
C	2.47498	0.78283	0.85537
C	1.91267	1.96037	1.33174
O	1.91252	-0.41534	1.22524
H	1.99312	4.10476	1.27664
H	3.87668	4.17724	-0.34035
H	4.87846	2.04261	-1.16309
H	1.06154	1.89525	2.00293
C	4.25381	-0.45691	-0.4653
C	1.46922	-1.20037	0.17129
H	1.84177	-0.89547	-0.80622
O	4.63884	-0.57321	-1.60965

C	4.558	-1.53348	0.56947
C	3.95488	-2.92576	0.27095
C	2.67978	-3.24844	1.06226
C	1.38036	-2.65298	0.49134
H	5.65129	-1.61138	0.54598
H	3.78436	-3.02028	-0.80928
H	4.70718	-3.67812	0.52648
H	2.55479	-4.33598	1.11734
H	2.80637	-2.89703	2.09319
H	1.10914	-3.16966	-0.43755
H	0.56138	-2.82878	1.20433
H	4.27412	-1.20129	1.57144
H	-0.10483	-0.68696	0.01427
Si	-1.58079	-0.09547	-0.12202
Si	-2.66845	-1.60369	-1.54018
Si	-1.35093	2.01459	-1.12053
Si	-2.57123	0.00661	1.99097
C	-1.9012	-1.51707	-3.26845
H	-2.36454	-2.25093	-3.93792
H	-2.02348	-0.52714	-3.7202
H	-0.82782	-1.73301	-3.22509
C	-4.52092	-1.22969	-1.64359
H	-5.02062	-1.91932	-2.33374
H	-4.99271	-1.34151	-0.66059
H	-4.70753	-0.20843	-1.99187
C	-2.45379	-3.37095	-0.89552
H	-2.94444	-4.08641	-1.56584
H	-1.39464	-3.64167	-0.83147
H	-2.88921	-3.49018	0.10172
C	0.2369	2.04719	-2.14713
H	1.11796	1.87431	-1.51764
H	0.21886	1.27039	-2.91979
H	0.36405	3.01542	-2.64486
C	-2.84107	2.36056	-2.23836
H	-2.74848	3.34235	-2.7171
H	-2.94008	1.60975	-3.02933
H	-3.76928	2.35616	-1.65569
C	-1.29585	3.40161	0.16808
H	-0.46941	3.26424	0.87088
H	-1.15944	4.37134	-0.32505
H	-2.22686	3.44729	0.74359
C	-4.17338	1.01445	1.92426
H	-4.6718	1.01909	2.90065
H	-3.98078	2.05485	1.64197
H	-4.8706	0.59412	1.19155
C	-1.37444	0.81468	3.21444
H	-1.77344	0.79303	4.23493
H	-0.4107	0.29321	3.21175
H	-1.19599	1.86285	2.94735
C	-2.99953	-1.73428	2.59621
H	-3.78673	-2.17678	1.97525
H	-2.12957	-2.39862	2.55892
H	-3.36376	-1.70903	3.62965

Zero-point correction= 0.573768 (Hartree/Particle)
Thermal correction to Energy= 0.612739
Thermal correction to Enthalpy= 0.613683
Thermal correction to Gibbs Free Energy= 0.500924
Sum of electronic and zero-point Energies= -2132.180365
Sum of electronic and thermal Energies= -2132.141394
Sum of electronic and thermal Enthalpies= -2132.140449
Sum of electronic and thermal Free Energies= -2132.253208
SCF Done: E(UM062X) = -2133.19761933

2a''

C	0	0.09067	2.89045	0.80038
C	0	1.06039	3.50981	0.01343
C	0	2.13204	2.76759	-0.46995

C	0	2.25748	1.41405	-0.15437
C	0	1.25468	0.79508	0.60271
C	0	0.17598	1.53009	1.08268
O	0	1.31771	-0.54899	0.86901
H	0	-0.74843	3.46506	1.18091
H	0	0.98022	4.5662	-0.22102
H	0	2.90144	3.22515	-1.08498
H	0	-0.58358	1.01776	1.66402
C	0	3.4699	0.66713	-0.62558
C	0	1.21676	-1.38515	-0.28687
H	0	1.75419	-0.92869	-1.12761
O	0	3.92611	0.86742	-1.73276
C	0	4.18119	-0.27195	0.33999
C	0	4.22099	-1.75744	-0.09424
C	0	3.21868	-2.66913	0.63224
C	0	1.8103	-2.74166	0.03354
H	0	5.20759	0.11109	0.36307
H	0	4.10161	-1.82153	-1.18389
H	0	5.2255	-2.13577	0.117
H	0	3.62051	-3.68834	0.65671
H	0	3.14403	-2.34326	1.67576
H	0	1.82681	-3.31629	-0.90123
H	0	1.15216	-3.27761	0.7264
H	0	3.76729	-0.18003	1.34699
H	0	0.1614	-1.4662	-0.57953

Zero-point correction= 0.240846 (Hartree/Particle)
Thermal correction to Energy= 0.252420
Thermal correction to Enthalpy= 0.253364
Thermal correction to Gibbs Free Energy= 0.203531
Sum of electronic and zero-point Energies= -615.638859
Sum of electronic and thermal Energies= -615.627284
Sum of electronic and thermal Enthalpies= -615.626340
Sum of electronic and thermal Free Energies= -615.676174
SCF Done: E(RM062X) = -616.082749478

TTMSS

Si	0	0.00985	-0.00901	-0.85028
H	0	0.02158	-0.0304	-2.34823
Si	0	-2.1533	-0.48022	-0.0855
Si	0	0.6671	2.11072	-0.10134
Si	0	1.49788	-1.62905	-0.04335
C	0	-3.27079	1.03714	-0.25808
H	0	-4.28787	0.81124	0.08246
H	0	-2.89736	1.87718	0.33826
H	0	-3.33032	1.36756	-1.30054
C	0	-2.05814	-0.96349	1.7428
H	0	-3.06053	-1.13501	2.15162
H	0	-1.48201	-1.88595	1.87808
H	0	-1.58062	-0.17973	2.34136
C	0	-2.91202	-1.9128	-1.05863
H	0	-3.91756	-2.14289	-0.68839
H	0	-2.99181	-1.66656	-2.1221
H	0	-2.30427	-2.81912	-0.97019
C	0	-0.16986	3.4686	-1.11667
H	0	0.11274	3.39999	-2.1721
H	0	-1.26073	3.39333	-1.05707
H	0	0.12085	4.46041	-0.752
C	0	0.16035	2.29679	1.71333
H	0	0.49724	3.26003	2.11365
H	0	-0.92864	2.25103	1.82898
H	0	0.59598	1.50456	2.33229
C	0	2.54363	2.31239	-0.23118
H	0	2.88653	2.17534	-1.26215
H	0	2.85028	3.31236	0.0965
H	0	3.06626	1.58072	0.39528
C	0	1.86154	-1.26623	1.7787
H	0	2.50115	-2.04541	2.20911

H	0	2.3795	-0.30785	1.89729
H	0	0.93969	-1.22392	2.36909
C	0	3.12061	-1.59119	-1.01253
H	0	3.82524	-2.33312	-0.62
H	0	2.95085	-1.81296	-2.07093
H	0	3.5974	-0.6074	-0.94959
C	0	0.74145	-3.35841	-0.17785
H	0	-0.16833	-3.44207	0.42704
H	0	0.47769	-3.59697	-1.21359
H	0	1.4474	-4.11926	0.1745

Zero-point correction= 0.348720 (Hartree/Particle)
Thermal correction to Energy= 0.374599
Thermal correction to Enthalpy= 0.375544
Thermal correction to Gibbs Free Energy= 0.294496
Sum of electronic and zero-point Energies= -1517.185656
Sum of electronic and thermal Energies= -1517.159776
Sum of electronic and thermal Enthalpies= -1517.158832
Sum of electronic and thermal Free Energies= -1517.239879
SCF Done: E(RM062X) = -1517.78017125

TTMSS$^{\bullet}$

Si	0	-0.00862	-0.01015	-0.64945
Si	0	0.2342	2.23458	-0.0561
Si	0	1.83115	-1.32335	-0.05727
Si	0	-2.07421	-0.91663	-0.05162
C	0	2.04762	2.73945	-0.24032
H	0	2.18783	3.7947	0.02021
H	0	2.68988	2.14426	0.41849
H	0	2.39708	2.59584	-1.26811
C	0	-0.27574	2.46489	1.75381
H	0	-0.12827	3.50383	2.07185
H	0	-1.33177	2.21549	1.90584
H	0	0.31751	1.82097	2.41214
C	0	-0.83824	3.35214	-1.13984
H	0	-0.75446	4.39641	-0.81765
H	0	-0.53118	3.29519	-2.18886
H	0	-1.89355	3.06559	-1.0834
C	0	3.3369	-0.88556	-1.11331
H	0	3.13544	-1.05209	-2.17603
H	0	3.6171	0.16525	-0.98502
H	0	4.19797	-1.50154	-0.82974
C	0	2.26014	-1.02761	1.76454
H	0	3.10742	-1.65248	2.07084
H	0	2.5332	0.0182	1.9441
H	0	1.40992	-1.26684	2.41235
C	0	1.41744	-3.15432	-0.29012
H	0	1.14523	-3.37201	-1.32807
H	0	2.27848	-3.77986	-0.02813
H	0	0.57943	-3.45632	0.34798
C	0	-2.02068	-1.42281	1.77318
H	0	-2.99175	-1.80922	2.10426
H	0	-1.27289	-2.2062	1.93924
H	0	-1.75794	-0.57062	2.40922
C	0	-2.48708	-2.43931	-1.09244
H	0	-3.45124	-2.86247	-0.78775
H	0	-2.54774	-2.18744	-2.15576
H	0	-1.7255	-3.21713	-0.97477
C	0	-3.42667	0.38526	-0.28733
H	0	-3.24578	1.26162	0.34517
H	0	-3.47159	0.72495	-1.32728
H	0	-4.40872	-0.02211	-0.02121

Zero-point correction= 0.340470 (Hartree/Particle)
Thermal correction to Energy= 0.366265
Thermal correction to Enthalpy= 0.367209
Thermal correction to Gibbs Free Energy= 0.284237
Sum of electronic and zero-point Energies= -1516.561812
Sum of electronic and thermal Energies= -1516.536017

Sum of electronic and thermal Enthalpies= -1516.535073
Sum of electronic and thermal Free Energies= -1516.618045
SCF Done: E(UM062X) = -1517.14723265

TS1b

C	-3.362	-0.62123	0.47344
C	-3.28006	0.75519	0.24036
C	-2.07409	1.3307	-0.1573
C	-0.97709	0.50023	-0.32959
C	-1.07333	-0.87115	-0.09772
C	-2.25996	-1.46198	0.30933
O	0.10418	-1.53903	-0.28879
H	-4.30883	-1.05128	0.78625
H	-4.16192	1.37416	0.36951
H	-1.98097	2.39764	-0.33888
H	-2.3186	-2.53016	0.48631
C	0.44865	0.77182	-0.68921
C	0.98055	-0.65036	-1.00332
H	0.7791	-0.79212	-2.07703
O	0.93081	1.80669	-1.18288
C	1.10747	0.96257	1.24315
C	2.57829	0.70618	1.18025
C	2.87293	-0.72149	0.71891
C	2.44172	-0.93955	-0.72918
H	0.76893	1.98339	1.3977
H	3.04886	1.43422	0.50926
H	3.00301	0.87143	2.18253
H	3.94321	-0.93303	0.80894
H	2.34801	-1.43095	1.37063
H	3.01235	-0.27128	-1.38665
H	2.65822	-1.96628	-1.04285
H	0.51026	0.20729	1.75288

Zero-point correction= 0.225277 (Hartree/Particle)
Thermal correction to Energy= 0.236101
Thermal correction to Enthalpy= 0.237045
Thermal correction to Gibbs Free Energy= 0.188397
Sum of electronic and zero-point Energies= -614.984007
Sum of electronic and thermal Energies= -614.973183
Sum of electronic and thermal Enthalpies= -614.972239
Sum of electronic and thermal Free Energies= -615.020887
SCF Done: E(UM062X) = -615.412506242

Int.b

C	0	5.43093	2.34727	-1.17458
C	0	4.64382	2.84655	-0.12432
C	0	3.94516	1.97404	0.69809
C	0	4.05915	0.60711	0.45323
C	0	4.83848	0.12596	-0.59713
C	0	5.54413	0.98566	-1.43368
O	0	4.82915	-1.22537	-0.72318
H	0	5.96937	3.04641	-1.8078
H	0	4.58611	3.91716	0.03947
H	0	3.32412	2.32702	1.51578
H	0	6.14803	0.59988	-2.24703
C	0	3.44059	-0.56707	1.08701
C	0	4.04739	-1.77321	0.35185
H	0	4.74639	-2.25141	1.05154
O	0	2.64015	-0.63411	1.99113
C	0	0.37572	-0.89692	-2.25672
C	0	1.30872	-1.13506	-1.1183
C	0	2.20837	-2.36635	-1.35408
C	0	3.0373	-2.79964	-0.14329
H	0	0.70397	-0.34891	-3.13199
H	0	1.93636	-0.24739	-0.97136
H	0	0.74889	-1.28052	-0.18506
H	0	1.57228	-3.21098	-1.64414
H	0	2.87357	-2.17045	-2.20231

H	0	2.38324	-3.01669	0.71108
H	0	3.5715	-3.72372	-0.38794
H	0	-0.53837	-1.47307	-2.34394

Zero-point correction= 0.223770 (Hartree/Particle)
Thermal correction to Energy= 0.236547
Thermal correction to Enthalpy= 0.237491
Thermal correction to Gibbs Free Energy= 0.183455
Sum of electronic and zero-point Energies= -614.996785
Sum of electronic and thermal Energies= -614.984008
Sum of electronic and thermal Enthalpies= -614.983064
Sum of electronic and thermal Free Energies= -615.037100
SCF Done: E(UM062X) = -615.424540035

TS2b

C	-3.742	2.50346	1.29456
C	-4.28318	2.70752	0.0144
C	-4.52948	1.62561	-0.81841
C	-4.22099	0.35061	-0.34735
C	-3.6672	0.16477	0.91698
C	-3.42617	1.23661	1.77324
O	-3.39657	-1.13032	1.21895
H	-3.56161	3.36336	1.93309
H	-4.50989	3.71553	-0.31606
H	-4.95315	1.74827	-1.81058
H	-3.00541	1.07679	2.7594
C	-4.35725	-0.9822	-0.94801
C	-3.82541	-1.95389	0.11839
H	-4.65004	-2.57866	0.48144
O	-4.76737	-1.30633	-2.03913
C	0.58824	-2.20574	-2.37065
C	-0.43722	-2.93493	-1.56037
C	-1.55107	-2.0209	-1.04569
C	-2.68576	-2.8162	-0.40211
H	1.48575	-2.74055	-2.67105
H	-0.8942	-3.73839	-2.16397
H	0.0475	-3.44081	-0.71308
H	-1.13608	-1.29903	-0.32759
H	-1.94941	-1.43977	-1.88834
H	-3.12036	-3.49634	-1.14498
H	-2.30455	-3.42977	0.4233
H	0.2364	-1.46053	-3.08222
H	1.28123	-1.18073	-1.18836
Si	1.91376	-0.12387	-0.19352
Si	1.4267	-0.6146	2.04011
Si	1.0113	1.94101	-0.81861
Si	4.22298	-0.17948	-0.56459
C	-0.30842	-0.0257	2.50632
H	-1.08421	-0.59841	1.9849
H	-0.47664	-0.1428	3.58412
H	-0.44747	1.03275	2.25712
C	2.67897	0.29833	3.12893
H	3.70242	-0.03139	2.91823
H	2.63485	1.37947	2.95557
H	2.48039	0.11807	4.19172
C	1.54653	-2.4716	2.37521
H	2.53381	-2.86477	2.11111
H	1.36692	-2.68998	3.43407
H	0.7996	-3.01686	1.78811
C	1.3565	3.24548	0.5102
H	0.9632	4.22238	0.20599
H	0.88973	2.98046	1.46535
H	2.43267	3.35575	0.68403
C	1.80308	2.51455	-2.43893
H	1.36126	3.45725	-2.78124
H	2.8801	2.67526	-2.31472
H	1.66541	1.76998	-3.23046
C	-0.85592	1.77261	-1.06196

H	-1.08311	1.05649	-1.85977
H	-1.34394	1.4152	-0.14723
H	-1.31277	2.73264	-1.32936
C	5.0481	1.39678	0.08317
H	4.68692	2.28109	-0.45408
H	4.84652	1.54743	1.14914
H	6.13504	1.34755	-0.05064
C	4.55966	-0.3198	-2.42107
H	4.14128	0.53486	-2.96322
H	5.63631	-0.35419	-2.62307
H	4.10884	-1.22859	-2.83342
C	4.99043	-1.67387	0.30808
H	4.87031	-1.608	1.3952
H	4.51992	-2.60571	-0.02345
H	6.0632	-1.74298	0.09428

Zero-point correction= 0.571370 (Hartree/Particle)
Thermal correction to Energy= 0.610879
Thermal correction to Enthalpy= 0.611823
Thermal correction to Gibbs Free Energy= 0.498207
Sum of electronic and zero-point Energies= -2132.187594
Sum of electronic and thermal Energies= -2132.148085
Sum of electronic and thermal Enthalpies= -2132.147141
Sum of electronic and thermal Free Energies= -2132.260757
SCF Done: E(UM062X) = -2133.20458968

TS3b

C	3.71713	-0.70635	0.19675
C	3.67564	0.69734	0.19344
C	2.46332	1.36178	0.06956
C	1.30485	0.59665	-0.04715
C	1.36463	-0.79498	-0.04951
C	2.56762	-1.48045	0.07837
O	0.14796	-1.38702	-0.1931
H	4.67699	-1.20476	0.29488
H	4.59913	1.25839	0.28938
H	2.39929	2.44559	0.06429
H	2.59673	-2.56407	0.07871
C	-0.1137	0.9541	-0.20582
C	-0.83228	-0.36802	-0.19906
H	-1.45056	-0.35052	0.89825
O	-0.6377	2.05073	-0.27814
C	-2.67909	-0.03646	1.66831
C	-3.70814	-0.30486	0.60262
C	-3.21709	0.14756	-0.78289
C	-1.9482	-0.60926	-1.1929
H	-2.64473	-0.69347	2.53475
H	-3.94443	-1.37556	0.57223
H	-4.64634	0.21816	0.83844
H	-3.9985	-0.02493	-1.52953
H	-3.00536	1.22229	-0.76658
H	-2.14448	-1.68821	-1.21965
H	-1.62444	-0.30798	-2.19632
H	-2.4711	1.012	1.88468

Zero-point correction= 0.220272 (Hartree/Particle)
Thermal correction to Energy= 0.231783
Thermal correction to Enthalpy= 0.232727
Thermal correction to Gibbs Free Energy= 0.181794
Sum of electronic and zero-point Energies= -614.983499
Sum of electronic and thermal Energies= -614.971988
Sum of electronic and thermal Enthalpies= -614.971044
Sum of electronic and thermal Free Energies= -615.021977
SCF Done: E(UM062X) = -615.406233597

2a

C	0	3.44345	-1.09827	0.54105
C	0	3.45392	0.17478	1.13463
C	0	2.42492	1.0697	0.88033

C	0	1.40027	0.66719	0.02514
C	0	1.40215	-0.59905	-0.55538
C	0	2.42519	-1.51134	-0.31039
O	0	0.33548	-0.84624	-1.35517
H	0	4.25784	-1.7847	0.75376
H	0	4.27123	0.45185	1.79158
H	0	2.40034	2.06061	1.32379
H	0	2.4161	-2.49421	-0.76755
C	0	0.17913	1.33888	-0.43752
C	0	-0.50324	0.32348	-1.36859
H	0	-0.5044	0.71652	-2.39194
O	0	-0.25949	2.43242	-0.16326
C	0	-4.42433	0.00011	0.87622
C	0	-3.35887	-1.09454	0.88806
C	0	-1.96692	-0.59322	0.49772
C	0	-1.91691	-0.01239	-0.91553
H	0	-4.59726	0.38481	-0.13377
H	0	-3.65409	-1.90363	0.2072
H	0	-3.30439	-1.53789	1.88891
H	0	-1.25102	-1.42085	0.56748
H	0	-1.64407	0.17344	1.21551
H	0	-2.48864	0.92117	-0.97051
H	0	-2.36004	-0.71734	-1.62964
H	0	-4.12207	0.84438	1.50563
H	0	-5.38002	-0.37552	1.25266

Zero-point correction= 0.238463 (Hartree/Particle)
Thermal correction to Energy= 0.251043
Thermal correction to Enthalpy= 0.251987
Thermal correction to Gibbs Free Energy= 0.197206
Sum of electronic and zero-point Energies= -615.654351
Sum of electronic and thermal Energies= -615.641771
Sum of electronic and thermal Enthalpies= -615.640827
Sum of electronic and thermal Free Energies= -615.695608
SCF Done: E(RM062X) = -616.096807718

Int.2

C	0	-4.52768	5.66814	0.65119
C	0	-4.40191	5.89014	-0.7269
C	0	-4.07774	4.84999	-1.59271
C	0	-3.88298	3.58402	-1.04913
C	0	-4.01342	3.39419	0.32179
C	0	-4.33345	4.40539	1.20907
O	0	-3.78205	2.07373	0.67319
H	0	-4.78079	6.49861	1.30279
H	0	-4.55935	6.88953	-1.11917
H	0	-3.9757	5.00692	-2.66184
H	0	-4.42663	4.21821	2.27274
C	0	-3.54005	2.26045	-1.61404
C	0	-3.49594	1.40004	-0.46003
O	0	-3.33686	1.92027	-2.78538
C	0	-6.94054	-1.41191	-0.08061
C	0	-5.70844	-0.56585	0.22822
C	0	-4.56377	-0.84391	-0.74303
C	0	-3.2776	-0.0617	-0.40831
H	0	-7.75692	-1.19914	0.6152
H	0	-5.97261	0.49847	0.18814
H	0	-5.36948	-0.75918	1.25461
H	0	-4.32562	-1.91481	-0.7382
H	0	-4.86898	-0.5885	-1.76512
H	0	-2.9141	-0.33753	0.58754
H	0	-2.50451	-0.30847	-1.14359
H	0	-6.70887	-2.48005	-0.01068
H	0	-7.30361	-1.21613	-1.09503

Zero-point correction= 0.225469 (Hartree/Particle)
Thermal correction to Energy= 0.238106
Thermal correction to Enthalpy= 0.239050
Thermal correction to Gibbs Free Energy= 0.183787

Sum of electronic and zero-point Energies= -615.031640
Sum of electronic and thermal Energies= -615.019003
Sum of electronic and thermal Enthalpies= -615.018059
Sum of electronic and thermal Free Energies= -615.073322
SCF Done: E(UM062X) = -615.460239625

TS4b

C	-3.09229	3.32483	0.9193
C	-3.06292	3.52547	-0.46993
C	-2.67306	2.5021	-1.32495
C	-2.31237	1.27863	-0.76285
C	-2.34916	1.1009	0.62021
C	-2.73488	2.10786	1.49433
O	-1.97482	-0.16233	0.99476
H	-3.39879	4.14169	1.5653
H	-3.34526	4.49237	-0.87308
H	-2.63969	2.63545	-2.40206
H	-2.75482	1.94452	2.56619
C	-1.85076	0.00341	-1.33495
C	-1.51849	-0.80481	-0.1491
O	-1.65727	-0.31564	-2.50458
C	-5.33514	-3.17492	0.14589
C	-4.02949	-2.44222	0.44343
C	-2.90066	-2.89307	-0.4825
C	-1.52793	-2.29941	-0.13434
H	-6.14344	-2.8356	0.79978
H	-4.18326	-1.36124	0.33636
H	-3.73477	-2.6072	1.48811
H	-2.81771	-3.98725	-0.4509
H	-3.14359	-2.62432	-1.51811
H	-1.18938	-2.64378	0.85053
H	-0.79	-2.63423	-0.8736
H	-5.21888	-4.25489	0.28665
H	-5.64773	-3.00698	-0.89025
H	-0.10765	-0.47719	-0.09723
Si	1.49578	-0.05968	0.08546
Si	2.48928	-1.77304	-1.15942
Si	1.74333	-0.22318	2.40272
Si	1.67001	2.07143	-0.876
C	2.3157	-3.40125	-0.20859
H	2.72323	-4.23409	-0.79334
H	2.86102	-3.36215	0.74133
H	1.26878	-3.62924	0.01701
C	4.3311	-1.43397	-1.42751
H	4.80738	-2.26813	-1.95542
H	4.47895	-0.53004	-2.02797
H	4.85307	-1.29373	-0.47501
C	1.6202	-1.89005	-2.83377
H	1.80875	-2.86041	-3.30716
H	0.53705	-1.74529	-2.75152
H	1.99426	-1.11261	-3.50875
C	0.67564	-1.656	3.01833
H	-0.38152	-1.44694	2.82066
H	0.93899	-2.59518	2.51924
H	0.79971	-1.79984	4.09793
C	3.55037	-0.53537	2.8695
H	3.66171	-0.62204	3.95654
H	3.92031	-1.46345	2.41977
H	4.19478	0.28184	2.5283
C	1.14621	1.37292	3.21995
H	0.15656	1.65323	2.84116
H	1.07522	1.25186	4.30696
H	1.83073	2.20358	3.01775
C	3.48931	2.59599	-0.83063
H	3.61506	3.59729	-1.2587
H	3.8608	2.62189	0.19972
H	4.1225	1.90574	-1.39711

C	0.67299	3.35214	0.089
H	0.65139	4.29977	-0.46244
H	-0.36114	3.03426	0.25089
H	1.12589	3.54247	1.06744
C	1.08585	1.98161	-2.66934
H	1.80105	1.41365	-3.27482
H	0.11223	1.48869	-2.76627
H	1.0099	2.98761	-3.09848

Zero-point correction= 0.573804 (Hartree/Particle)
Thermal correction to Energy= 0.613226
Thermal correction to Enthalpy= 0.614170
Thermal correction to Gibbs Free Energy= 0.500530
Sum of electronic and zero-point Energies= -2132.213625
Sum of electronic and thermal Energies= -2132.174203
Sum of electronic and thermal Enthalpies= -2132.173258
Sum of electronic and thermal Free Energies= -2132.286899
SCF Done: E(UM062X) = -2133.22813466

TS1c

C	1.0183	3.26047	0.74002
C	2.18958	3.48188	0.01765
C	2.94555	2.3992	-0.42051
C	2.54969	1.09366	-0.12999
C	1.34666	0.88849	0.55545
C	0.58522	1.96291	1.00177
O	0.88754	-0.38694	0.77768
H	0.42556	4.10137	1.08654
H	2.51212	4.49442	-0.20047
H	3.86493	2.54538	-0.97971
H	-0.34074	1.76735	1.53506
C	3.43415	-0.05088	-0.53758
C	0.69909	-1.16053	-0.36145
H	1.15586	-0.75638	-1.26342
O	3.9828	-0.05191	-1.61938
C	3.72382	-1.14519	0.48194
C	3.35128	-2.57789	0.03684
C	2.02977	-3.09552	0.6206
C	0.75585	-2.62749	-0.10558
H	4.81029	-1.08938	0.61827
H	3.3451	-2.62549	-1.05968
H	4.14955	-3.24992	0.36619
H	2.03597	-4.19162	0.61179
H	1.96863	-2.79378	1.67301
H	0.67264	-3.12743	-1.0776
H	-0.11948	-2.93327	0.48603
H	3.26083	-0.9126	1.44452
Sn	-2.54064	-0.12657	-0.18338
H	-0.92625	-0.823	-0.671
C	-2.55271	-0.15137	1.96564
H	-3.187	-0.95948	2.34075
H	-1.53975	-0.30974	2.34608
H	-2.93217	0.79158	2.37077
C	-2.7075	1.89697	-0.87448
H	-1.79874	2.4623	-0.65079
H	-2.86738	1.92674	-1.9556
H	-3.55227	2.39776	-0.39209
C	-4.21953	-1.24145	-0.9147
H	-4.2615	-1.21841	-2.00679
H	-4.16619	-2.28642	-0.59824
H	-5.15247	-0.81849	-0.53032

Zero-point correction= 0.342579 (Hartree/Particle)
Thermal correction to Energy= 0.364231
Thermal correction to Enthalpy= 0.365175
Thermal correction to Gibbs Free Energy= 0.288393
Sum of electronic and zero-point Energies= -738.472019
Sum of electronic and thermal Energies= -738.450367
Sum of electronic and thermal Enthalpies= -738.449423

Sum of electronic and thermal Free Energies= -738.526205
SCF Done: E(UM062X) = -949.946732783

TS1d

C	1.5826	-3.12087	0.26237
C	2.28943	-2.97199	-0.94246
C	2.88847	-1.75999	-1.25429
C	2.76562	-0.71244	-0.34156
C	2.05554	-0.87369	0.84633
C	1.44715	-2.08208	1.17675
O	2.01738	0.23617	1.62436
H	1.12097	-4.07833	0.48578
H	2.36143	-3.80998	-1.62704
H	3.44104	-1.61162	-2.177
H	0.89998	-2.19299	2.10605
C	3.25996	0.67134	-0.3524
C	2.75486	1.28282	0.96515
H	3.60739	1.53881	1.6038
O	3.89962	1.26217	-1.19201
C	-1.46012	3.02396	-1.20898
C	-0.38984	3.25157	-0.18695
C	0.71326	2.19334	-0.23426
C	1.85612	2.49015	0.7351
H	-2.36679	3.61949	-1.15166
H	0.06839	4.24664	-0.32157
H	-0.83249	3.26237	0.81972
H	0.28226	1.21403	0.01242
H	1.10776	2.11521	-1.25568
H	2.48231	3.3033	0.35013
H	1.45847	2.80835	1.70633
H	-1.15125	2.74657	-2.21436
H	-2.11589	1.49016	-0.66547
Sn	-2.28065	-0.20719	-0.05855
C	-4.30361	-0.91319	-0.0985
C	-1.06495	-1.43083	-1.32904
C	-1.54978	-0.30576	1.95599
H	-1.06978	-2.47284	-0.99644
H	-1.42699	-1.39871	-2.36048
H	-0.02654	-1.08339	-1.31948
H	-4.71592	-0.86889	-1.10988
H	-4.35314	-1.95155	0.24253
H	-4.94117	-0.31146	0.55448
H	-1.57075	-1.33791	2.3198
H	-2.17183	0.29811	2.62271
H	-0.52064	0.06151	2.02268

Zero-point correction= 0.340506 (Hartree/Particle)
Thermal correction to Energy= 0.362289
Thermal correction to Enthalpy= 0.363233
Thermal correction to Gibbs Free Energy= 0.286998
Sum of electronic and zero-point Energies= -738.480605
Sum of electronic and thermal Energies= -738.458822
Sum of electronic and thermal Enthalpies= -738.457878
Sum of electronic and thermal Free Energies= -738.534113
SCF Done: E(UM062X) = -949.954760189

Me3SnH

Sn	1.48131	-0.93915	0.79657
H	2.05471	-0.12891	-0.60928
C	2.20323	0.08242	2.5279
H	1.85073	1.11696	2.54644
H	1.85525	-0.41044	3.4397
H	3.29609	0.09643	2.54649
C	2.20283	-2.94945	0.77964
H	1.84805	-3.49421	1.65867
H	1.85722	-3.48107	-0.11076
H	3.29569	-2.9721	0.78524
C	-0.65454	-0.92799	0.78021

H	-1.04002	0.09488	0.78152
H	-1.04128	-1.4348	-0.10774
H	-1.04946	-1.44006	1.66177

Zero-point correction= 0.116317 (Hartree/Particle)
Thermal correction to Energy= 0.125095
Thermal correction to Enthalpy= 0.126039
Thermal correction to Gibbs Free Energy= 0.081875
Sum of electronic and zero-point Energies= -123.487824
Sum of electronic and thermal Energies= -123.479046
Sum of electronic and thermal Enthalpies= -123.478102
Sum of electronic and thermal Free Energies= -123.522266
SCF Done: E(RM062X) = -334.528661225

Me3Sn•

Sn	-2.68856	-0.27408	-0.26207
C	-2.06676	-1.71767	1.21683
H	-1.77077	-2.65877	0.7474
H	-1.22161	-1.3411	1.79812
H	-2.89467	-1.92019	1.90404
C	-3.27033	1.52749	0.77323
H	-3.6369	2.28202	0.07339
H	-4.07043	1.29898	1.48469
H	-2.42868	1.95066	1.32673
C	-4.43117	-1.05742	-1.26632
H	-4.80377	-0.35288	-2.01359
H	-4.20365	-2.00181	-1.76612
H	-5.22572	-1.2369	-0.53463

Zero-point correction= 0.108520 (Hartree/Particle)
Thermal correction to Energy= 0.117083
Thermal correction to Enthalpy= 0.118027
Thermal correction to Gibbs Free Energy= 0.073297
Sum of electronic and zero-point Energies= -122.866900
Sum of electronic and thermal Energies= -122.858337
Sum of electronic and thermal Enthalpies= -122.857393
Sum of electronic and thermal Free Energies= -122.902123
SCF Done: E(UM062X) = -333.910021012

Int.0

C	0	3.40797	-1.03119	-0.65187
C	0	3.81356	0.28883	-0.44764
C	0	2.90792	1.21619	0.07388
C	0	1.64046	0.77224	0.36878
C	0	1.20196	-0.52377	0.18415
C	0	2.11634	-1.44286	-0.34319
O	0	-0.05286	-0.9624	0.48068
H	0	4.10908	-1.75271	-1.05932
H	0	4.82552	0.59573	-0.69199
H	0	3.19328	2.25053	0.24096
H	0	1.79294	-2.46807	-0.49648
C	0	-1.28011	1.11066	0.07954
C	0	-0.92484	-0.0037	1.07131
H	0	-0.45317	0.44902	1.95228
O	0	-1.18809	2.27776	0.38087
C	0	-1.8878	0.60762	-1.21064
C	0	-3.16373	-0.18707	-0.85975
C	0	-2.87377	-1.3036	0.14731
C	0	-2.21056	-0.75542	1.41606
H	0	-2.10255	1.46045	-1.85812
H	0	-3.90237	0.50553	-0.43579
H	0	-3.60129	-0.59911	-1.77437
H	0	-3.80256	-1.8202	0.41018
H	0	-2.2076	-2.04456	-0.30808
H	0	-2.88696	-0.05875	1.92713
H	0	-1.97116	-1.56193	2.1165
H	0	-1.17195	-0.05532	-1.71185

Zero-point correction= 0.227140 (Hartree/Particle)
Thermal correction to Energy= 0.238839

Thermal correction to Enthalpy= 0.239783
Thermal correction to Gibbs Free Energy= 0.187753
Sum of electronic and zero-point Energies= -614.974515
Sum of electronic and thermal Energies= -614.962815
Sum of electronic and thermal Enthalpies= -614.961871
Sum of electronic and thermal Free Energies= -615.013901
SCF Done: E(UM062X) = -615.405916367

B_ts1

C	-3.43002	-0.70568	0.48412
C	-3.48312	0.67077	0.26038
C	-2.33062	1.36257	-0.13551
C	-1.19187	0.61346	-0.27626
C	-1.0989	-0.75097	-0.08137
C	-2.25008	-1.42974	0.31672
O	0.09682	-1.36678	-0.24989
H	-4.32923	-1.23118	0.78912
H	-4.41585	1.20906	0.39319
H	-2.33769	2.43265	-0.31672
H	-2.20779	-2.50177	0.48025
C	0.91214	0.92989	-0.46143
C	0.9874	-0.52012	-0.98277
H	0.69135	-0.50839	-2.03902
O	1.012	1.86653	-1.2592
C	1.31651	1.07589	1.00094
C	2.76589	0.56747	1.12417
C	2.8736	-0.88801	0.65922
C	2.40568	-1.04534	-0.79071
H	1.2286	2.12837	1.28134
H	3.41913	1.20146	0.51078
H	3.1022	0.66434	2.16142
H	3.90618	-1.24014	0.75153
H	2.25637	-1.52356	1.30534
H	3.0596	-0.4746	-1.46134
H	2.4403	-2.09336	-1.10639
H	0.67002	0.47729	1.65061

Zero-point correction= 0.226451 (Hartree/Particle)
Thermal correction to Energy= 0.237172
Thermal correction to Enthalpy= 0.238116
Thermal correction to Gibbs Free Energy= 0.189529
Sum of electronic and zero-point Energies= -614.959198
Sum of electronic and thermal Energies= -614.948477
Sum of electronic and thermal Enthalpies= -614.947533
Sum of electronic and thermal Free Energies= -614.996120
SCF Done: E(UM062X) = -615.395520387

B_ts3

C	3.15797	3.0375	0.00072
C	2.14125	3.94134	0.2817
C	0.808	3.50993	0.23747
C	0.56517	2.20153	-0.0934
C	1.5535	1.27425	-0.38077
C	2.88315	1.70671	-0.32895
O	1.15238	0.00056	-0.70081
H	4.1938	3.35942	0.03854
H	2.3746	4.97022	0.53716
H	-0.00785	4.193	0.45834
H	3.70646	1.02827	-0.52466
C	2.93989	-1.2956	0.32893
C	2.17032	-0.95731	-0.95367
H	2.87314	-0.5895	-1.71079
O	4.14883	-1.28322	0.3649
C	2.05525	-1.76846	1.45826
C	1.30179	-3.03087	0.98831
C	0.55231	-2.79013	-0.325
C	1.47408	-2.23885	-1.41634
H	2.67207	-1.9658	2.3374

H	2.0298	-3.84108	0.85298
H	0.60499	-3.35296	1.76919
H	0.09034	-3.72171	-0.66893
H	-0.25988	-2.07623	-0.15607
H	2.25302	-2.97247	-1.66114
H	0.91641	-2.03121	-2.33551
H	1.3341	-0.97539	1.69337
Sn	-2.34873	0.12936	0.06809
H	-1.16618	1.43211	0.13912
C	-4.33817	0.89756	0.23017
H	-4.4915	1.38356	1.19738
H	-4.54285	1.63005	-0.55494
H	-5.06872	0.08878	0.13746
C	-2.00638	-1.218	1.70078
H	-0.95384	-1.22578	1.99724
H	-2.59978	-0.92238	2.57033
H	-2.28944	-2.23891	1.42604
C	-2.09487	-0.85479	-1.81348
H	-2.76953	-0.43814	-2.56605
H	-1.06555	-0.71217	-2.15239
H	-2.29118	-1.92809	-1.73297

Zero-point correction= 0.343188 (Hartree/Particle)
Thermal correction to Energy= 0.364633
Thermal correction to Enthalpy= 0.365577
Thermal correction to Gibbs Free Energy= 0.290119
Sum of electronic and zero-point Energies= -738.458029
Sum of electronic and thermal Energies= -738.436584
Sum of electronic and thermal Enthalpies= -738.435639
Sum of electronic and thermal Free Energies= -738.511097
SCF Done: E(UM062X) = -949.934383091

B_Pa

C	0	3.0709	3.78499	-0.40486
C	0	1.77014	4.2359	-0.2233
C	0	0.72011	3.31664	-0.24246
C	0	0.97175	1.96738	-0.44001
C	0	2.28463	1.52329	-0.62216
C	0	3.34398	2.43059	-0.60536
O	0	2.42308	0.17502	-0.81523
H	0	3.8975	4.48878	-0.38958
H	0	1.57162	5.29101	-0.06667
H	0	-0.3024	3.65408	-0.10261
H	0	4.36953	2.10493	-0.73384
C	0	4.56872	-0.25907	0.26113
C	0	3.72554	-0.34703	-1.01801
H	0	4.24217	0.18873	-1.82417
O	0	5.67878	0.22201	0.25522
C	0	3.94332	-0.94152	1.45473
C	0	3.72365	-2.42675	1.09961
C	0	2.90085	-2.58065	-0.18278
C	0	3.54363	-1.82935	-1.35341
H	0	4.59977	-0.81865	2.3187
H	0	4.70268	-2.90519	0.96656
H	0	3.23164	-2.93411	1.93524
H	0	2.79905	-3.64057	-0.43736
H	0	1.89124	-2.18743	-0.02076
H	0	4.53159	-2.25111	-1.57771
H	0	2.9329	-1.91096	-2.25822
H	0	2.97486	-0.47157	1.66586
H	0	0.17201	1.23431	-0.46302

Zero-point correction= 0.240362 (Hartree/Particle)
Thermal correction to Energy= 0.251998
Thermal correction to Enthalpy= 0.252942
Thermal correction to Gibbs Free Energy= 0.202050
Sum of electronic and zero-point Energies= -615.646501
Sum of electronic and thermal Energies= -615.634865
Sum of electronic and thermal Enthalpies= -615.633921

Sum of electronic and thermal Free Energies= -615.684813
SCF Done: E(RM062X) = -616.091730296

A_Int.1

C	0	-3.56123	-0.44936	0.33727
C	0	-3.29613	0.90799	0.15003
C	0	-1.99476	1.34517	-0.09729
C	0	-0.98245	0.39985	-0.15415
C	0	-1.23844	-0.95896	0.02684
C	0	-2.53474	-1.39307	0.27654
H	0	-4.58003	-0.7755	0.52447
H	0	-4.1104	1.62519	0.19187
H	0	-1.77199	2.39648	-0.25925
H	0	-2.75075	-2.44966	0.41245
C	0	0.50369	0.61711	-0.36747
C	0	0.98476	-0.78867	-0.84244
H	0	0.71694	-0.83841	-1.90592
O	0	0.85616	1.62131	-1.22232
C	0	1.16999	1.01501	0.99467
C	0	2.68348	0.82875	0.98731
C	0	3.03684	-0.6242	0.66902
C	0	2.48757	-1.02006	-0.70268
H	0	0.87855	2.04578	1.22168
H	0	3.13284	1.49227	0.23917
H	0	3.08714	1.11839	1.96381
H	0	4.12236	-0.76762	0.68679
H	0	2.62125	-1.27635	1.44976
H	0	2.99608	-0.41698	-1.46537
H	0	2.71681	-2.06927	-0.9247
H	0	0.71428	0.37355	1.75966
C	0	0.04353	-1.75746	-0.09811
H	0	0.44257	-2.00768	0.89515
H	0	-0.09024	-2.70062	-0.63739

Zero-point correction= 0.252276 (Hartree/Particle)
Thermal correction to Energy= 0.263210
Thermal correction to Enthalpy= 0.264155
Thermal correction to Gibbs Free Energy= 0.215509
Sum of electronic and zero-point Energies= -579.081481
Sum of electronic and thermal Energies= -579.070546
Sum of electronic and thermal Enthalpies= -579.069602
Sum of electronic and thermal Free Energies= -579.118248
SCF Done: E(UM062X) = -579.518197411

A_TS1a

C	3.54845	-0.51836	-0.29478
C	3.3595	0.84034	-0.04833
C	2.07602	1.34088	0.16516
C	0.99056	0.47309	0.11907
C	1.17812	-0.89235	-0.11273
C	2.45955	-1.38986	-0.31851
H	4.55056	-0.90552	-0.45161
H	4.21475	1.50812	-0.01508
H	1.91148	2.39434	0.37241
H	2.61338	-2.45396	-0.47889
C	-0.43982	0.94701	0.27654
C	-1.0203	-0.95145	0.88157
H	-0.6266	-0.85111	1.892
O	-0.78614	1.67246	1.22784
C	-1.21902	1.03285	-1.04934
C	-2.72605	0.88217	-0.87144
C	-3.11044	-0.55956	-0.54222
C	-2.51633	-1.02058	0.79342
H	-0.97623	2.02285	-1.45756
H	-3.04777	1.55045	-0.06364
H	-3.23893	1.20073	-1.78507
H	-4.2001	-0.65705	-0.49006
H	-2.77716	-1.22068	-1.35387

H	-2.9297	-0.40074	1.59734
H	-2.83188	-2.05631	0.99928
H	-0.83682	0.29728	-1.76715
C	-0.09412	-1.71076	-0.03392
H	0.11143	-2.71261	0.37019
H	-0.54181	-1.85692	-1.02588

Zero-point correction= 0.250156 (Hartree/Particle)
Thermal correction to Energy= 0.261163
Thermal correction to Enthalpy= 0.262107
Thermal correction to Gibbs Free Energy= 0.213256
Sum of electronic and zero-point Energies= -579.061613
Sum of electronic and thermal Energies= -579.050606
Sum of electronic and thermal Enthalpies= -579.049661
Sum of electronic and thermal Free Energies= -579.098512
SCF Done: E(UM062X) = -579.507244123

A_Int.a

C	0	3.50393	-0.75445	-0.27257
C	0	3.44491	0.61405	-0.0281
C	0	2.20722	1.22819	0.13258
C	0	1.02984	0.48838	0.02365
C	0	1.08165	-0.8971	-0.20187
C	0	2.33012	-1.50122	-0.34167
H	0	4.4644	-1.24696	-0.39094
H	0	4.35723	1.19753	0.04633
H	0	2.13655	2.2914	0.34312
H	0	2.3825	-2.577	-0.49121
C	0	-0.27084	1.23531	0.16163
C	0	-1.1203	-1.28897	0.89336
H	0	-0.68298	-1.1646	1.87969
O	0	-0.45536	2.00361	1.0823
C	0	-1.27499	1.14735	-0.98338
C	0	-2.73261	0.93826	-0.54605
C	0	-3.15931	-0.53014	-0.45392
C	0	-2.60859	-1.28823	0.76482
H	0	-1.18432	2.12175	-1.48172
H	0	-2.88001	1.44437	0.4161
H	0	-3.38561	1.43845	-1.26876
H	0	-4.25337	-0.58128	-0.4101
H	0	-2.87128	-1.04554	-1.38039
H	0	-3.03668	-0.84766	1.67265
H	0	-2.99029	-2.32307	0.72063
H	0	-0.96933	0.39766	-1.71974
C	0	-0.18973	-1.72995	-0.19325
H	0	0.09444	-2.78659	-0.06224
H	0	-0.69094	-1.68165	-1.16965

Zero-point correction= 0.250208 (Hartree/Particle)
Thermal correction to Energy= 0.262193
Thermal correction to Enthalpy= 0.263137
Thermal correction to Gibbs Free Energy= 0.211980
Sum of electronic and zero-point Energies= -579.076144
Sum of electronic and thermal Energies= -579.064159
Sum of electronic and thermal Enthalpies= -579.063215
Sum of electronic and thermal Free Energies= -579.114372
SCF Done: E(UM062X) = -579.515894373

A_TS2a

C	2.9359	3.27065	0.6278
C	3.8867	3.04195	-0.36224
C	4.21185	1.7347	-0.70839
C	3.61352	0.65458	-0.05684
C	2.63322	0.87666	0.92723
C	2.31078	2.19419	1.25391
H	2.66269	4.28574	0.90018
H	4.36354	3.87461	-0.86913
H	4.93195	1.53025	-1.49501
H	1.5387	2.37414	1.999

C	4.01938	-0.72173	-0.51387
C	1.3595	-1.24102	0.506
H	1.58188	-0.99875	-0.53122
O	4.11968	-0.96338	-1.69919
C	4.4301	-1.77126	0.51175
C	3.74212	-3.14268	0.34595
C	2.53841	-3.3771	1.26801
C	1.22357	-2.69847	0.83302
H	5.50596	-1.89384	0.33298
H	3.45851	-3.26424	-0.70709
H	4.4822	-3.92089	0.55742
H	2.34893	-4.45456	1.33977
H	2.80409	-3.05199	2.283
H	0.83313	-3.21858	-0.05108
H	0.48092	-2.8403	1.63278
H	4.32928	-1.39442	1.53349
H	-0.2041	-0.73774	0.39802
Si	-1.59712	-0.07169	0.01493
Si	-2.70366	-1.76451	-1.16047
Si	-1.0629	1.7208	-1.39996
Si	-2.83378	0.65587	1.85692
C	-1.79473	-2.09604	-2.7875
H	-2.26051	-2.92321	-3.33531
H	-1.79887	-1.21598	-3.4397
H	-0.74891	-2.3637	-2.59825
C	-4.49802	-1.27826	-1.51666
H	-4.99932	-2.04823	-2.11438
H	-5.05971	-1.1637	-0.5825
H	-4.55819	-0.33117	-2.06297
C	-2.70782	-3.36022	-0.14106
H	-3.21875	-4.16517	-0.68191
H	-1.68611	-3.69128	0.07262
H	-3.21934	-3.21933	0.81694
C	0.54258	1.35081	-2.32404
H	1.39642	1.34242	-1.6361
H	0.50425	0.37603	-2.82288
H	0.73689	2.113	-3.08747
C	-2.4762	1.97007	-2.63839
H	-2.26575	2.8165	-3.30247
H	-2.62642	1.08309	-3.26364
H	-3.419	2.17529	-2.11846
C	-0.85185	3.33588	-0.43584
H	-0.02227	3.25897	0.27363
H	-0.62631	4.15893	-1.12432
H	-1.75673	3.60258	0.12096
C	-4.2666	1.72904	1.23724
H	-4.89694	2.05584	2.07248
H	-3.90304	2.625	0.72252
H	-4.89854	1.17582	0.53394
C	-1.75865	1.69193	3.02222
H	-2.3619	2.12224	3.82998
H	-0.9719	1.08222	3.47993
H	-1.27794	2.51691	2.48451
C	-3.55816	-0.80559	2.81575
H	-4.26027	-1.37142	2.1931
H	-2.77558	-1.49569	3.14874
H	-4.10253	-0.45899	3.70179
C	1.85611	-0.26637	1.54229
H	2.46158	-0.78843	2.29804
H	1.00605	0.15697	2.10263

Zero-point correction= 0.598426 (Hartree/Particle)
Thermal correction to Energy= 0.637492
Thermal correction to Enthalpy= 0.638437
Thermal correction to Gibbs Free Energy= 0.525452
Sum of electronic and zero-point Energies= -2096.239971
Sum of electronic and thermal Energies= -2096.200904
Sum of electronic and thermal Enthalpies= -2096.199960

Sum of electronic and thermal Free Energies= -2096.312945
SCF Done: E(UM062X) = -2097.28628538

A_Pb

C	0	2.60739	3.24063	0.76391
C	0	3.67454	3.19062	-0.12743
C	0	4.14595	1.9554	-0.55608
C	0	3.58342	0.77004	-0.07791
C	0	2.49039	0.81098	0.8077
C	0	2.01811	2.06107	1.20987
H	0	2.2211	4.19781	1.10126
H	0	4.12862	4.10579	-0.49394
H	0	4.96224	1.88738	-1.26896
H	0	1.16316	2.10714	1.87978
C	0	4.18267	-0.50754	-0.60072
C	0	1.33014	-1.35549	0.10842
H	0	1.96151	-1.19606	-0.77229
O	0	4.46376	-0.61022	-1.77756
C	0	4.54937	-1.641	0.35119
C	0	3.90682	-3.00914	0.01785
C	0	2.69908	-3.40254	0.88424
C	0	1.33731	-2.84656	0.44361
H	0	5.63475	-1.73033	0.22368
H	0	3.64835	-3.02583	-1.04867
H	0	4.67528	-3.77613	0.15372
H	0	2.61973	-4.49578	0.89324
H	0	2.90822	-3.11879	1.92415
H	0	1.00536	-3.39488	-0.44672
H	0	0.60031	-3.05777	1.22916
H	0	4.38173	-1.36505	1.39571
C	0	1.78674	-0.44671	1.26737
H	0	2.44361	-1.00764	1.94129
H	0	0.9208	-0.16514	1.87593
H	0	0.32482	-1.0544	-0.20691

Zero-point correction= 0.265159 (Hartree/Particle)
Thermal correction to Energy= 0.276884
Thermal correction to Enthalpy= 0.277829
Thermal correction to Gibbs Free Energy= 0.227961
Sum of electronic and zero-point Energies= -579.718617
Sum of electronic and thermal Energies= -579.706891
Sum of electronic and thermal Enthalpies= -579.705947
Sum of electronic and thermal Free Energies= -579.755815
SCF Done: E(RM062X) = -580.172700418

A_TS1b

C	-3.49579	-0.46006	0.44274
C	-3.28785	0.88929	0.14304
C	-2.02411	1.33822	-0.2328
C	-0.99205	0.41182	-0.29914
C	-1.18704	-0.93655	0.00006
C	-2.45001	-1.38178	0.37333
H	-4.48744	-0.79666	0.72984
H	-4.119	1.58554	0.1981
H	-1.8338	2.37826	-0.48271
H	-2.62586	-2.42939	0.60323
C	0.43813	0.64191	-0.69144
C	1.0234	-0.77149	-0.93726
H	0.81151	-0.94262	-2.00299
O	0.88107	1.67152	-1.2349
C	1.13551	0.99083	1.21371
C	2.6202	0.85627	1.09885
C	3.02975	-0.55287	0.66601
C	2.53363	-0.88324	-0.74319
H	0.72032	1.98735	1.33505
H	2.99454	1.59767	0.38408
H	3.07163	1.09157	2.07526
H	4.12073	-0.64521	0.69069

H	2.64058	-1.27999	1.39156
H	3.00213	-0.18616	-1.44933
H	2.86068	-1.89105	-1.02536
H	0.61589	0.22128	1.78461
C	0.1072	-1.71636	-0.13522
H	0.52758	-1.93136	0.857
H	-0.03714	-2.68058	-0.63255

Zero-point correction= 0.249982 (Hartree/Particle)
Thermal correction to Energy= 0.260976
Thermal correction to Enthalpy= 0.261920
Thermal correction to Gibbs Free Energy= 0.213150
Sum of electronic and zero-point Energies= -579.058777
Sum of electronic and thermal Energies= -579.047783
Sum of electronic and thermal Enthalpies= -579.046839
Sum of electronic and thermal Free Energies= -579.095609
SCF Done: E(UM062X) = -579.503144115

A_Int.b

C	0	3.45526	0.82647	-0.47817
C	0	3.3449	0.50123	0.87965
C	0	2.19639	-0.11627	1.35815
C	0	1.179	-0.40017	0.45093
C	0	1.275	-0.0729	-0.89975
C	0	2.42693	0.54734	-1.37573
H	0	4.36082	1.3074	-0.8367
H	0	4.16299	0.73277	1.55445
H	0	2.07855	-0.38367	2.40418
H	0	2.52568	0.81092	-2.42529
C	0	-0.12411	-1.06175	0.71048
C	0	-0.79578	-1.29978	-0.65113
H	0	-0.58771	-2.35965	-0.85864
O	0	-0.57507	-1.3863	1.7854
C	0	-2.85703	2.55496	0.53714
C	0	-2.22214	1.20718	0.48754
C	0	-2.83249	0.31183	-0.61003
C	0	-2.31805	-1.1309	-0.60742
H	0	-2.54042	3.34048	-0.13905
H	0	-1.14284	1.31518	0.30729
H	0	-2.32363	0.69496	1.45226
H	0	-3.92069	0.28701	-0.4767
H	0	-2.6628	0.78038	-1.58789
H	0	-2.65867	-1.63387	0.30614
H	0	-2.76661	-1.66615	-1.45204
H	0	-3.79371	2.69879	1.06314
C	0	0.01486	-0.44919	-1.65278
H	0	0.23597	-0.98839	-2.57907
H	0	-0.5299	0.45904	-1.93719

Zero-point correction= 0.248196 (Hartree/Particle)
Thermal correction to Energy= 0.261061
Thermal correction to Enthalpy= 0.262005
Thermal correction to Gibbs Free Energy= 0.208210
Sum of electronic and zero-point Energies= -579.077115
Sum of electronic and thermal Energies= -579.064250
Sum of electronic and thermal Enthalpies= -579.063305
Sum of electronic and thermal Free Energies= -579.117101
SCF Done: E(UM062X) = -579.514619379

A_TS2b

C	-5.87432	-2.2221	-0.23141
C	-4.99709	-2.51139	0.82274
C	-4.14673	-1.52852	1.3113
C	-4.19621	-0.26853	0.71944
C	-5.06501	0.03037	-0.32579
C	-5.9187	-0.95667	-0.8121
H	-6.53218	-3.00238	-0.60289
H	-4.98579	-3.508	1.25236
H	-3.44954	-1.71791	2.12248

H	-6.60557	-0.74911	-1.62787
C	-3.38162	0.92551	1.04738
C	-3.67043	1.98849	-0.01785
H	-3.83637	2.9505	0.47849
O	-2.5998	1.03594	1.96671
C	1.2131	3.29407	-0.71314
C	-0.11596	2.99195	-1.33143
C	-1.15605	2.54481	-0.30263
C	-2.46513	2.11974	-0.9672
H	2.02756	3.60981	-1.36056
H	-0.50238	3.87151	-1.8732
H	0.00815	2.20839	-2.0952
H	-0.74644	1.72314	0.2993
H	-1.3452	3.3655	0.40274
H	-2.73359	2.85313	-1.73975
H	-2.32273	1.16106	-1.48942
H	1.22934	3.71512	0.29025
H	1.7404	1.7055	-0.41182
Si	2.16721	0.19869	-0.13996
Si	0.66477	-1.33283	-1.07626
Si	2.178	-0.03098	2.18687
Si	4.30105	-0.11064	-1.04888
C	-0.87809	-1.50409	0.00439
H	-1.34543	-0.53036	0.18375
H	-1.62452	-2.15565	-0.46565
H	-0.63049	-1.92561	0.98556
C	1.52341	-3.02024	-1.1729
H	2.37727	-2.99269	-1.85963
H	1.89518	-3.33346	-0.19099
H	0.82962	-3.78907	-1.53272
C	0.12857	-0.84775	-2.82559
H	0.99215	-0.65998	-3.47229
H	-0.46537	-1.65053	-3.27801
H	-0.4858	0.05875	-2.81731
C	2.27514	-1.85746	2.68097
H	2.30359	-1.95954	3.7721
H	1.40868	-2.41987	2.31655
H	3.1763	-2.33035	2.27539
C	3.6858	0.8573	2.91208
H	3.66906	0.81958	4.00747
H	4.6211	0.39515	2.57624
H	3.70528	1.91028	2.61103
C	0.60573	0.73722	2.89281
H	0.53395	1.79721	2.62351
H	-0.29919	0.25083	2.51328
H	0.59104	0.66752	3.98697
C	5.13045	-1.62505	-0.27194
H	5.2815	-1.48209	0.80396
H	4.52288	-2.52619	-0.4076
H	6.11187	-1.80724	-0.72493
C	5.37935	1.40938	-0.72146
H	5.50457	1.5838	0.35232
H	6.3749	1.28188	-1.16199
H	4.92955	2.30846	-1.15546
C	4.18192	-0.37337	-2.92067
H	3.58984	-1.26344	-3.16112
H	3.70657	0.48512	-3.40736
H	5.17697	-0.50324	-3.36155
C	-4.9261	1.47496	-0.75969
H	-4.82286	1.56261	-1.84705
H	-5.8198	2.04217	-0.47617

Zero-point correction= 0.595681 (Hartree/Particle)
Thermal correction to Energy= 0.635433
Thermal correction to Enthalpy= 0.636377
Thermal correction to Gibbs Free Energy= 0.521259
Sum of electronic and zero-point Energies= -2096.246632
Sum of electronic and thermal Energies= -2096.206880

Sum of electronic and thermal Enthalpies= -2096.205935
Sum of electronic and thermal Free Energies= -2096.321054
SCF Done: E(UM062X) = -2097.29120247

A_TS3b

C	3.78442	-0.60092	0.28244
C	3.63737	0.78994	0.34489
C	2.38773	1.37097	0.16107
C	1.30401	0.53193	-0.08061
C	1.44334	-0.85301	-0.15541
C	2.69417	-1.43255	0.03202
H	4.76712	-1.03902	0.42997
H	4.50496	1.41178	0.54211
H	2.24024	2.44599	0.20586
H	2.8241	-2.51013	-0.01918
C	-0.11897	0.90252	-0.30058
C	-0.89856	-0.37826	-0.35747
H	-1.40284	-0.42095	0.7954
O	-0.57289	2.02868	-0.38639
C	-2.50612	-0.2287	1.74664
C	-3.66382	-0.31528	0.78799
C	-3.32041	0.33176	-0.56316
C	-2.14263	-0.39041	-1.23075
H	-2.38921	-1.01201	2.49307
H	-3.93966	-1.36521	0.62777
H	-4.54859	0.18067	1.21338
H	-4.19375	0.30294	-1.22298
H	-3.05196	1.38302	-0.41257
H	-2.42554	-1.43185	-1.43511
H	-1.92007	0.0795	-2.19746
H	-2.24999	0.77014	2.10192
C	0.11832	-1.5138	-0.47149
H	0.12831	-1.92253	-1.49149
H	-0.10172	-2.34998	0.20264

Zero-point correction= 0.244478 (Hartree/Particle)
Thermal correction to Energy= 0.256324
Thermal correction to Enthalpy= 0.257268
Thermal correction to Gibbs Free Energy= 0.205551
Sum of electronic and zero-point Energies= -579.058101
Sum of electronic and thermal Energies= -579.046255
Sum of electronic and thermal Enthalpies= -579.045311
Sum of electronic and thermal Free Energies= -579.097028
SCF Done: E(UM062X) = -579.497248467

A_Int.2

C	0	-3.08368	3.73795	0.81149
C	0	-3.08722	3.65273	-0.58432
C	0	-2.78732	2.44982	-1.21638
C	0	-2.48687	1.35009	-0.4219
C	0	-2.48235	1.42744	0.97149
C	0	-2.78203	2.62819	1.60157
H	0	-3.31998	4.6847	1.28824
H	0	-3.32441	4.53292	-1.17387
H	0	-2.78238	2.35383	-2.29809
H	0	-2.78335	2.70745	2.68536
C	0	-2.13652	-0.03574	-0.84294
C	0	-1.90518	-0.78891	0.37697
O	0	-2.06772	-0.47187	-1.98949
C	0	-5.15742	-3.8325	0.14688
C	0	-4.01695	-2.85841	-0.1332
C	0	-2.81497	-3.09566	0.7798
C	0	-1.59275	-2.23828	0.39041
H	0	-6.01603	-3.64117	-0.50273
H	0	-3.69512	-2.93708	-1.17917
H	0	-4.37204	-1.8271	-0.0062
H	0	-3.09595	-2.89184	1.8221
H	0	-2.52179	-4.15205	0.73749

H	0	-0.77094	-2.44253	1.08907
H	0	-1.2647	-2.51963	-0.61675
H	0	-5.49723	-3.75095	1.18524
H	0	-4.83667	-4.86693	-0.01612
C	0	-2.12519	0.08249	1.57726
H	0	-1.2279	0.13672	2.21009
H	0	-2.93035	-0.3048	2.21907

Zero-point correction= 0.249271 (Hartree/Particle)
Thermal correction to Energy= 0.262179
Thermal correction to Enthalpy= 0.263123
Thermal correction to Gibbs Free Energy= 0.208152
Sum of electronic and zero-point Energies= -579.104281
Sum of electronic and thermal Energies= -579.091373
Sum of electronic and thermal Enthalpies= -579.090429
Sum of electronic and thermal Free Energies= -579.145400
SCF Done: E(UM062X) = -579.543025168

A_TS4b

C	-3.27983	3.5548	0.80061
C	-3.28759	3.4664	-0.59608
C	-2.90428	2.28721	-1.22747
C	-2.51687	1.21363	-0.43134
C	-2.50943	1.29291	0.96262
C	-2.8907	2.47262	1.59108
H	-3.57741	4.48409	1.27712
H	-3.59041	4.32632	-1.18505
H	-2.89626	2.18939	-2.30917
H	-2.88514	2.55654	2.67436
C	-2.04383	-0.12947	-0.85711
C	-1.62754	-0.82963	0.36656
O	-1.941	-0.54205	-2.00708
C	-5.14847	-3.58709	0.06314
C	-3.93222	-2.69825	-0.18024
C	-2.75654	-3.07133	0.72272
C	-1.45817	-2.31782	0.387
H	-5.9917	-3.29447	-0.56862
H	-3.61716	-2.75825	-1.22935
H	-4.20584	-1.64816	-0.00941
H	-3.03009	-2.89124	1.77139
H	-2.55961	-4.14787	0.63949
H	-0.67797	-2.59453	1.10974
H	-1.10868	-2.62206	-0.60861
H	-5.47465	-3.5275	1.10744
H	-4.9155	-4.63548	-0.15144
H	-0.14539	-0.39525	0.2621
Si	1.41452	-0.03306	0.03889
Si	2.18604	-1.69654	-1.42188
Si	2.39222	-0.18755	2.15681
Si	1.43219	2.10036	-0.92507
C	2.26048	-3.34522	-0.49425
H	2.63818	-4.13888	-1.1492
H	2.92876	-3.27967	0.37199
H	1.27153	-3.64748	-0.13454
C	3.93683	-1.25663	-1.99548
H	4.35901	-2.07019	-2.59698
H	3.92895	-0.35443	-2.61677
H	4.60953	-1.07718	-1.15017
C	1.06726	-1.84301	-2.93386
H	1.31338	-2.74474	-3.50674
H	0.00665	-1.87125	-2.66464
H	1.20438	-0.97931	-3.59355
C	1.62088	-1.64013	3.0936
H	0.55185	-1.4739	3.26702
H	1.72445	-2.57194	2.52672
H	2.10125	-1.78119	4.06855
C	4.2554	-0.47881	1.99616
H	4.72739	-0.53742	2.98374

H	4.46585	-1.41611	1.46903
H	4.73617	0.33309	1.43958
C	2.10187	1.40921	3.12879
H	1.03456	1.65016	3.18965
H	2.49002	1.32503	4.15027
H	2.60586	2.25414	2.64618
C	3.23298	2.67734	-1.04995
H	3.29365	3.64569	-1.5603
H	3.67423	2.79819	-0.05396
H	3.85042	1.96394	-1.60551
C	0.49212	3.36573	0.11398
H	0.60533	4.36241	-0.32946
H	-0.57653	3.13825	0.16164
H	0.8779	3.40795	1.13809
C	0.68401	1.97634	-2.65726
H	1.39839	1.50932	-3.345
H	-0.22705	1.36708	-2.66385
H	0.44087	2.97011	-3.05082
C	-2.05551	-0.01888	1.56971
H	-1.24091	0.11849	2.29452
H	-2.8789	-0.51144	2.10654

Zero-point correction= 0.597804 (Hartree/Particle)
Thermal correction to Energy= 0.637344
Thermal correction to Enthalpy= 0.638288
Thermal correction to Gibbs Free Energy= 0.525169
Sum of electronic and zero-point Energies= -2096.274080
Sum of electronic and thermal Energies= -2096.234541
Sum of electronic and thermal Enthalpies= -2096.233596
Sum of electronic and thermal Free Energies= -2096.346716
SCF Done: E(UM062X) = -2097.31678229

2q

C	0	3.58295	-1.17011	0.53659
C	0	3.55136	-0.8507	-0.82714
C	0	2.46444	-0.16864	-1.35803
C	0	1.42906	0.18444	-0.49658
C	0	1.446	-0.1359	0.85794
C	0	2.53611	-0.82159	1.38731
H	0	4.44101	-1.70176	0.93769
H	0	4.38166	-1.13742	-1.46472
H	0	2.40665	0.09615	-2.40977
H	0	2.57337	-1.08298	2.44137
C	0	0.18115	0.9172	-0.82468
C	0	-0.54898	1.19981	0.49815
H	0	-0.35139	2.25813	0.7246
O	0	-0.18891	1.23329	-1.93351
C	0	-5.03677	2.89525	-1.23553
C	0	-4.30562	1.82162	-0.43406
C	0	-2.79725	2.05583	-0.38866
C	0	-2.06305	0.98911	0.42171
H	0	-6.11531	2.71449	-1.26246
H	0	-4.70164	1.78859	0.58982
H	0	-4.50382	0.83502	-0.87277
H	0	-2.39124	2.08143	-1.40508
H	0	-2.59755	3.04254	0.05431
H	0	-2.46365	0.9719	1.44486
H	0	-2.26271	-0.00385	-0.00525
H	0	-4.87303	3.88666	-0.79963
C	0	0.17579	0.32012	1.54223
H	0	-0.44196	-0.55009	1.79997
H	0	0.37674	0.85505	2.47558
H	0	-4.67314	2.92365	-2.26795

Zero-point correction= 0.262660 (Hartree/Particle)
Thermal correction to Energy= 0.275670
Thermal correction to Enthalpy= 0.276614
Thermal correction to Gibbs Free Energy= 0.221821
Sum of electronic and zero-point Energies= -579.733900

Sum of electronic and thermal Energies=	-579.720890
Sum of electronic and thermal Enthalpies=	-579.719946
Sum of electronic and thermal Free Energies=	-579.774739

SCF Done: E(RM062X) = -580.185163388
